# The Self-Assembly of Cationic Metal Complexes on Gold Nanoparticle Surface

**DOI:** 10.1021/acsomega.4c04098

**Published:** 2024-06-14

**Authors:** Cássio Roberto Arantes do Prado, Matheus Henrique de Oliveira Pessoa, Lucas da Silva dos Santos, Aline da Silva Xavier da Cruz, Luís Rogério Dinelli, André Luiz Bogado

**Affiliations:** † Instituto de Ciências Exatas e Naturais do Pontal, Universidade Federal de Uberlândia, Ituiutaba, Minas Gerais 38304-402, Brazil; ‡ Instituto de Química, Universidade Federal de Uberlândia, Av. João Naves de Avila 2121, Uberlândia, Minas Gerais 38400-902, Brazil

## Abstract

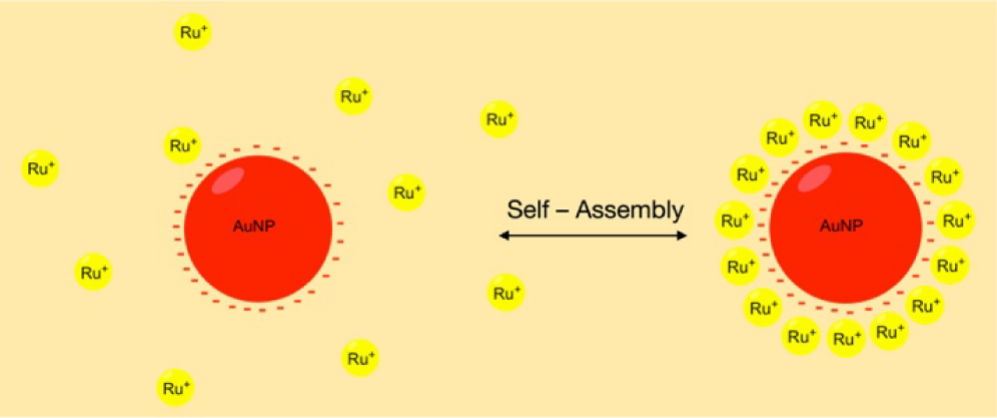

This work aims to study the interaction between cationic metal complexes (M*^z^*^+^) and gold nanoparticles (AuNPs*^z–^*). The M*^z^*^+^ complexes were chosen from previous works described in the literature and were synthesized as defined. For example, they are as follows: **1** = [RuCl(dppb)(bipy)(py)](PF_6_); **2** = [RuCl(dppb)(bipy)(vpy)](PF_6_); **3** = [RuCl(dppb)(bipy)(mepy)](PF_6_); **4** = [RuCl(dppb)(bipy)(*t*bpy)](PF_6_); **5** = [RuCl_2_(dppb)(bipy)](PF_6_); **6** = [Fe(bipy)_3_]Cl_2_; **7** = [Ru(bipy)_3_](PF_6_)_2_; **8** = [TPyP{RuCl(dppb)(bipy)}_4_](PF_6_)_4_; and **9** = [RuCl(*p*-cymene)(Di*i*pmp)](PF_6_). The interactions between M*^z^*^+^ and AuNPs*^z–^* were carried out using conductometry and UV–vis spectroscopy. These experiments allowed determination of kinetic parameters, revealing three different steps in the interaction process: induction time, flocculation, and agglomeration. The self-assembly between M*^z^*^+^ and AuNPs^*z*–^ was investigated using three different models of binding site, namely, Langmuir or direct plot, Benesi–Hildebrand, and Scatchard. These models provide the fraction of total binding sites occupied (θ), the formation constant (*K*_f_), which is dependent on the temperature and geometric structure of each group of M*^z^*^+^, and the Gibbs free energy of reaction (Δ*G*_*r*_), which was negative for each pair of M*^z^*^+^ and AuNPs*^z–^*, revealing a spontaneous agglomeration process. The Hill coefficient (*n*) was 1 for almost all complexes, indicating that agglomeration is an independent process, except for **5**, where *n* = 2, suggesting a positive propensity to bind onto the AuNPs*^z–^* surface. The models have confirmed a noncovalent interaction between these species. The relative error in site binding does not show any variation with changes in the temperature, but a fine-tuning of the *n* value to 1.00 was observed with the increase of the temperature. Finally, the reduction reaction of the 4-nitrophenolate anion (4-NP^–^) by NaBH_4_ catalyzed by AuNPs*^z–^* was used in the presence of M*^z^*^+^ as an evaluation test to show how the M*^z^*^+^ species will disturb the 4-NP^–^ binding site on the surface of gold nanoparticles.

## Introduction

The interaction between cationic metal complexes (M*^z^*^+^) and negatively charged gold nanoparticles (AuNPs*^z^*^–^) is an attractive way to produce new materials.^1,2^ As the basic chemical attributes of the related complexes remain unchanged after agglomeration with AuNPs*^z^*^–^, these materials can be used for different proposes, such as catalyst or electrocatalyst of several organic substrates.^3,4^ Interactions with neutral metal complexes have also been described previously but most often involve a support material or high-cost steps. Daneshvar and coworkers^5^ described a ruthenium hydride complex [RuHCl(PPh_3_)_3_(CO)] immobilized on gold nanoparticles that showed excellent catalytic activity in Suzuki–Miyaura cross-coupling reactions.

Support materials can also be applied to cationic metal complexes to promote an interaction with gold nanoparticles. Kowalska et al.^6^ investigated the effect of interactions between ruthenium complexes, containing phosphonic and carboxylic acid binding groups, AuNPs, and titania. They observed an increase in photocatalytic activity under UV–vis irradiation for the decomposition of acetic acid and dehydrogenation of methanol. Zheng and collaborators^7^ verified that ruthenium complexes [Ru(bpy)_2_(4,4′-(CH_2_PO_3_H_2_)_2_bpy)]^2+^ preadsorbed on titania can induce a change in the size of gold nanoparticles and observed an increase in photocatalytic efficiency in the oxidation reaction of 2-propanol under visible light irradiation.

The main idea of the present work is to provide a self-assembly building process between the negative surface of AuNPs*^z^*^–^ and the positive charge of cationic complexes (M*^z+^*) using accessible techniques. If we consider a nanoparticle as a “macromolecule” (Host) in front of a single cationic metal complex (Guest), then the interaction between them can be investigated by the noncovalent binding behavior between M*^z+^* and AuNPs*^z–^*, using three typical binding models of *host–guest* in supramolecular chemistry: (1) Langmuir isotherm, or the “direct” plot,^8^ (2) Benesi–Hildebrand,^9^ and (3) Scatchard.^10^

Metal nanoparticles, in water solution, applied in the catalytic reduction of 4-nitrophenolate (4-PN^–^) by NaBH_4_ is the most used reaction to test the catalytic activity of these type of materials.^11,12^ This reaction can be easily monitored by UV–vis spectroscopy due to the strong absorption of the 4-NP^–^ anion at λ = 400 nm. The reaction was first observed by Pal^13,14^ and Esumi.^15^ This reaction was used in the presence of M*^z^*^+^ as an evaluation test to show how the M*^z^*^+^ species will disturb the 4-NP^–^ binding site on the surface of gold nanoparticles. It showed an appropriate reaction for testing the catalytic binding site of gold nanoparticles.

## Experimental Section

### Materials and Methods

All chemicals used were of reagent grade or comparable purity, which were supplied and used as received from Aldrich: 4-nitrophenol (NP), NaBH_4_, HAuCl_4_, sodium citrate tribasic, and aminophenol (AP). Gold nanoparticles (AuNPs*^z–^*) were synthesized through the reduction of HAuCl_4_ with a solution of 1% sodium citrate as described by Frens.^16^

The cationic ruthenium complexes (M*^z^*^+^) used in this work to aggregate onto the surface of AuNPs*^n^*^–^ were synthesized as described previously: **1** = [RuCl(dppb)(bipy)(py)](PF_6_),^17^**2** = [RuCl(dppb)(bipy)(vpy)](PF_6_),^2^**3** = [RuCl(dppb)(bipy)(mepy)](PF_6_),^18^**4** = [RuCl(dppb)(bipy)(*t*bpy)](PF_6_),^17^**5** = [RuCl_2_(dppb)(bipy)](PF_6_),^19^**6** = [Fe(bipy)_3_]Cl_2_,^20^**7** = [Ru(bipy)_3_](PF_6_)_2_,^21^**8** = {TPyP[RuCl(dppb)(bipy)_4_}(PF_6_)_4_,^18^ and **9** = [RuCl(*p*-cymene)(Di*i*pmp)](PF_6_).^22^ Regarding the quality of the experiments, the purity and structure of each complex were verified before use, and characterization data are available in Figure S1–S24 and Tables S1 −3).

### Conductometry

Conductometry measurements were carried out using a Mettler Toledo conductivity meter, model FiveEasy FE30, with a platinum electrode model in Lab710 (*K*_cel_ = 0.76 cm^–1^), and automatic room temperature compensation. Inside the conductivity cell, a colloidal suspension of AuNPs*^z–^* (10 mL) was added, and then a stock solution of each complex in acetone (5.3 × 10^–5^ mol L^–1^), from **1** to **7** was added (20 μL for each addition), under magnetic stirring, until the molar conductivity decreased to zero (Ω cm^2^ mol^–1^). The molar conductivity of a colloidal solution of AuNPs*^z–^* in the presence of complexes **1**–**8** is available in Figures S26–33.

### Kinetics of Binding Site

The interaction between cationic metal complexes (M*^z+^*) from **1** to **7** and gold nanoparticles (AuNPs*^z–^*) was investigated using the electronic spectra of absorption in the UV–vis region, which were measured by a Shimadzu model UV-1800 spectrophotometer, coupled with an electrically thermostatic support, model TCC-100, using a quartz cuvette with an optical path of 1 cm. An aliquot of a colloidal suspension of AuNPs*^z–^* (2.5 mL) was added inside the quartz cell, and from here two different approaches were adopted: (i) addition of 100 μL of a stock solution in acetone of each complex (5.3 × 10^–5^ mol L^–1^), from **1** to **7**, after 2 min. An electronic spectrum of absorption was recorded after each addition. This procedure was repeated three times in the temperature range from 20.0 to 35.0 °C. (ii) addition of only one aliquot (100, 150, 200, 250, 300, 500, or 1000 μL) of a stock solution in acetone of each complex (5.3 × 10^–5^ mol L^–1^), from **1** to **7**, at constant temperature of 25. °C. This assay was also repeated three times for each complex and temperature. The chosen wavelength in the visible range was λ = 625 nm, which allowed the observation of three different stages, here called induction time, flocculation, and agglomeration. The data kinetics of M*^z+^* and AuNPs*^z–^* in the presence of complexes **1**–**8** is available in Figures S34–S61 andTables S4–S9.

### Self-Assembly between M*^z+^* and AuNPs*^z–^*

Three different mathematical models of binding were applied to investigate the interaction between cationic metal complexes (M*^z+^*) and gold nanoparticles (AuNPs*^z–^*), which were as follows: (A) Langmuir or direct plot,^8^ (B) Benesi–Hildebrand,^9^ and (C) Scatchard.^10^ The agglomerates M*^z+^*/AuNPs*^z–^* were prepared in a quartz cell (1 cm length) with a colloidal solution of AuNPs*^z–^* (2.5 mL), and addition of each complex in acetone solution (50 μL, 1.0 × 10^–5^ mol L^–1^), until the species precipitates or fills the cuvette volume (4 mL). Each new addition and absorbance measurement at λ = 625 nm were carried out after 3 min. Under these conditions, the fraction of total binding sites occupied (θ), was obtained as follows,
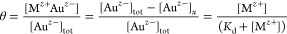
where [M^*z*+^ Au^*z*–^] represents the concentration of M*^z+^*/AuNPs*^z–^* agglomerates, [Au^*z*–^]_tot_ the initial concentration of AuNPs*^z–^*, [Au^z–^]*_x_* the concentration of AuNPs*^z–^* on time, and *K*_d_ is the dissociation constant. An expanded demonstration of θ can be seen in the Supporting Information as well as the isotherm data of Langmuir (Figures S62–S86), Benesi–Hildebrand (Figures S87–S111), and Scatchard (Figures S112–S136) for each interaction among complexes **1–9** with AuNPs*^z–^*.

### Reduction of 4-Nitrophenol

The 4-nitrophenol reduction (4-NP) was carried out similar to that described by Pal and coworkers, using silver nanoparticles^13^ as a catalyst. Instead of applying AgNO_3_ solution, which produces silver nanoparticles under reducing conditions, here a colloidal solution of gold nanoparticles (AuNPs*^z–^*) prepared as described by Frens^16^ was applied. Because AuNPs*^z–^* has no molar mass, various efforts were made to find the ideal volume of the colloidal suspension to catalyze the reduction of 4-NP. A volume of 48 μL was found to be the best. The remainder of the experiment was carried out as described by Pal T.,^13^ and a brief description follows. Distilled water (1.85 mL) was added in a quartz cell with 1 cm of optic path with subsequent addition of 4-nitrophenol (100 μL; 1.2 × 10^–3^ mol L^–1^), as substrate, and NaBH_4_ (50 μL; 0.1 mol L^–1^), as a reducing agent. The production of 4-nitrophenolate (4-NP^–^) is observed rapidly, providing a characteristic band at 400 nm. After that, AuNPs*^z–^* (48 μL) was added inside the quartz cell, starting the reduction reaction. UV/vis were recorded on a Shimadzu spectrophotometer, model UV-1800, coupled to TCC-100 temperature-controlled cell (at 25.0 ± 0.1 °C), scanning the wavelength between 200 and 800 nm.

The reduction reaction of 4-NP^–^ was also carried out in the presence of the complexes **1**. The complexes were dissolved in methanol, in different concentration to reach a θ regime between 0.3–0.6 and added in the colloidal suspension of gold nanoparticles at 35 °C. In this case, the fraction of total binding sites occupied (θ), was used to control the amount of M*^z+^* onto surface of AuNPs *^z–^*.

## Results and Discussion

### Kinetics of Binding Site

**Figure 1:**
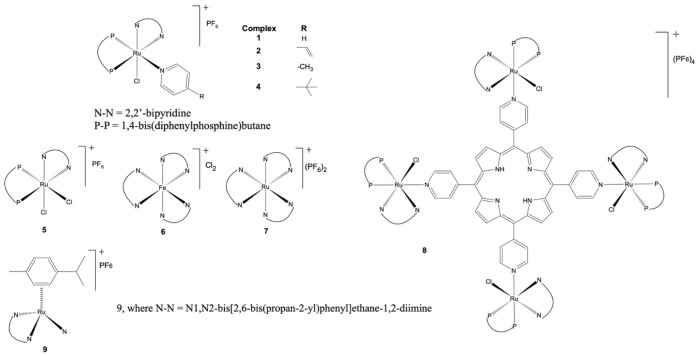
General structure and abbreviation of the target complexes (M^*z*+^).

The Interaction process between a colloidal suspension of gold nanoparticles (AuNPs*^z–^*) and a homogeneous solution of a cationic metal complexes M^z+^ can be followed by conductometry, UV/vis, SEM and TEM images.^1−4^ In the present work, nine cationic metal complexes were selected, with different charges, functional groups, and structures to interact with the surface of gold nanoparticles (Figure 1).

Conductometry is a cheap and accessible way to observe this type of interaction, monitoring the decrease in the molar conductivity of a colloidal solution of AuNPs*^z–^* when cationic metal complexes (M*^z+^*) are added. The concentration of M^*z*+^ was defined by mol L^–1^ or g L^–1^, and an idea of the number of cationic species, from **1** to **8**, to neutralize the anionic surface of AuNPs*^z–^* can be achieved (see Table 1).

**Table 1:** Number of M^*z*+^ Species to Neutralize 10 mL of a Colloidal Suspension of AuNPs^*z*–^ with a Plasmon Band Centered at λ = 520 nm

complex	ionic charge of each complex	number of molecules until Λm = 0 (Ω cm^2^ mol^–1^) × 10^18^
**1**	+1	6.2
**2**	+1	6.1
**3**	+1	5.8
**4**	+1	6.9
**5**	+1	5.8
**6**	+2	94.5
**7**	+2	92.1
**8**	+4	4.3

The number of M^*z*+^ to neutralize the molar conductivity of 10 mL of AuNPs*^z–^*, with a plasmonic band centered at λ = 520 nm, was found in the scale of 10^18^ molecules (Table 1). Graphs of molar conductivity against the concentration of each M^*z*+^ described in Table 1 are available inFigures S26–S33).

The complexes with a charge of +1, from **1** to **5**, have shown a similar behavior in neutralizing the negative charge of AuNPs^*z*–^. The number of molecules up to Λm = 0 (Ω cm^2^ mol^–1^) was close for complexes from **1** to **5,** regardless of the steric hindrance or stereochemistry of each complex. In the case of the complexes with charge +2, a slightly higher order of magnitude was observed for **6** and **7** when compared to the complex with charge +1. This behavior corroborates the results of the formation constant (*K*_f_), which will be presented in the next section. Complexes **6** and **7** have shown the highest *K*_f_ values among the target complexes. The number of molecules to neutralize the negative charge on the surface of AuNPs^*z*–^ and *K*_f_ is intrinsically linked to the charge of the guest molecule. The higher the charge, the higher the values. However, when the charge and the mass of the guest molecule both increase, the values are no longer comparable. For example, complex **8** has a charge +4 and is a macrocycle with the highest molecular mass among the complexes used in this work. The charge +4 comes from four ruthenium complexes coordinated in the peripheral environment of a porphyrin cycle (see Figure 1). Complex **8** has a totally different structure than the others, which are typical mononuclear complexes of the Werner type. It is worth mentioning that the *K*_f_ value for **8** was also the lowest observed value.

The data obtained from this protocol are susceptible to the concentration and temperature variation, so a protocol using temperature-controlled UV/vis spectroscopy was developed to study the interaction between M^*z*+^ and AuNPs^*z*–^, which easily provided kinetic parameters.

**Figure 2:**
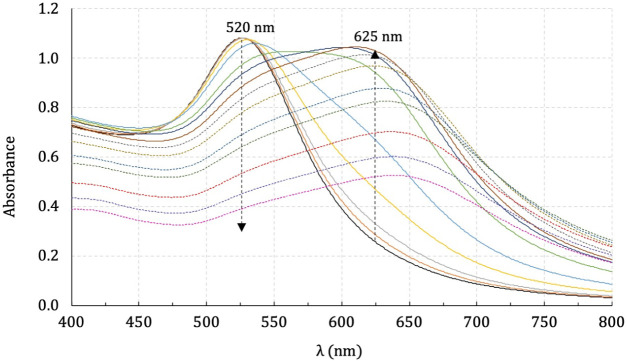
Interaction of AuNPs^*z*–^ and [RuCl_2_(dppb)(4-Mebipy)]^+^**3** accompanied by UV/vis.

Figure 2 shows initially a plasmon band centered at 520 nm for the colloidal suspension of AuNPs*^z–^* (2.5 mL), due to the characteristic Mie resonance for these kind of nanoparticles. An addition of 100 μL of a stock solution, every 2 min, to acetone of each complex (5.3 × 10^–5^ mol L^–1^) was done until 2200 μL. As a result, it causes a rapid increase in a broadened band, centered at around 625 nm, which then decays exponentially (dashed lines) followed by a bathochromic effect (Figure 2 represents an assay using [RuCl_2_(dppb)(4-Mebipy)]^+^). This red shift of the original plasmon band is attributed to a longitudinal coupling of plasmon absorbance of the nanoparticles.^23,24^

The nature of the coupled plasmon band is dependent on the temperature and concentration of cationic specimens, labeled herein as guest molecules. In a blank test, adding acetone without complex, under the same condition, has shown a hypochromic effect over the plasmon band (Figure S25). It was not observed on any other band in the range of 400–800 nm.

Whitesides and coworkers^25^ described the interaction between gold nanoparticles and cationic species in three stages: First, ther is *flocculation*, which is the instability of colloidal dispersions, followed by *agglomeration*, for the cases involving reversible association of nanoparticles and finally an *aggregation*, for the cases involving an irreversible association.

A similar approach was applied to describe the interaction between cations of coordination metal complexes (M*^z+^*) and AuNPs*^z–^*, such as the following: (a) *induction time*: is a time interval needed to start the process of noncovalent interaction; (b) *flocculation*: it is a process in which suspended particles clump together because the attractive forces between them overcome any repulsive forces, increasing the instability of colloidal dispersions; (c) *agglomeration*: is a particle size enlargement in which the guest molecules are joined in an assembly, involving reversible association.

**Figure 3:**
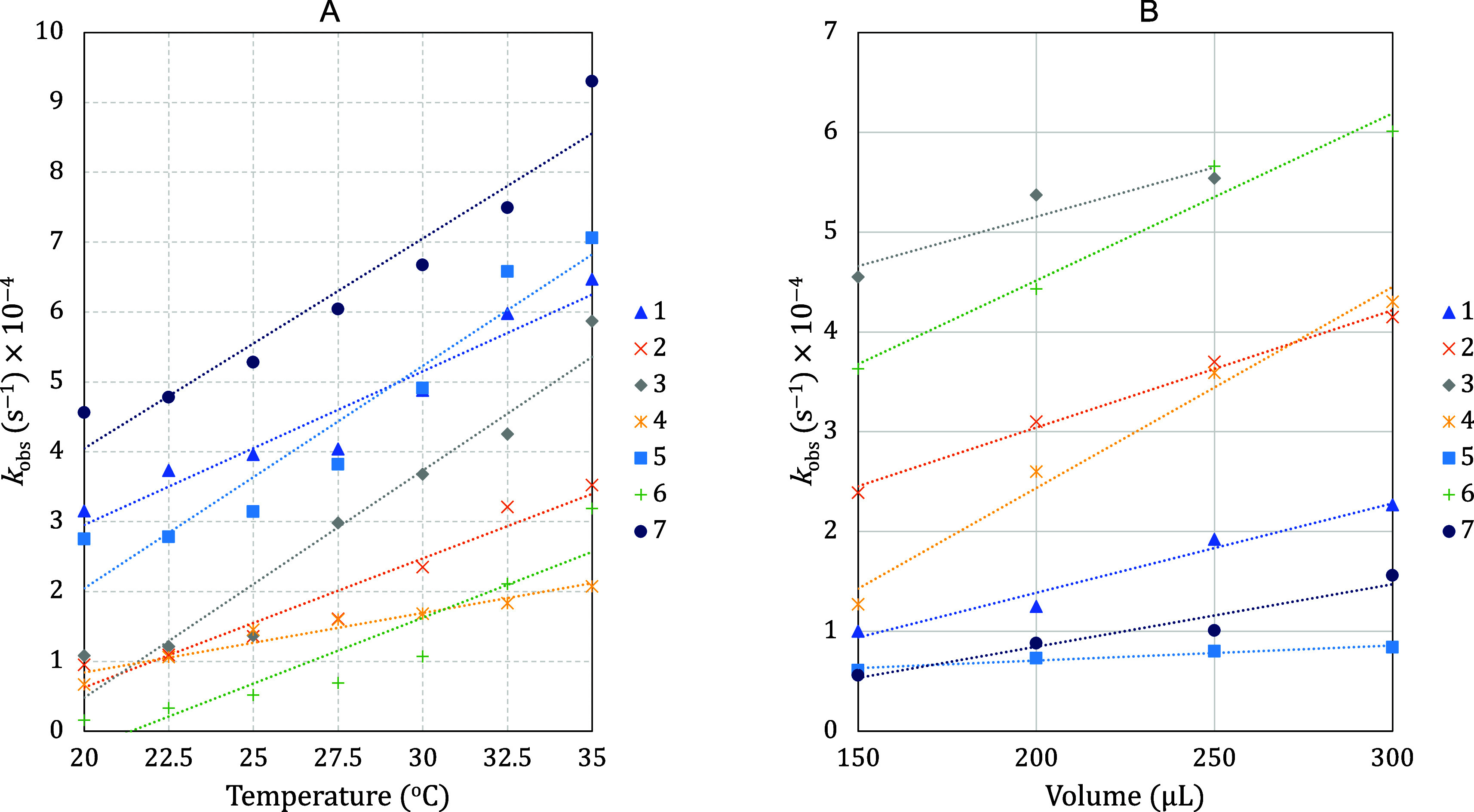
*k*_obs_ (s^–1^) for the band decay at 520 nm, using complexes from **1** to **7**, as a function of temperature variation (A) and volume variation of 5.3 × 10^–5^ mol L^–1^ M^z+^ solution (B).

**Figure 4:**
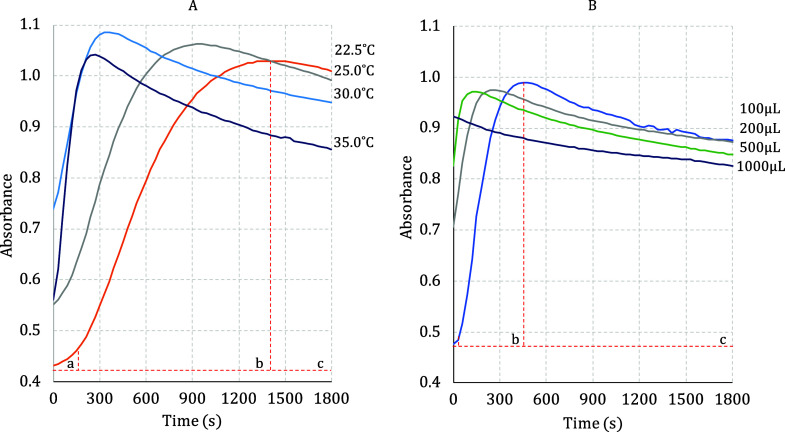
Time dependence of the coupled plasmon band of AuNPs^*n*–^ (2.5 mL) at 625 nm. (A) In the presence 100 μL from a stock solution of [RuCl_2_(dppb)(4-Mebipy)]^+^**3** in acetone (5.3 × 10 mol L^–1^) at temperature range of 22.5–35.0 °C. (B) In the presence of 100–1000 μL of [RuCl_2_(dppb)(4-Mebipy)]^+^ in acetone (5.3 × 10 mol L^–1^) added in only one portion at 25.0 °C. **a** = induction time, **b** = flocculation and **c** = agglomeration.

In a previous work, it was demonstrated that the interaction product can be isolated in a powder form and redissolved in an appropriate solvent.^3^ It was also demonstrated by SEM and TEM images where the powder contained spherical gold nanoparticles radially enlarged by cationic ruthenium complexes.^1,2^ Therefore, the term agglomeration seems to be a better phrase to describe the last step here, which is the reversible noncovalent interaction between gold nanoparticles and cations of coordination metal complexes. All these stages are dependent on the temperature and the concentration of the guest molecule, as depicted in Figures 3 and 4.

To explore the kinetic behavior of the experiment described in the Figure 2, two different approaches were conducted: (i) For each selected temperature from 22.5 to 35.0 °C was added a constant aliquot of M^*z*+^ in acetone solution (100 μL; 5.3 × 10^–5^ mol L^–1^) into colloidal suspension of AuNPs^*z*–^ (2.5 mL) until 2200 μL. (ii) At constant temperature of 25 °C, only one aliquot of 100, 150, 200, 250, 300, 500, or 1000 μL of each M^*z*+^ in acetone solution (5.3 × 10^–5^ mol L^–1^) was added.

These experiments show changes in two wavelengths, 520 and 625 nm, with at least two different phenomena reported at 625 nm, namely flocculation and agglomeration. Kinetic plots at 520 and 625 nm are available in Figures S34–S47 for the first setup and in Figures S48–S61 for the second setup. The induction period in a few cases was too fast to be measured under the experimental conditions, making it more applicable at low temperatures, where the kinetic energy of the related species is lower. Therefore, the rate constants were not measured during this stage.

In general, the rate constant (*k*_obs_) related to the consumption of the band centered at 520 nm, increases with the increasing temperature or concentration of the guest molecule from **1** to **7** (Figure 3). These results represent decreases in the concentrations of free AuNPs^*z*–^ available in solution. Tables S4 and S7 summarize the observed rate constant for each run. It was not possible to establish a correlation with the structures of the M^*z*+^ species and the tendencies observed in Figure 3.

Figure 4 represents the graphical situation of each set up at 625 nm using complex **3** as the guest molecule, illustrating the induction time, flocculation, and agglomeration periods. It is possible to observe a trend where the rate of the flocculation period increases faster than the agglomeration period. Regardless of the change in the temperature or concentration of the guest molecule, this behavior was observed for each selected complex, from **1** to **7** (Tables S5 and S6 due to temperature variation and Tables S8 and S9 due to concentration variation). The rate constant obtained for flocculation and agglomeration periods increases with the increasing of the concentration of the guest molecule and temperature, acting under pseudo-first order condition in two different situations.

### Self-Assembly between M^*z*+^ and AuNPs*^z-^*

The process of the noncovalent interaction between cations of coordination metal complexes (M^*z*+^) and the negative surface of gold nanoparticles (AuNPs*^z–^*) can be defined as a reversible chemical reaction, observed experimentally, in either forward or reverse direction described as follows:
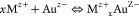
where Au^*z*–^ represents the unliganded gold nanoparticles, M^*z*+^ is the cation of the coordination metal complexes that binds to Au^*z*–^, and M^*z*+^_*x*_Au^*z*–^ are the agglomerates. Then, three consistent expressions for ligand-binding experiments, using either the association or the dissociation reactions, were applied to describe the reversibility interaction between M^*z*+^ and AuNPs*^z–^*. Table 2 summarizes the extrapolation of each model.

**Table 2:** Extrapolation of Each Multi-Site Binding Models^i^^8^

model	equation	plot	extrapolation
Langmuir		θ vs [M^z+^]	,
Benesi–Hildebrand			,
Scatchard			*n* = *x*-intercept,

i [M^*z*+^] = concentration (g L^–1^) of each cation of coordination metal complex; *n* = Hill coefficient; *K*_d_ = dissociation constant; *K*_f_ = formation constant; θ = fraction of the total binding sites occupied.

**Figure 5:**
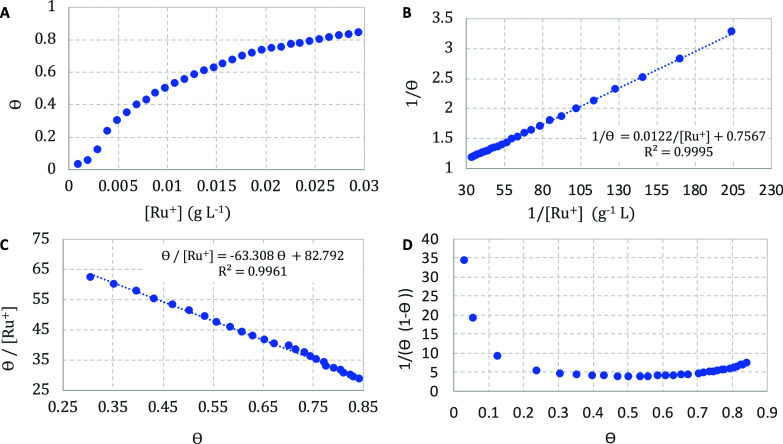
Three representations of ligand-binding isotherms: (A) Langmuir; (B) Benesi–Hildebrand; (C) Scatchard; and (D) approximate relative error in one-site binding for addition of [RuCl(dppb)(bipy)(py)]^+^**1** in a colloidal solution of AuNPs^*n*–^.

Figure 5 summarizes the representation of ligand-biding isotherms defined in Table 2, regarding the addition of constant aliquots of [RuCl(dppb)(bipy)(py)]^+^ (50 μL, 1.0 × 10^–5^ mol L^–1^) in a colloidal solution of AuNPs*^z–^* (2.5 mL). Each new addition and absorbance measurement at λ = 625 nm were carried out after 3 min.

These models provide the dissociation constant (*K*_d_), formation constant (*K*_f_ = 1/*K*_d_) and Hill coefficient (*n*) for each complex as presented in Table 3. A plot of θ vs [M^*z*+^] at a constant concentration of AuNPs*^z–^* will yield a hyperbola whose midpoint will provide *K*_d_. This nonlinear curve is known as the Langmuir isotherm, and further manipulation of that yields two linear forms that are more accessible to obtain the *K*_d_ and the Hill constant (*n*): the double-reciprocal plot called the Benesi–Hildebrand^9^ binding curve, and the x-reciprocal called the Scatchard plot,^10,26^ All graphs, for each M^*z*+^, relating to the three models are available in triplicate in the Supporting Information (Langmuir = Figures S62–S86, Benesi–Hildebrand = Figures S87–S111, and Scatchard = Figures S112–S136).

**Table 3:** *K*_f_, *n,* and Δ*G*_*r*_ Values Using Benesi–Hildebrand and Scatchard Models at 25°C^i^

	Benesi–Hildebrand			Scatchard		
complex	*K*_f_ (**±**SD)	*n* (**±**SD)	Δ*G*_*r*_(kJ mol^–1^)	*K*_f_ (**±**SD)	*n* (**±**SD)	Δ*G*_*r*_(kJ mol^–1^)
**1**	68 (3)	1.32 (0.03)	–10.6	64 (1)	1.32 (0.04)	–10.3
**2**	61 (5)	1.35 (0.03)	–10.2	60 (4)	1.34 (0.03)	–10.1
**3**	67 (4)	1.33 (0.04)	–10.4	65 (5)	1.34 (0.06)	–10.3
**4**	81 (7)	1.19 (0.03)	–10.8	74 (7)	1.20 (0.03)	–10.7
**5**	34 (2)	1.86 (0.04)	–8.7	31 (2)	1.84 (0.05)	–8.5
**6**	485 (37)	1.13 (0.07)	–15.3	445 (6)	1.11 (0.06)	–15.1
**7**	415 (32)	1.21 (0.03)	–14.9	396 (33)	1.20 (0.03)	–14.8
**8**	20 (2)	1.01 (0.05)	–7.5	19 (2)	1.02 (0.03)	–7.3
**9**	239 (15)	1.32 (0.03)	–13.6	290 (14)	1.27 (0.03)	–14.0

i **1** = [RuCl(dppb)(bipy)(py)](PF6); **2** = [RuCl(dppb)(bipy)(vpy)](PF6); **3** = [RuCl(dppb)(bipy)(mepy)](PF6); **4** = [RuCl(dppb)(bipy)(tbpy)](PF6); **5** = [RuCl2(dppb)(bipy)](PF6); **6** = [Fe(bipy)3]Cl2; **7** = [Ru(bipy)3](PF6)2; **8** = {TPyP[RuCl(dppb)(bipy)4}(PF6)4; and **9** = [RuCl(p-cymene)(Diipmp)](PF6). SD = standard deviation.

The results have shown a spontaneous agglomeration between gold nanoparticles and selected cationic complexes since the changes in Gibbs free energy were all negative (see Table 3). There are similarities among the results when comparing the Benesi–Hildebrand and Scatchard models, which allows a detailed discussion.

Complexes **1**–**4** have an analogous coordination geometry, so it is plausible that the values of *K*_f_ and *n* are close to each other. This indicates that a change in the periphery of the ligands, in this sort of structure, will not affect how these guest molecules bind to the surface of the gold nanoparticles.

Complexes **6** and **7** have also given a close behavior to bind on the surface of the gold nanoparticles, but they have shown higher *K*_f_ values when compared with complexes **1**–**4**. The structures of complexes **6** and **7** are also similar but distinct from the others, with three bipyridine coordinated in an octahedral geometry around a group-8 metal, Fe, and Ru, respectively. However, complexes **6** and **7** have a charge +2, while complexes **1**–**4** are monocations. It suggests that an increase in the positive charge of the complex can improve the *K*_f_ values.

When the data of complex **7** are compared with those of **1,** the *K*_f_ value increases by 7-fold. In this case, higher values of *K*_f_ were also accompanied by higher values of the rate constant, when the concentration of **7** increased. There is no step of the coupled plasmon band when an aliquot of this complex was added in a colloidal suspension of gold nanoparticles (see Tables S8 and S9). It means that the agglomeration stage was directly observed, suggesting a strong bond between complex **7** and the surface of gold nanoparticles. It is worth mentioning here that the number of species up to Λm = 0 (Ω cm^2^ mol^–1^) with **6** and **7** also provided the highest values (Table 1).

Controversially, complex **8,** which is a macrocycle with charge +4, presented the lowest value of *K*_f_ among the complexes studied, only 20 ± 2. It seems that the size of the guest molecule is also important for the flocculation step, as it must have impaired electronic mobility due to the high mass. Moreover, the charge +4 in complex **8** is distributed across four ruthenium complexes, symmetrically distributed at the peripheral environment of a porphyrin cycle (see Figure 1). It must be considered before correlating the *K*_f_ of **8** with the others, which are mononuclear complexes.

Complex **9** has shown a strong interaction with AuNPs*^z–^*, with *K*_f_ = 239 and 290 for the Benesi–Hildebrand and Scatchard models, respectively. It seems that more nucleophilic organometallic compounds have a greater predilection to interact with AuNPs*^z–^* than classic coordination metal complexes.

In such experiments, it was assumed that AuNPs*^z–^* has only one binding site, where it should be uniquely noncovalent by electrostatic interaction with M*^z^*^+^ species. Otherwise, a covalent bond between AuNPs*^z–^* and a guest molecule or a metal–metal bond, such as Ru–Au, would be considered as another sort of binding site. Then, in a simplest case, where only one binding site exists per AuNPs*^z–^*, the Hill coefficient (*n*) was found to be approximately equal to 1; indicating that agglomeration is an independent process, i.e., the presence of other guest molecules does not affect the binding process.

As can be seen in Table 3, the Hill coefficient was found to be close to 1 for almost all complexes, regardless of the method used, i.e., Benesi–Hildebrand or Scartchard. Only complex **5** presented a higher *n* value, close to 2, suggesting a positive cooperativity between different binding sites, but it will be described here as a nonspecific phenomenon. However, according to this result, the highest value of *n* of **5** does not indicate a stronger interaction with AuNPs*^z–^* since the value of *K*_f_ was one of the lowest observed among the applied complexes. Complex **5** has a Ru^3+^ ion as its metal center, and it is reasonable to think that an interaction with AuNPs*^z–^* might follow a different path.

The appropriate complexation binding ranges between M*^z+^* and AuNPs*^z–^* can be explored by the work described by Weber and coworkers.^27^ It is possible to determine the concentration of M*^z^*^+^ and AuNPs*^z–^* that will allow achieving adequate binding between these specimens by plotting a curve as illustrated in Figure 5D. The probability (*p*) of binding can be defined as the ratio of the concentration of the agglomerate [M*^z+^*Au*^z–^*], divided by the concentration of the minor component added, either [AuNPs*^z–^*]_0_ or [M*^z+^*]_0._
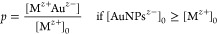

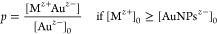


**Figure 6:**
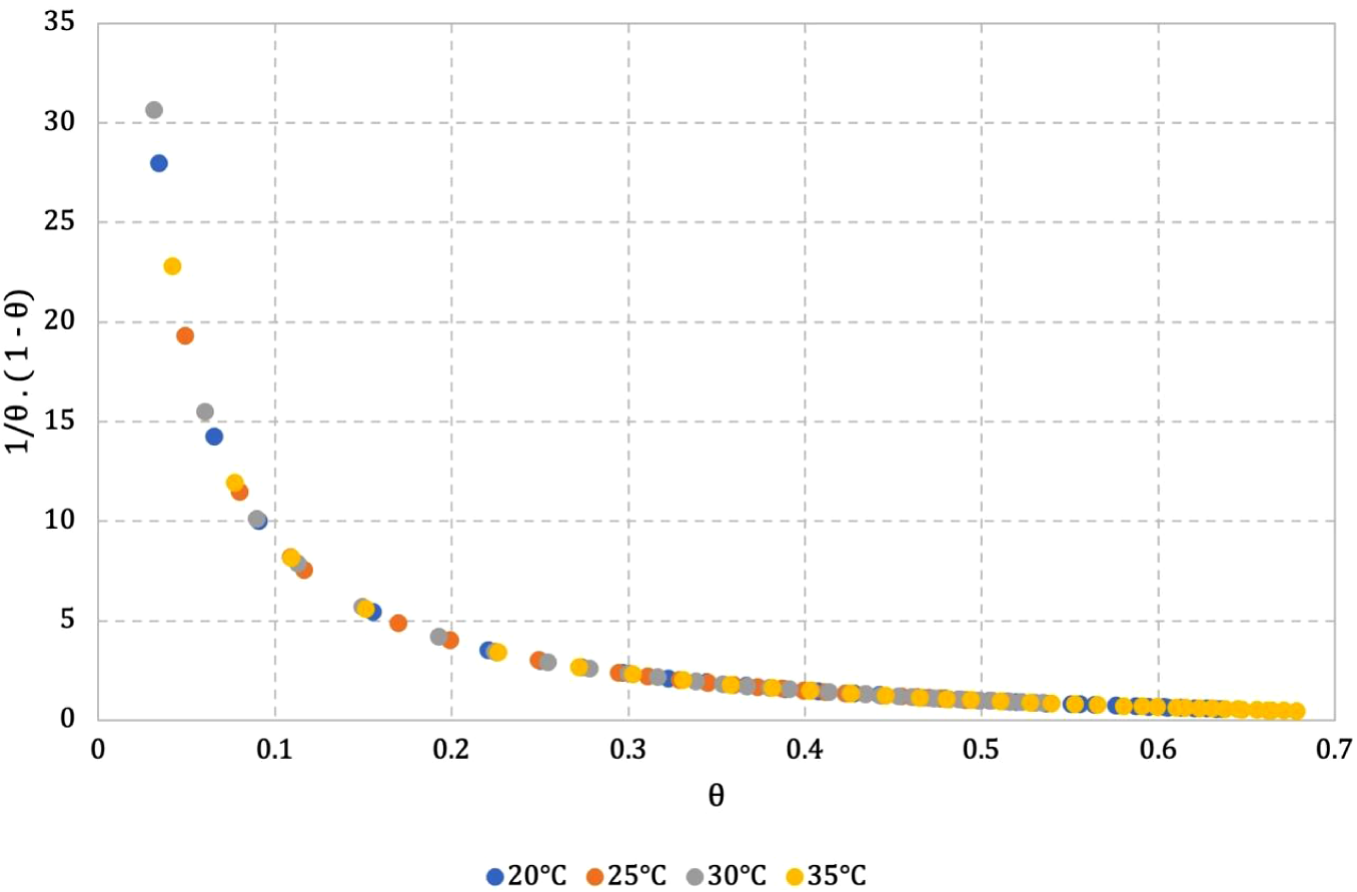
Temperature dependence on the relative error in one-site binding for AuNPs^*z*–^ as the host and [RuCl(p-cymene)(Diipmp)](PF_6_) **9** as the guest.

The sharp increase at the beginning and the end of the curve significantly limits the observable effective binding.^8^ According to Figure 5D, the relative error in *K*_d_ for [RuCl(dppb)(bipy)(py)]^+^ is minimized when θ is within 30 to 70% regime, when the concentration of the gold nanoparticles was kept constant. In fact, this result was observed for almost all complexes, except for complex **9**, which does not increase the value of approximate relative error at its high concentration, since agglomeration is achieved when θ = 0.68 at 35 °C (Figure 6).

In the initial points, instability is due to the probability of lesser formation of agglomerates since the concentration of the complex is low. At the end points, instability is caused by the excess of the complex, resulting in the formation of precipitates.

The relative error in site binding does not show any variation with changes in the temperature (Figure 6 and Table 4) for all complexes used as guest molecules in this work. Plots of approximate relative error for each complex are available in Figures S137–S161.

**Table 4:** Equilibrium Constants Observed by Benesi–Hildebrand and Scartchard Models Under Temperature Variation, Using [RuCl(*p*-cymene)(Diipmp)](PF_6_) **9** as a Guest Molecule

	Benesi–Hildebrand			Scartchard		
temperature (°C)	*n*	*K*_d_ × 10^–3^	*K*_f_	*n*	*K*_d_ × 10^–3^	*K*_f_
20	1.83	4.6	218	1.78	4.4	229
25	1.35	4.1	239	1.31	4.0	249
30	1.28	3.5	289	1.27	3.4	290
35	1.00	1.5	669	0.99	1.4	699

It is interesting to observe in Table 4 a decrease in the *K*_d_ values with increasing temperature and a simultaneous fine-tune of the *n* value to 1.00, while the relative error is kept constant in the temperature range from 20 to 35 °C (Figure 6). This remarkable result shows that researchers always observe an effective binding if the θ regime is projected to the minimum value of 1/θ (1−θ) (*y*-axis in Figures 6and 5D or Figures S137–S161) regardless of temperature. However, at the set temperature of 35 °C, the binding process involving **9** and AuNPs*^z–^* is completely independent, and *K*_d_ is equal to the M*^z+^* concentration when half of the binding sites are filled, i.e., θ = 0.5.

## Reduction of 4-Nitrophenol

### Gold Nanoparticles as Catalysts

**Figure 7:**
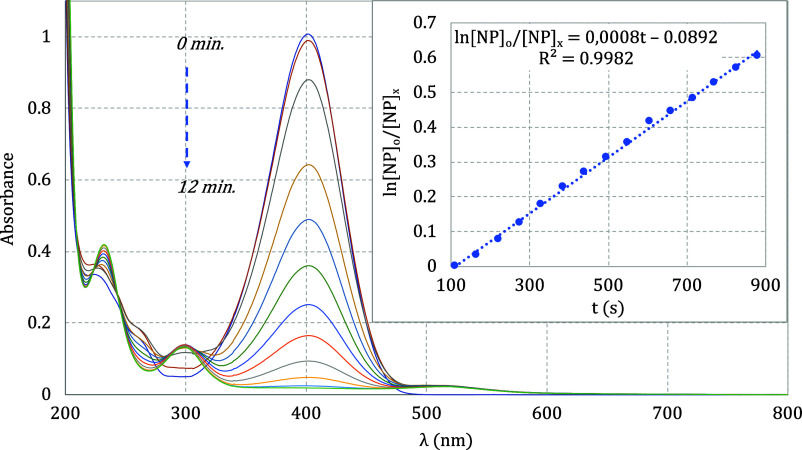
Reduction of 4-nitrophenolate by NaBH_4_ catalyzed by AuNPs^*z*–^ is accompanied by UV–vis spectra. Inside: Pseudo-first order law at 301.65 K.

The reduction reaction of 4-nitrophenol (4-NP) with NaBH_4_ was carried out in water solution, immediately producing 4-nitrophenolate (4-NP^–^). Then, gold nanoparticles (AuNPs*^z–^*) were added to the system as a catalyst to catalyze the reduction reaction. The time dependence was monitored by UV–vis at 400 nm, decreasing the absorbance from 1.0 to 0 in 12 min (Figure 7). The rate law agrees with a pseudo-first order since the NaBH_4_ amount is 80-fold higher than the 4-NP (see Figure 1). On this condition, *k*_obs_ was 3.7 × 10^–3^ s^–1^ within *t*_1/2_ = 189 s. The *k*_obs_ values are not linear in the temperature range of 25–50 °C (see Table S10). Similar behavior was observed for gold nanoparticles encapsulated in microgels and used as catalysts for the reduction of hexacyanoferrate(III) in the presence of NaBH_4_.^11^ Therefore, the effect of temperature variation over *k*_obs_ was investigated in a linear range between 28.5 and 36.0 °C (see Table 5). The results provided values of *E*_a_ = 173 k*J* mol^–1^ and *A* = 6.6 × 10^26^ s^–1^ from an Arrhenius plot (ln *k*_obs_ vs 1/T), and Δ*H*‡ = 170 kJ mol^–1^ and Δ*S*‡ = 260 J K^–1^ (Δ*S*‡/R = 31.27 *e*.*u.*) from Eyring plot (ln *k*_obs_ vs 1/T) (see Figures S162 and S163 respectively). These results agree with a previously published work^28^ and suggest that the reduction reaction, onto surface of AuNPs*^z–^* has an activated state highly entropic in a dissociative pathway.

**Table 5:** Rate Constant and Half-Life at the Temperature Range between 28.5 and 36 °C^i^

temperature (°C)	*k*_obs_ (s^–1^) x 10^–3^	SD × 10^–4^	*t*_1/2_ (s)
28.5	0.8	± 0.3	866
30.0	1.1	± 1.5	619
31.5	1.4	± 0.6	510
34.5	2.8	± 3.6	244
36.0	4.1	± 1.2	169

i SD = standard deviation.

### A Binding-Site Perturbation

The reduction reaction of 4-NP by NaBH_4_ catalyzed by AuNPs*^z–^* to produce 4-aminophenol (4-AP) was used in the presence of M*^z^*^+^ as an evaluation test to show that 4-NP^–^ and M*^z^*^+^ species interact with the same binding site onto the surface of gold nanoparticles, i.e., the negative surface of AuNPs*^z–^*.

The outcomes, presented in the above sections, have supported a precise way of controlling the amount of M*^z+^* onto the surface of gold nanoparticles by noncovalent binding. The complex [RuCl(dppb)(bipy)(py)]^+^ (**1**) was chosen to be applied in this experiment.

**Figure 8:**
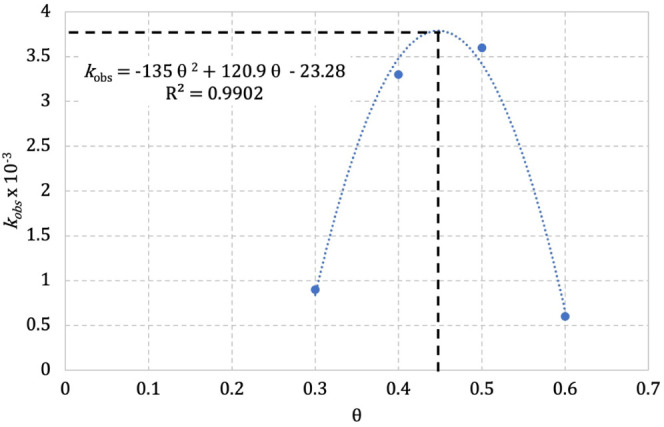
*k*_obs_ for the reduction of 4-nitrophenol at 35 °C, within θ regime between 0.3 and 0.6 for **1** and AuNPs^z–^ as a catalyst.

In this sense, the rate constant for the reduction of 4-NP by NaBH_4_ was determined at 35 °C, with different values of θ for **1** and AuNPs*^z–^*. The range of 30–60% of the covered nanoparticle surface was chosen based on the data in Figure 5A and D. In such conditions, the rate constant increase until a maximum with θ = 0.45, then immediately dropped (Figure 8). Table 6 summarizes the results under the selected coverage scheme.

**Table 6:** *k*_obs_ and *t*_1/2_ for Reduction of 4-Nitrophenol Using Variable θ for **1** and AuNPs^z–^ Interaction^i^

θ	*k*_obs_ (s^–1^) × 10^–3a^	*t*_1/2_ (s)
0.3	0.9	770
0.4	3.3	210
0.5	3.6	192
0.6	0.6	1155

i a = T = 35°C.

**Figure 9:**
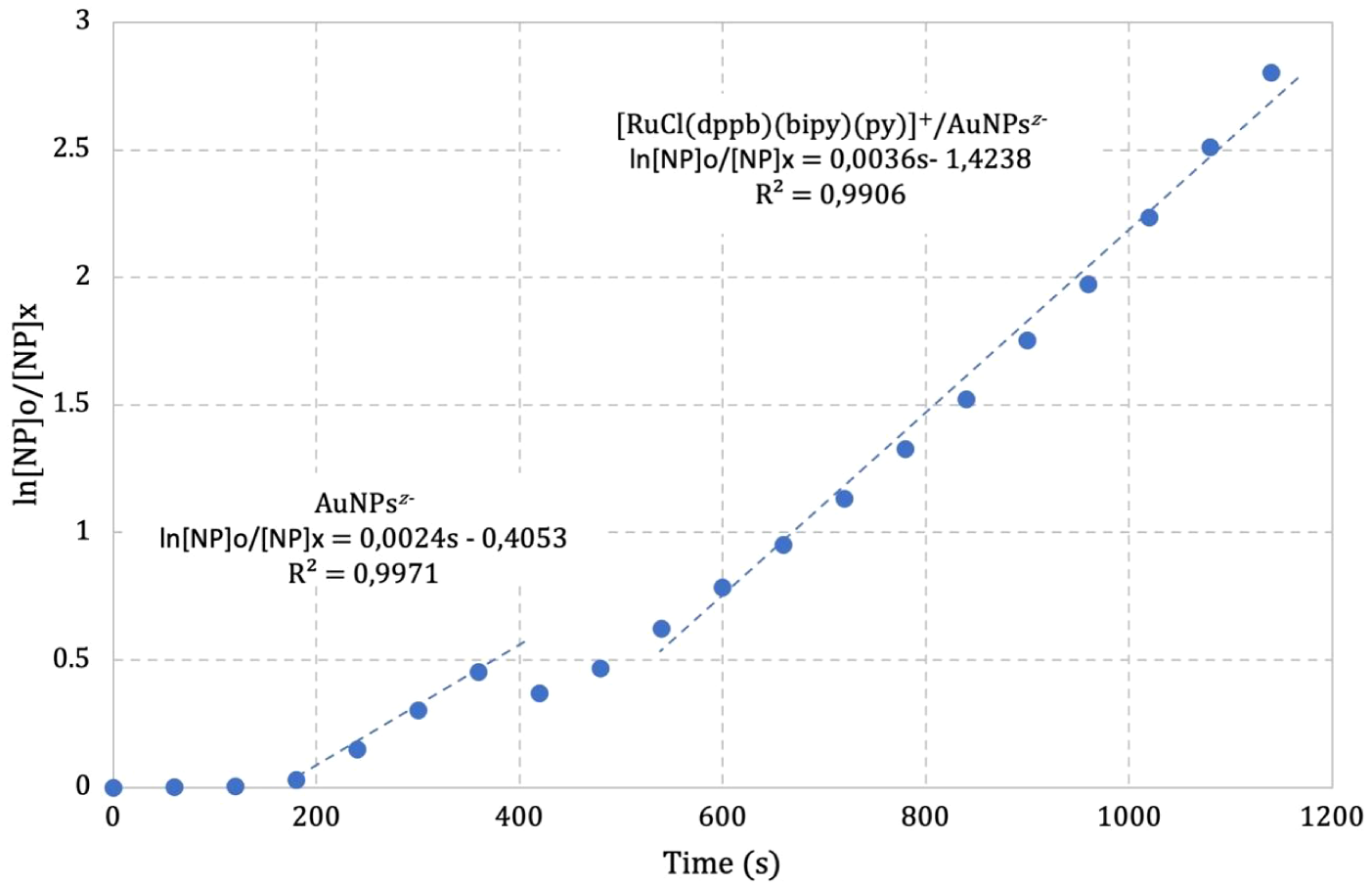
Pseudo-first order law at 35 °C to the reaction between 4-NP and NaBH_4_ catalyzed by AuNPs^*z*–^ until 360 s and in the presence of **1**.

A relationship between θ and *k*_obs_ was recognized from Figure 8, which was assumed to be a cooperative or noncooperative partnership between **1** and AuNPs*^z^*^–^. An inhibition of the catalytic activity of AuNPs*^z–^* was observed before and after 45% surface coverage with **1**, but at θ = 0.45 the catalytic activity was practically the same for AuNPs*^z–^* alone, *k*_obs_ = 3.6 × 10^–3^ s^–1^. An additional assay was carried out at the maximum value of θ in Figure 8 to confirm this observation. The reduction reaction of nitrophenol by NaBH_4_ was started in the presence of AuNPs*^z–^* as a primary catalyst, then **1** (7.83 × 10^–3^ g L^–1^) was added after 360 s reaching θ = 0.45 (Figure 9). A subtle decrease in the rate constant was observed, which was considered as an induction time to agglomerate the species, i.e., M*^z^*^+^, AuNPs*^z–^*, and 4-NP^–^. It is interesting to note that the rate constant continues to increase from the reaction initiated only with AuNPs*^z–^* in the presence of **1**; *k*_obs_ was 2.4 × 10^–3^ to 3.6 × 10^–3^ s^–1^. There is no evidence of an inhibition of the catalytic activity under these conditions, suggesting a synergistic effect of cooperation between **1** and AuNPs^z–^ when θ = 0.45 at 35 °C. The most important result here is not how much catalytic activity increases in the presence of **1**, but the possibility of modulating cooperativity or noncooperativity behavior by changing the θ ratio.

## Conclusion

A self-assembly between cationic metal complexes and gold nanoparticles was investigated using three different models of binding, namely, Langmuir or direct plot, Benesi–Hildebrand, and Scatchard. The models have confirmed a noncovalent interaction between these species, which were labeled here as agglomerates. This process was accompanied by UV–vis at controlled temperature, producing a fast increase of an enlarged band with concomitant red shift of the original plasmon band of gold nanoparticles. The nature of this coupled plasmon band is dependent on the temperature and the concentration of the cationic metal complexes, and it was observed in three steps: induction time, flocculation, and agglomeration. The process of interaction between cations of coordination metal complexes and the negative surface of gold nanoparticles (AuNPs^*z*–^) can be defined as a reversible chemical reaction with a spontaneous agglomeration between the species. The Gibbs free energy were all negative, and the Benesi–Hildebrand and Scatchard models provided very close results regarding the formation constant (*K*_f_) and Hill coefficient (*n*). Therefore, the value of *n* was close to 1.0 for almost all complexes, suggesting an independent process for agglomeration with a positive propensity to bind onto the AuNPs*^z–^* surface. Complex **5** presented a value of *n* close to 2, indicating the influence of two different binding sites for its agglomeration. The relative error in site binding does not show any variation with changes in the temperature, but a fine-tune of the *n* value to 1.00 was observed with the increase of the temperature, which was accompanied with the increase of *K*_f_, as described by the interaction between **9** and AuNPs*^z–^*. An effective inhibition of the catalytic performance of AuNPs^*z*–^ was followed in the presence of complexe **1,** before and after 45% surface coverage with **1**. But at θ = 0.45, the catalytic activity was practically the same for AuNPs*^z–^* alone. These results suggest that complex **1** is affecting the same binding site on the surface of gold nanoparticles used by 4-nitrophenolate (4-NP^–^), i.e., the negative surface of AuNPs*^z–^*. The manipulation of total binding sites occupied (θ) provides a way to modulate the cooperativity or noncooperativity behavior of supramolecular systems involving catalysis.

## Supporting Information Available

Graphs and data (PDF)
