# Supplementary material for: The Self-Assembly of Cationic Metal Complexes on Gold Nanoparticle Surface
Source: ACS Omega. 2024 Jun 14;9(26):28989–99. doi: 10.1021/acsomega.4c04098 (PMC11223192; doi:10.1021/acsomega.4c04098)
Supplement: Supplementary file 1 — ao4c04098_si_001.pdf [file ao4c04098_si_001.pdf]

Supporting Information Material

Self-Assembly of Cationic Metal Complexes on Gold Nanoparticles Surface

*Cássio Roberto Arantes do Prado[a,b], Matheus Henrique de Oliveira Pessoa[a], Lucas da Silva dos Santos[a,b], Aline da Silva Xavier da Cruz[a], Luís Rogério Dinelli[a], André Luiz Bogado[a]**

[a] Instituto de Ciências Exatas e Naturais do Pontal, Universidade Federal de Uberlândia, Rua vinte, 1600, CEP 38304-402, Ituiutaba - MG, Brazil

[b]Instituto de Química, Universidade Federal de Uberlândia, Av. João Naves de Avila 2121, 38400-902, Uberlândia, MG, Brazil

Table of Contents

Characteristics of the complexes "guest molecules" 2

Characterization of the Cationic Metal Complexes 5

UV/Vis spectroscopic data. 5

FTIR spectroscopic data. 10

Cyclic Voltammetry 14

Blank test in acetone 18

Description of θ 19

Conductivity as a function of concentration of M^{z+} 20

Kinetics for M^{z+} and $AuNPs^{z-}$ agglomerates 24

Langmuir isotherm for M^{z+} and $AuNPs^{z-}$ interactions 40

Benesi-Hildebrand isotherm for M^{z+} and $AuNPs^{z-}$ interactions 53

Scatchard isotherm for M^{z+} and $AuNPs^{z-}$ interactions 66

Approximate relative error for $M^{z+}$and $AuNPs^{z-}$ interactions 79

Kinetic of 4-nitrophenol reduction 92

Characteristics of the complexes “guest molecules”

1 [RuCl(dppb)(bipy)(py)]PF_6
Molar mass: 943.23 g mol^{-1}
Reaction scheme:

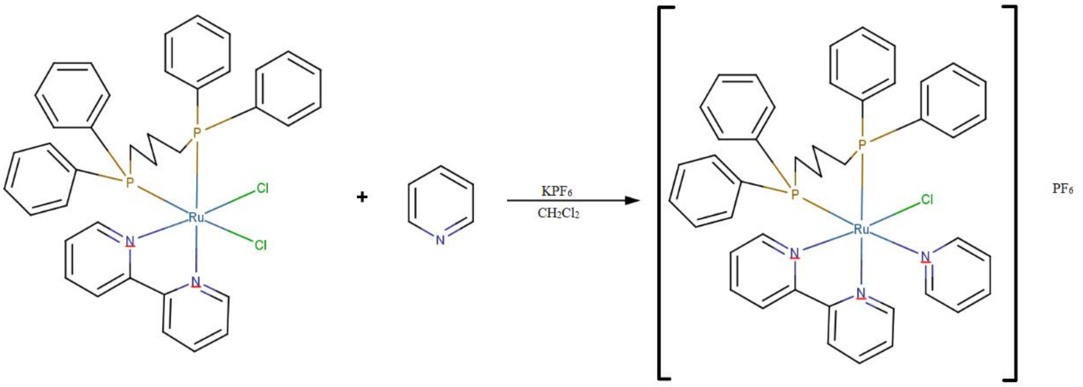

2 [RuCl(dppb)(bipy)(vpy)]PF_6
Molar mass: 969.27 g mol^{-1}
Reaction scheme:

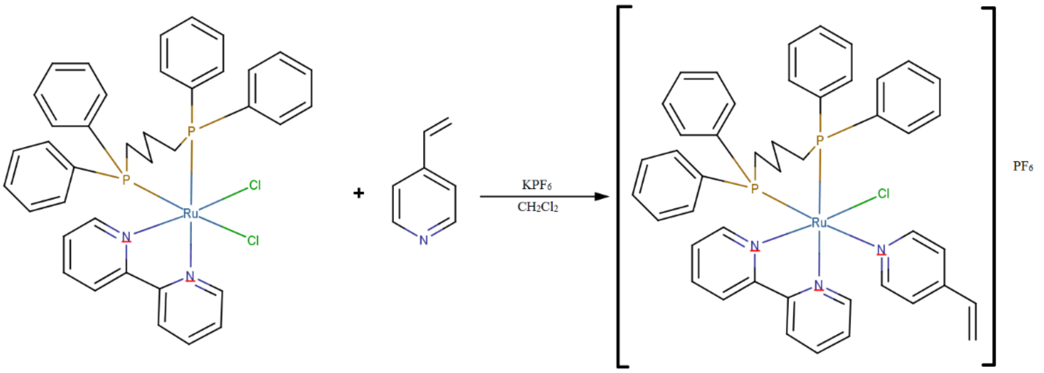

3 [RuCl(dppb)(bipy)(mepy)]PF_6
Molar mass: 957.26 g mol^{-1}
Reaction scheme:

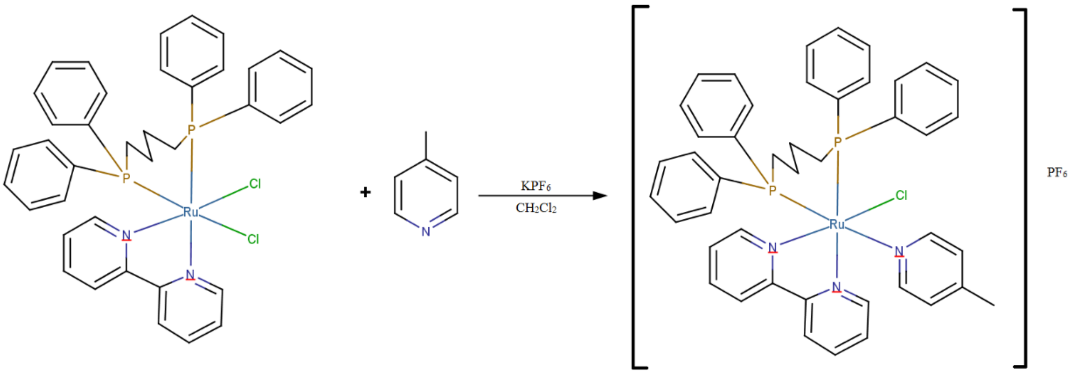

4 [RuCl(dppb)(bipy)(*t*bpy)]PF_6
Molar mass: 999.34 g mol^{-1}
Reaction scheme:

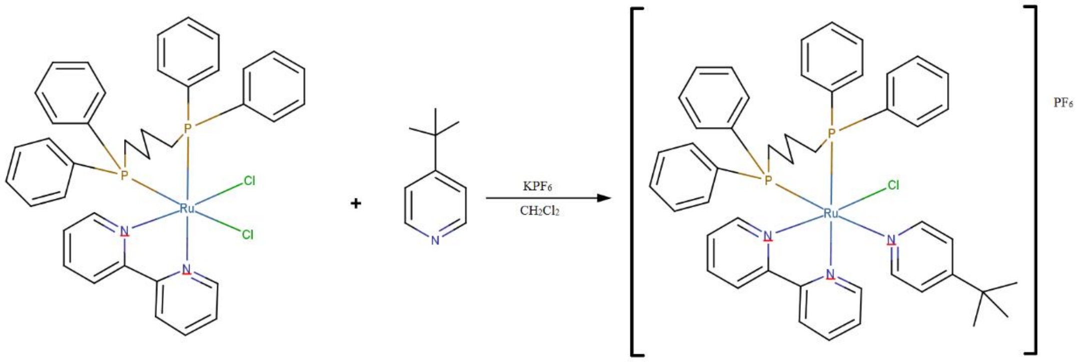

5 *cis*-[$RuCl_2$(dppb)(bipy)]PF_6
Molar mass: 899.59 g mol^{-1}
Reaction scheme:

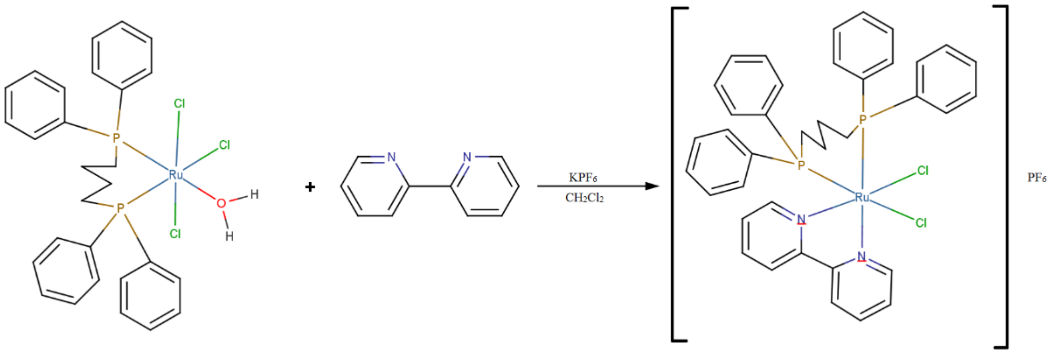

6 [Fe$(bipy)_3$]Cl_2
Molar mass: 595.29 g mol^{-1}
Reaction scheme:

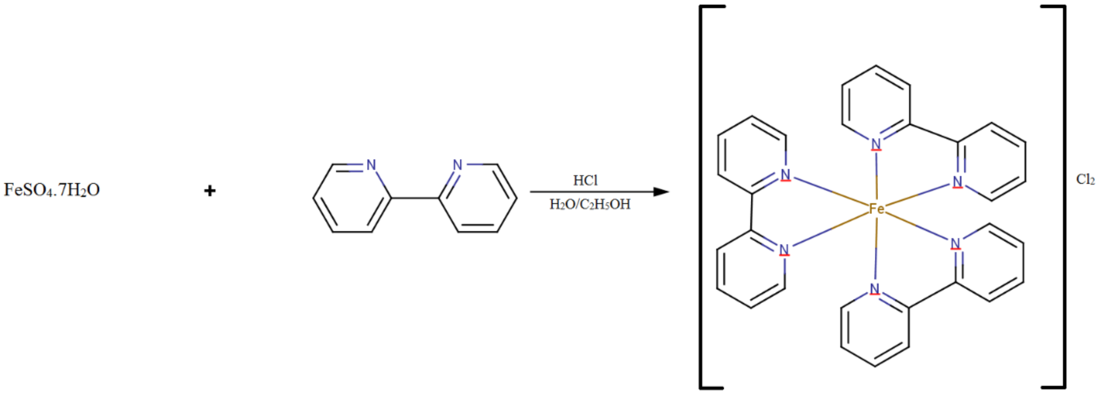

7 [Ru(bipy)3](PF6)2

Molar mass: 859.53 g mol^{-1}

Reaction scheme:

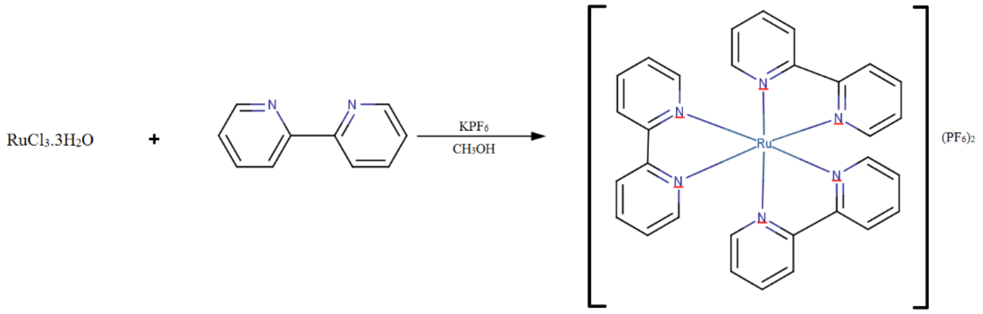

8 {TPyP[RuCl(dppb)(bipy)]4}(PF6)4

Molar mass: 4075.23 g mol^{-1}

Reaction scheme:

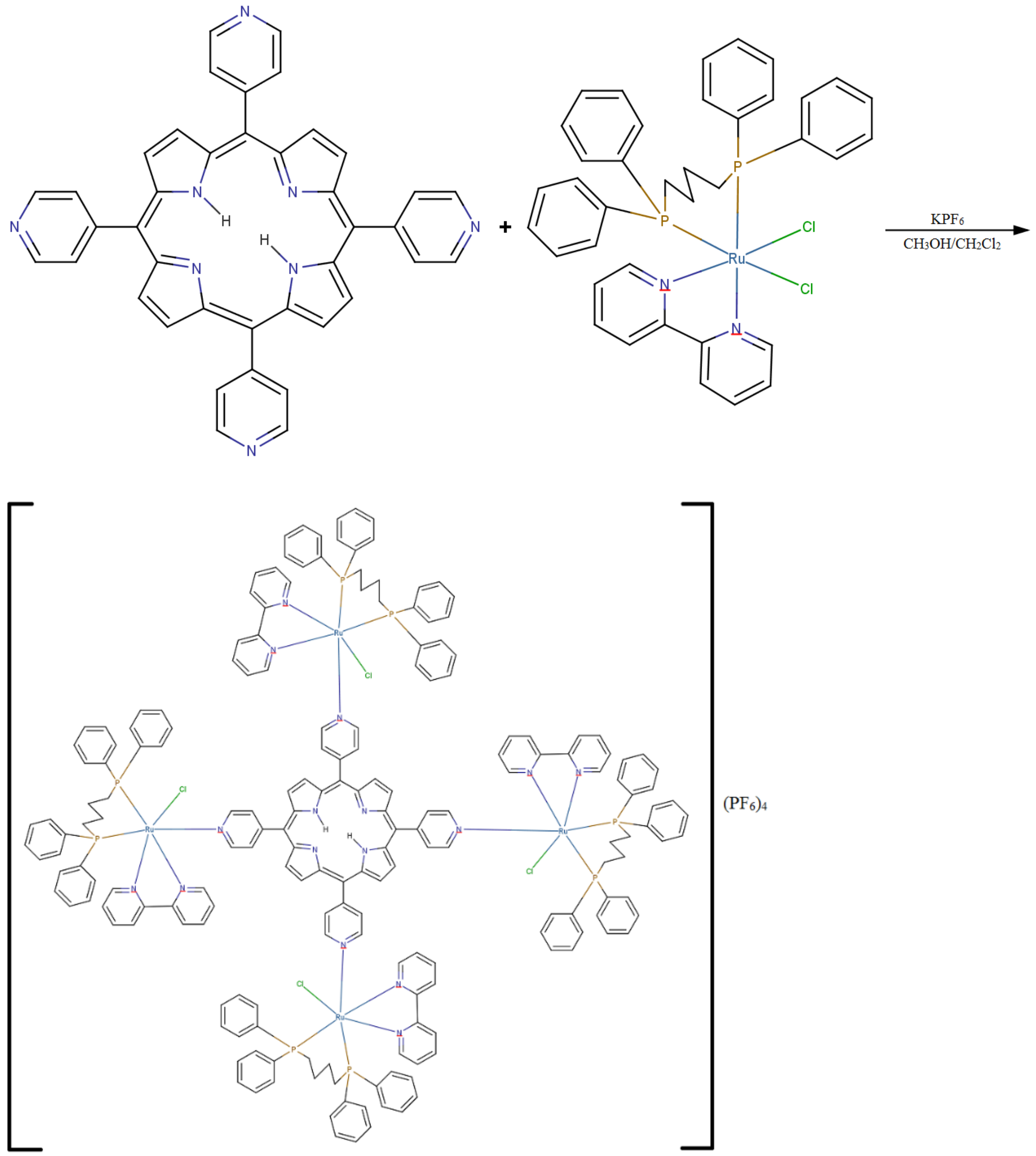

Characterization of the Cationic Metal Complexes

UV/Vis spectroscopic data.

Table S. 1: Uv/vis spectroscopy data for cationic metal complexes.

Complex	λ (nm)	Transfer	Ɛ (10^3 L mol^{-1} cm^{-1})
**1**	230, 294, 444	IL, IL, MLCT	41.9, 16.6, 2.36
**2**	247, 303, 338, 426	IL, IL, IL; MLCT	30.4, 10.2, 6.05, 1,12
**3**	231, 299, 417, 451	IL, IL, MLCT, MLCT	80.7, 25.9, 5.20, 4,30
**4**	229, 299, 449	IL, IL, TCML	28.5, 8.89, 1.45
**5**	244, 287, 356, 449	IL, IL, MLCT; MLCT	-
**6**	247, 298, 349, 552	IL; IL, MLCT, MLCT	23.6, 55.4, 5.20, 7,00
**7**	242, 287, 392, 451	IL, IL, MLCT, MLCT	84.0, 171, 17.6, 29,1
**8**	421, 515, 550, 590, 645	Soret, β e α	-
**9**	266, 368, 428	IL, MLCT, MLCT	12.0, 3.2, 3.8

IL = intraligand. MLCT = metal ligand charge transfer.ε = molar absorption coefficient.

Figure S. 1: UV/vis data of the [RuCl(dppb)(bipy)PF6 (**1**) in acetone solution.

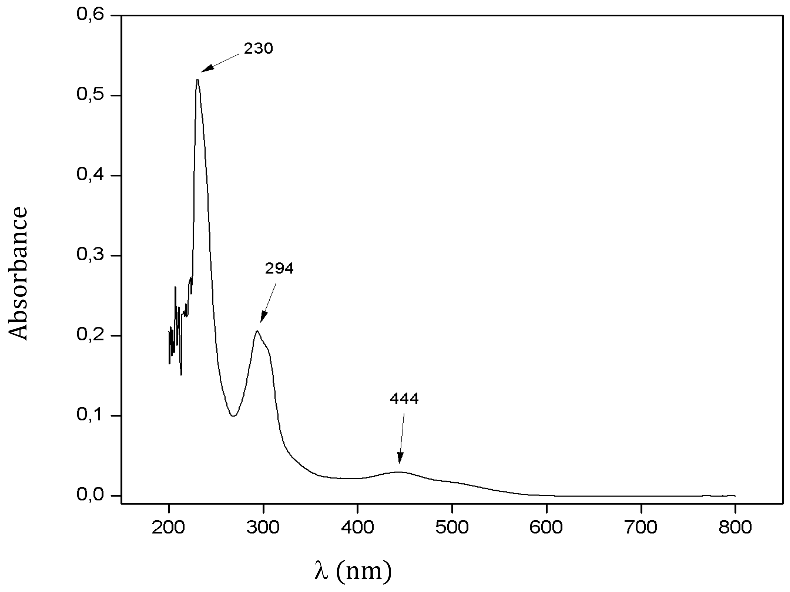

Figure S. 2: UV/vis data of the [RuCl(dppb)(vpy)PF6 (**2**) in acetone solution.

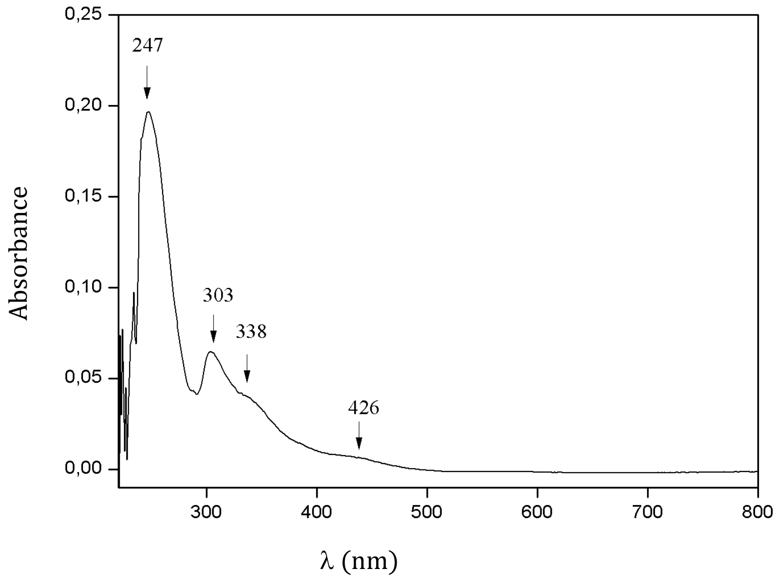

Figure S. 3: UV/vis data of the [RuCl(dppb)(vpy)PF6 (**3**) in acetone solution.

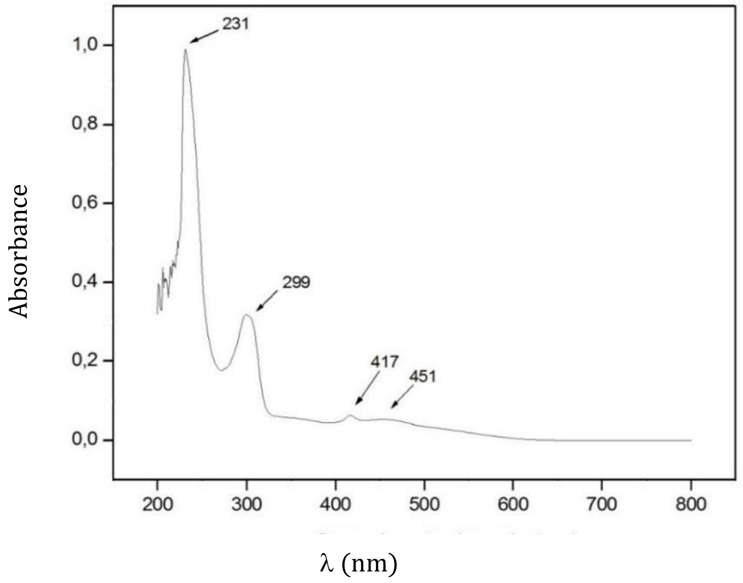

Figure S. 4: UV/vis data of the [RuCl(dppb)(tbpy)PF_6 (**4**) in acetone solution.

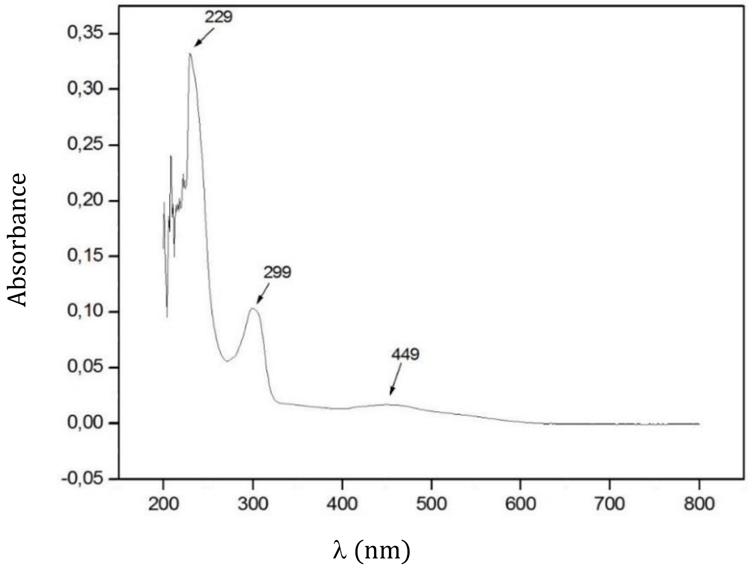

Figure S. 5: UV/vis data of the [$RuCl_2$(dppb)(bipy)PF_6 (**5**) in acetone solution.

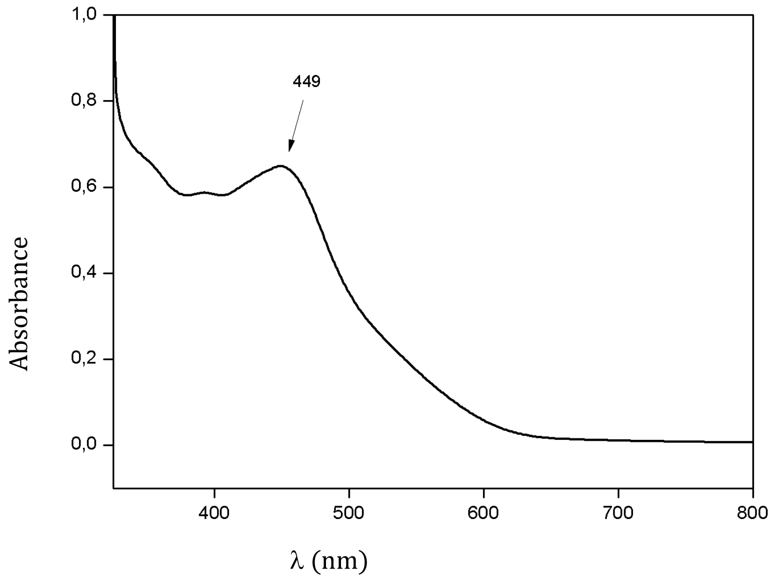

Figure S. 6: UV/vis data of the [Fe(bipy)$_3$]Cl$_2$ (**6**) in water solution.

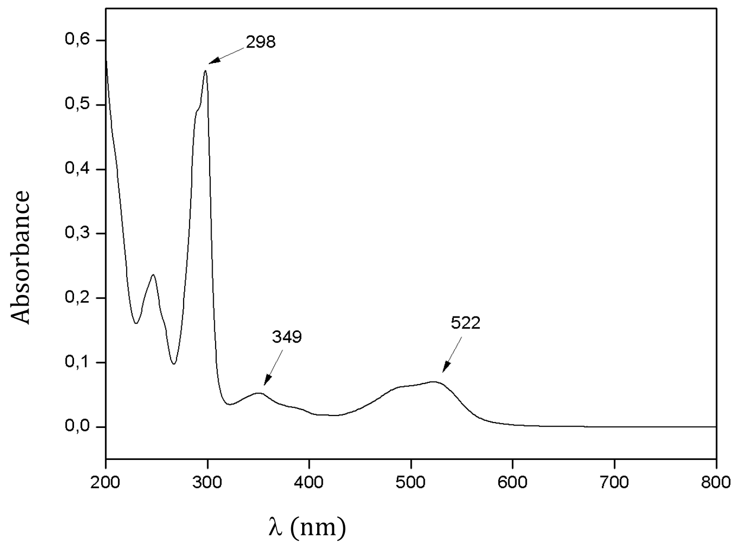

Figure S. 7: UV/vis data of the [Ru(bipy)$_3$]Cl$_2$ (**7**) in acetone solution.

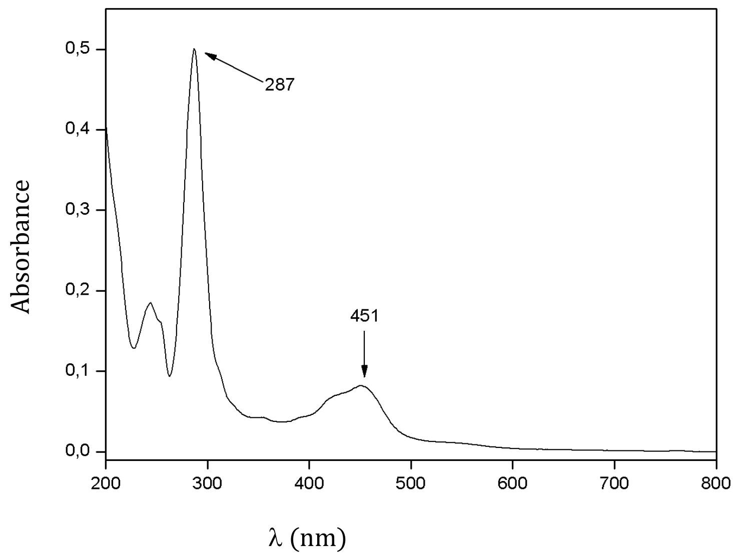

Figure S. 8: UV/vis data of the {TPyP[RuCl(dppb)(bipy)]$_4$}(PF_6)$_4$ (**8**) in acetone solution.

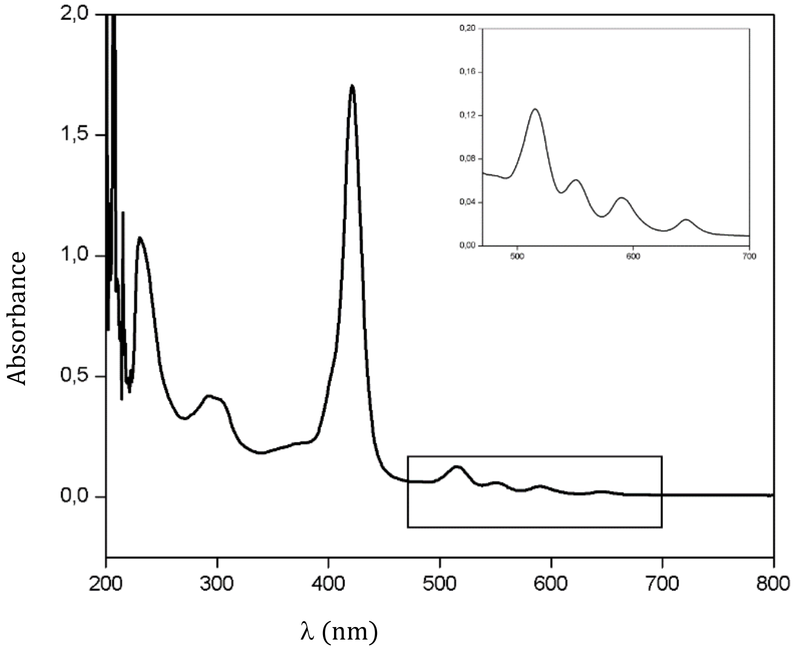

Figure S. 9: UV/vis data of the [RuCl(*p*-cymene) (Di*i*pmp)](PF_6) (**9**) in acetonitrile solution (black line). Uv/vis data of the [$RuCl_2$(*p*-cym)]$_2$ precursor in acetonitrile solution (red line).

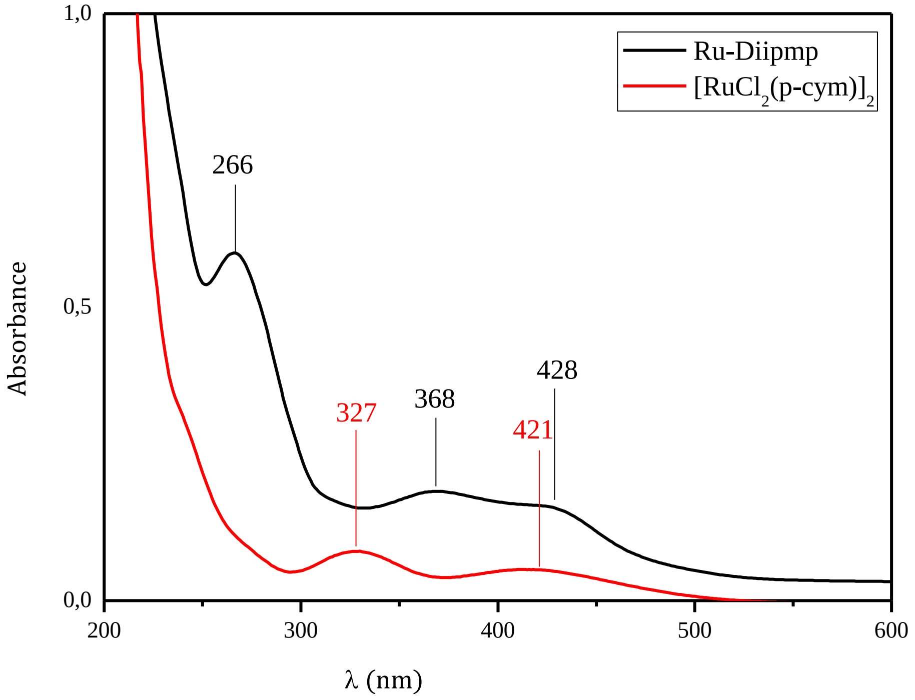

FTIR spectroscopic data.

Table S. 2: FTIR spectroscopic data for cationic metal complexes

Complex	IV KBr (cm^{-1})	Vibrational modes
**1**	3055-2923; 1602; 1435; 1092; 842; 697-557	$\nu_{(C-H)}$; $\nu_{(C-C)}$; $\nu_{(C=C)}$; $\nu_{(C-P)}$; $\nu_{(P-F)}$; $\delta_{(C-H)}$
**2**	3056; 1611; 1435; 1097; 839; 557	$\nu_{(C-H)}$; $\nu_{(C-C)}$; $\nu_{(C=C)}$; $\nu_{(C-P)}$; $\nu_{(P-F)}$; $\delta_{(C-H)}$
**3**	3057-2925; 1620; 1435; 1092; 842; 697-557	$\nu_{(C-H)}$; $\nu_{(C-C)}$; $\nu_{(C=C)}$; $\nu_{(C-P)}$; $\nu_{(P-F)}$; $\delta_{(C-H)}$
**4**	3056-2962; 1615; 1435; 1092; 841; 698-558	$\nu_{(C-H)}$; $\nu_{(C-C)}$; $\nu_{(C=C)}$; $\nu_{(C-P)}$; $\nu_{(P-F)}$; $\delta_{(C-H)}$
**5**	3056-2925; 1603; 1431; 1092; 841; 521	$\nu_{(C-H)}$; $\nu_{(C-C)}$; $\nu_{(C=C)}$; $\nu_{(C-P)}$; $\nu_{(P-F)}$; $\delta_{(C-H)}$
**6**	3073; 1602; 1406	$\nu_{(C-H)}$; $\nu_{(C-C)}$; $\nu_{(C=C)}$
**7**	3085; 1606; 1447; 838; 558	$\nu_{(C-H)}$; $\nu_{(C-C)}$; $\nu_{(C=C)}$; $\nu_{(P-F)}$; $\delta_{(C-H)}$
**9**	3070-2970, 1633, 1568	$\nu_{(C-H)}$, $\nu_{(C-N)pyridine}$, $\nu_{(C-H)imine}$

Figure S. 10: FTIR data of the [RuCl(dppb)(bipy)(py)]PF_6 (**1**).

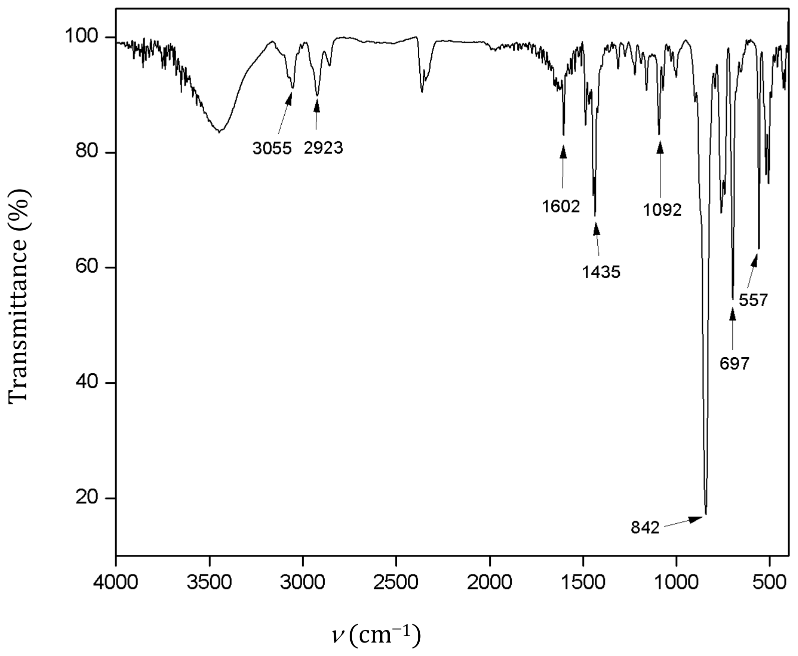

Figure S. 11: FTIR data of the [RuCl(dppb)(bipy)(vpy)]PF_6 (2).

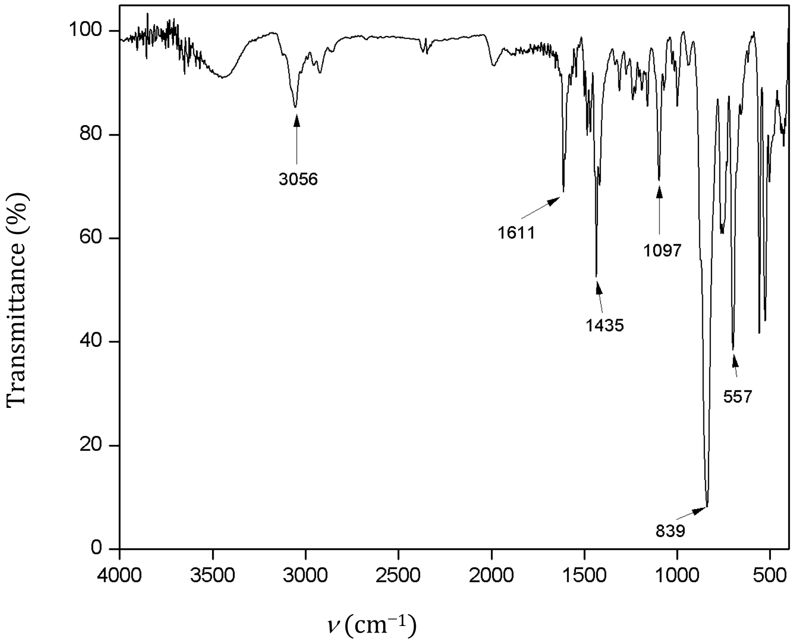

Figure S. 12: FTIR data of the [RuCl(dppb)(bipy)(mepy)]PF_6 (3).

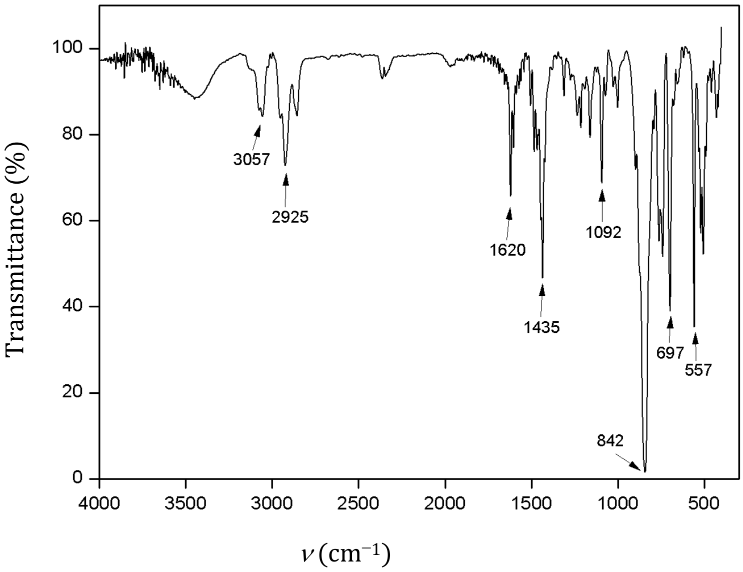

Figure S. 13: FTIR data of the [RuCl(dppb)(bipy)(*t*bpy)]PF_6 (**4**).

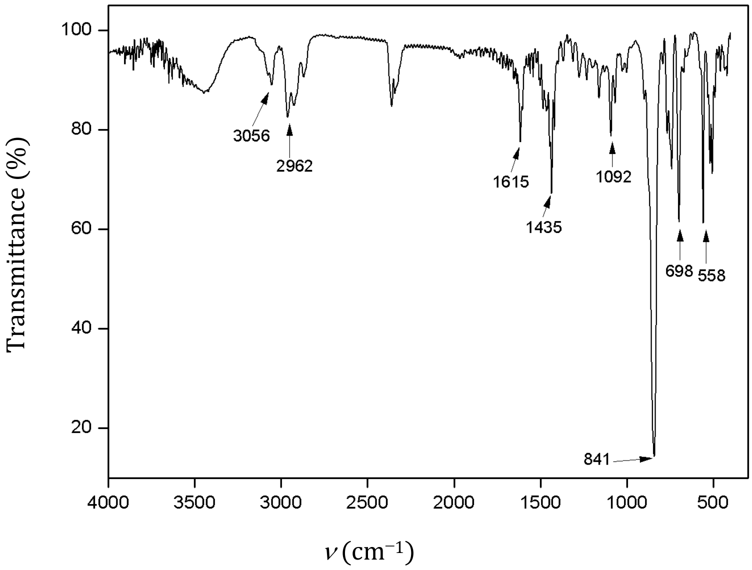

Figure S.: 14:FTIR data of the [$RuCl_2$(dppb)(bipy)]PF_6 (**5**)

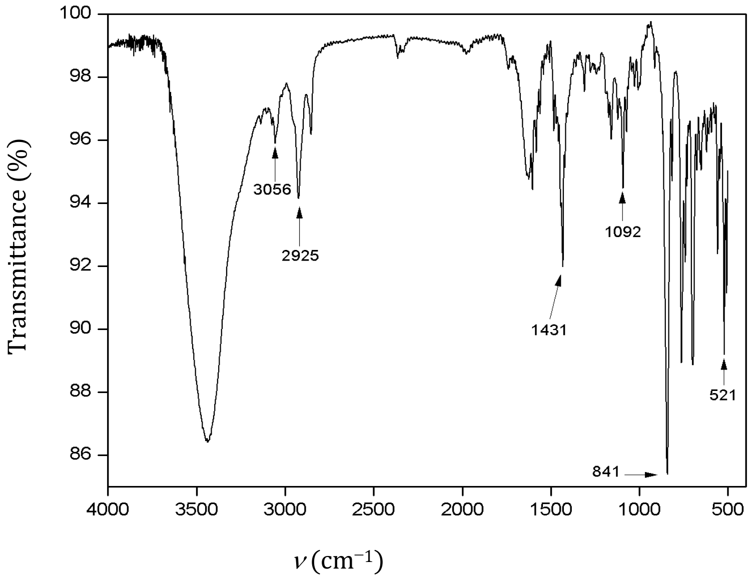

Figure S. 15: FTIR data of the [Fe(bipy)$_3$]Cl$_2$ (**6**).

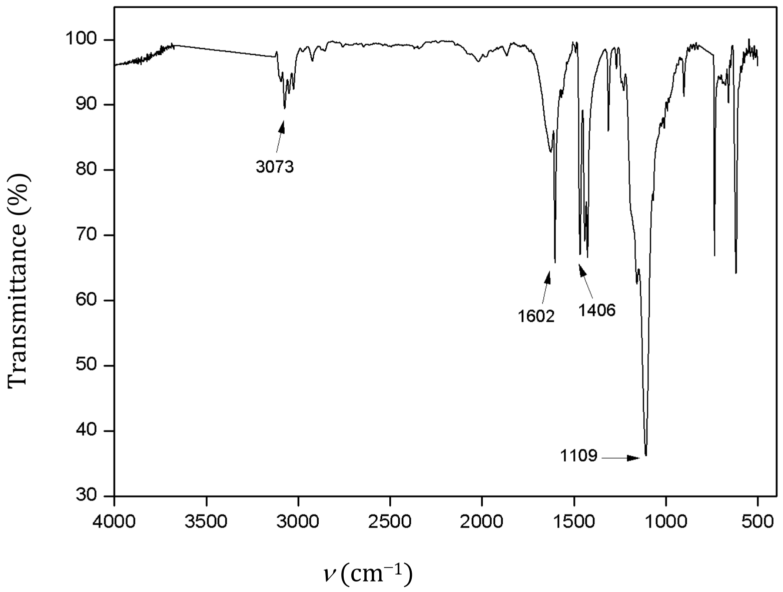

Figure S. 16: FTIR data of the [Ru(bipy)$_3$]Cl$_2$ (**7**)

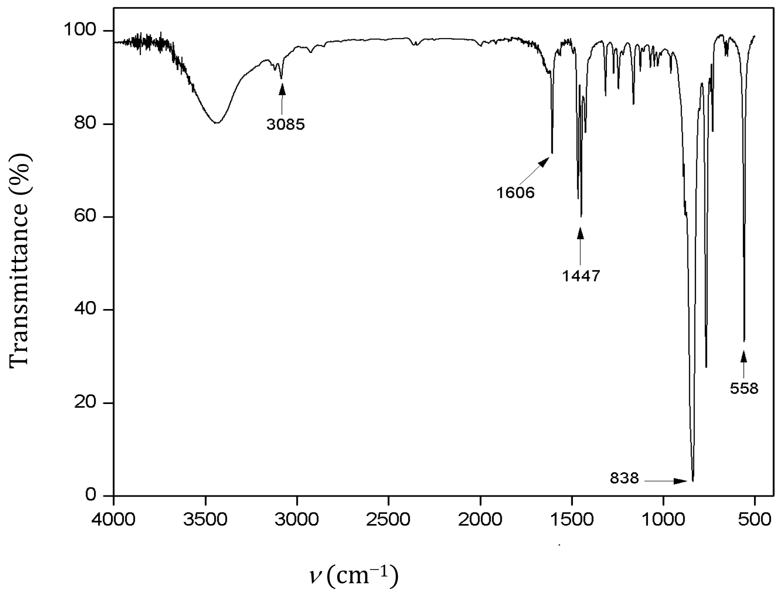

Figure S. 17FTIR data of the [RuCl(*p*-cymene) (Di*i*pmp)](PF_6) (**9**) (red line), and the free ligand Di*i*pmp (black line).

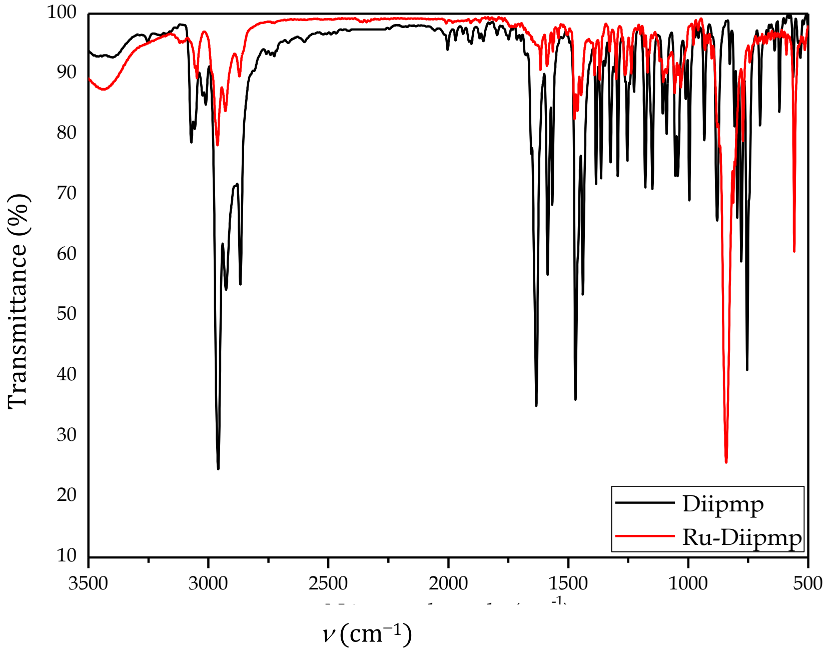

Cyclic Voltammetry

Table S. 3: Oxidation potential (E_{ox}) and reduction potential (E_{red}) for cationic metal complexes

**Complex**	E_{ox} (V)	E_{red} (V)
**1**	1.20	1.08
**2**	1.20	1.060
**3**	1.17	1.08
**4**	1.18	1.08
**7**	0.98	0.87
**8**	0.74, 1.23	0.51, 1.15
**9**	1.20	1.16

Figure S. 18: Cyclic voltammogram of the $[RuCl(dppb)(bipy)(py)]PF_6$ (**1**) in CH_2Cl_2 solution of HTBA 0.1 mol L^{-1}. Electrodes: W = Pt, CW = Pt, and reference = Ag/AgCl. Scan rate = 100 mV s^{-1}. Scan direction →.

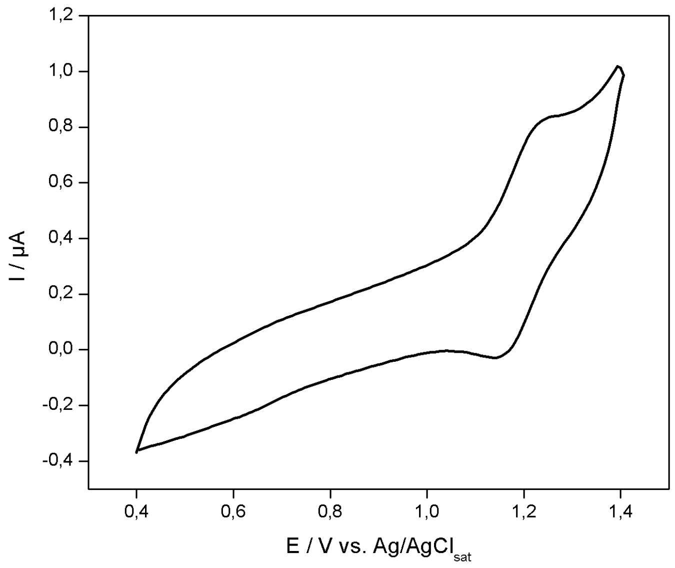

Figure S. 19: Cyclic voltammogram of the $[RuCl(dppb)(bipy)(vpy)]PF_6$ (**2**) in CH_2Cl_2 solution of HTBA 0.1 mol L^{-1}. Electrodes: W = Pt, CW = Pt, and reference = Ag/AgCl. Scan rate = 100 mV s^{-1}. Scan direction →.

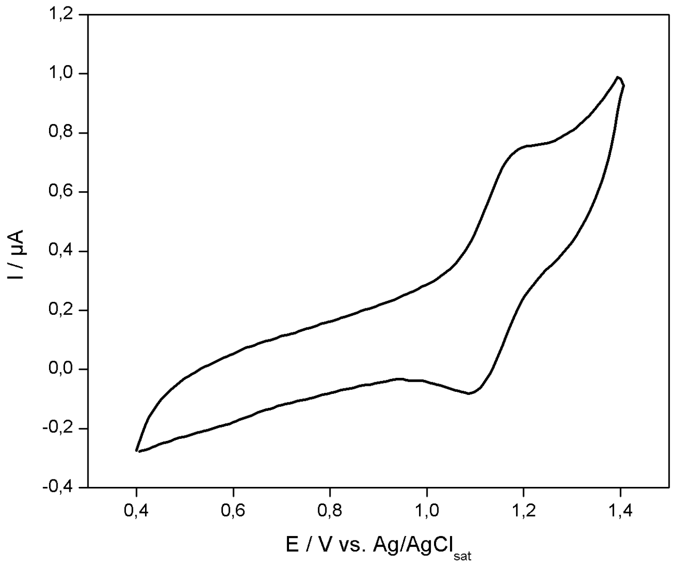

Figure S. 20: Cyclic voltammogram of the [RuCl(dppb)(bipy)(mepy)]PF_6 (**3**) in CH_2Cl_2 solution of HTBA 0.1 mol L^{-1}. Electrodes: W = Pt, CW = Pt, and reference = Ag/AgCl. Scan rate = 100 mV s^{-1}. Scan direction →.

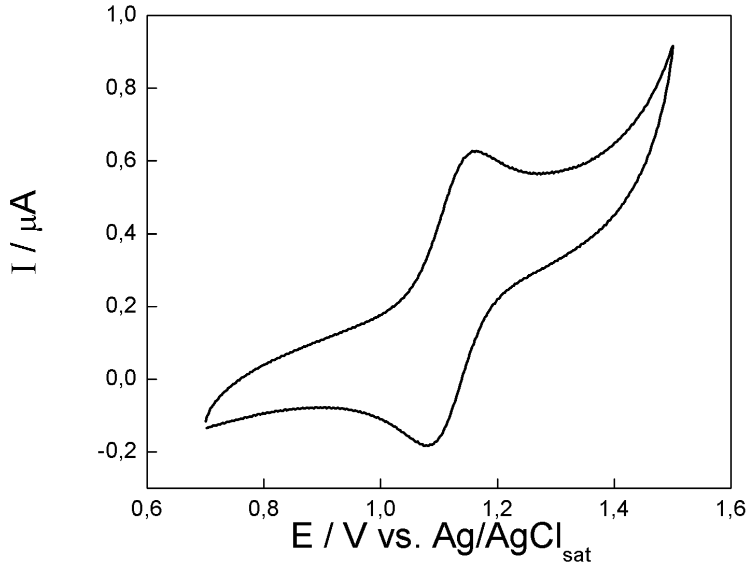

Figure S. 21: Cyclic voltammogram of the [RuCl(dppb)(bipy)(mepy)]PF_6 (**4**) in CH_2Cl_2 solution of HTBA 0.1 mol L^{-1}. Electrodes: W = Pt, CW = Pt, and reference = Ag/AgCl. Scan rate = 100 mV s^{-1}. Scan direction →.

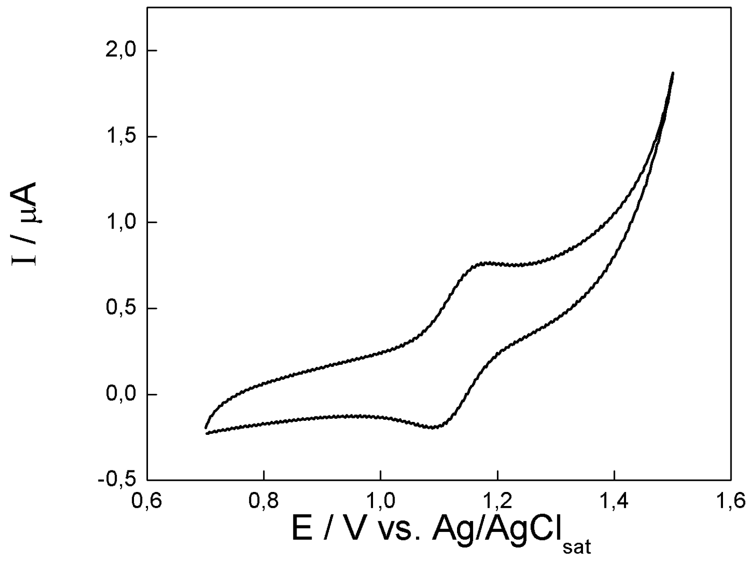

Figure S. 22: Cyclic voltammogram of the $[Fe(bipy)_3]Cl_2$ (**6**) in water solution of KCl 0.1 mol L^{-1}. Electrodes: W = Pt, CW = Pt, and reference = Ag/AgCl. Scan rate = 100 mV s^{-1}. Scan direction →.

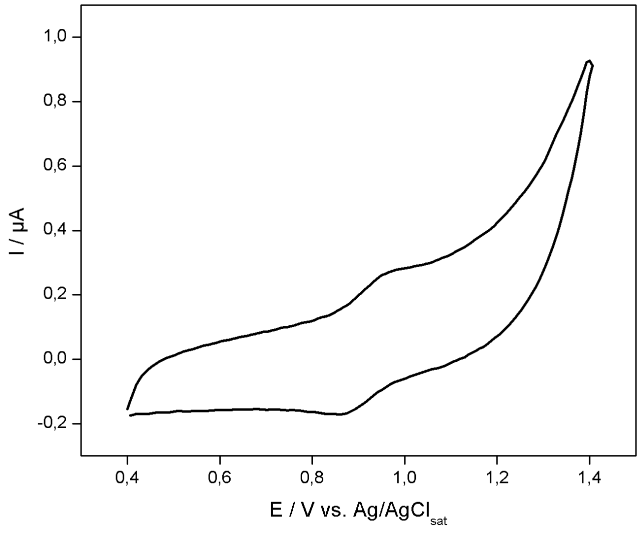

Figure S. 23: Cyclic voltammogram of the $\{TPyP[RuCl(dppb)(bipy)]_4\}(PF_6)_4$ (**8**) in CH_2Cl_2 solution of HTBA 0.1 mol L^{-1}. Electrodes: W = Pt, CW = Pt, and reference = Ag/AgCl. Scan rate = 100 mV s^{-1}. Scan direction →.

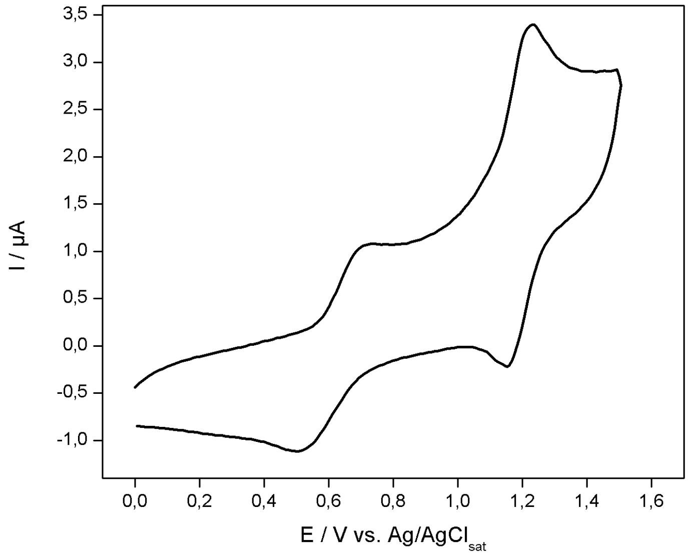

Figure S. 24: Cyclic voltammogram of the [RuCl(*p*-cymene)(Di*i*pmp)]PF_6 (**9**) in acetonitrile solution of HTBA 0.1 mol L^{-1}. Electrodes: W = glass carbon, CW = Pt, and reference = Ag/AgCl. Scan rate = 100 mV s^{-1}. Scan direction →.

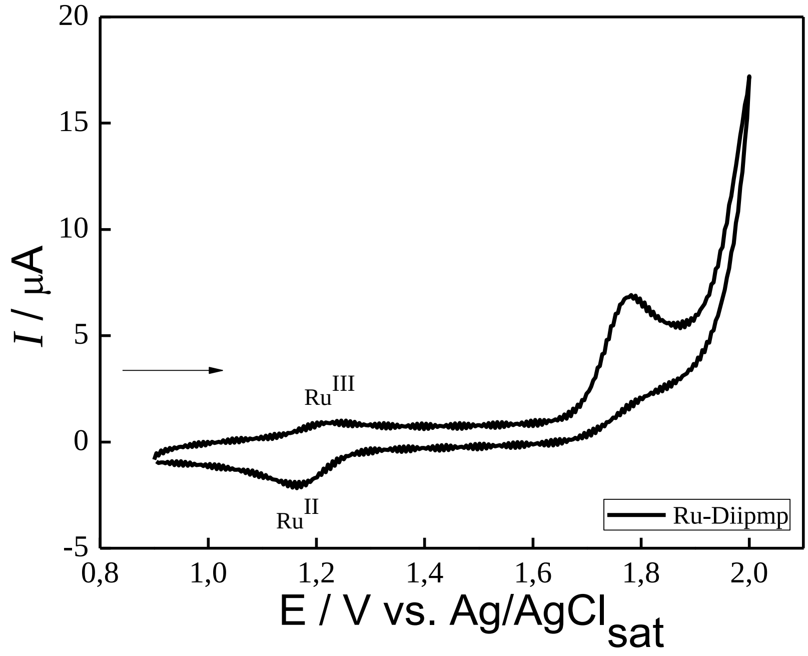

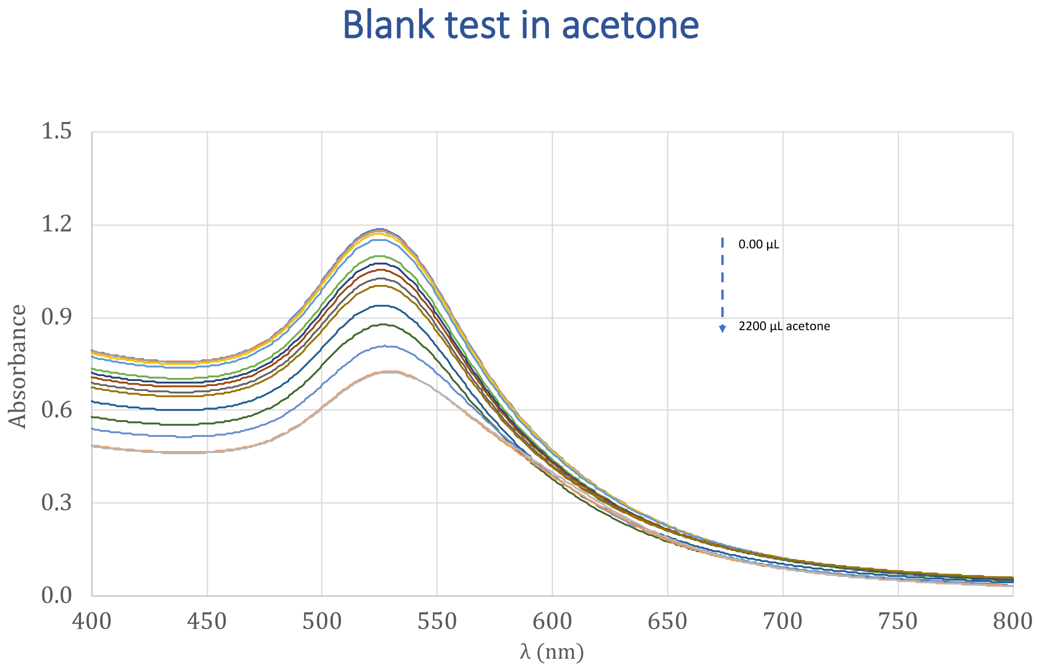

Figure S. 25: Addition of acetone into colloidal suspension of $AuNPs^{z-}$.

Description of θ

The fraction of total binding sites occupied (θ) is described in the equation (2):

$$\theta = \frac{[AuM]}{[Au]_{tot}} = \frac{[Au]_{tot}-[Au]_x}{[Au]_{tot}} = \frac{[M]}{(Kd+[M])} \text{ (eqn. 2)}$$

where, $[Au]_{tot}$ represents the initial concentration of $AuNPs^{z-}$, $[Au]_x$ the concentration of $AuNPs^{z-}$ on the time and K_d is the dissociation constant. Since $[AuM]$ represents the $Ru^+/AuNPs^{z-}$ agglomerates, an alternative demonstration of θ can be described from eqn. 3 as follows:

$$\theta = \frac{[Au^- M^+]}{[Au^-]_{tot}}$$

Where:

$$[Au]_{tot} = [Au]_x + [AuM]$$

$$[AuM] = [Au]_{tot} - [Au]_x$$

$$\theta = \frac{[Au]_{tot} - [Au]_x}{[Au]_x + [Au]_{tot} - [Au]_x}$$

$$\theta = \frac{[Au]_{tot} - [Au]_x}{[Au]_{tot}}$$

$$\theta = 1 - \frac{[Au]_x}{[Au]_{tot}} \text{ (eqn. 3)}$$

This non-linear curve is known as the Langmuir isotherm or the direct plot as depicted in the literature [5, 30] (Fig 5A), which efforts to obtain the dissociation constant (K_d) is such a tough. Further manipulation of the Langmuir isotherm yields two linear forms that are more accessible to obtain the K_d and the Hill constant (n), the double-reciprocal plot called Benesi-Hildebrand binding curve and the x-reciprocal called Scatchard plot (Fig 5B and C). summarizes these three representation of ligand-biding isotherm for addition of $[RuCl(dppb)(bipy)(py)]^+$ in a colloidal solution of $AuNPs^{z-}$. These models provide the dissociation constant (K_d), formation constant ($K_f = 1/K_d$) and Hill coefficient (n) (Figure 5D). summarize the results obtained from Benesi-Hildebrand and Scatchard models for a series of ruthenium complexes applied to form aggregates with $AuNPs^{x-}$.

Conductivity as a function of concentration of M^{z+}

Figure S. 26: Graph of molar conductivity as a function of concentration for the interaction of the $[Fe(bipy)_3]Cl_2$ complex with $AuNPs^{z-}$.

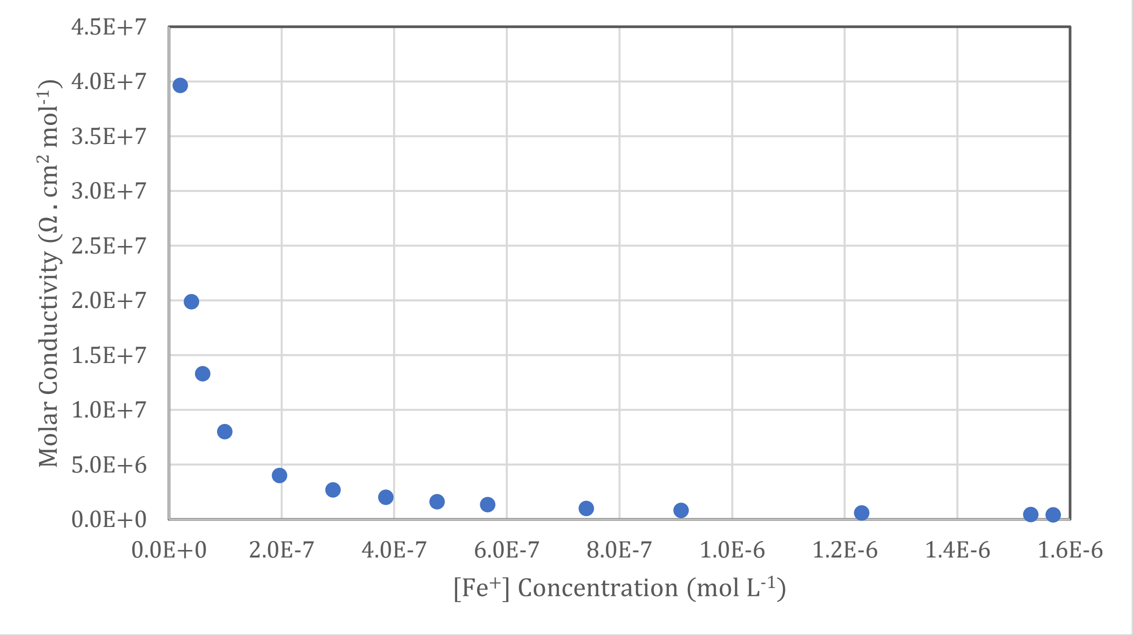

Figure S. 27: Graph of molar conductivity as a function of concentration for the interaction of the $[Ru(bipy)_3](PF_6)_2$ complex with $AuNPs^{z-}$.

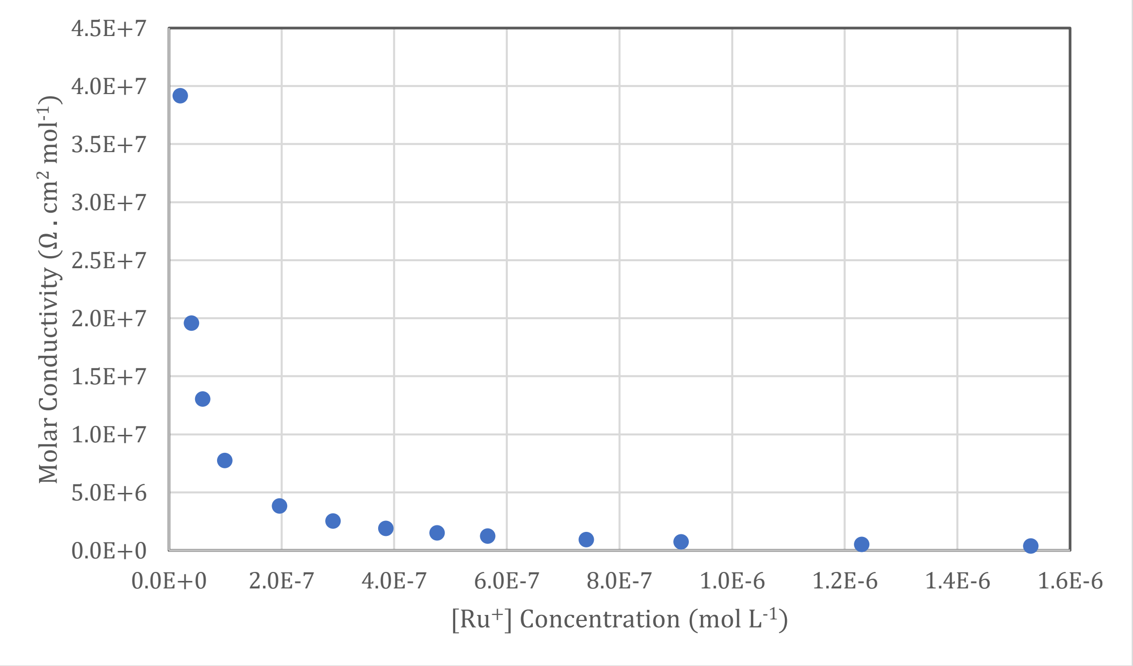

Figure S. 28: Graph of molar conductivity as a function of concentration for the interaction of the $[RuCl(dppb)(bipy)(py)]PF_6$ complex with $AuNPs^{Z-}$.

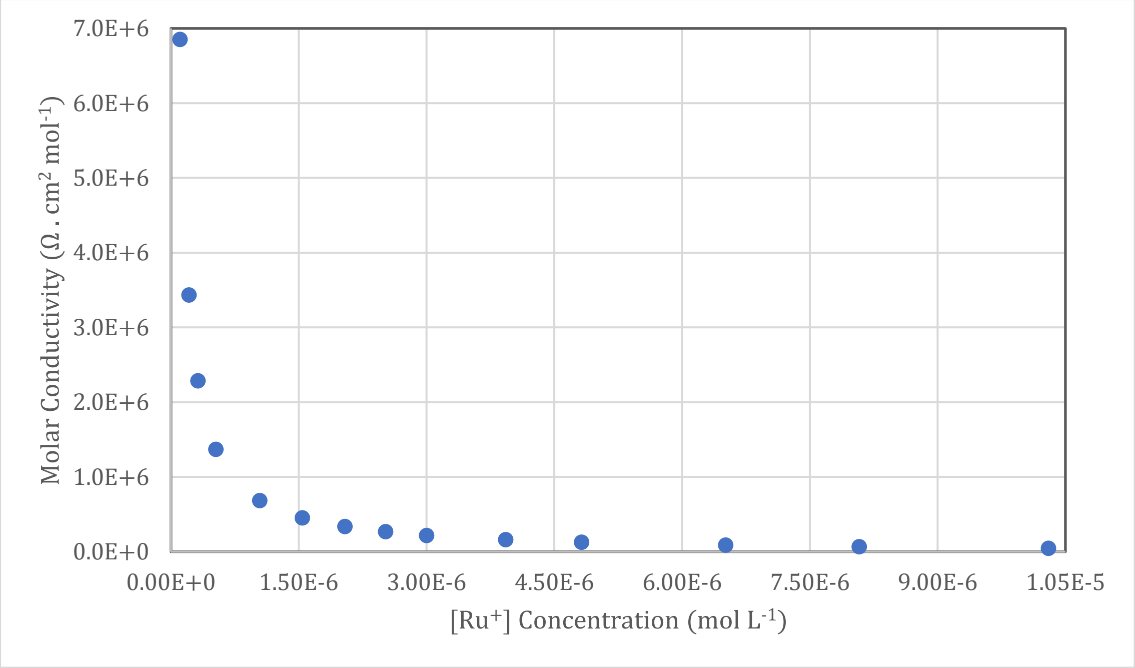

Figure S. 29: Graph of molar conductivity as a function of concentration for the interaction of the $[RuCl(dppb)(bipy)(t\text{bpy})]PF_6$ complex with $AuNPs^{Z-}$.

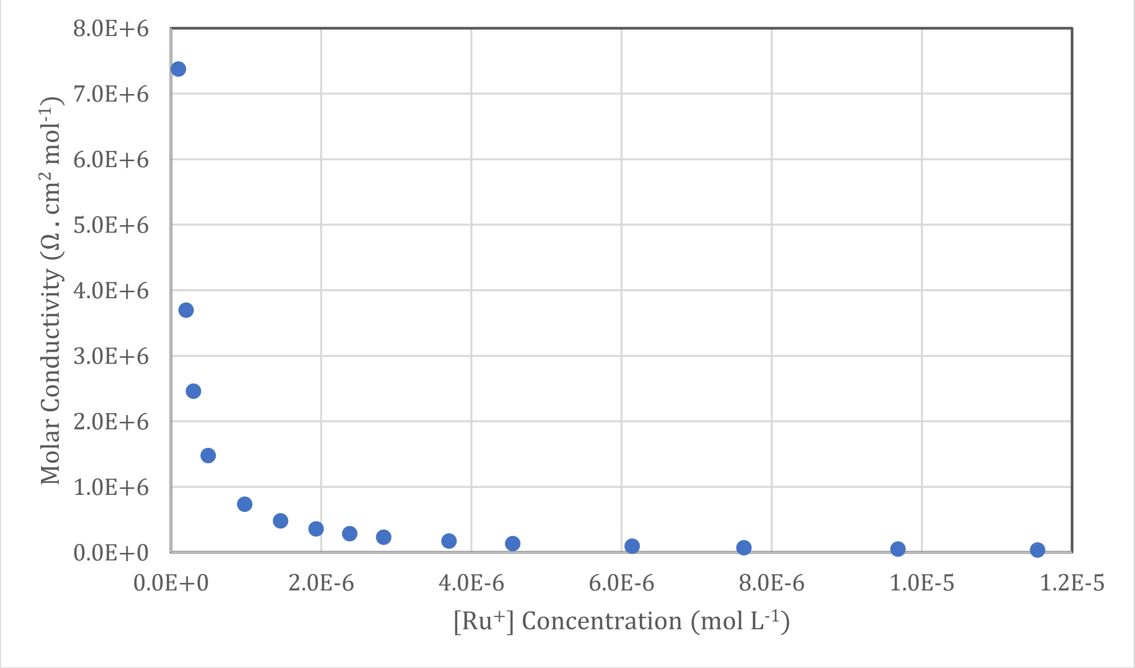

Figure S. 30: Graph of molar conductivity as a function of concentration for the interaction of the [RuCl(dppb)(bipy)(mepy)]PF_6 complex with AuNPs^{z-}.

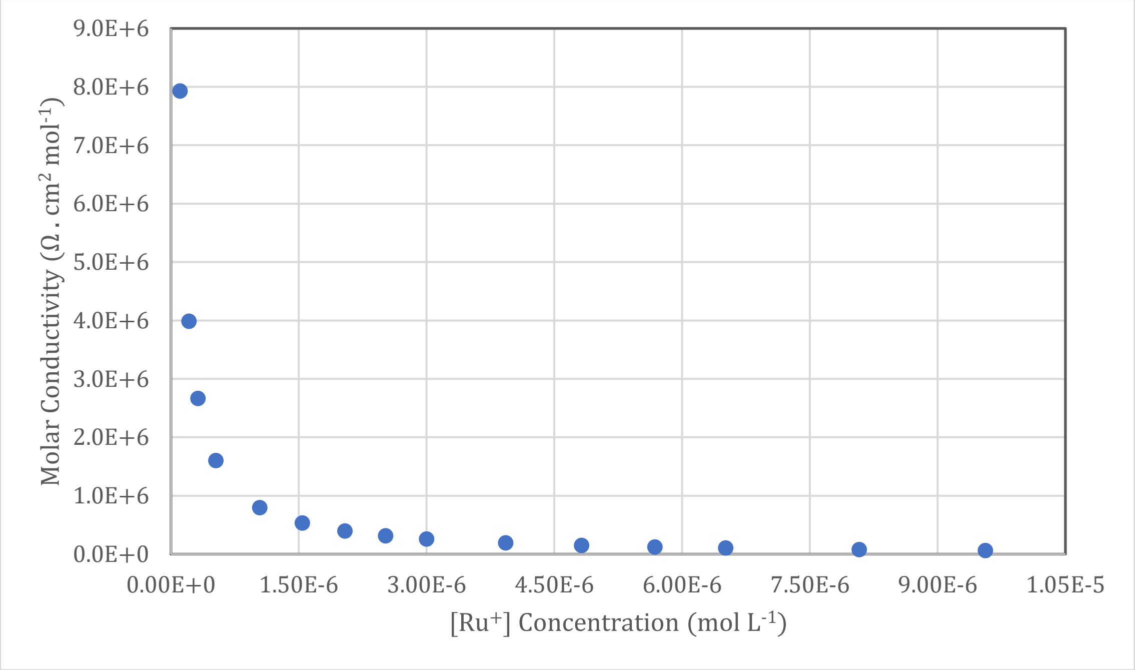

Figure S. 31: Graph of molar conductivity as a function of concentration for the interaction of the [RuCl(dppb)(bipy)(vpy)]PF_6 complex with AuNPs^{z-}.

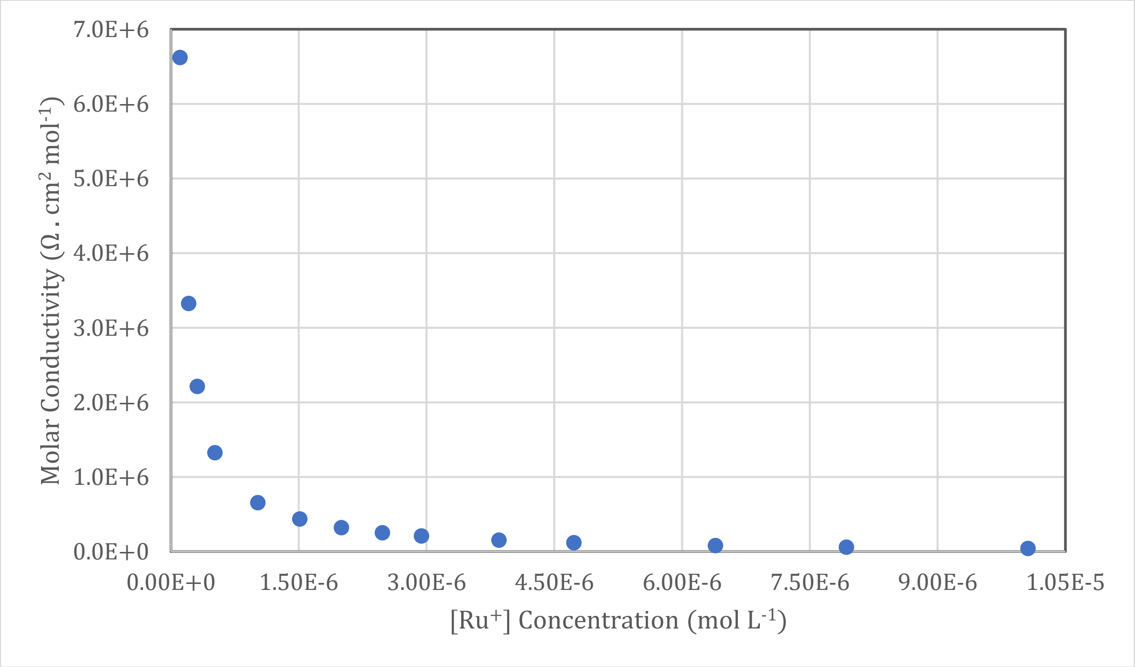

Figure S. 32: Graph of molar conductivity as a function of concentration for the interaction of the *cis*-$[RuCl_2(dppb)(bipy)]PF_6$ complex with $AuNPs^{z-}$.

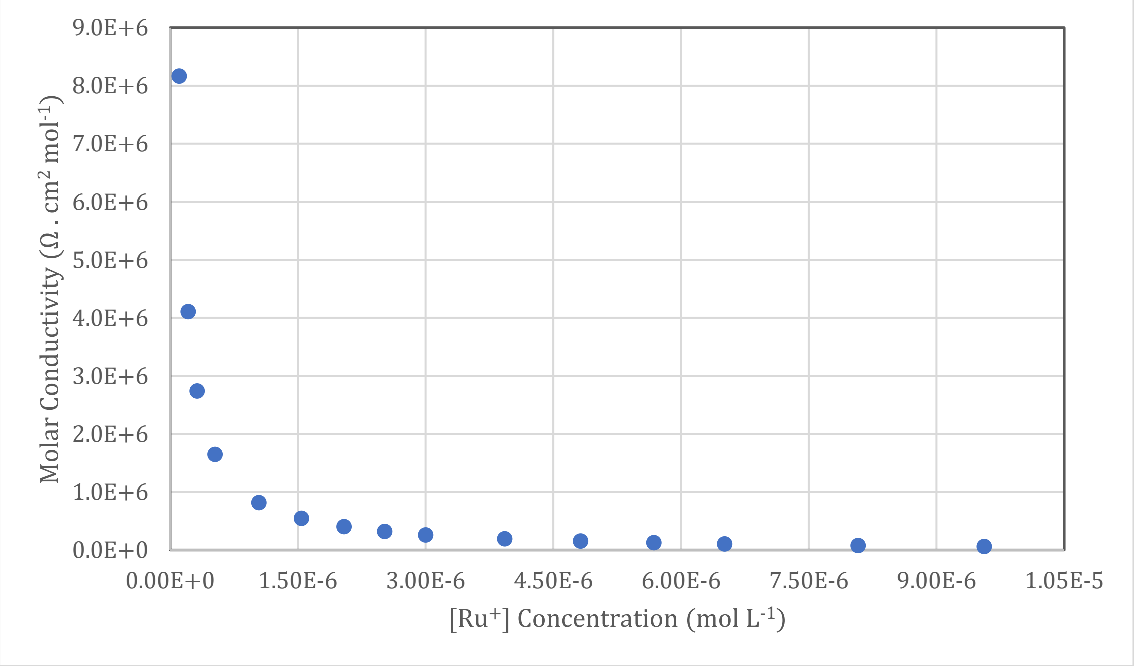

Figure S. 33: Graph of molar conductivity as a function of concentration for the interaction of the $\{TPyP[RuCl(dppb)(bipy)]_4\}(PF_6)_4$ complex with $AuNPs^{z-}$.

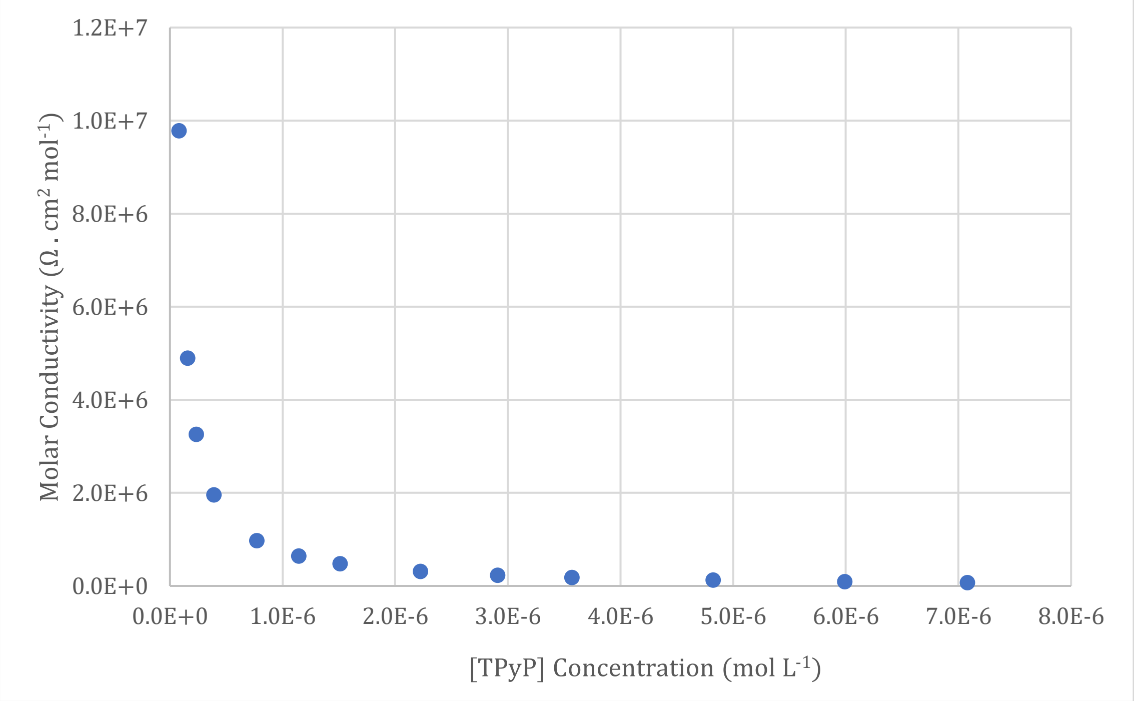

Kinetics for M^{z+} and $AuNPs^{z-}$ agglomerates

Effect of temperature variation

Table S. 4: *k* rate constants calculated at **520 nm** wavelength with constant aliquot addition. *k* unit: s^{-1}.

**T (°C)**	**1** (10^{-4})	**2** (10^{-4})	**3** (10^{-4})	**4** (10^{-4})	**5** (10^{-4})	**6** (10^{-4})	**7** (10^{-4})
**20.0**	3.15	0.95	1.08	0.67	2.75	0.16	4.56
**22.5**	3.73	1.09	1.21	1.06	2.78	0.33	4.78
**25.0**	3.96	1.36	1.37	1.45	3.14	0.52	5.28
**27.5**	4.04	1.61	2.98	1.60	3.82	0.69	6.04
**30.0**	4.88	2.35	3.68	1.68	4.91	1.07	6.67
**32.5**	5.98	3.21	4.25	1.83	6.58	2.11	7.49
**35.0**	6.47	3.52	5.87	2.07	7.06	3.19	9.30

Table S. 5: *k* rate constants calculated at 625 nm wavelength with constant aliquot addition for the **flocculation** period. *k* unit: s^{-1}.

**T (°C)**	**1** (10^{-3})	**2** (10^{-3})	**3** (10^{-3})	**4** (10^{-3})	**5** (10^{-3})	**6** (10^{-3})	**7** (10^{-3})
**20.0**	1.67	0.54	5.36	1.09	2.86	0.42	3.65
**22.5**	2.00	1.25	1.25	1.24	4.32	0.60	3.83
**25.0**	2.54	1.37	1.37	1.98	4.56	0.89	4.91
**27.5**	2.90	1.68	1.68	2.03	5.57	1.44	5.31
**30.0**	3.39	1.80	1.80	2.15	5.84	4.69	5.63
**32.5**	3.89	2.71	2.71	2.70	6.05	5.02	5.67
**35.0**	4.20	3.45	3.45	3.87	8.22	6.08	5.69

Table S. 6: *k* rate constants calculated at 625 nm wavelength with constant aliquot addition for the **agglomeration** period. *k* unit: s^{-1}.

T (°C)	**1** (10^{-4})	**2** (10^{-5})	**3** (10^{-4})	**4** (10^{-5})	**5** (10^{-4})	**6** (10^{-4})	**7** (10^{-4})
**20.0**	0.64	4.36	0.64	-	0.91	-	-
**22.5**	0.78	5.36	0.65	-	0.93	-	-
**25.0**	0.83	6.69	0.79	-	1.07	-	-
**27.5**	0.87	6.78	1.13	6.69	1.22	-	-
**30.0**	1.02	7.54	1.44	8.29	1.37	-	-
**32.5**	1.23	8.13	1.52	8.36	1.40	1.98	-
**35.0**	1.43	8.79	1.81	9.96	1.56	2.51	-

Figure S. 34: Interaction kinetics between the [RuCl(dppb)(bipy)(py)]PF_6 complex and $AuNPs^{z-}$ performed by UV-Vis spectroscopy for the 520 nm band with temperature variation (°C). ([Ru^+] Concentration: 1 x 10^{-5} mol L^{-1}).

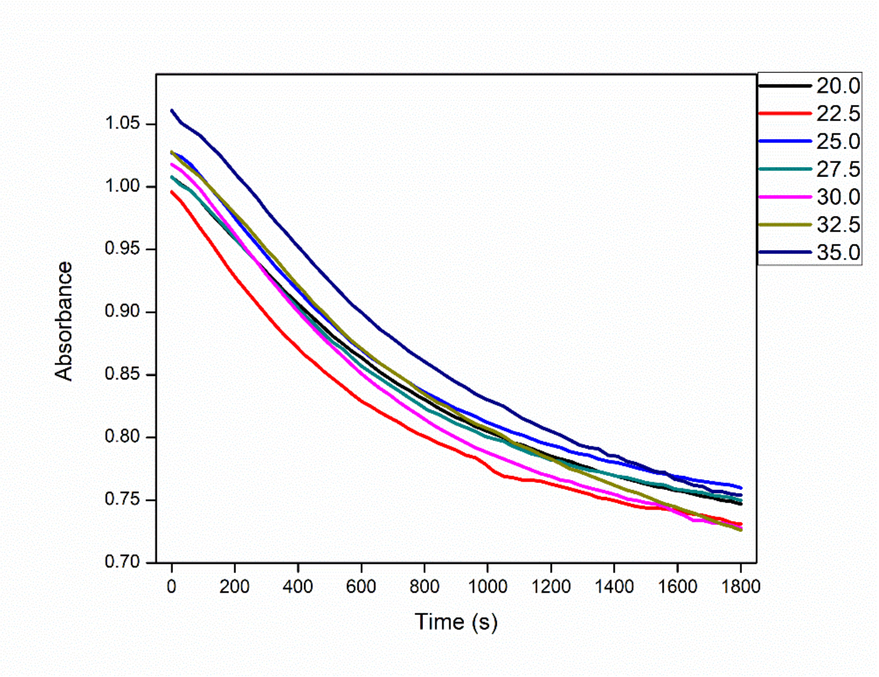

Figure S. 35: Interaction kinetics between the [RuCl(dppb)(bipy)(py)]PF_6 complex and $AuNPs^{z-}$ performed by UV-Vis spectroscopy for the 625 nm band with temperature variation (°C). ([Ru^+] Concentration: 1 x 10^{-5} mol L^{-1}).

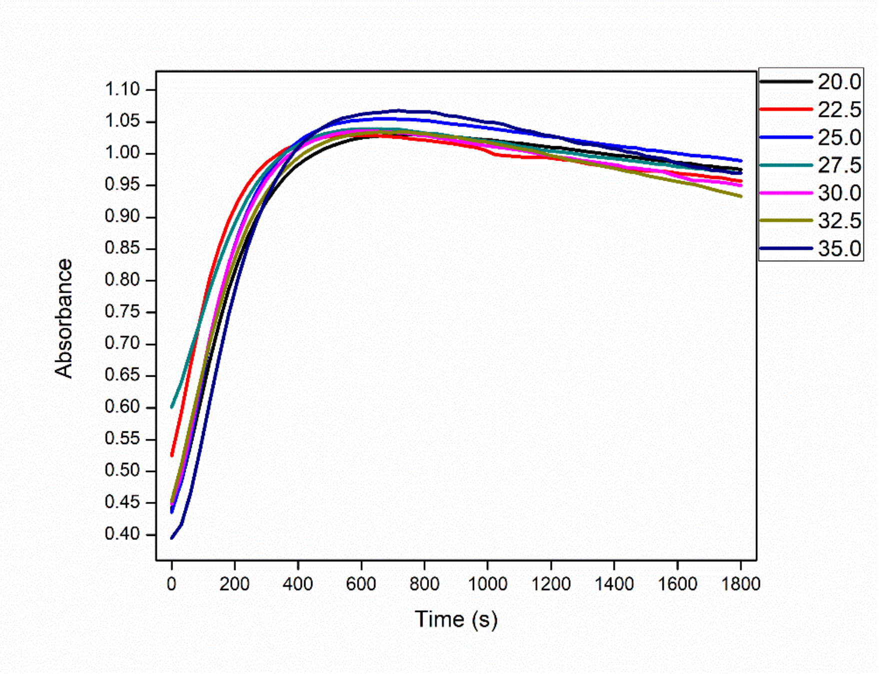

Figure S. 36: Interaction kinetics between the [RuCl(dppb)(bipy)(vpy)]PF_6 complex and $AuNPs^{z-}$ performed by UV-Vis spectroscopy for the 520 nm band with temperature variation (°C). ([Ru^+] Concentration: 1 x 10^{-5} mol L^{-1}).

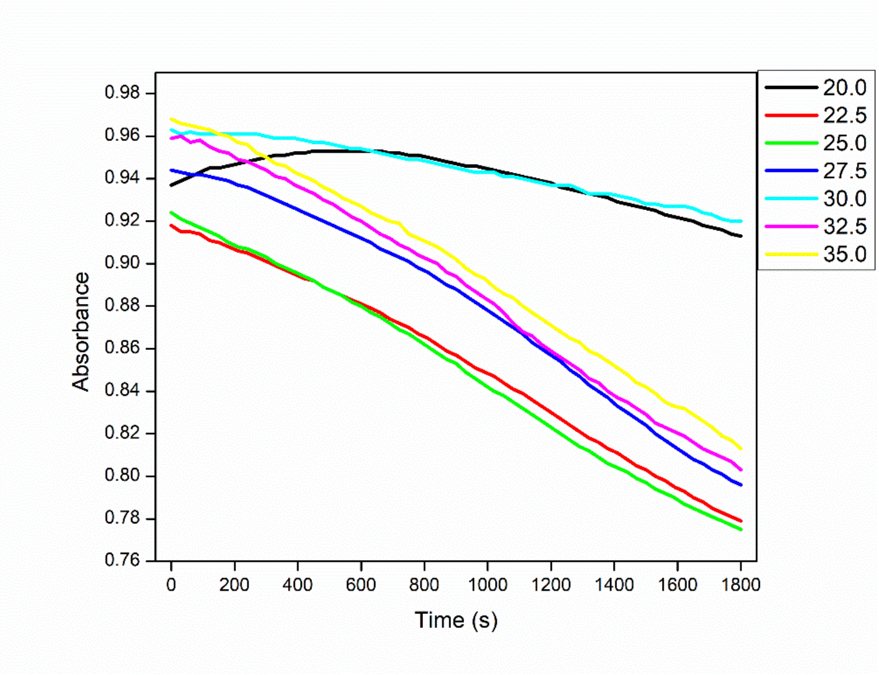

Figure S. 37: Interaction kinetics between the [RuCl(dppb)(bipy)(vpy)]PF_6 complex and $AuNPs^{z-}$ performed by UV-Vis spectroscopy for the 625 nm band with temperature variation (°C). ([Ru^+] Concentration: 1 x 10^{-5} mol L^{-1}).

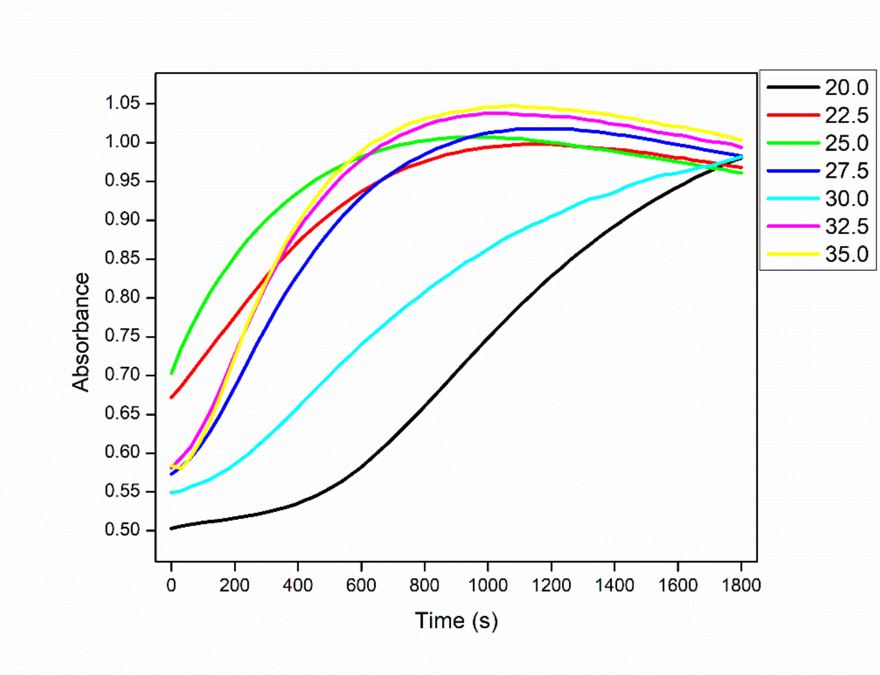

Figure S. 38: Interaction kinetics between the [RuCl(dppb)(bipy)(mepy)]PF_6 complex and AuNPs^{z-} performed by UV-Vis spectroscopy for the 520 nm band with temperature variation (°C). ([Ru^+] Concentration: 1 x 10^{-5} mol L^{-1}).

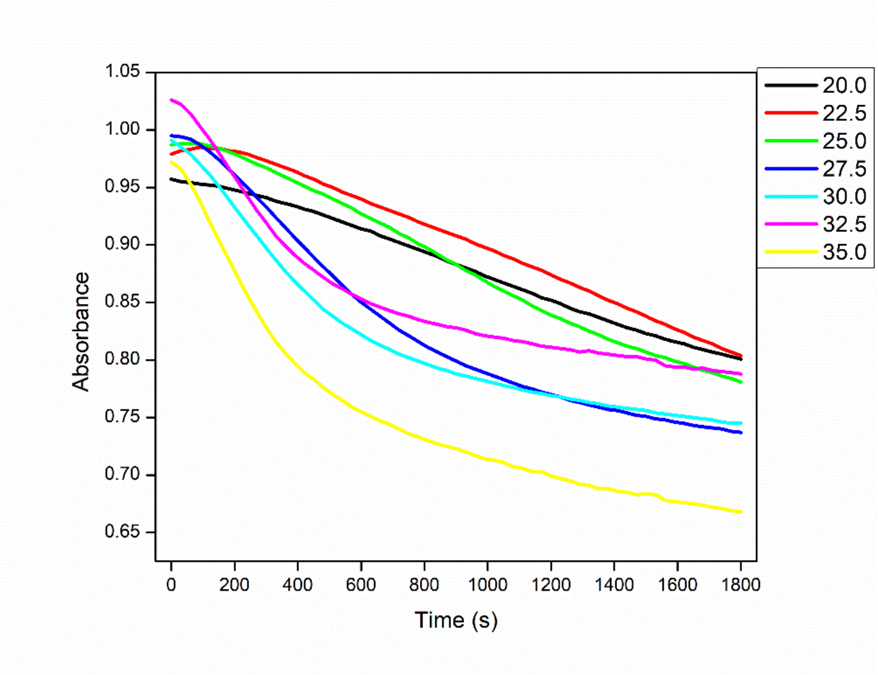

Figure S. 39: Interaction kinetics between the [RuCl(dppb)(bipy)(mepy)]PF_6 complex and AuNPs^{z-} performed by UV-Vis spectroscopy for the 625 nm band with temperature variation (°C). ([Ru^+] Concentration: 1 x 10^{-5} mol L^{-1}).

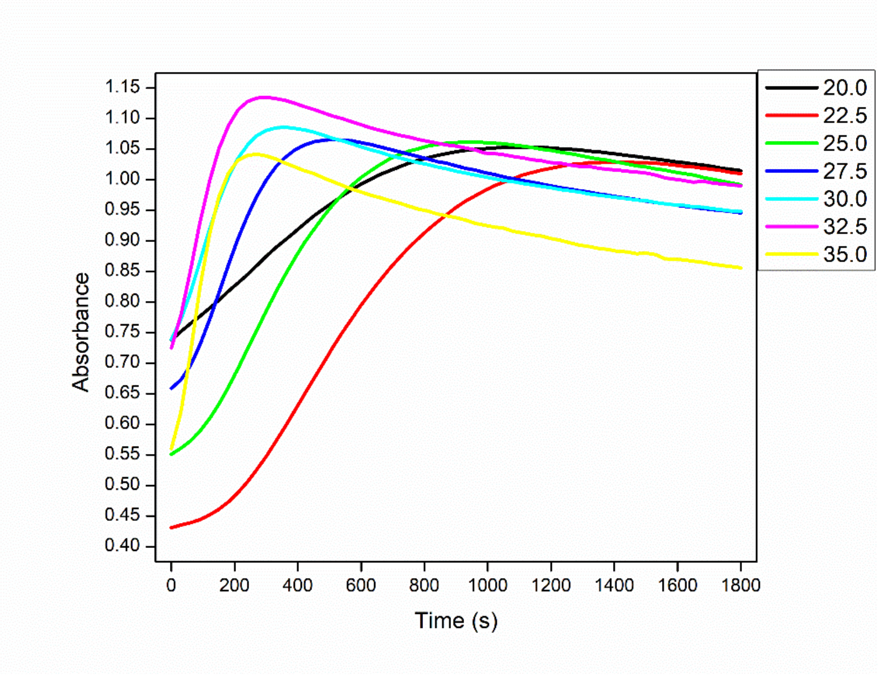

Figure S. 40: Interaction kinetics between the $[RuCl(dppb)(bipy)(tbpy)]PF_6$ complex and $AuNPs^{z-}$ performed by UV-Vis spectroscopy for the 520 nm band with temperature variation (°C). ($[Ru^+]$ Concentration: 1×10^{-5} mol L^{-1}).

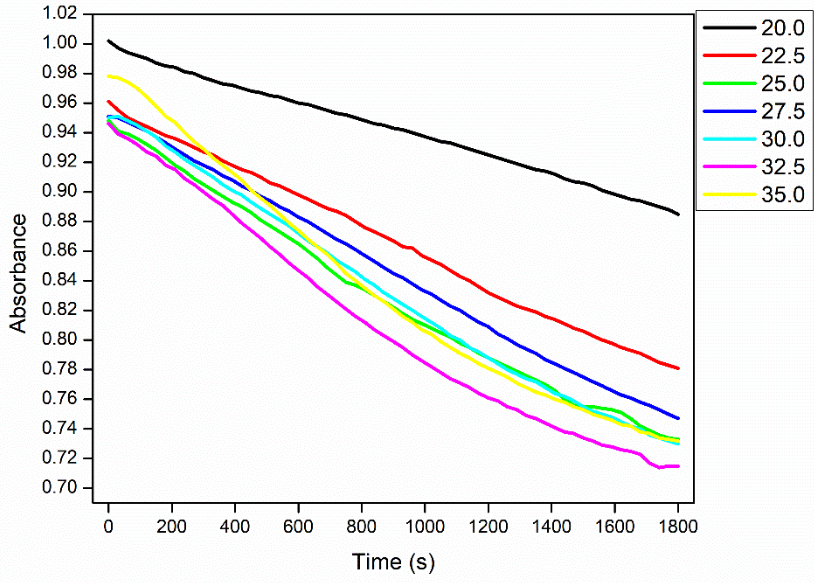

Figure S. 41: Interaction kinetics between the $[RuCl(dppb)(bipy)(tbpy)]PF_6$ complex and $AuNPs^{z-}$ performed by UV-Vis spectroscopy for the 625 nm band with temperature variation (°C). ($[Ru^+]$ Concentration: 1×10^{-5} mol L^{-1}).

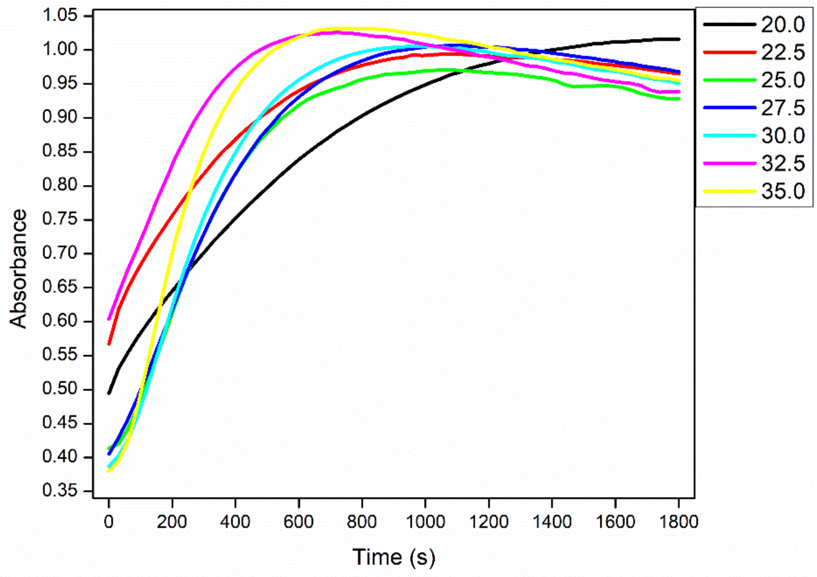

Figure S. 42: Interaction kinetics between the *cis*-[$RuCl_2$(dppb)(bipy)]PF_6 complex and $AuNPs^{z-}$ performed by UV-Vis spectroscopy for the 520 nm band with temperature variation (°C). ([Ru^+] Concentration: 1 x 10^{-5} mol L^{-1}).

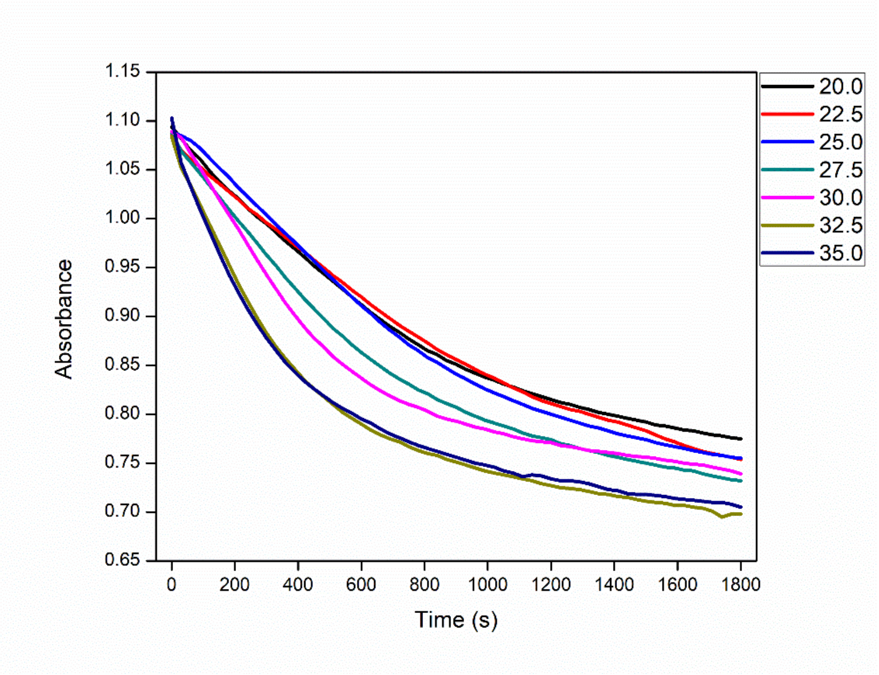

Figure S. 43: Interaction kinetics between the *cis*-[$RuCl_2$(dppb)(bipy)]PF_6 complex and $AuNPs^{z-}$ performed by UV-Vis spectroscopy for the 625 nm band with temperature variation (°C). ([Ru^+] Concentration: 1 x 10^{-5} mol L^{-1}).

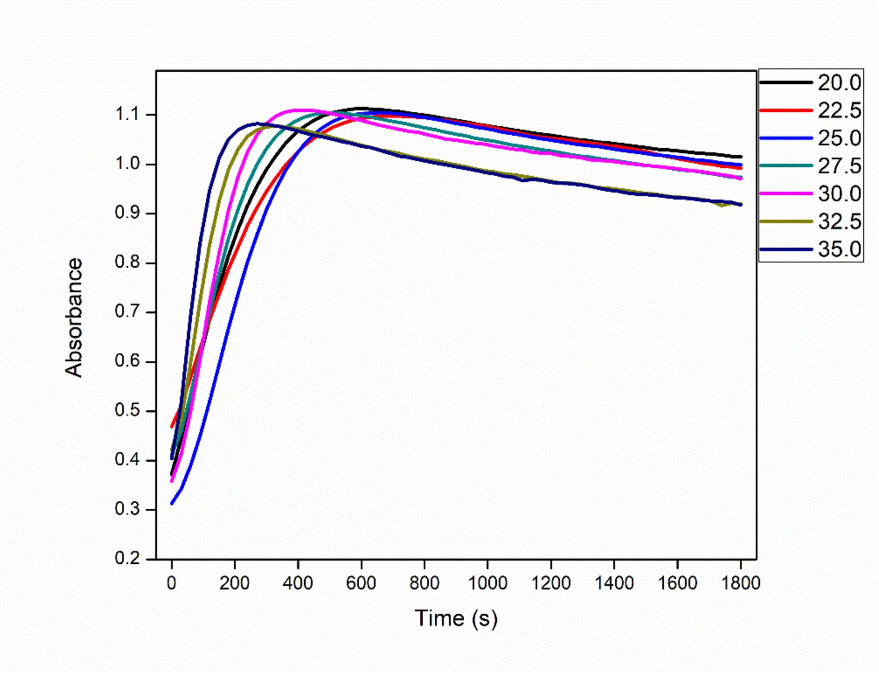

Figure S. 44: Interaction kinetics between the $[Fe(bipy)_3]Cl_2$ complex and AuNPs^{z-} performed by UV-Vis spectroscopy for the 520 nm band with temperature variation. (°C). ([Fe^{+}] Concentration: 1 x 10^{-5} mol L^{-1}).

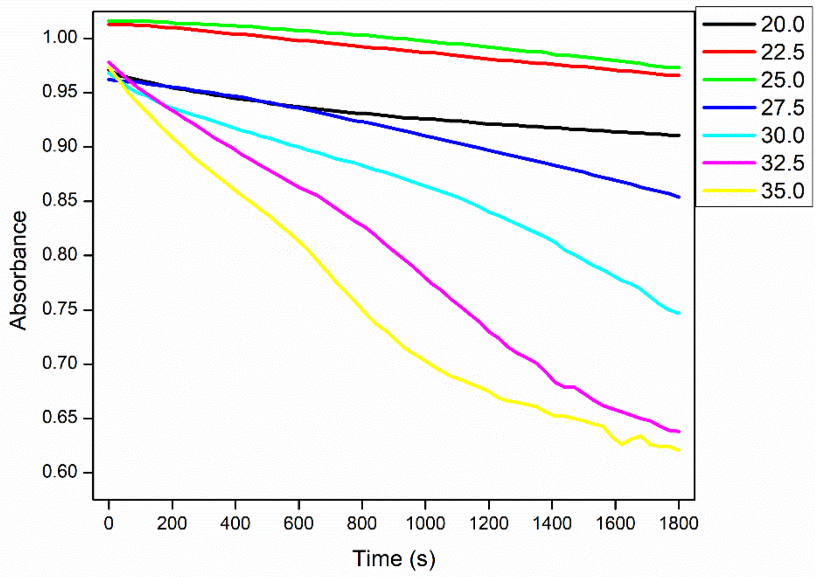

Figure S. 45: Interaction kinetics between the $[Fe(bipy)_3]Cl_2$ complex and AuNPs^{z-} performed by UV-Vis spectroscopy for the 625 nm band with temperature variation (°C). ([Fe^{+}] Concentration: 1 x 10^{-5} mol L^{-1}).

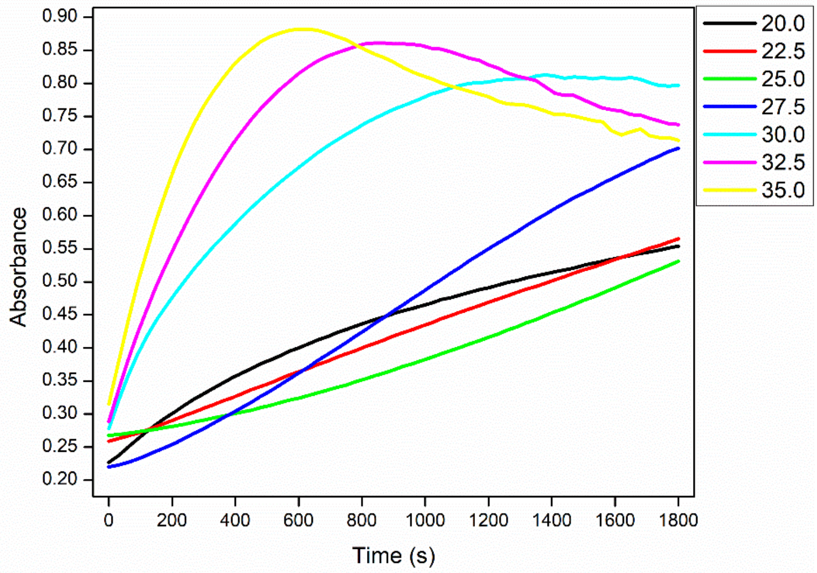

Figure S. 46: Interaction kinetics between the $[Ru(bipy)_3](PF_6)_2$ complex and $AuNPs^{z-}$ performed by UV-Vis spectroscopy for the 520 nm band with temperature variation (°C). ($[Ru^+]$ Concentration: 1×10^{-5} mol L^{-1}).

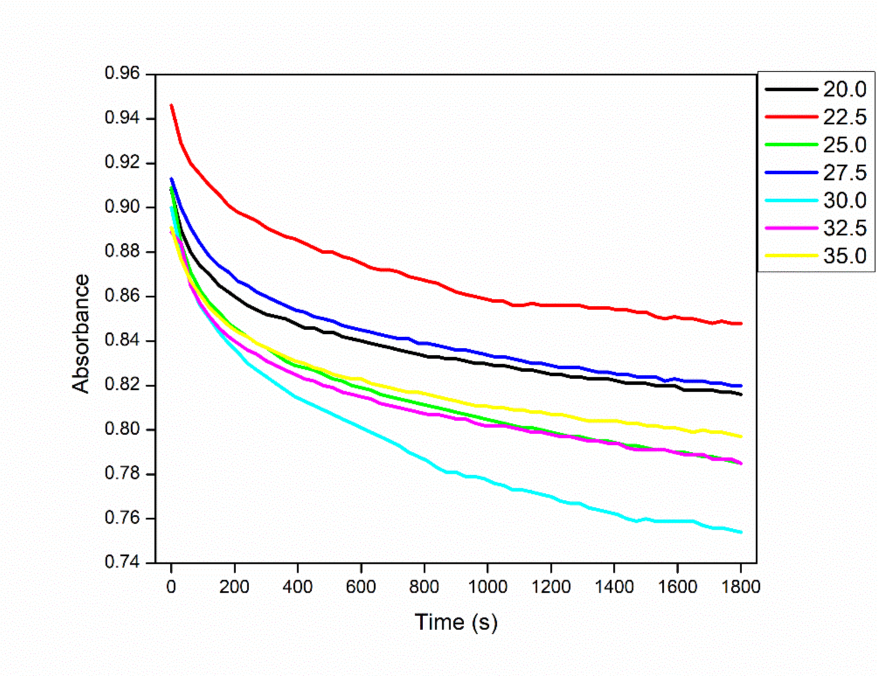

Figure S. 47: Interaction kinetics between the $[Ru(bipy)_3](PF_6)_2$ complex and $AuNPs^{z-}$ performed by UV-Vis spectroscopy for the 625 nm band with temperature variation (°C). ($[Ru^+]$ Concentration: 1×10^{-5} mol L^{-1}).

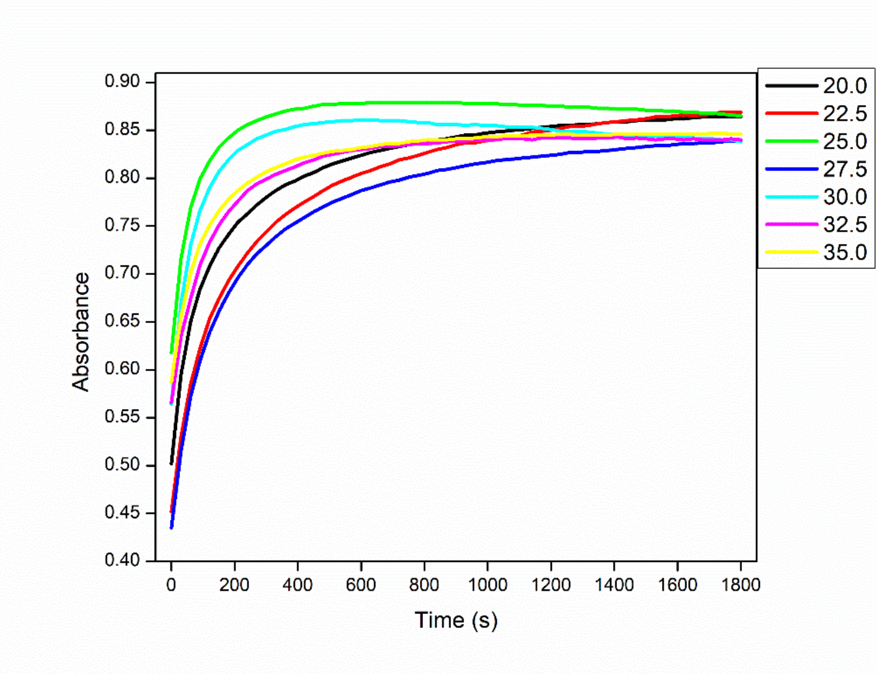

Effect of concentration variation

Table S. 7: *k* rate constants calculated at wavelength **520** nm at constant temperature. *k* unit: s^{-1}.

Volume (µL)	1 (10^{-3})	2 (10^{-4})	3 (10^{-4})	4 (10^{-4})	5 (10^{-3})	6 (10^{-4})	7 (10^{-3})
**150**	1.00	2.39	4.55	1.27	0.61	3.63	0.56
**200**	1.25	3.10	5.37	2.60	0.73	4.43	0.88
**250**	1.92	3.70	5.54	3.59	0.80	5.66	1.01
**300**	2.27	4.15	4.90	4.30	0.84	6.01	1.56
**500**	1.63	5.84	1.17	5.70	1.10	6.84	1.62
**1000**	1.41	7.17	1.33	6.24	1.44	7.68	1.72

Table S. 8: *k* rate constants calculated at wavelength **625** nm at constant temperature for the **flocculation** period. *k* unit: s^{-1}.

Volume (µL)	1 (10^{-3})	2 (10^{-3})	3 (10^{-3})	4 (10^{-3})	5	6 (10^{-5})	7
**150**	6.81	2.57	4.67	1.08	-	-	-
**200**	-	3.09	2.93	1.90	-	2.02	-
**250**	-	3.31	2.89	2.19	-	2.41	-
**300**	-	3.09	-	6.85	-	3.87	-
**500**	-	-	-	-	-	4.26	-
**1000**	-	-	-	-	-	5.84	-

Table S. 9: *k* rate constants calculated at wavelength **625** nm at constant temperature for the **agglomeration** period. *k* unit: s^{-1}.

Volume (µL)	1 (10^{-4})	2 (10^{-4})	3 (10^{-4})	4 (10^{-4})	5 (10^{-4})	6 (10^{-3})	7 (10^{-4})
**150**	1.51	1.20	1.24	-	0.36	2.05	1.53
**200**	1.81	1.22	1.21	1.35	0.58	3.22	1.77
**250**	1.90	1.43	1.19	2.00	0.81	5.38	2.92
**300**	1.97	1.54	1.78	2.93	0.98	5.45	3.14
**500**	2.04	3.95	1.38	2.91	1.08	5.77	3.29
**1000**	2.13	2.52	1.29	3.50	1.09	7.39	3.52

Figure S. 48: Interaction kinetics between the $[RuCl(dppb)(bipy)(py)]PF_6$ complex and $AuNPs^{z-}$ performed by UV-Vis spectroscopy for the 520 nm band with concentration variation (μL). (Temperature: 25.0 °C).

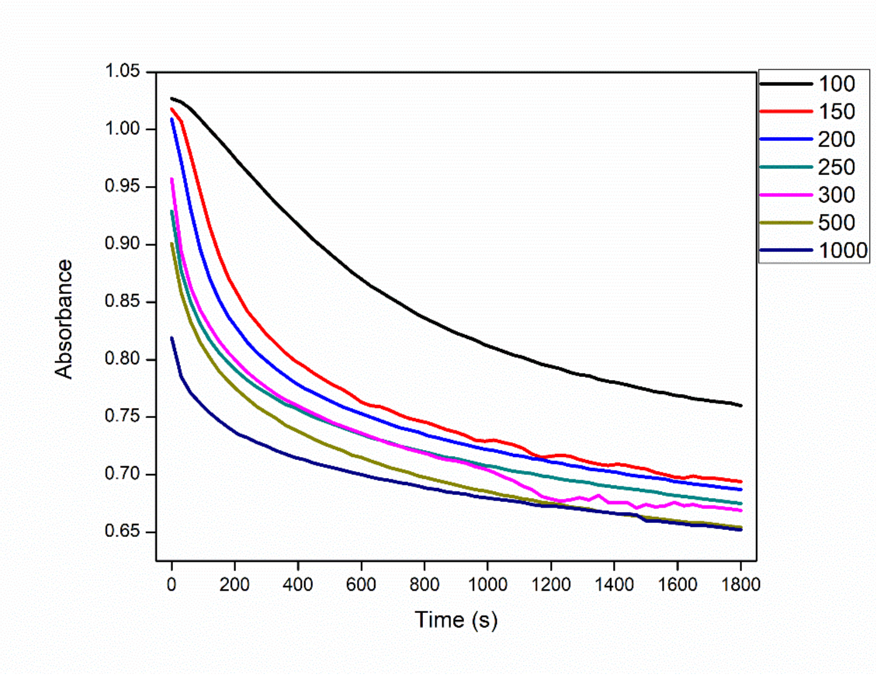

Figure S. 49: Interaction kinetics between the $[RuCl(dppb)(bipy)(py)]PF_6$ complex and $AuNPs^{z-}$ performed by UV-Vis spectroscopy for the 625 nm band with concentration variation (μL). (Temperature: 25.0 °C).

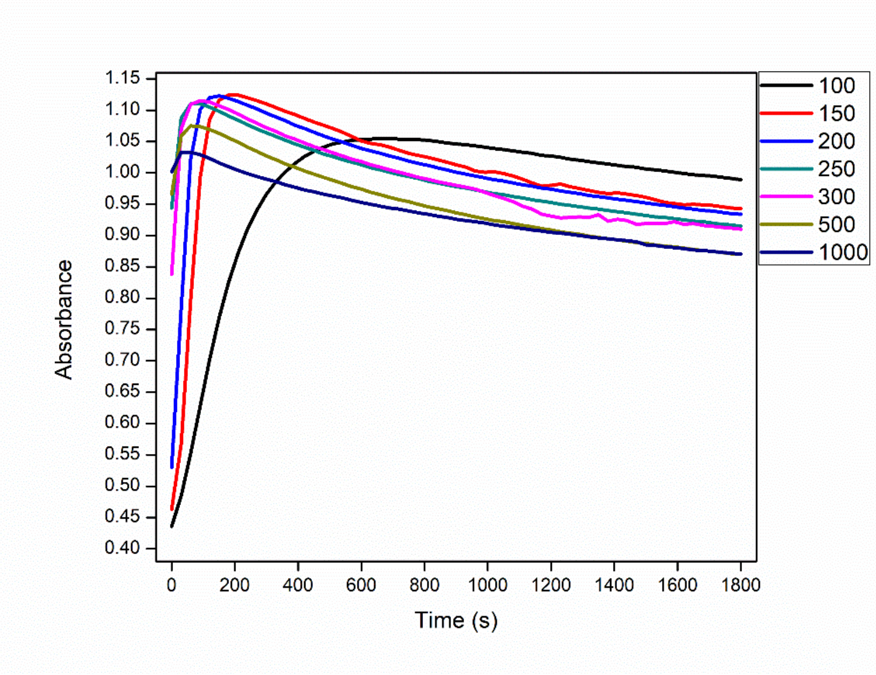

Figure S. 50: Interaction kinetics between the [RuCl(dppb)(bipy)(vpy)]PF_6 complex and $AuNPs^{z-}$ performed by UV-Vis spectroscopy for the 520 nm band with concentration variation (µL). ([Ru^+] Concentration: 1 x 10^{-5} mol L^{-1}). (Temperature: 25.0 °C).

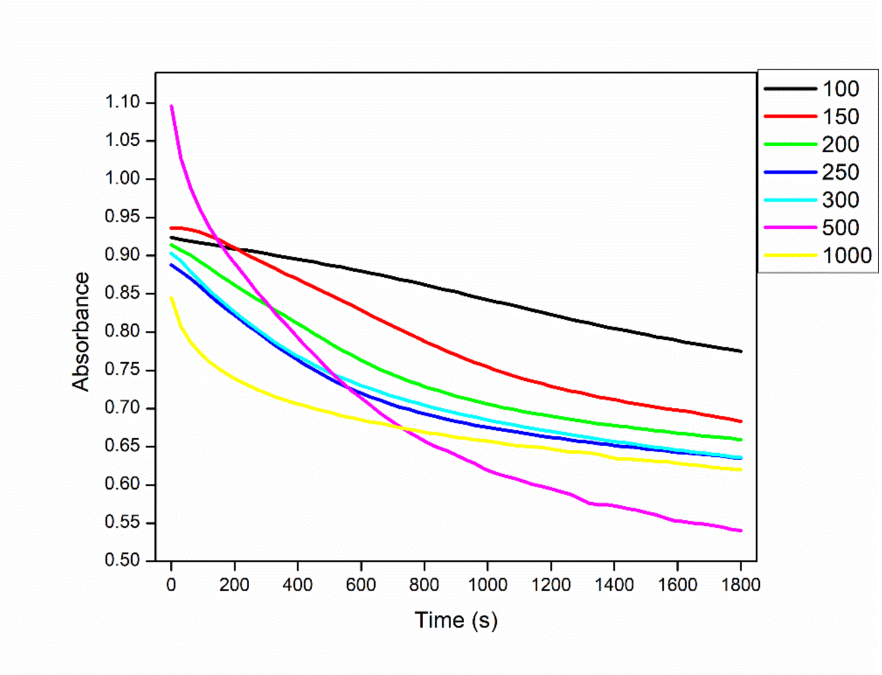

Figure S. 51: Interaction kinetics between the [RuCl(dppb)(bipy)(vpy)]PF_6 complex and $AuNPs^{z-}$ performed by UV-Vis spectroscopy for the 625 nm band with concentration variation (µL). ([Ru^+] Concentration: 1 x 10^{-5} mol L^{-1}). (Temperature: 25.0 °C).

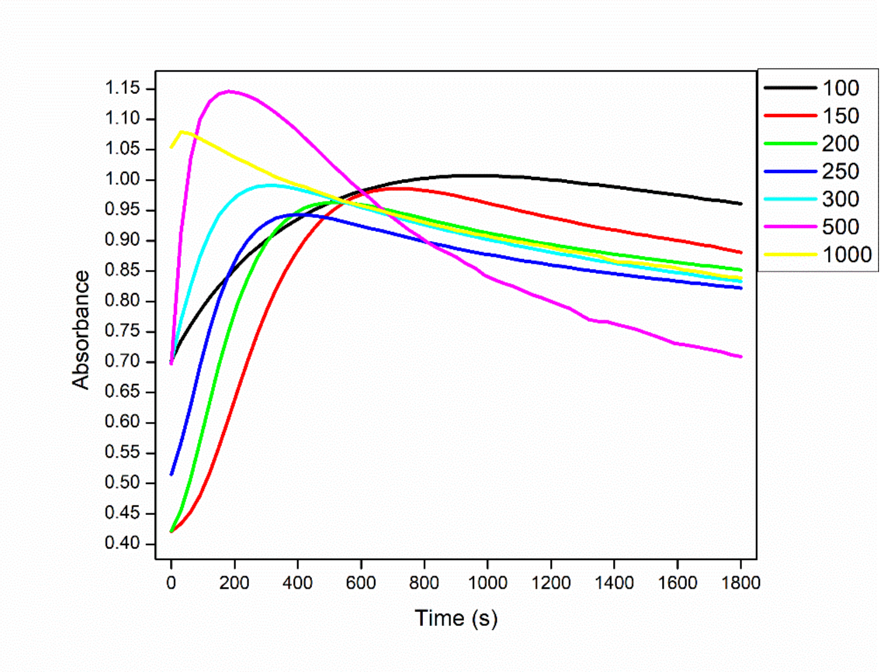

Figure S. 52: Interaction kinetics between the [RuCl(dppb)(bipy)(mepy)]PF_6 complex and $AuNPs^{z-}$ performed by UV-Vis spectroscopy for the 520 nm band with concentration variation (µL). ([Ru^+] Concentration: 1 x 10^{-5} mol L^{-1}). (Temperature: 25.0 °C).

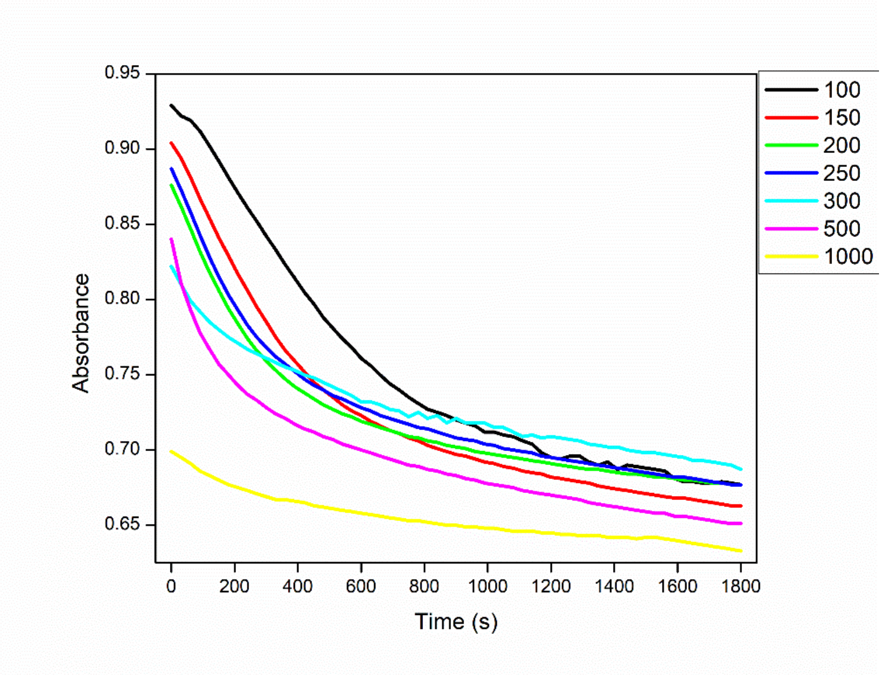

Figure S. 53: Interaction kinetics between the [RuCl(dppb)(bipy)(mepy)]PF_6 complex and $AuNPs^{z-}$ performed by UV-Vis spectroscopy for the 625 nm band with concentration variation (µL). ([Ru^+] Concentration: 1 x 10^{-5} mol L^{-1}). (Temperature: 25.0 °C).

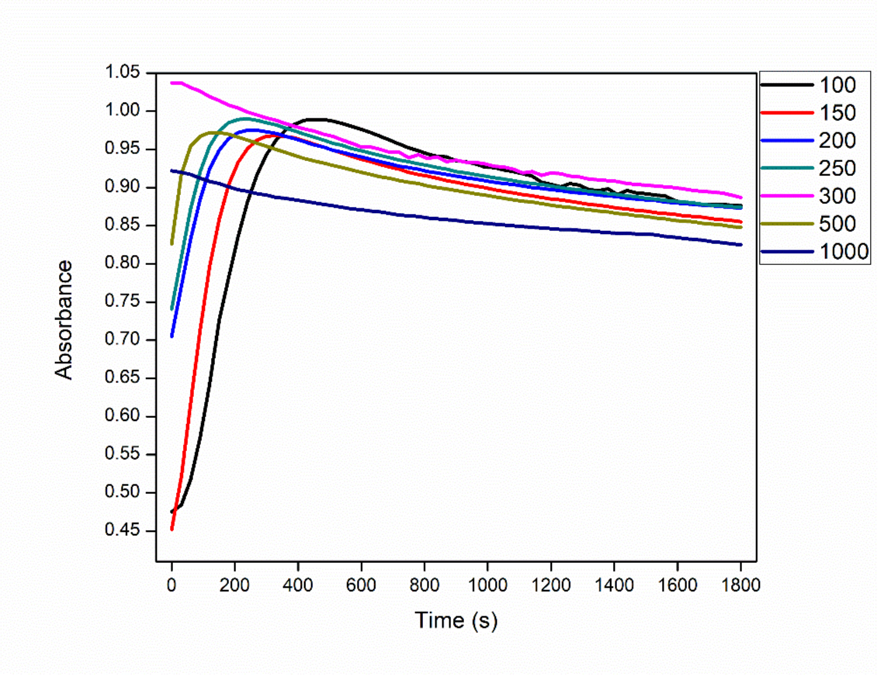

Figure S. 54: Interaction kinetics between the [RuCl(dppb)(bipy)(tbpy)]PF_6 complex and $AuNPs^{Z-}$ performed by UV-Vis spectroscopy for the 520 nm band with concentration variation (µL). (Temperature: 25.0 °C).

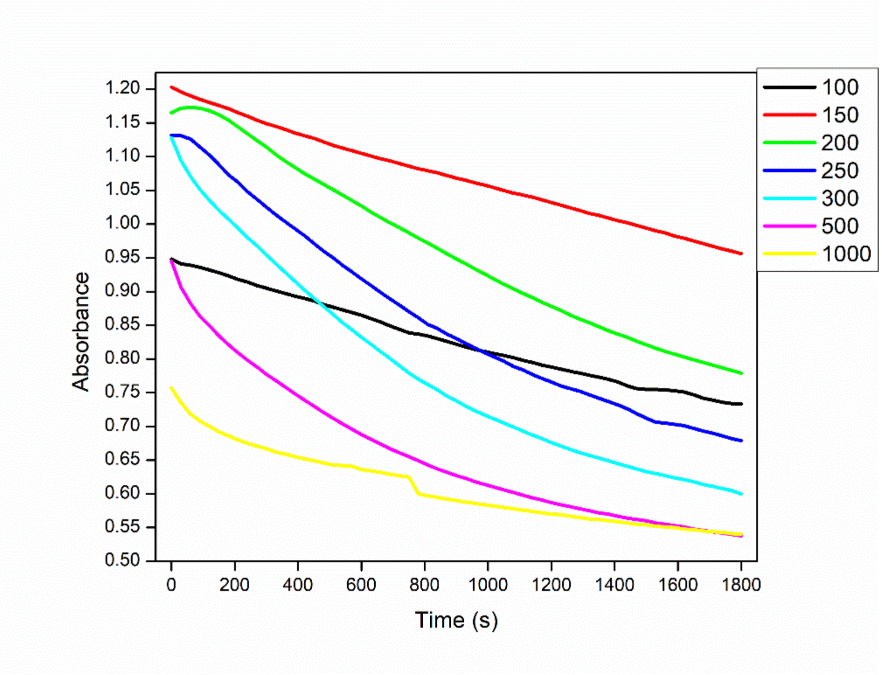

Figure S. 55: Interaction kinetics between the [RuCl(dppb)(bipy)(tbpy)]PF_6 complex and $AuNPs^{Z-}$ performed by UV-Vis spectroscopy for the 625 nm band with concentration variation (µL). (Temperature: 25.0 °C).

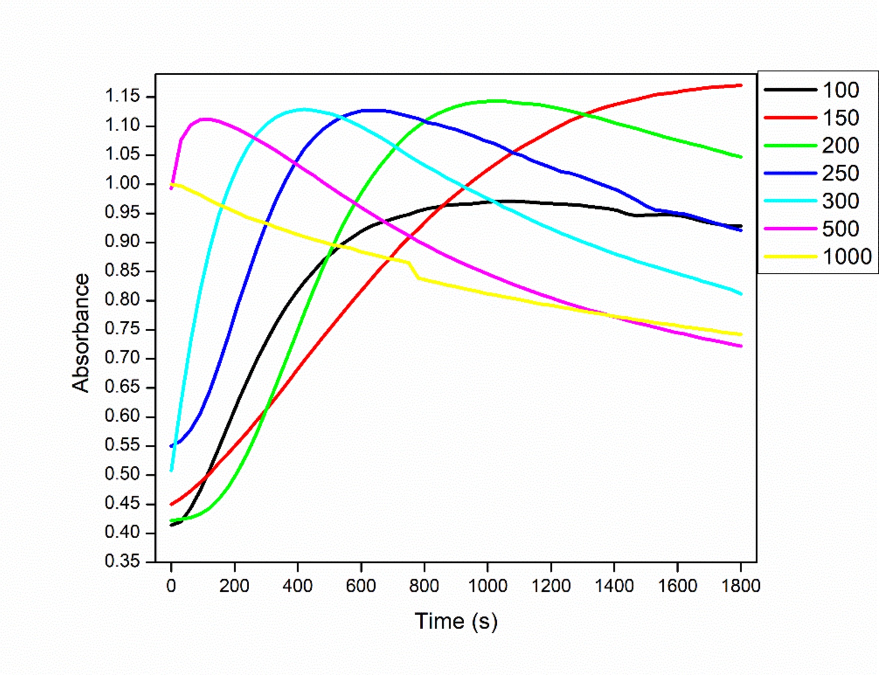

Figure S. 56: Interaction kinetics between the *cis*-[RuCl$_2$(dppb)(bipy)]PF$_6$ complex and AuNPs^{z-} performed by UV-Vis spectroscopy for the 520 nm band with concentration variation (µL). (Temperature: 25.0 °C).

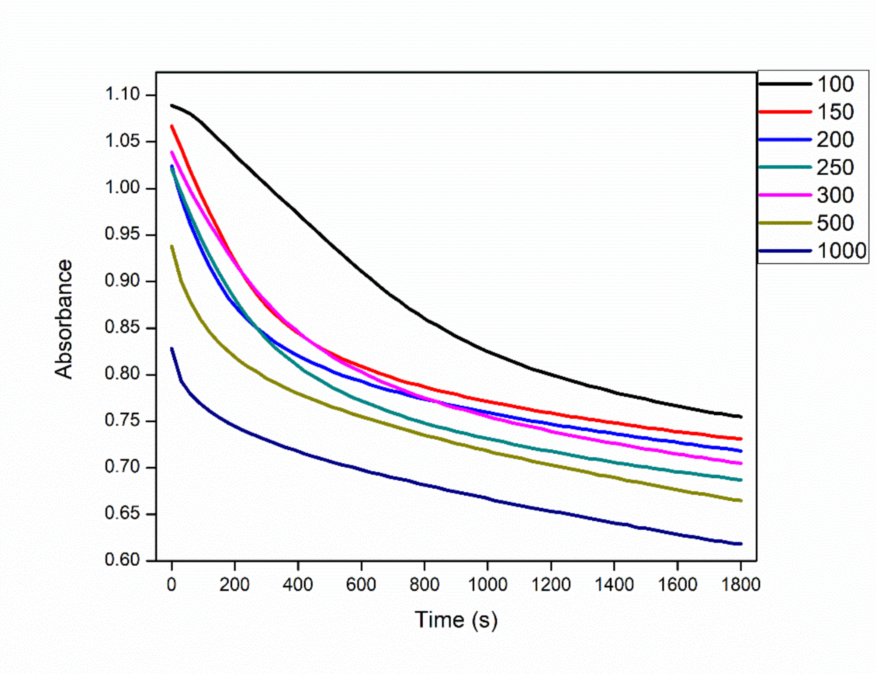

Figure S. 57: Interaction kinetics between the *cis*-[RuCl$_2$(dppb)(bipy)]PF$_6$ complex and AuNPs^{z-} performed by UV-Vis spectroscopy for the 625 nm band with concentration variation (µL). (Temperature: 25.0 °C).

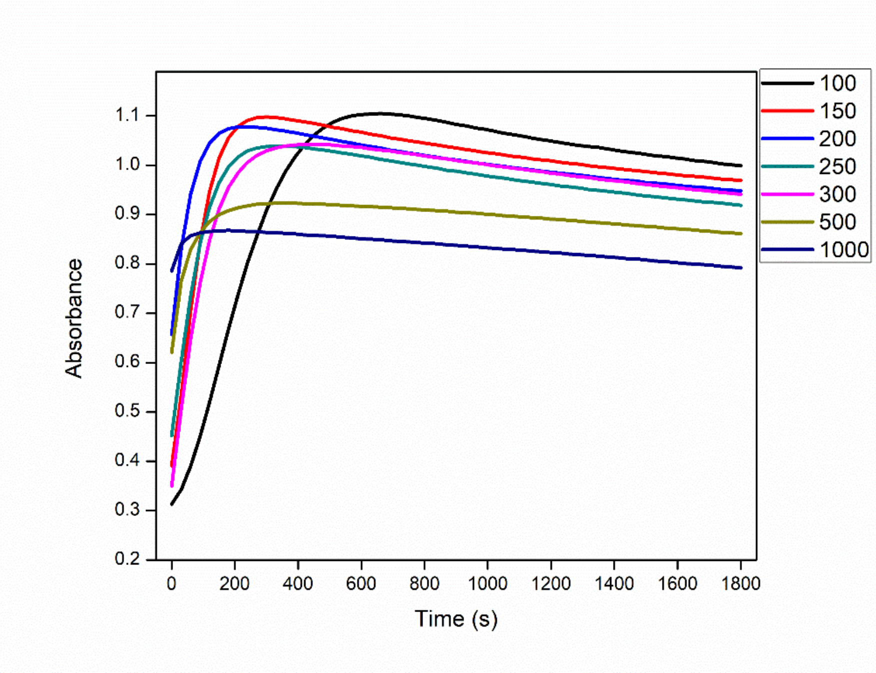

Figure S. 58: Interaction kinetics between the $[Fe(bipy)_3]Cl_2$ complex and $AuNPs^{z-}$ performed by UV-Vis spectroscopy for the 520 nm band with concentration variation (µL). (Temperature: 25.0 °C).

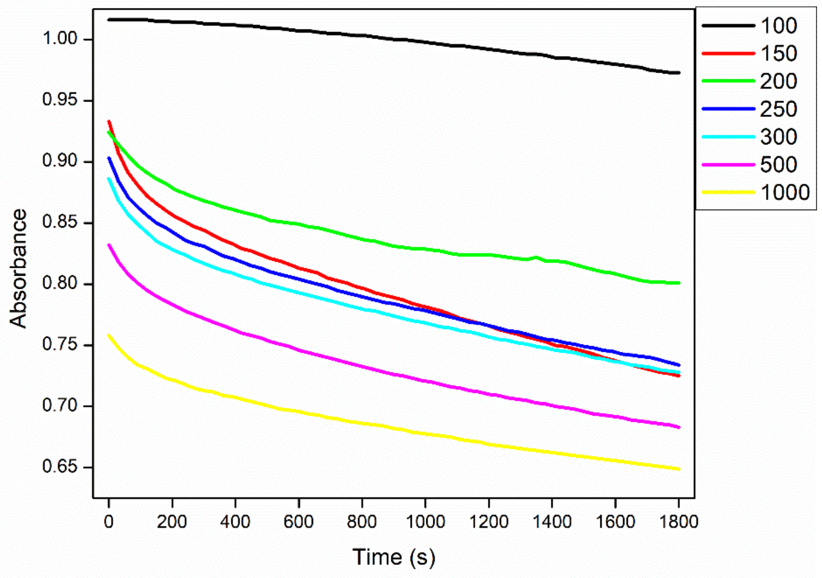

Figure S. 59: Interaction kinetics between the $[Fe(bipy)_3]Cl_2$ complex and $AuNPs^{z-}$ performed by UV-Vis spectroscopy for the 625 nm band with concentration variation (µL). (Temperature: 25.0 °C).

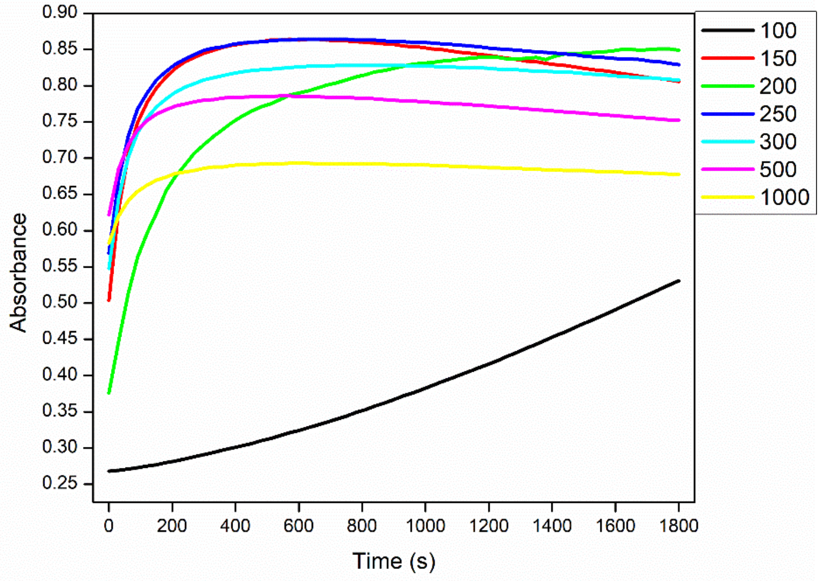

Figure S. 60: Interaction kinetics between the $[Ru(bipy)_3](PF_6)_2$ complex and $AuNPs^{z-}$ performed by UV-Vis spectroscopy for the 520 nm band with concentration variation (µL). (Temperature: 25.0 °C).

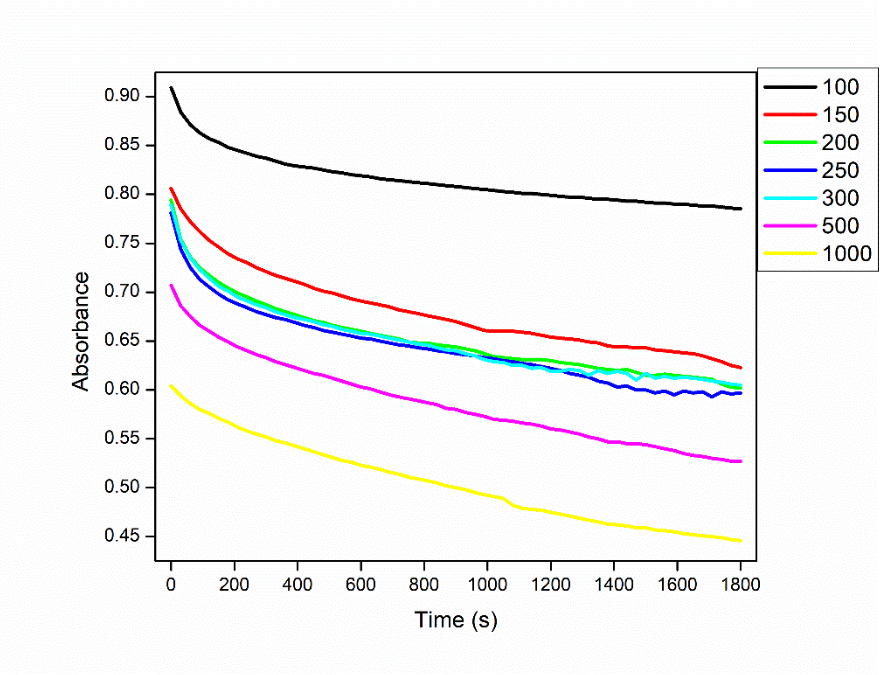

Figure S. 61: Interaction kinetics between the $[Ru(bipy)_3](PF_6)_2$ complex and $AuNPs^{z-}$ performed by UV-Vis spectroscopy for the 625 nm band with concentration variation (µL). (Temperature: 25.0 °C).

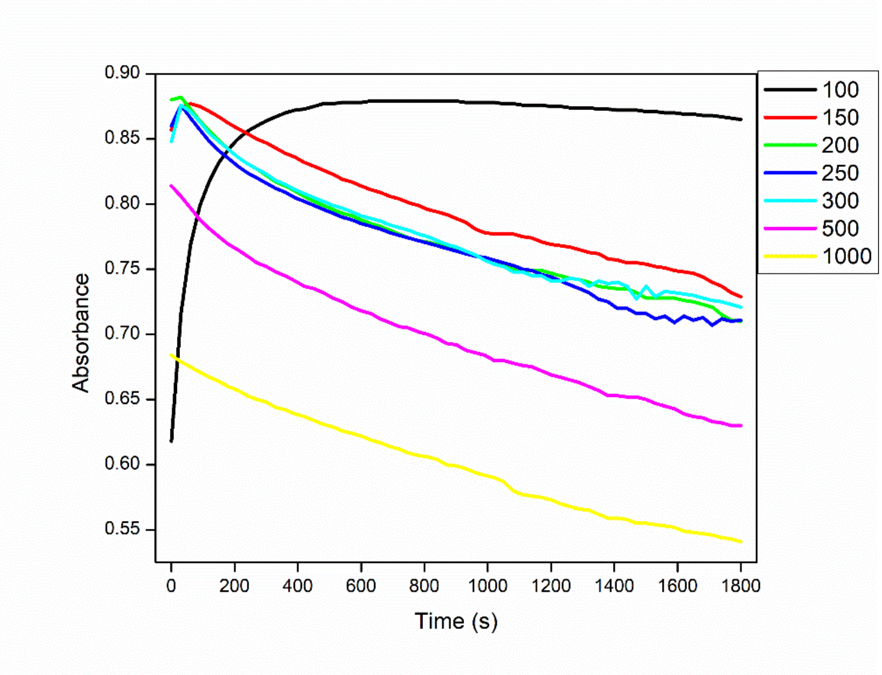

Langmuir isotherm for M^{z+} and $AuNPs^{z-}$ interactions

Figure S. 62:Langmuir isotherm for the interaction of the $[Fe(bipy)_3]Cl_2$ complex with $AuNPs^{z-}$ (first measure).

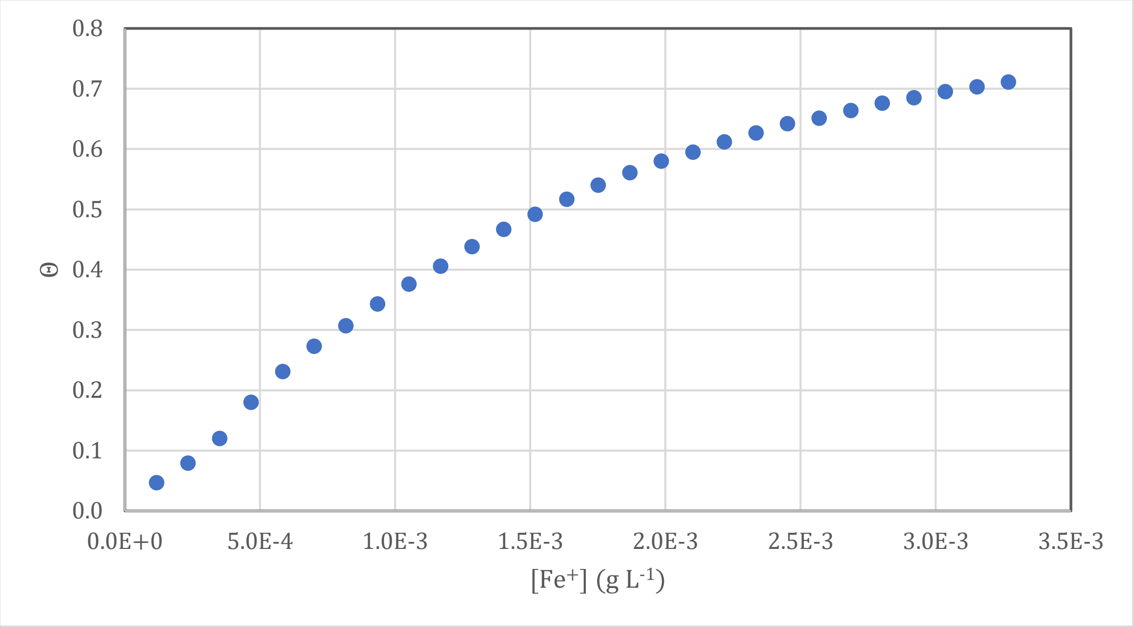

Figure S. 63: Langmuir isotherm for the interaction of the $[Fe(bipy)_3]Cl_2$ complex with $AuNPs^{z-}$ (second measure).

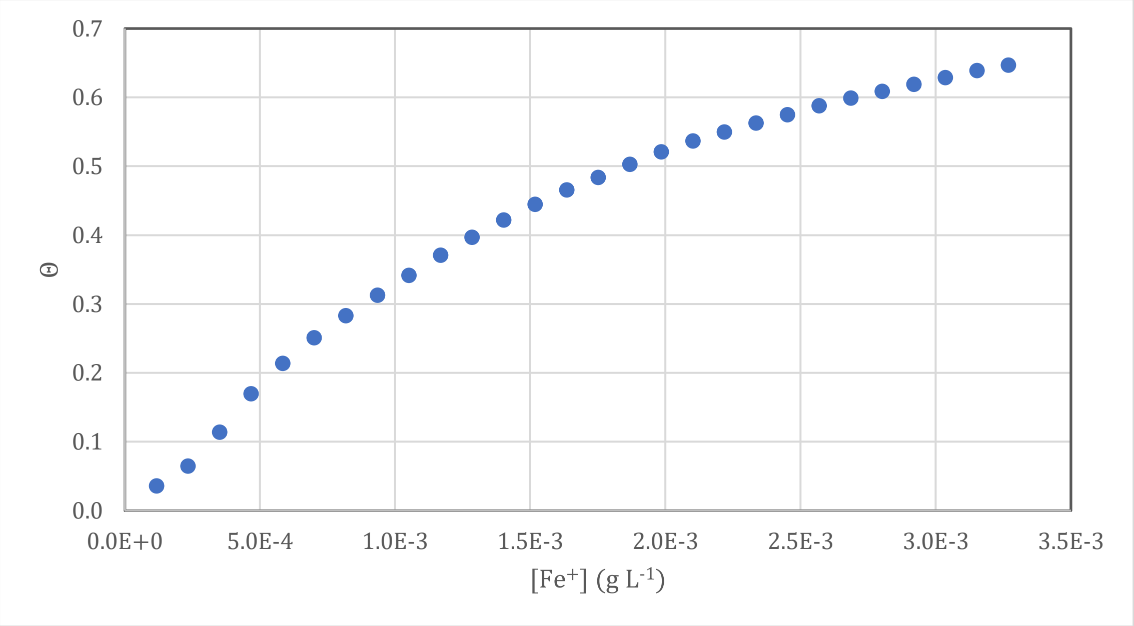

Figure S. 64: Langmuir isotherm for the interaction of the $[Fe(bipy)_3]Cl_2$ complex with $AuNPs^{z-}$ (third measure).

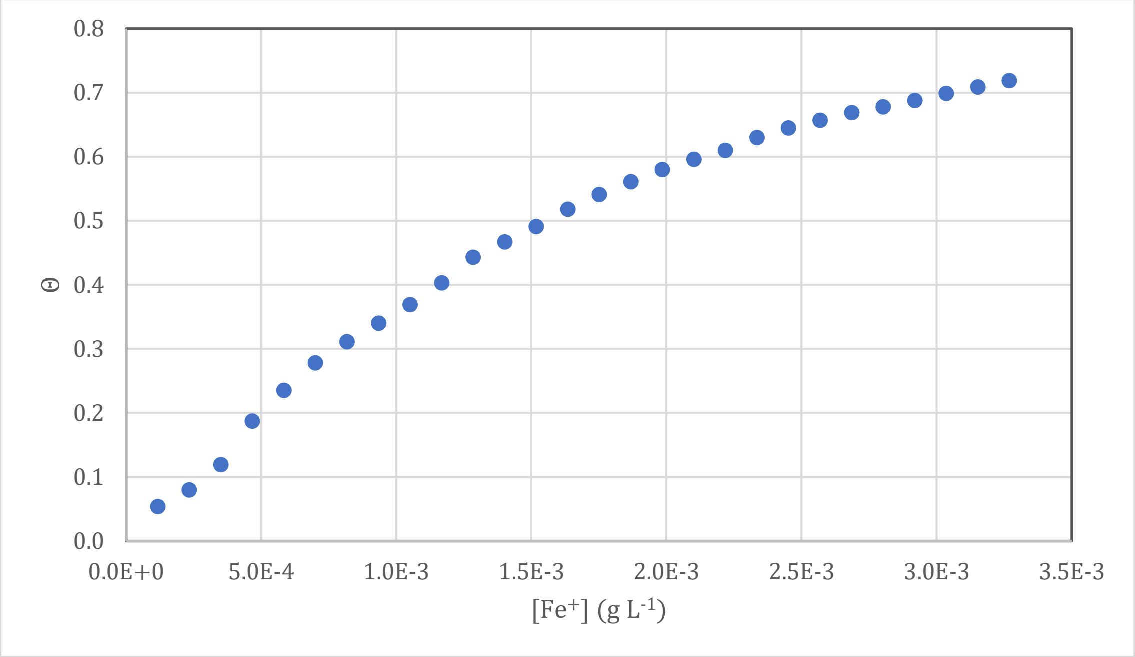

Figure S. 65: Langmuir isotherm for the interaction of the $[Ru(bipy)_3](PF_6)_2$ complex with $AuNPs^{z-}$ (first measure).

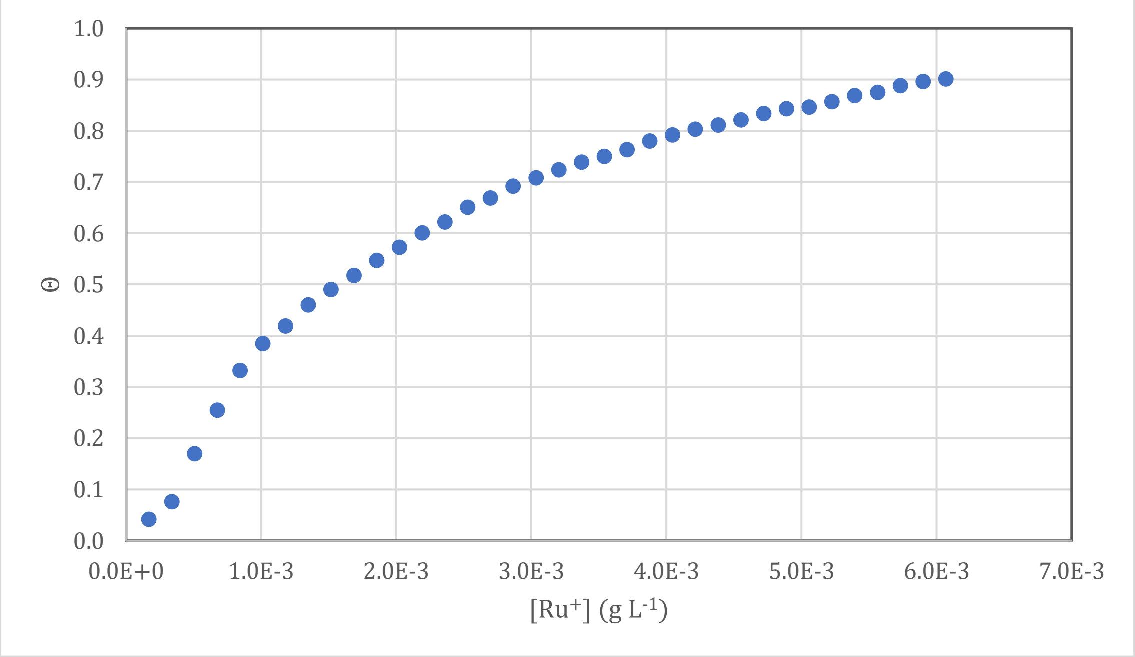

Figure S. 66: Langmuir isotherm for the interaction of the $[Ru(bipy)_3](PF_6)_2$ complex with $AuNPs^{z-}$ (second measure).

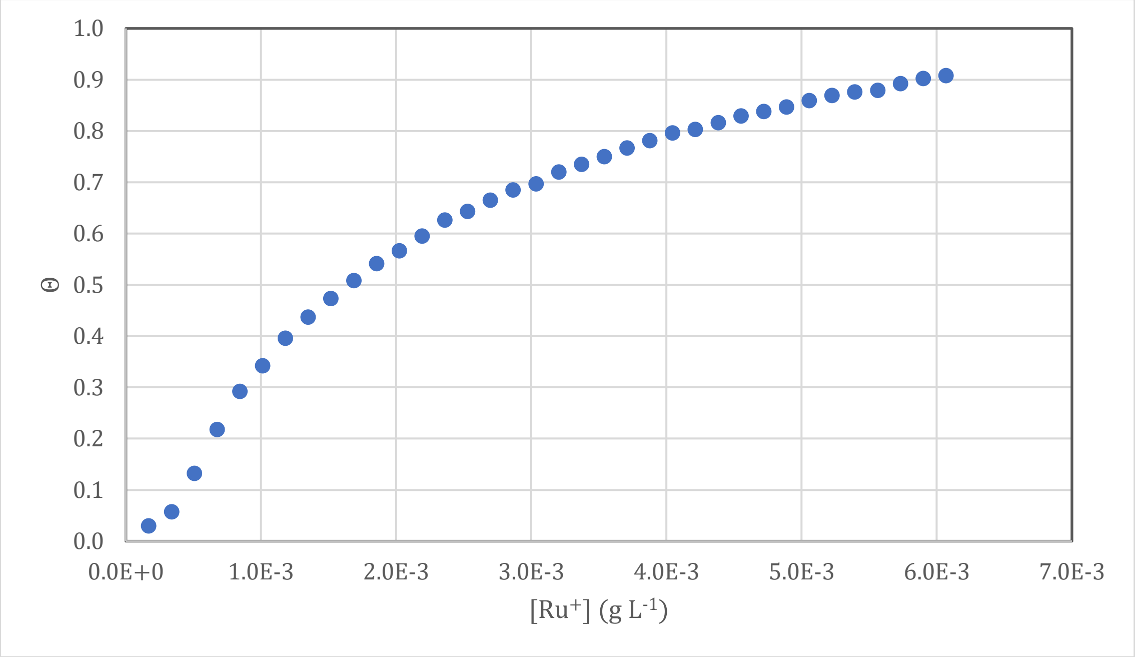

Figure S. 67: Langmuir isotherm for the interaction of the $[Ru(bipy)_3](PF_6)_2$ complex with $AuNPs^{z-}$ (third measure).

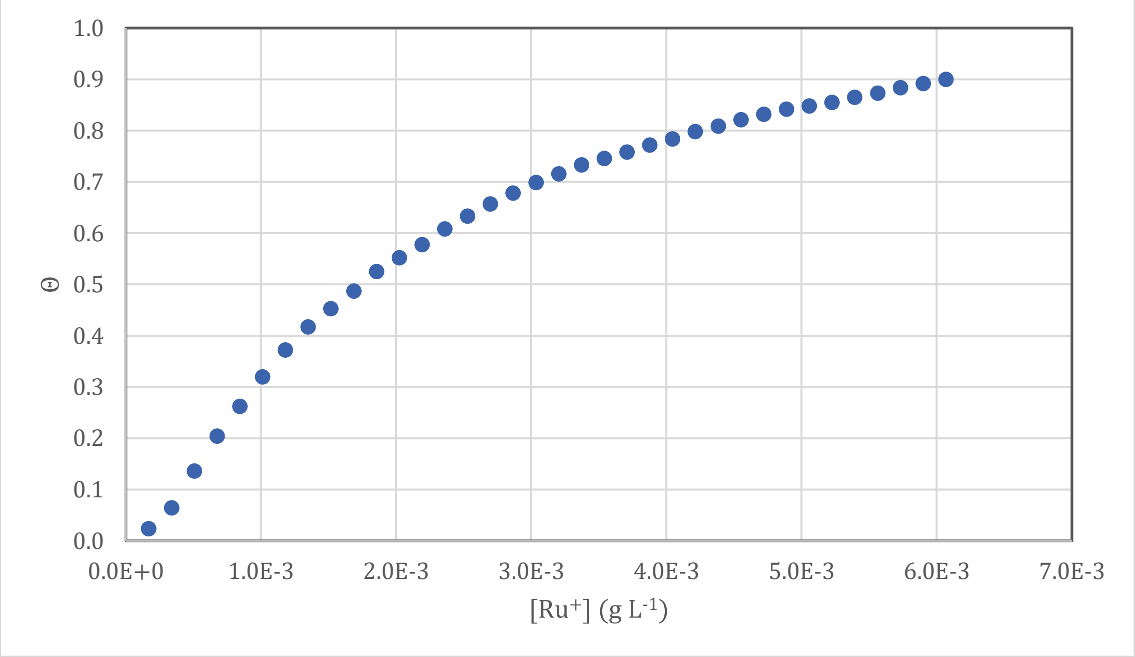

Figure S. 68: Langmuir isotherm for the interaction of the [RuCl(dppb)(bipy)(py)]PF_6 complex with $AuNPs^{z-}$ (first measure).

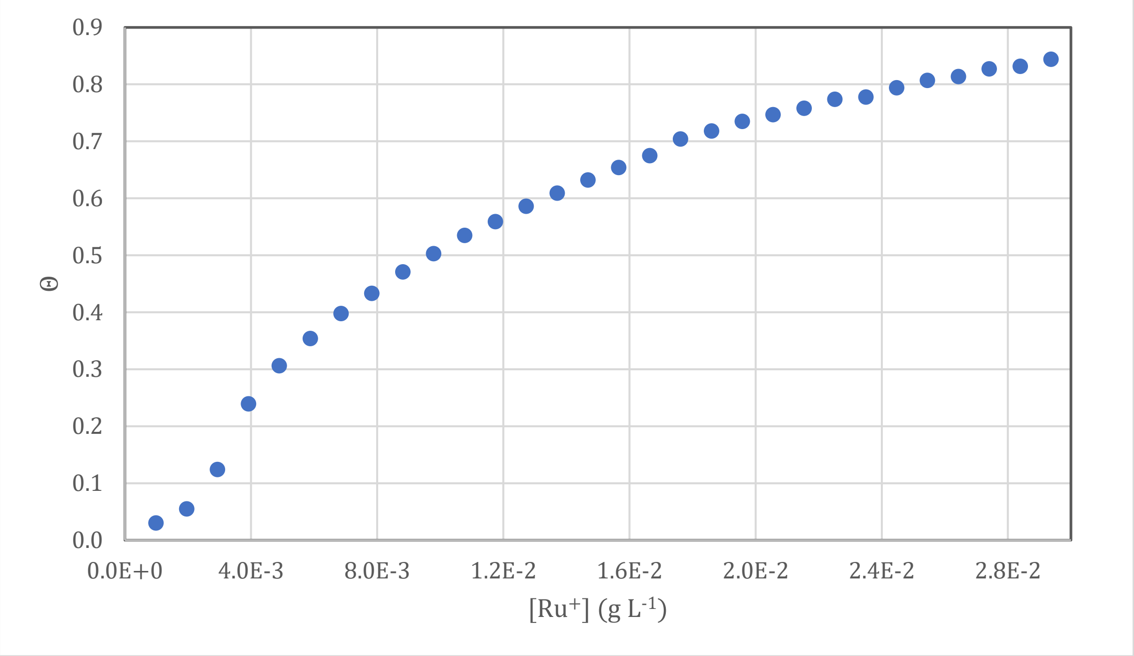

Figure S. 69: Langmuir isotherm for the interaction of the [RuCl(dppb)(bipy)(py)]PF_6 complex with $AuNPs^{z-}$ (second measure).

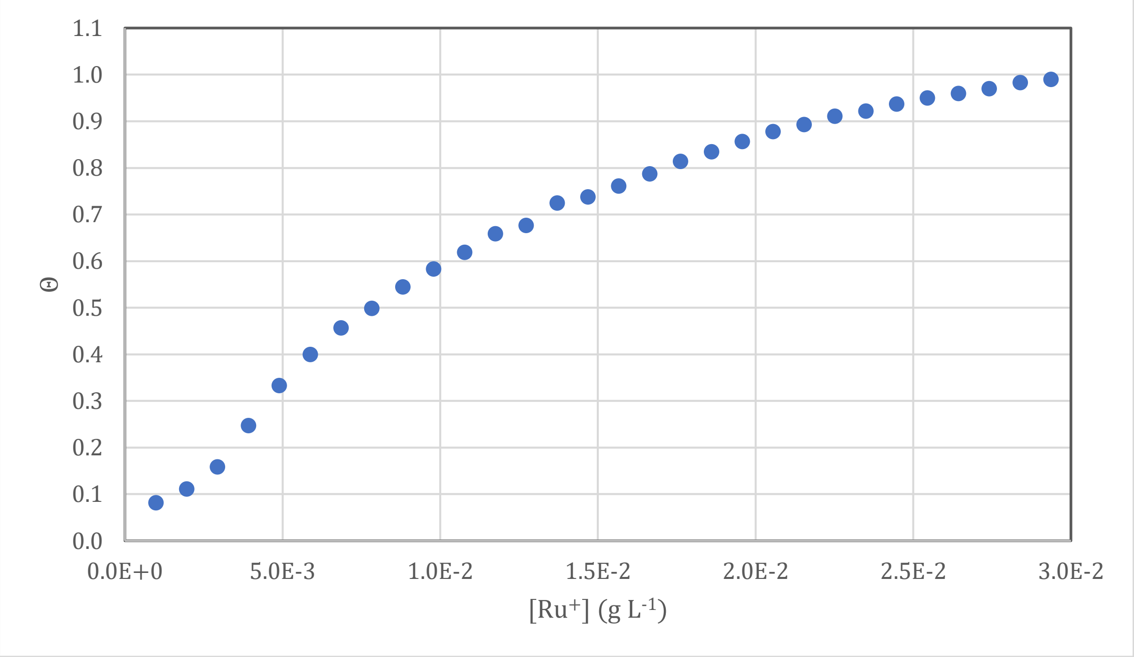

Figure S. 70: Langmuir isotherm for the interaction of the [RuCl(dppb)(bipy)(py)]PF_6 complex with AuNPs^{z-} (third measure).

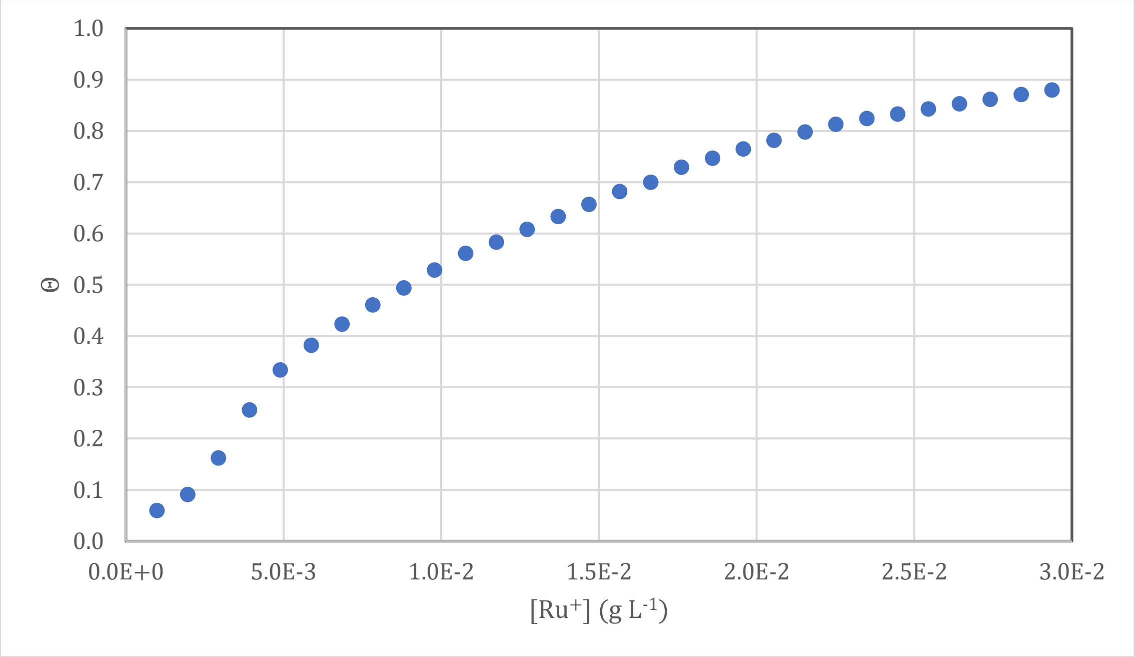

Figure S. 71: Langmuir isotherm for the interaction of the [RuCl(dppb)(bipy)(*t*bpy)]PF_6 complex with AuNPs^{z-} (first measure).

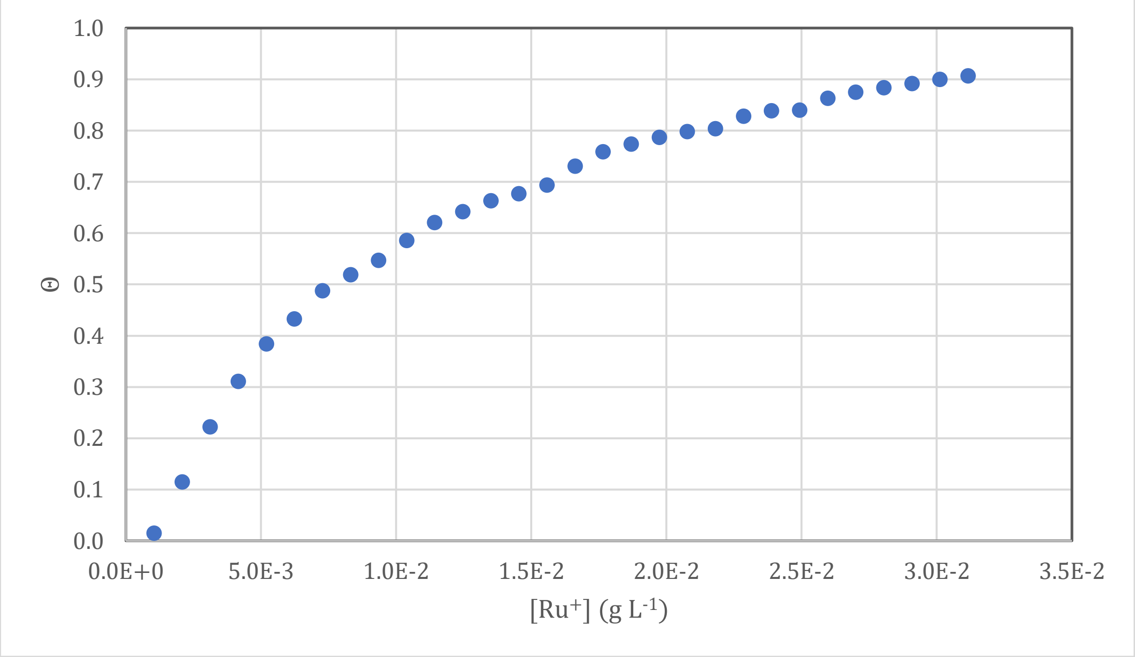

Figure S. 72: Langmuir isotherm for the interaction of the [RuCl(dppb)(bipy)(*t*bpy)]PF_6 complex with AuNPs^{z-} (second measure).

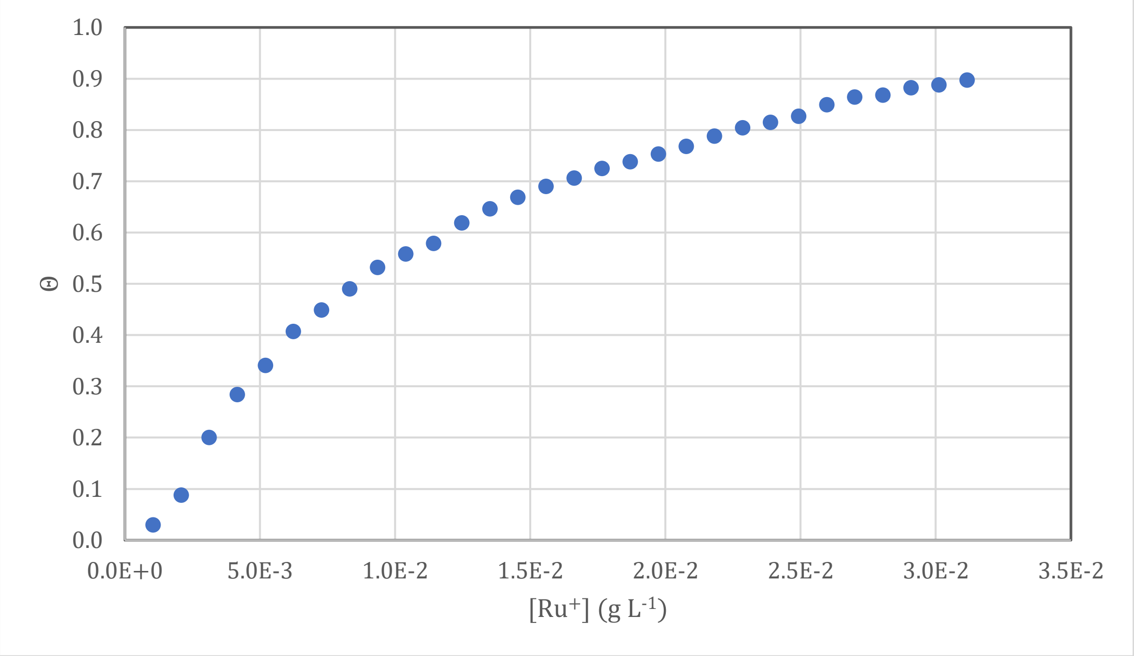

Figure S. 73: Langmuir isotherm for the interaction of the [RuCl(dppb)(bipy)(*t*bpy)]PF_6 complex with AuNPs^{z-} (third measure).

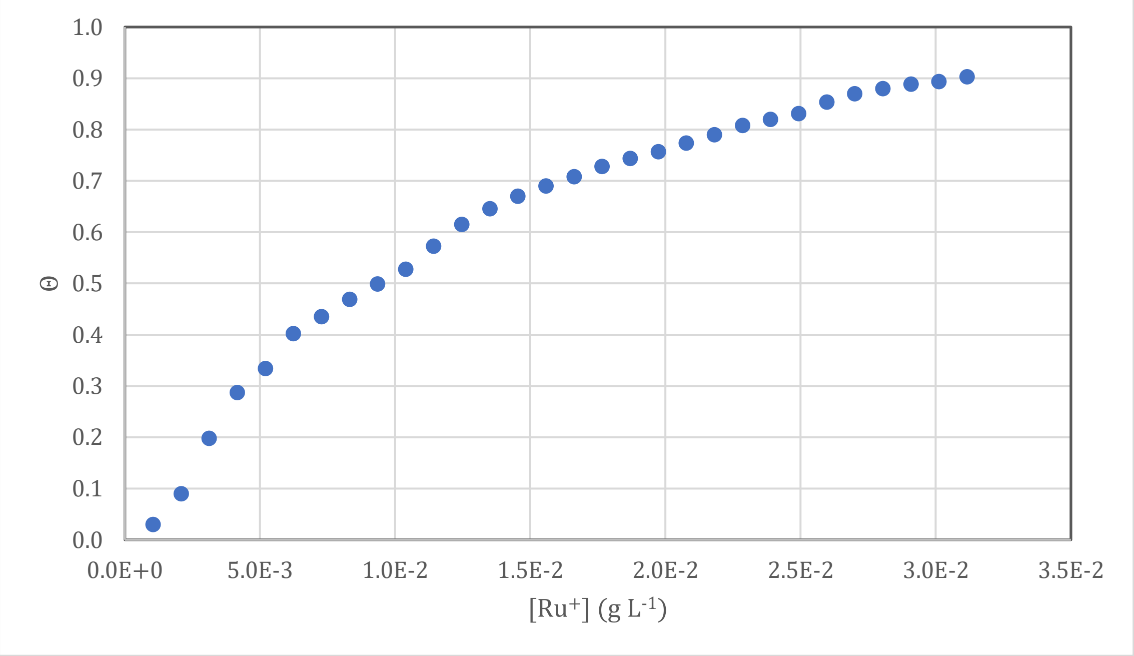

Figure S. 74: Langmuir isotherm for the interaction of the [RuCl(dppb)(bipy)(mepy)]PF_6 complex with $AuNPs^{z-}$ (first measure).

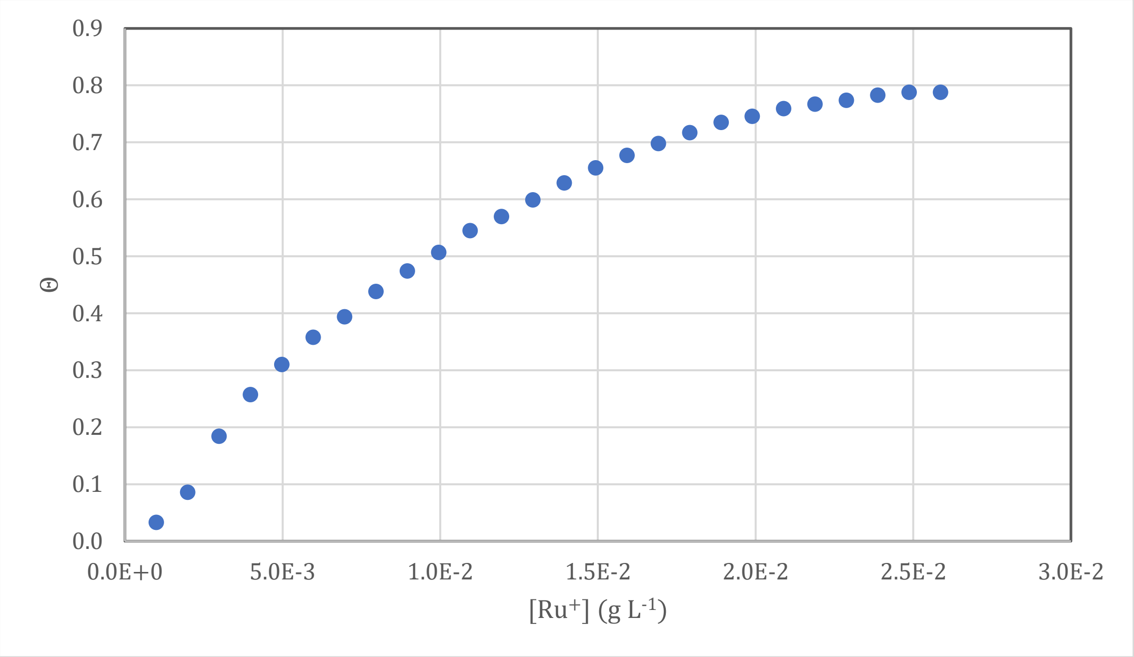

Figure S. 75: Langmuir isotherm for the interaction of the [RuCl(dppb)(bipy)(mepy)]PF_6 complex with $AuNPs^{z-}$ (second measure).

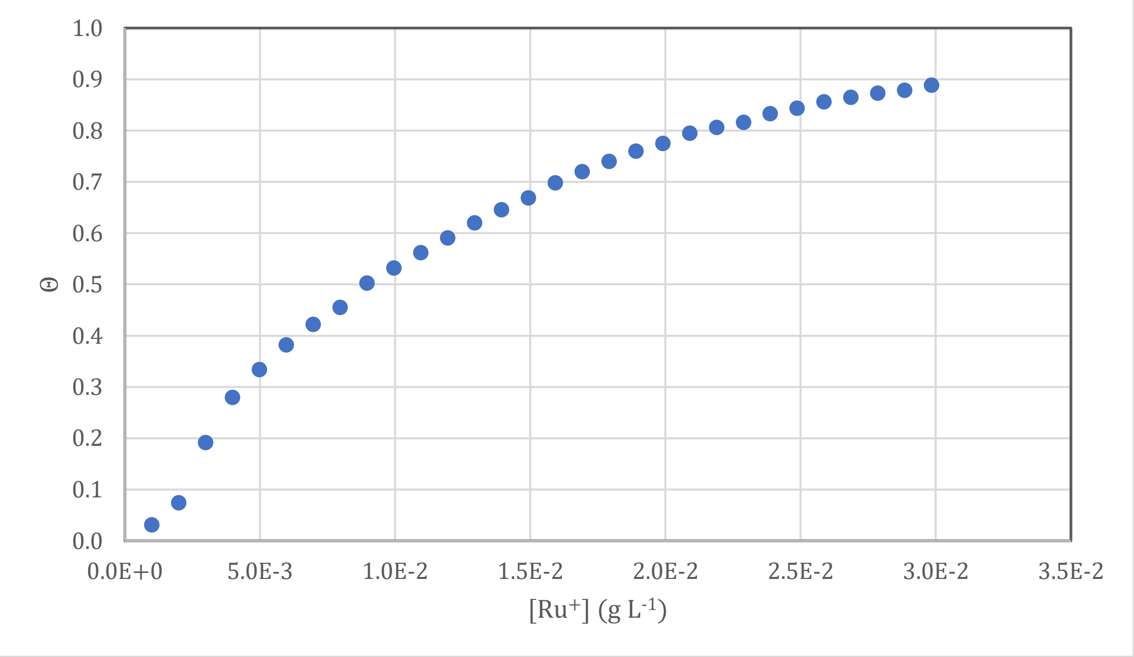

Figure S. 76: Langmuir isotherm for the interaction of the [RuCl(dppb)(bipy)(mepy)]PF_6 complex with $AuNPs^{z-}$ (third measure).

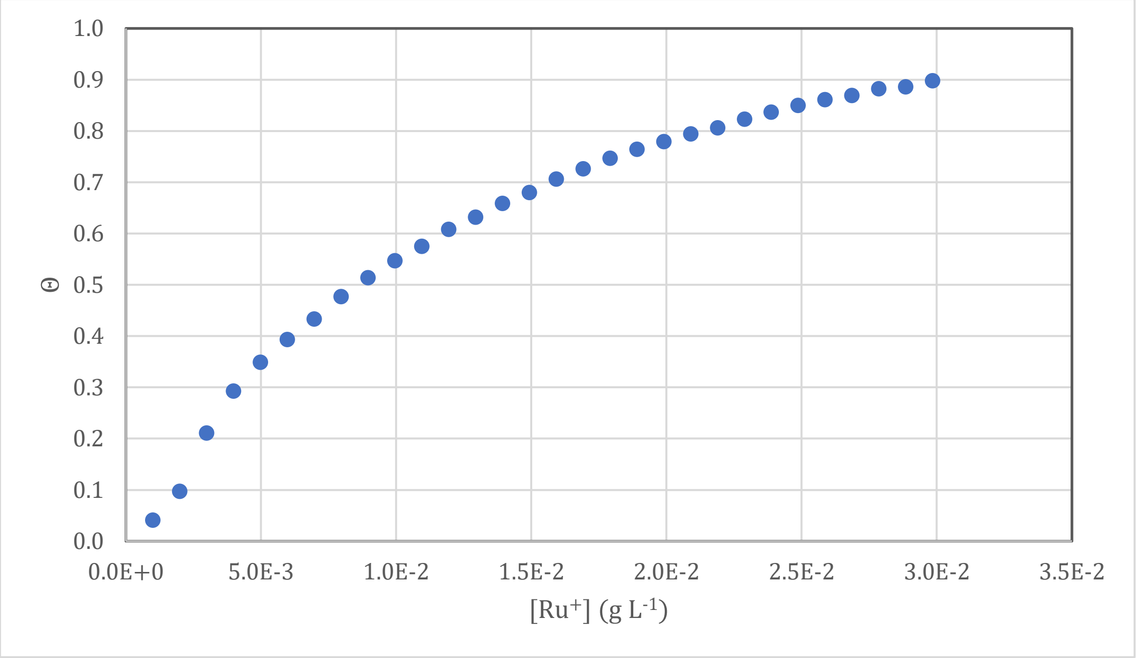

Figure S. 77: Langmuir isotherm for the interaction of the [RuCl(dppb)(bipy)(vpy)]PF_6 complex with $AuNPs^{z-}$ (first measure).

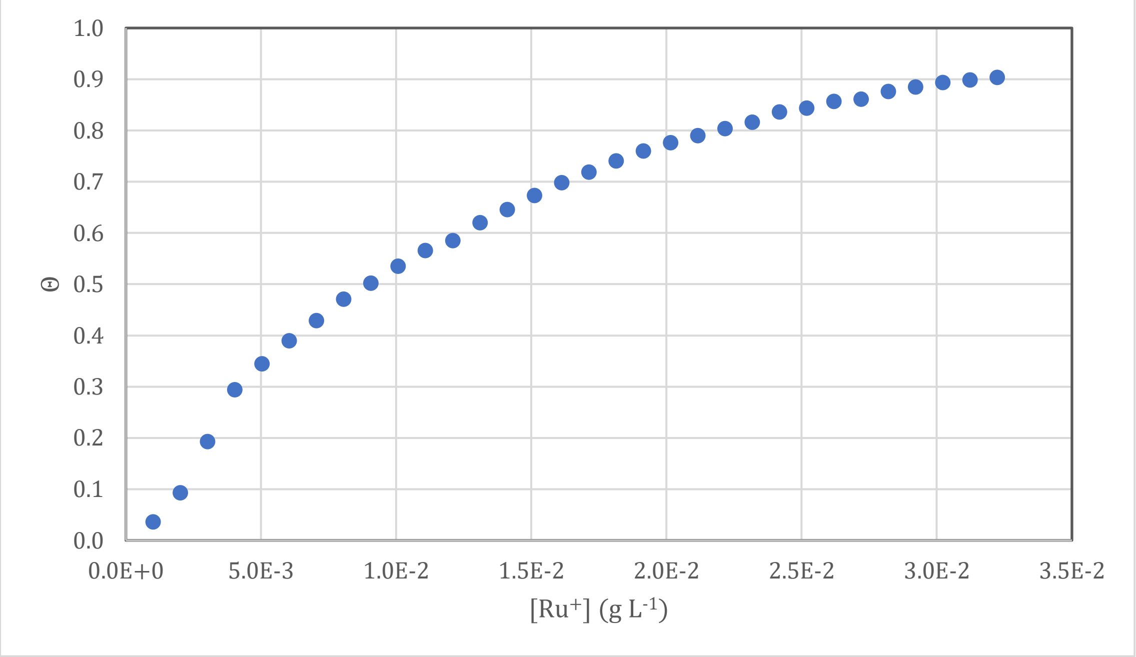

Figure S. 78: Langmuir isotherm for the interaction of the [RuCl(dppb)(bipy)(vpy)]PF_6 complex with $AuNPs^{z-}$ (second measure).

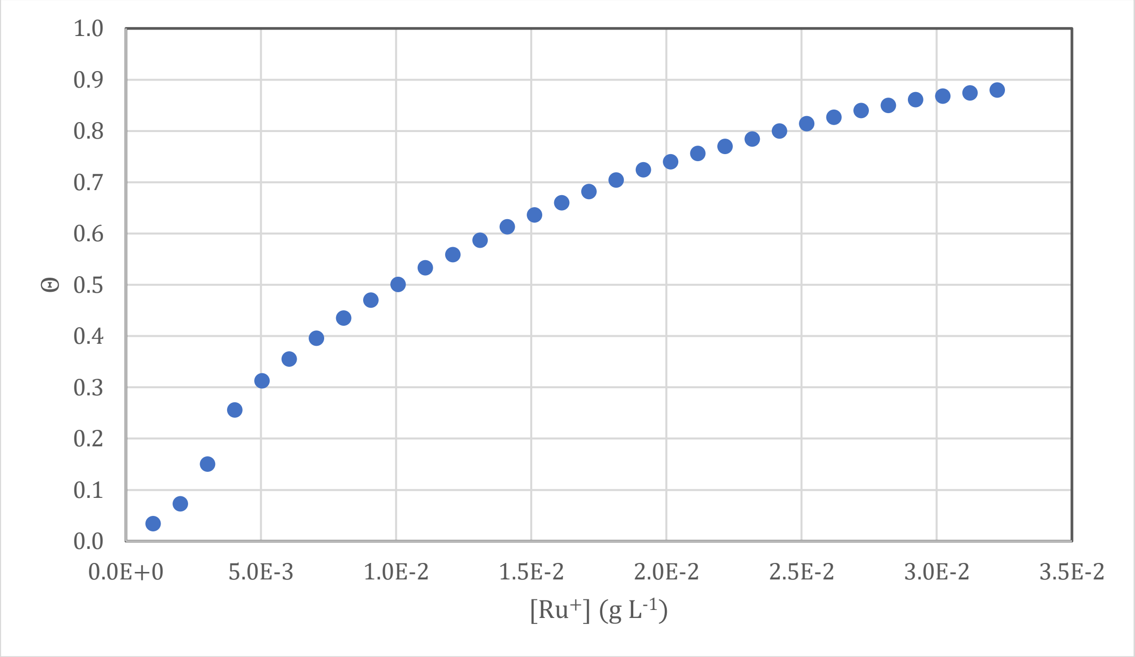

Figure S. 79: Langmuir isotherm for the interaction of the [RuCl(dppb)(bipy)(vpy)]PF_6 complex with $AuNPs^{z-}$ (third measure).

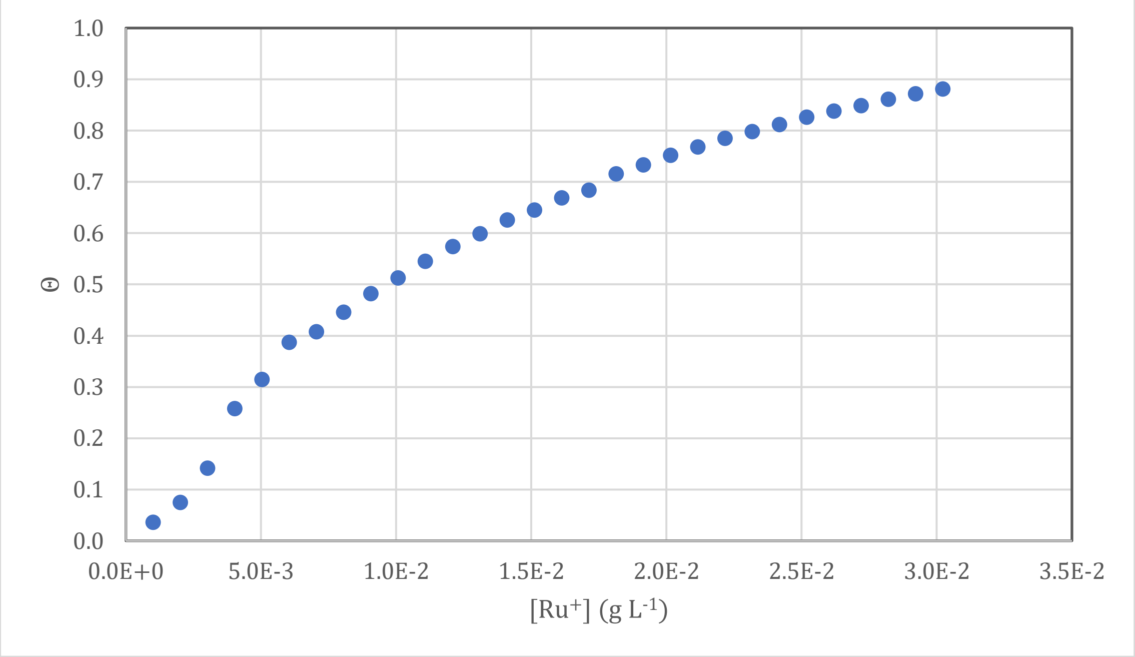

Figure S. 80: Langmuir isotherm for the interaction of the *cis*-$[RuCl_2(dppb)(bipy)]PF_6$ complex with $AuNPs^{z-}$ (first measure).

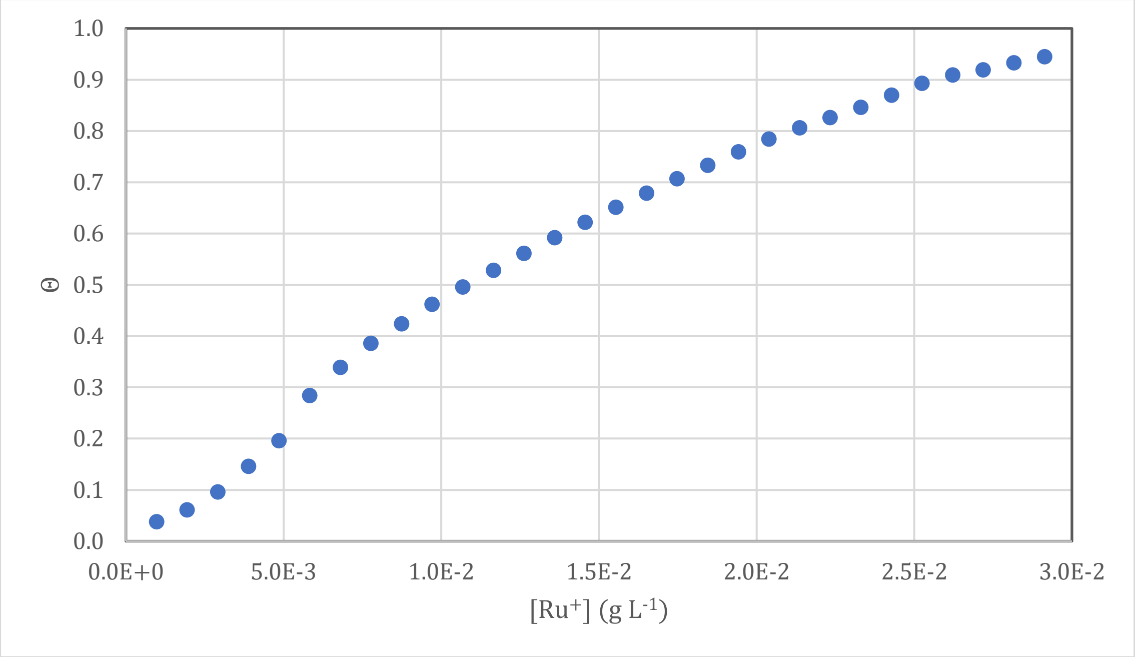

Figure S. 81: Langmuir isotherm for the interaction of the *cis*-$[RuCl_2(dppb)(bipy)]PF_6$ complex with $AuNPs^{z-}$ (second measure).

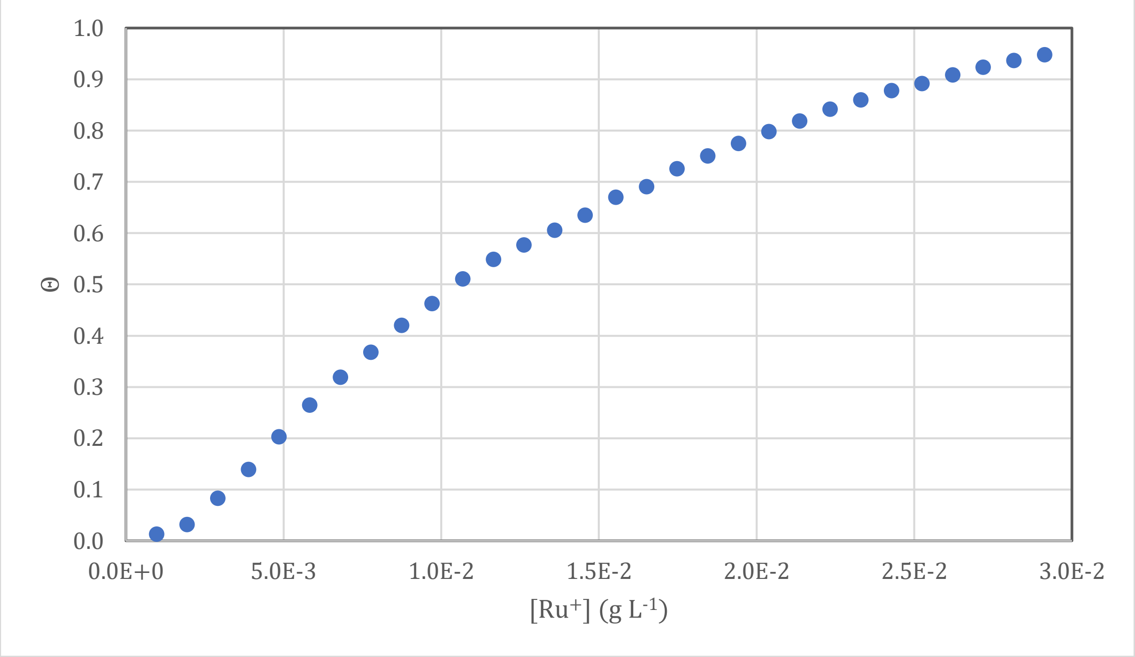

Figure S. 82: Langmuir isotherm for the interaction of the *cis*-[$RuCl_2$(dppb)(bipy)]PF_6 complex with $AuNPs^{z-}$ (third measure).

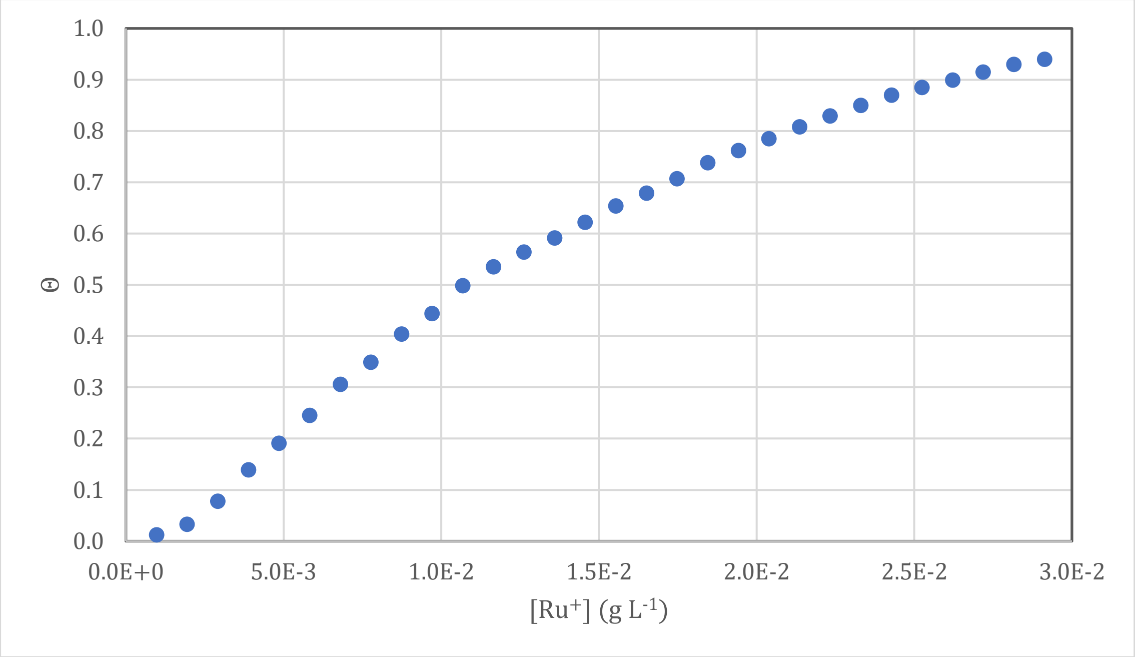

Figure S. 83: Langmuir isotherm for the interaction of the {TPyP[RuCl(dppb)(bipy)]$_4$}(PF_6)$_4$ complex with $AuNPs^{z-}$ (first measure).

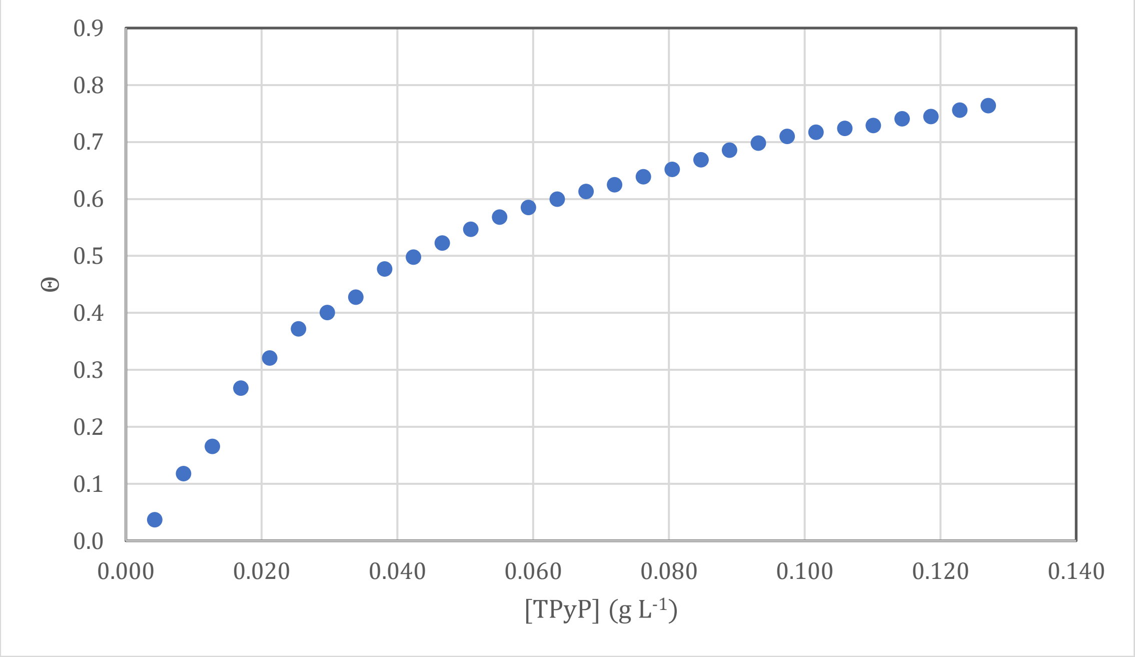

Figure S. 84: Langmuir isotherm for the interaction of the $\{TPyP[RuCl(dppb)(bipy)]_4\}(PF_6)_4$ complex with $AuNPs^{z-}$ (second measure).

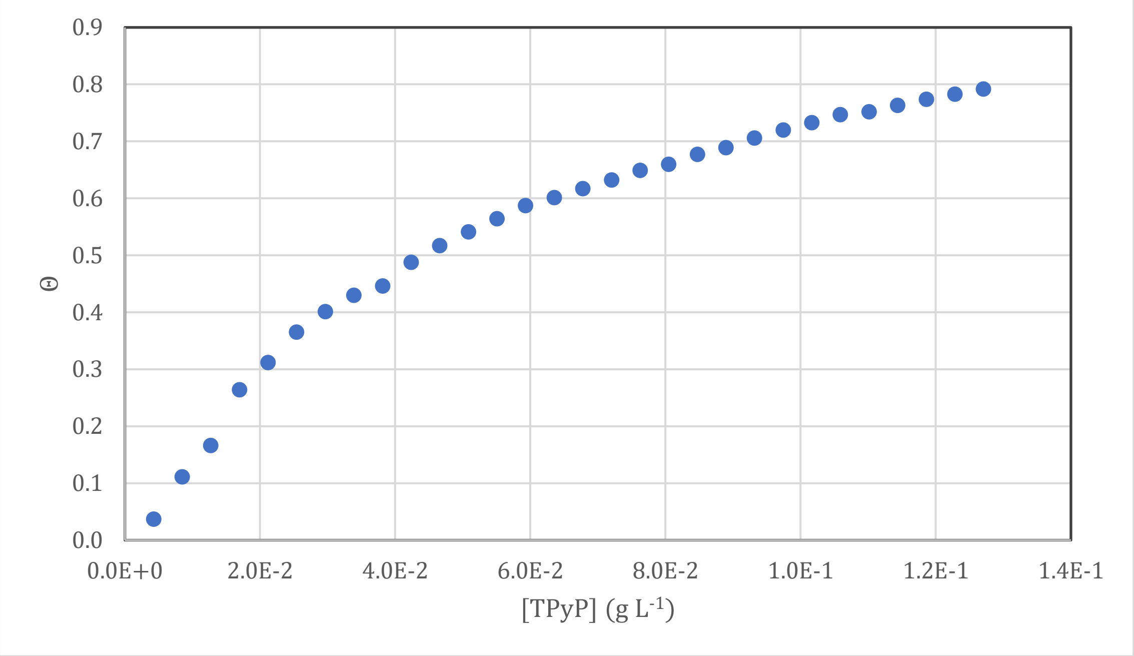

Figure S. 85: Langmuir isotherm for the interaction of the $\{TPyP[RuCl(dppb)(bipy)]_4\}(PF_6)_4$ complex with $AuNPs^{z-}$ (third measure).

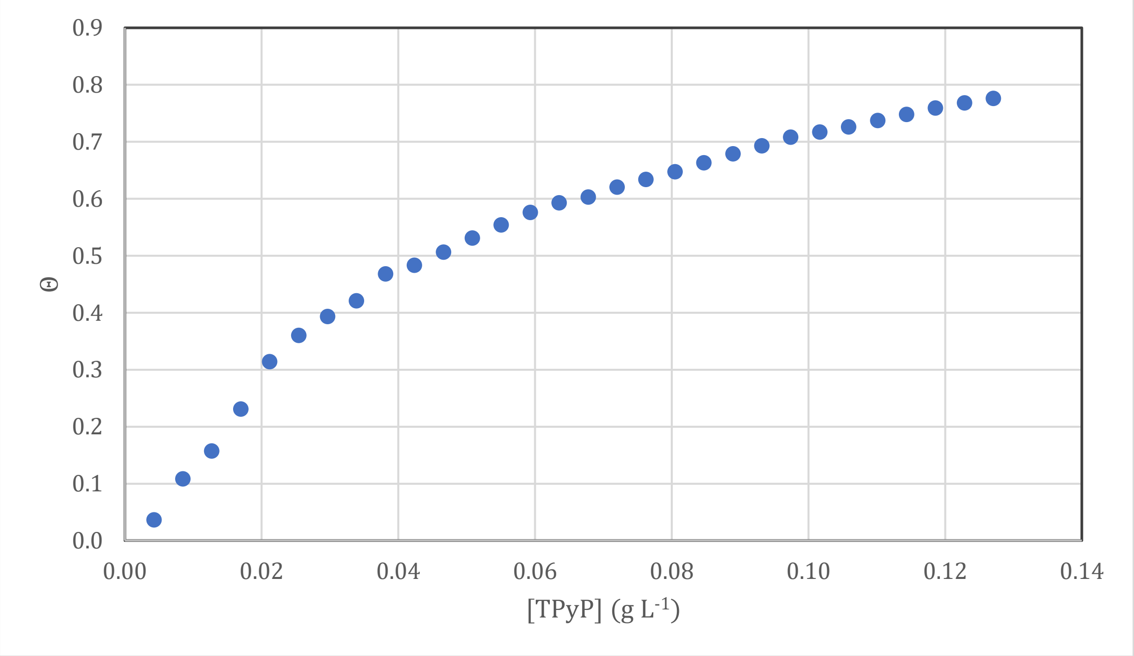

Figure S. 86: Langmuir isotherm for the interaction of the [RuCl(*p*-cymene)(Di*i*pmp)](PF_6) complex with $AuNPs^{z-}$.

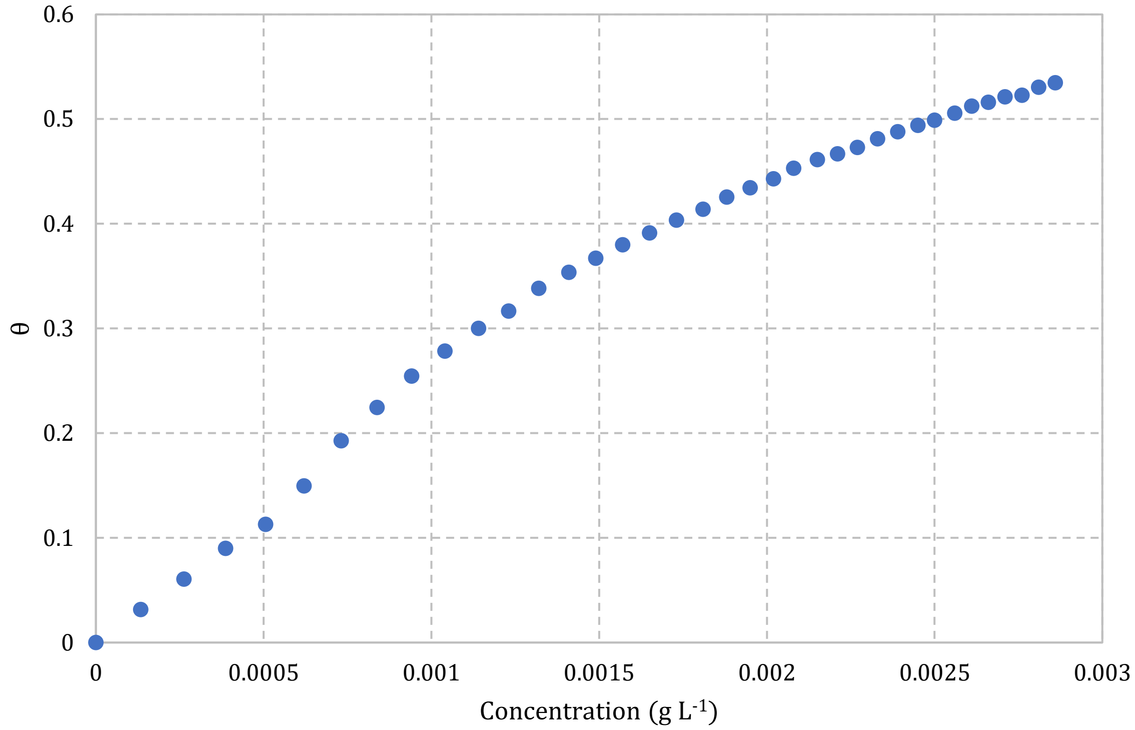

Benesi-Hildebrand isotherm for M^{z+} and $AuNPs^{z-}$ interactions

Figure S. 87: Benesi-Hildebrand isotherm for the interaction of the $[Fe(bipy)_3]Cl_2$ complex with $AuNPs^{z-}$ (first measure).

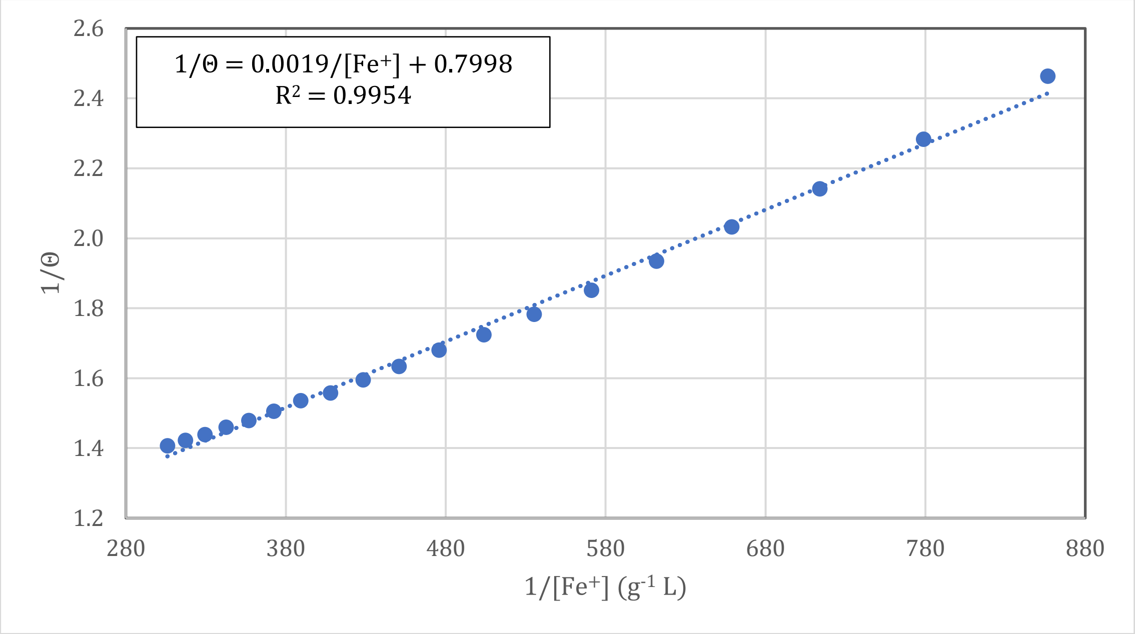

Figure S. 88: Benesi-Hildebrand isotherm for the interaction of the $[Fe(bipy)_3]Cl_2$ complex with $AuNPs^{z-}$ (second measure).

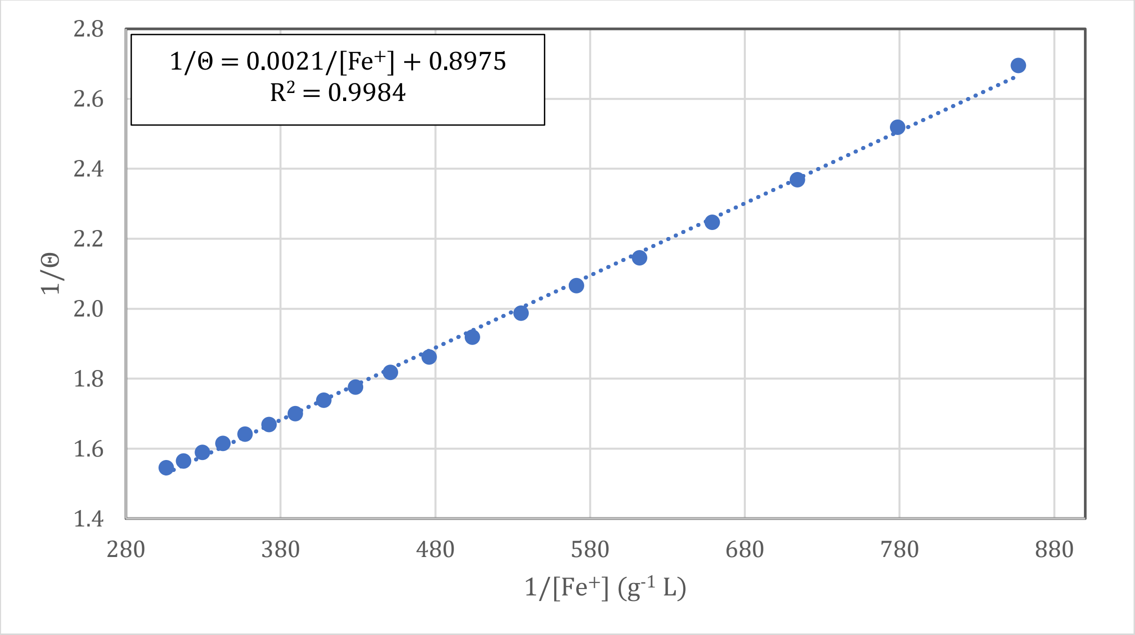

Figure S. 89: Benesi-Hildebrand isotherm for the interaction of the $[Fe(bipy)_3]Cl_2$ complex with $AuNPs^{z-}$ (third measure).

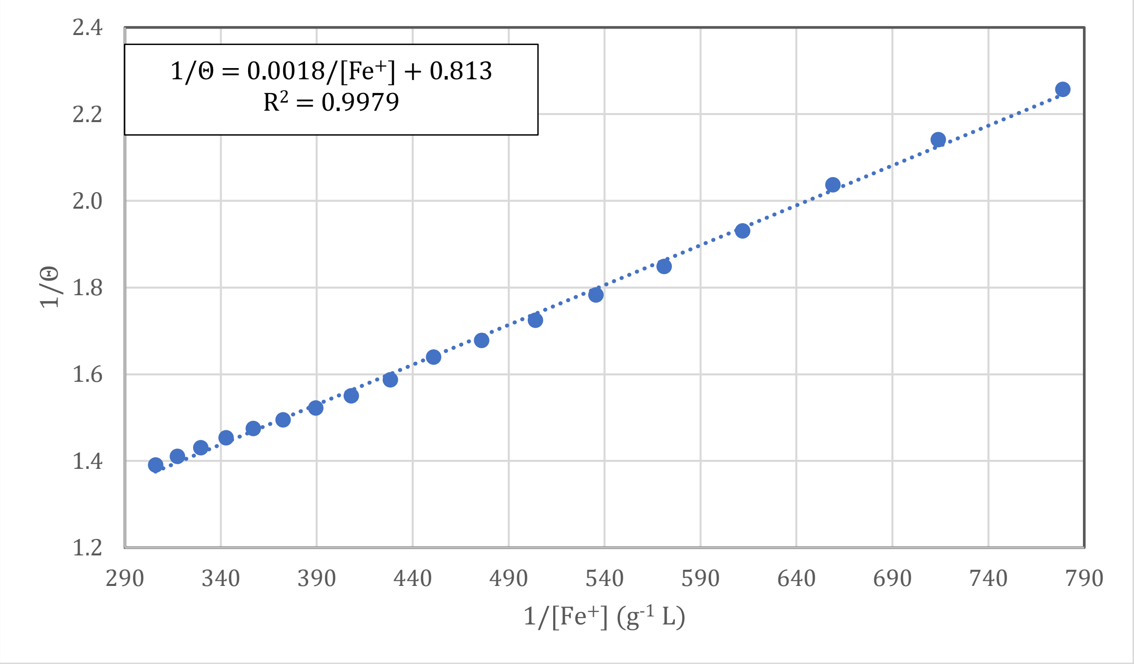

Figure S. 90: Benesi-Hildebrand isotherm for the interaction of the $[Ru(bipy)_3](PF_6)_2$ complex with $AuNPs^{z-}$ (first measure).

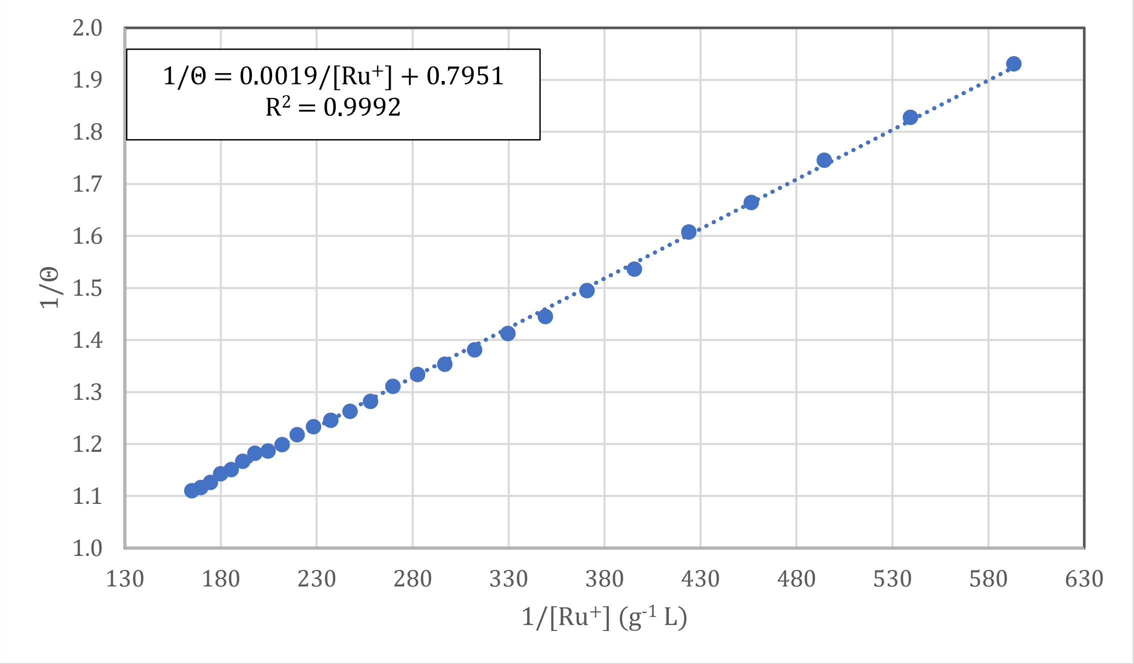

Figure S. 91: Benesi-Hildebrand isotherm for the interaction of the $[Ru(bipy)_3](PF_6)_2$ complex with $AuNPs^{z-}$ (second measure).

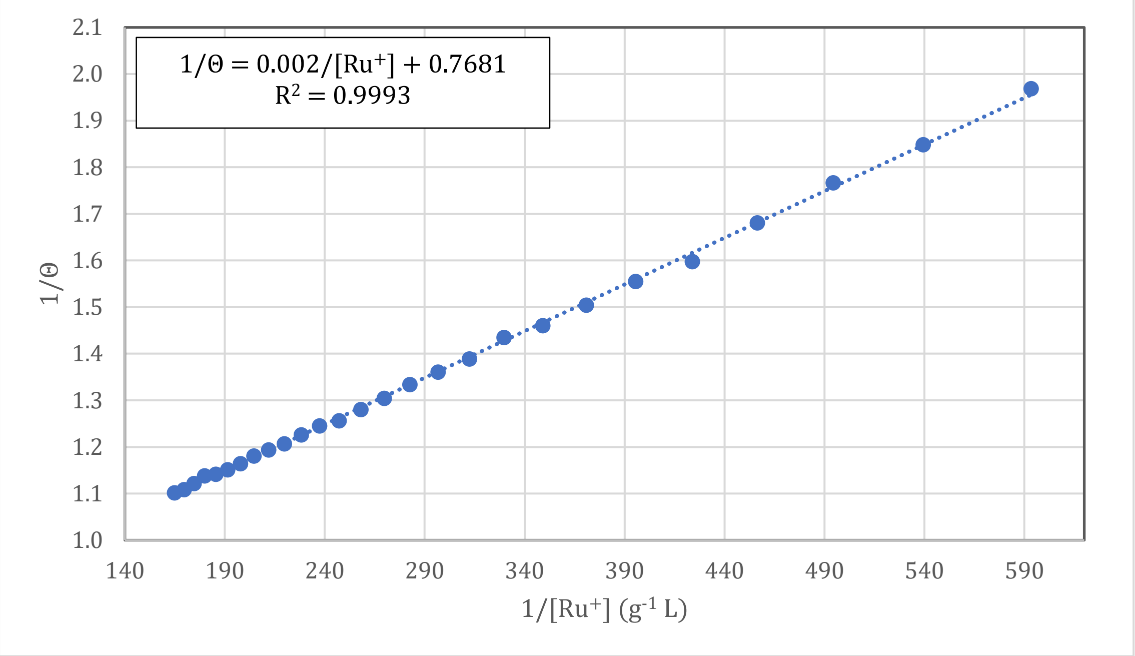

Figure S. 92: Benesi-Hildebrand isotherm for the interaction of the $[Ru(bipy)_3](PF_6)_2$ complex with $AuNPs^{z-}$ (third measure).

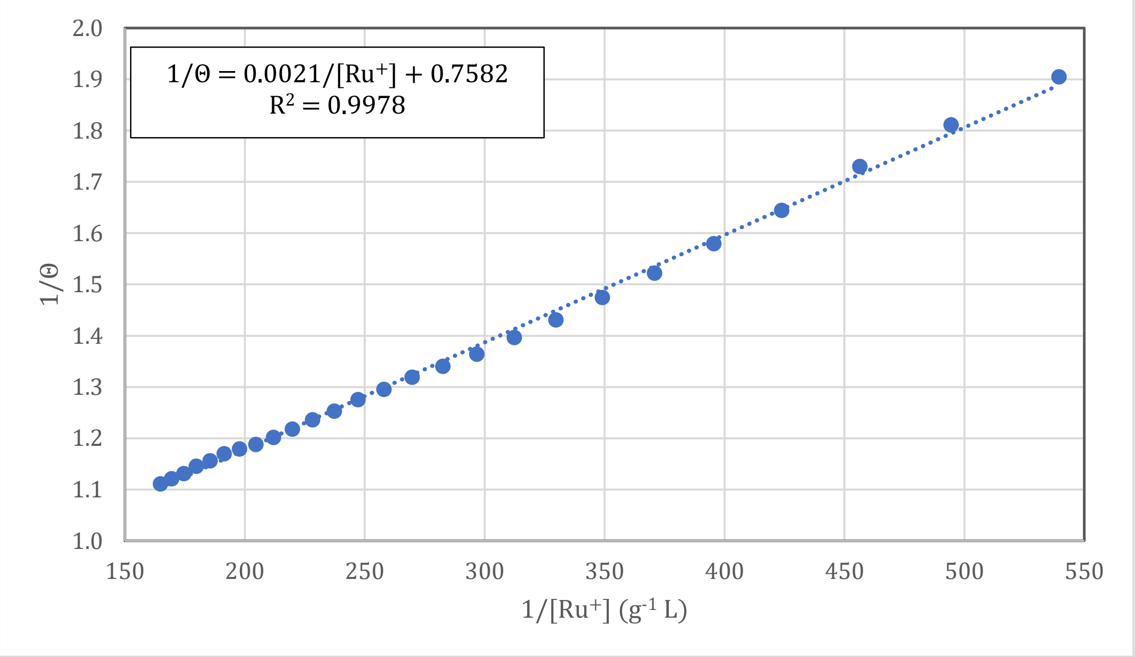

Figure S. 93: Benesi-Hildebrand isotherm for the interaction of the $[RuCl(dppb)(bipy)(py)]PF_6$ complex with $AuNPs^{z-}$ (first measure).

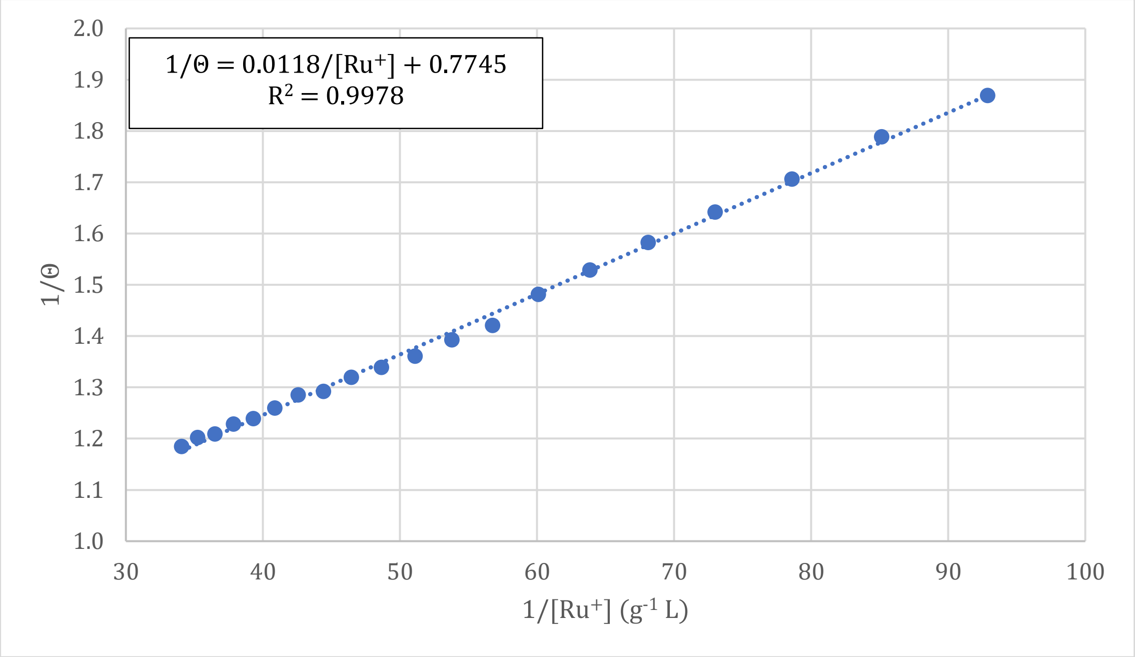

Figure S. 94: Benesi-Hildebrand isotherm for the interaction of the $[RuCl(dppb)(bipy)(py)]PF_6$ complex with $AuNPs^{z-}$ (second measure).

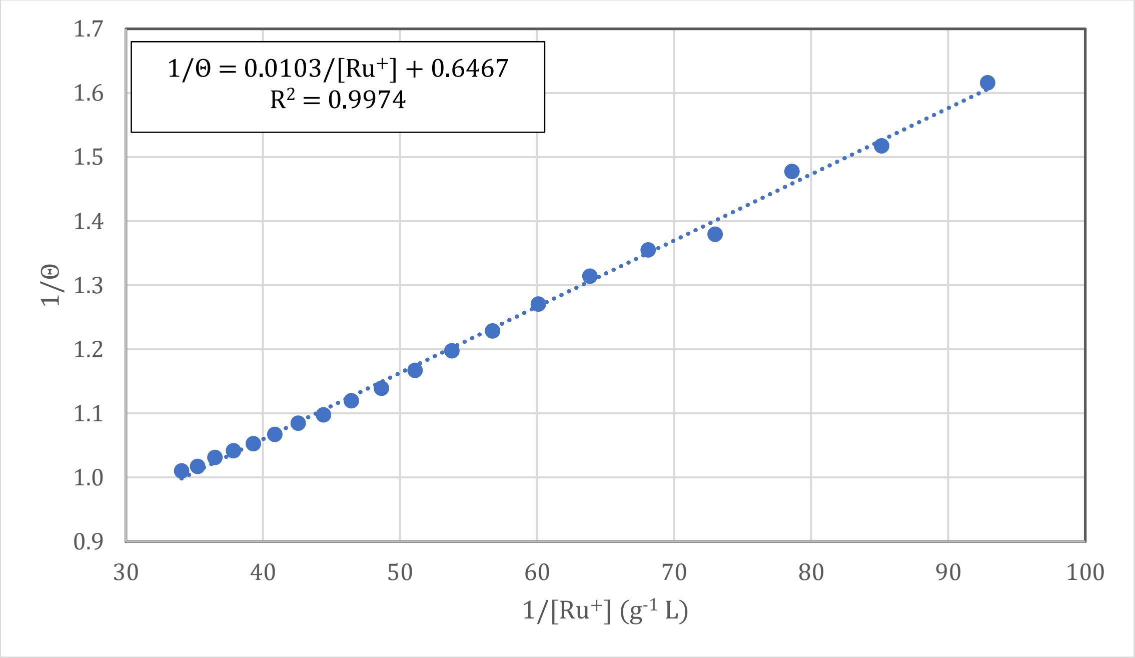

Figure S. 95: Benesi-Hildebrand isotherm for the interaction of the $[RuCl(dppb)(bipy)(py)]PF_6$ complex with $AuNPs^{z-}$ (third measure).

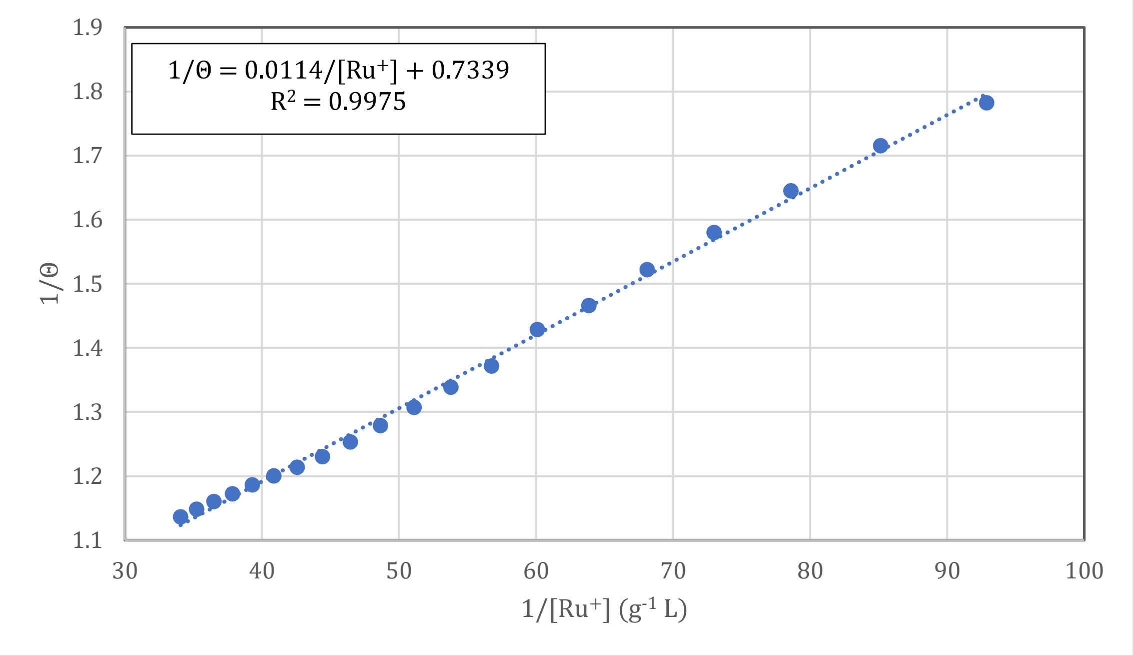

Figure S. 96: Benesi-Hildebrand isotherm for the interaction of the $[RuCl(dppb)(bipy)(tbpy)]PF_6$ complex with $AuNPs^{z-}$ (first measure).

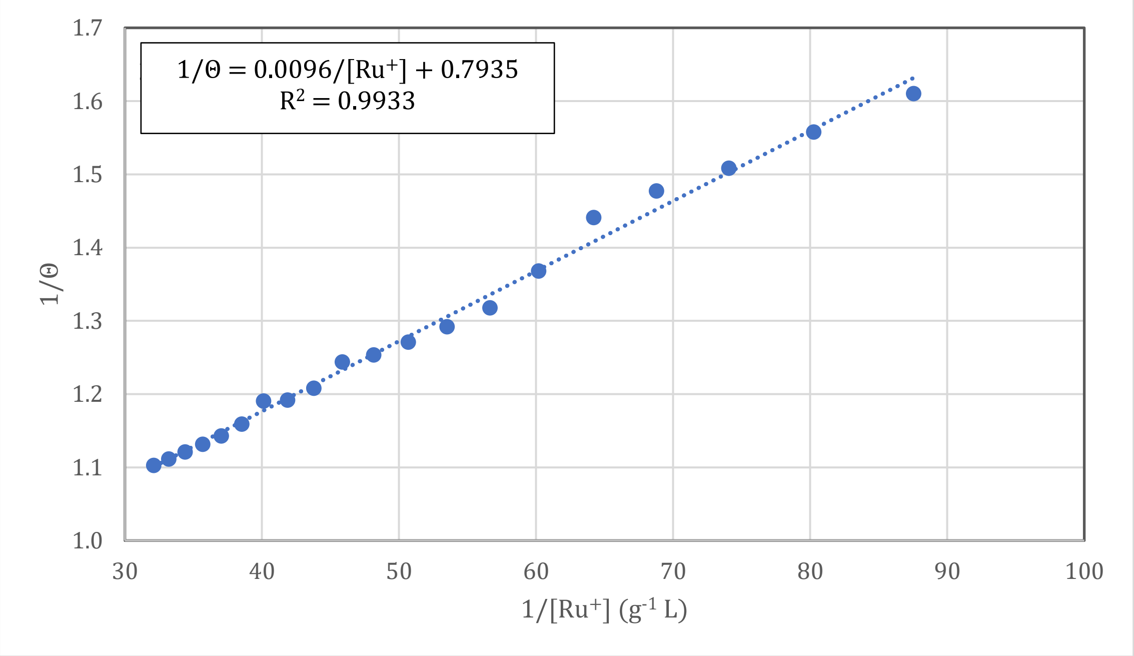

Figure S. 97: Benesi-Hildebrand isotherm for the interaction of the [RuCl(dppb)(bipy)(*t*bpy)]PF_6 complex with $AuNPs^{z-}$ (second measure).

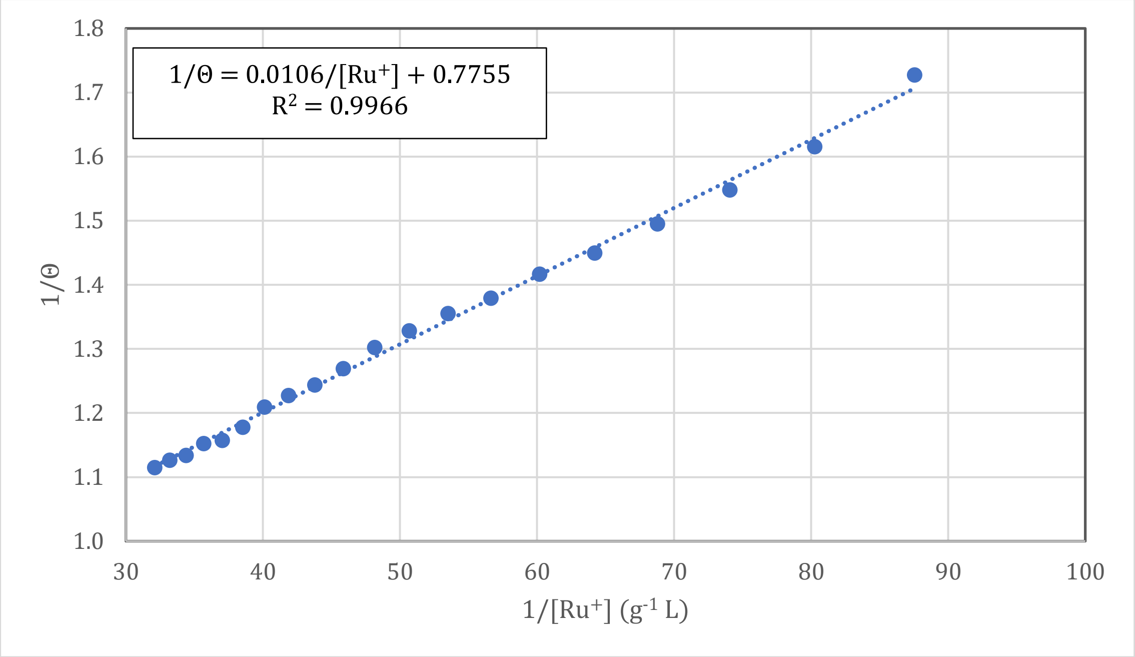

Figure S. 98: Benesi-Hildebrand isotherm for the interaction of the [RuCl(dppb)(bipy)(*t*bpy)]PF_6 complex with $AuNPs^{z-}$ (third measure).

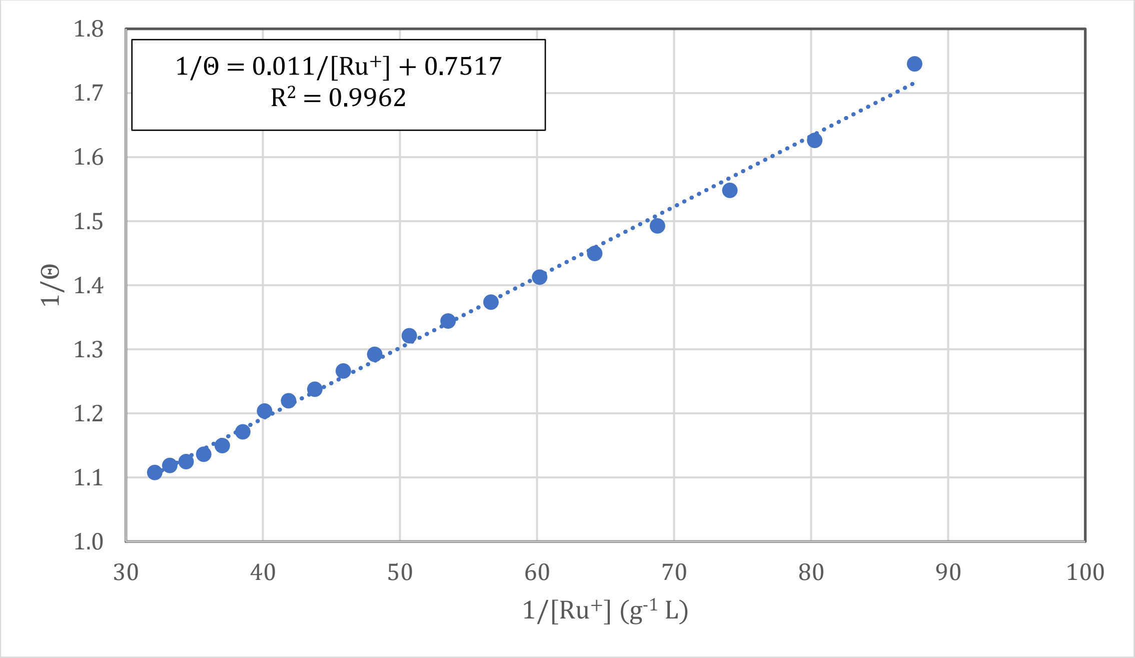

Figure S. 99: Benesi-Hildebrand isotherm for the interaction of the [RuCl(dppb)(bipy)(mepy)]PF_6 complex with $AuNPs^{z-}$ (first measure).

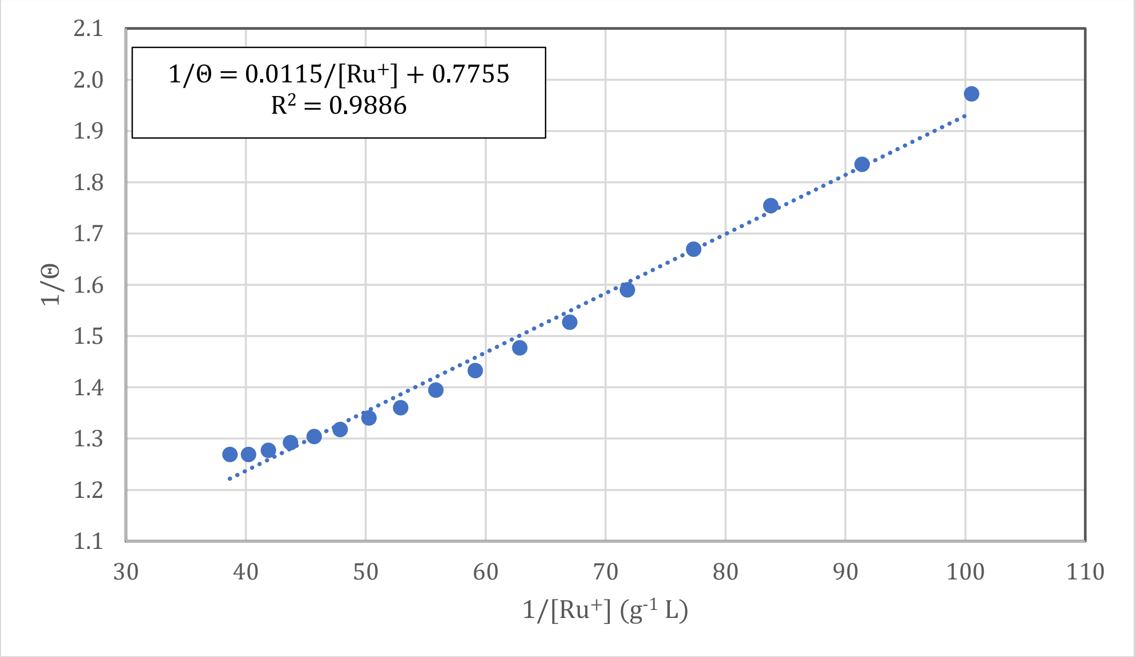

Figure S. 100: Benesi-Hildebrand isotherm for the interaction of the [RuCl(dppb)(bipy)(mepy)]PF_6 complex with $AuNPs^{z-}$ (second measure).

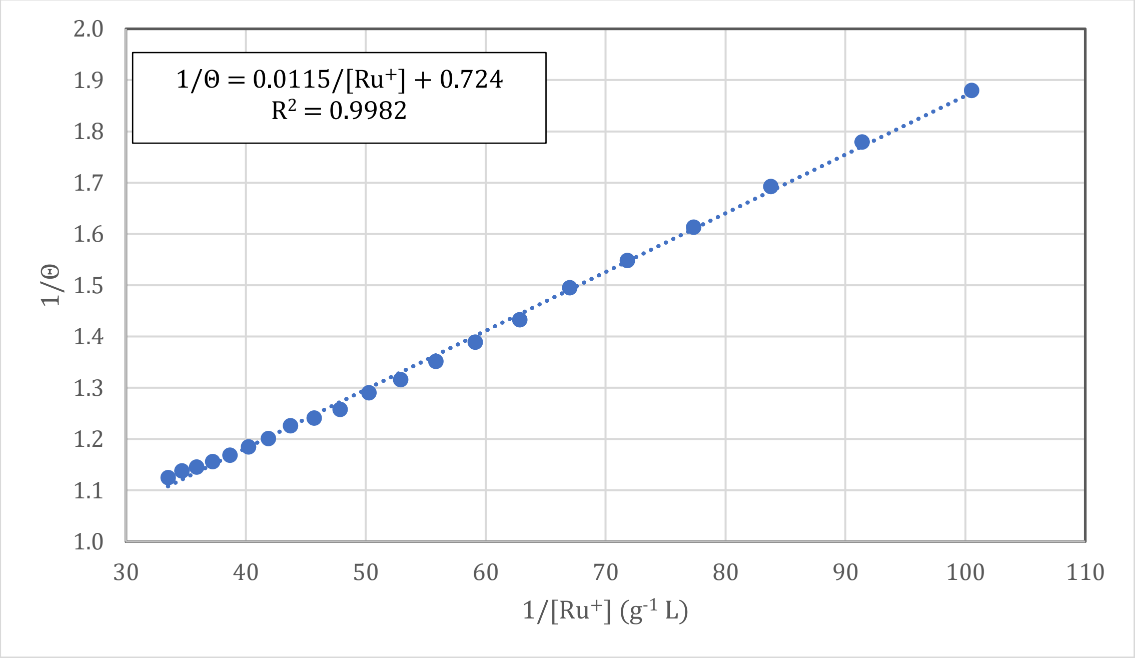

Figure S. 101: Benesi-Hildebrand isotherm for the interaction of the [RuCl(dppb)(bipy)(mepy)]PF_6 complex with $AuNPs^{z-}$ (first measure).

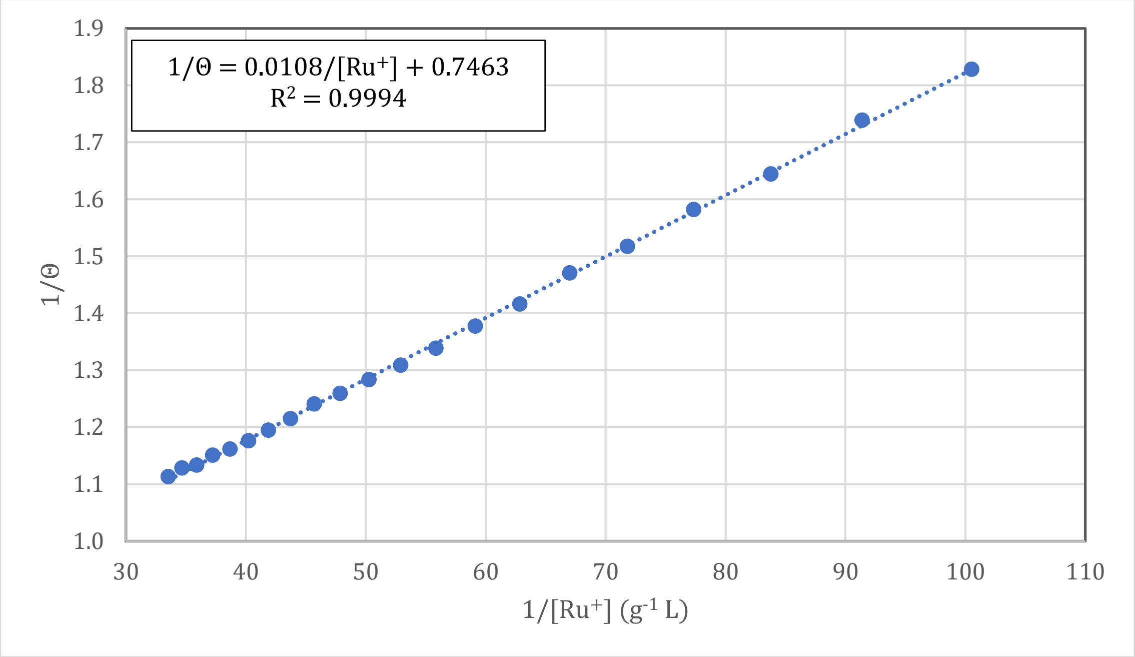

Figure S. 102: Benesi-Hildebrand isotherm for the interaction of the [RuCl(dppb)(bipy)(vpy)]PF_6 complex with $AuNPs^{z-}$ (first measure).

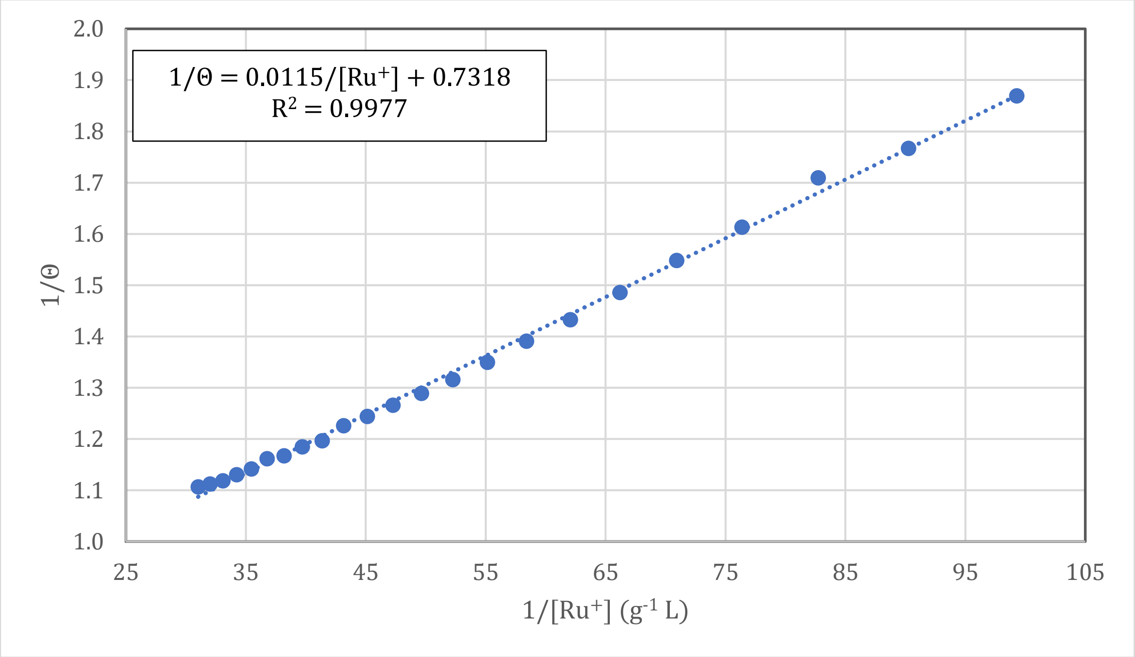

Figure S. 103: Benesi-Hildebrand isotherm for the interaction of the [RuCl(dppb)(bipy)(vpy)]PF_6 complex with $AuNPs^{z-}$ (second measure).

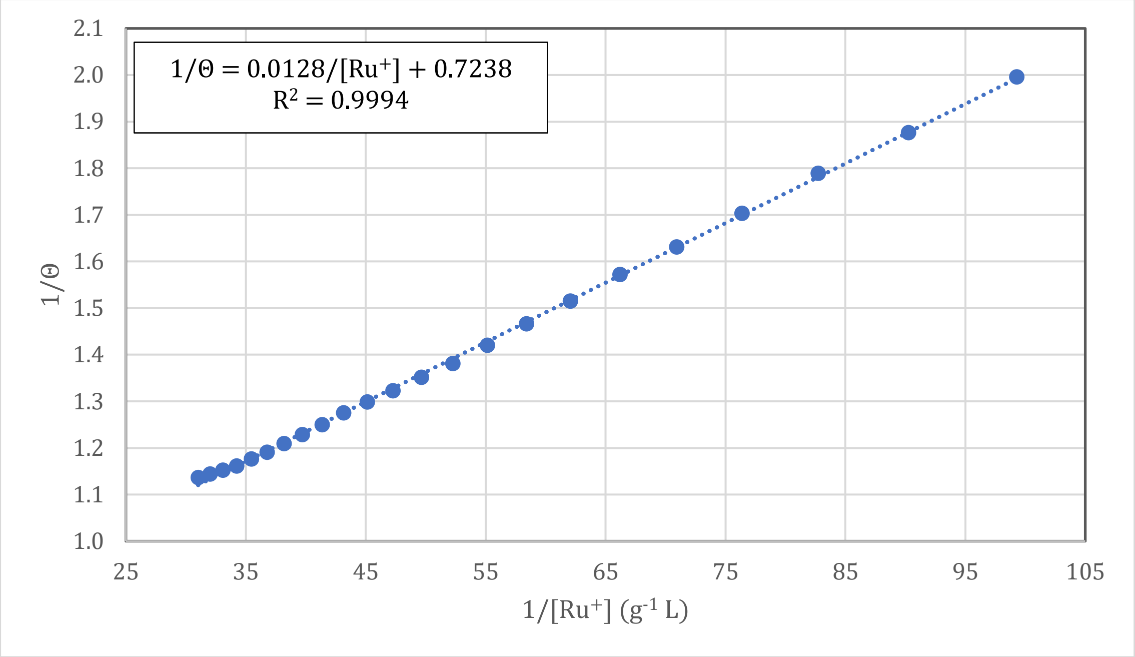

Figure S. 104: Benesi-Hildebrand isotherm for the interaction of the [RuCl(dppb)(bipy)(vpy)]PF_6 complex with $AuNPs^{z-}$ (third measure).

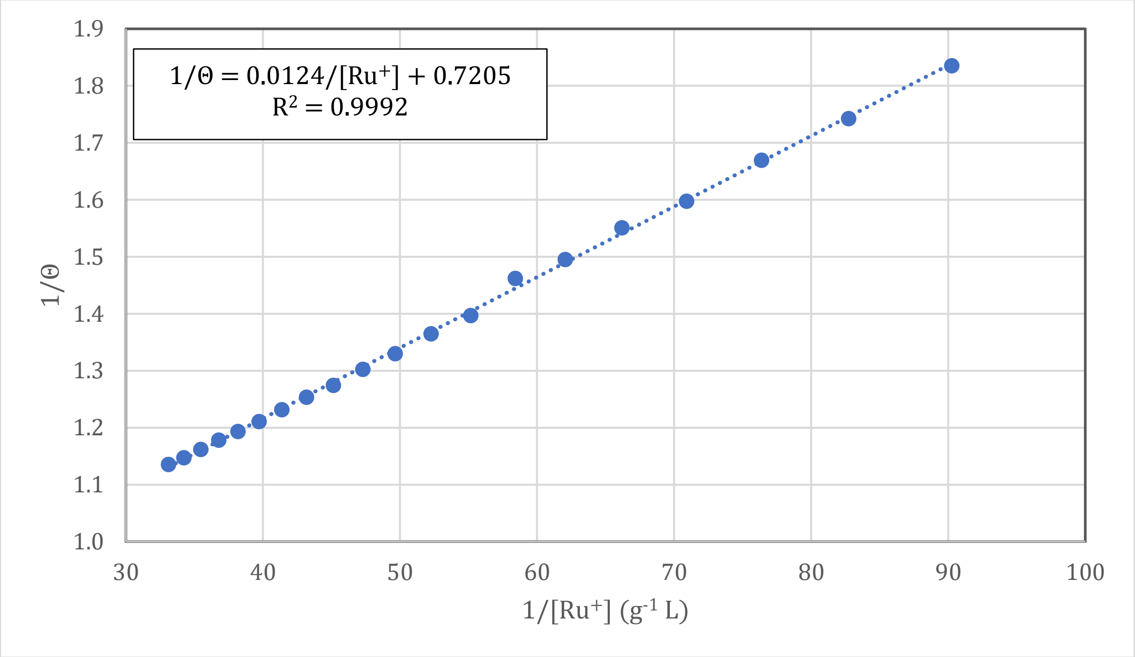

Figure S. 105: Benesi-Hildebrand isotherm for the interaction of the *cis*-$[RuCl_2(dppb)(bipy)]PF_6$ complex with $AuNPs^{z-}$ (first measure).

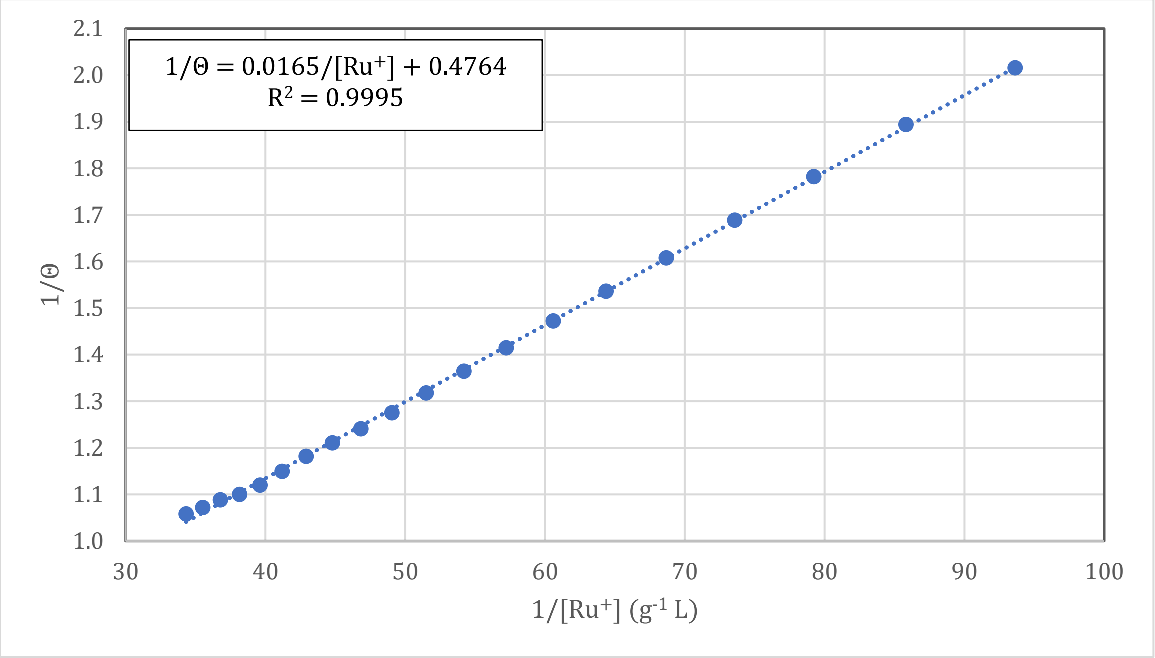

Figure S. 106: Benesi-Hildebrand isotherm for the interaction of the *cis*-$[RuCl_2(dppb)(bipy)]PF_6$ complex with $AuNPs^{z-}$ (second measure).

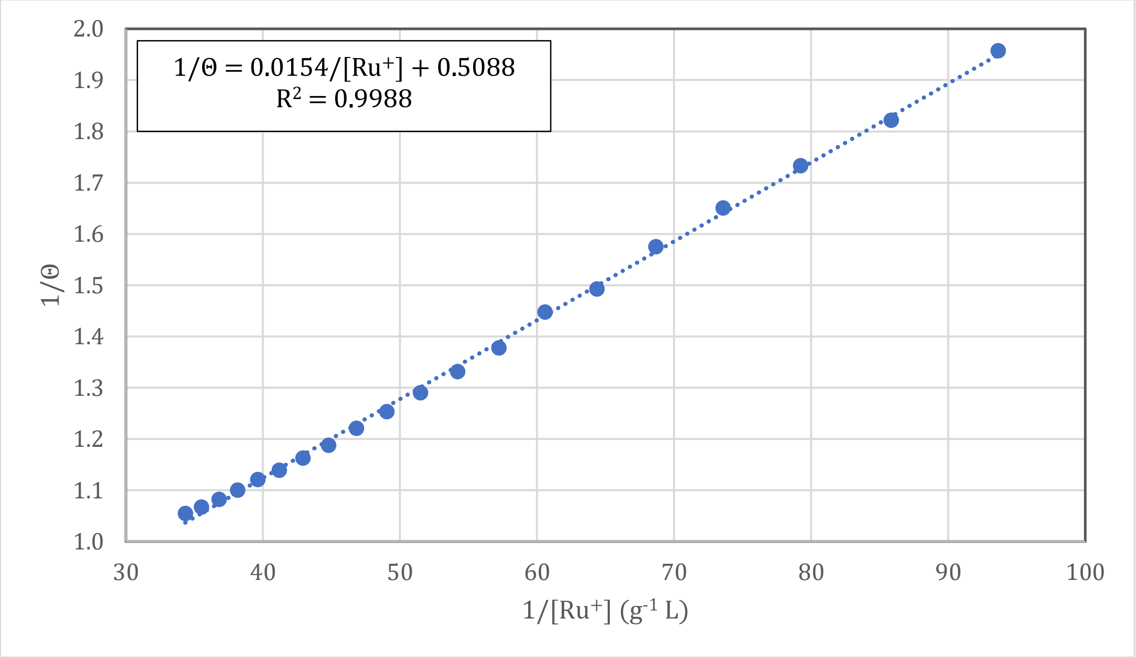

Figure S. 107: Benesi-Hildebrand isotherm for the interaction of the *cis*-$[RuCl_2(dppb)(bipy)]PF_6$ complex with $AuNPs^{z-}$ (third measure).

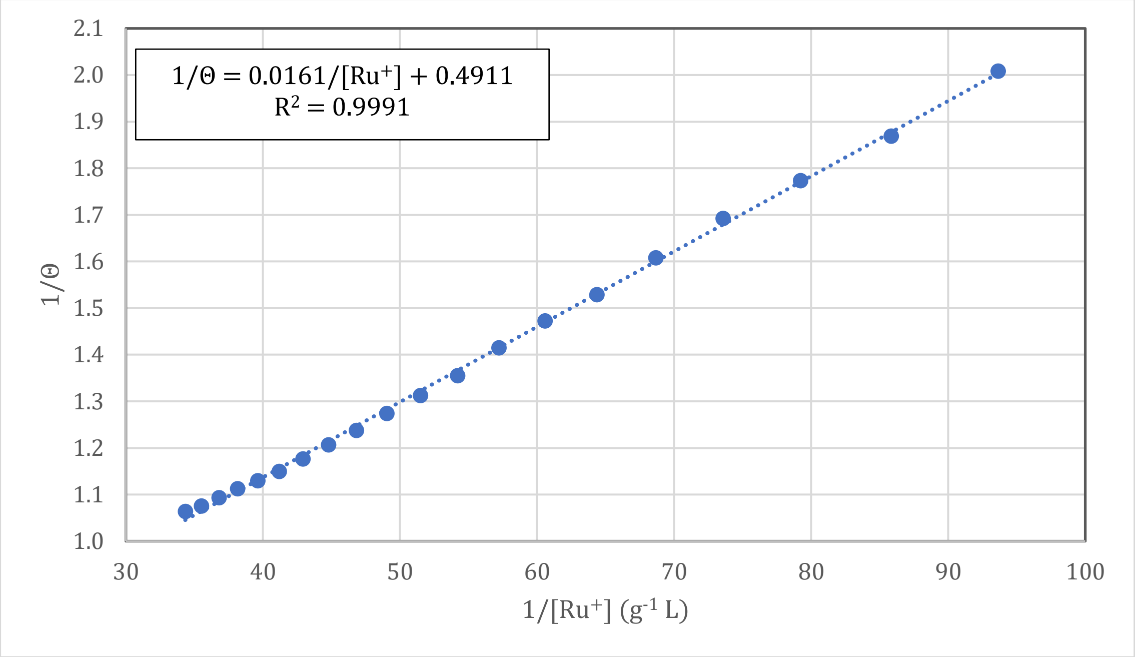

Figure S. 108: Benesi-Hildebrand isotherm for the interaction of the $\{TPyP[RuCl(dppb)(bipy)]_4\}(PF_6)_4$ complex with $AuNPs^{z-}$ (first measure).

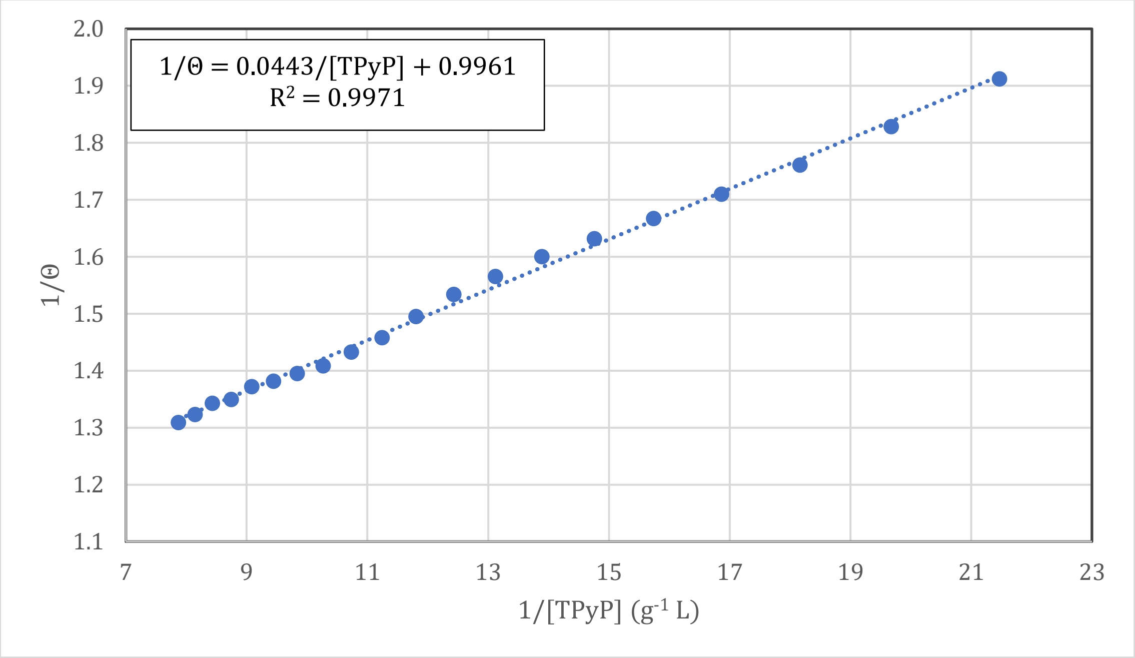

Figure S. 109: Benesi-Hildebrand isotherm for the interaction of the $\{TPyP[RuCl(dppb)(bipy)]_4\}(PF_6)_4$ complex with $AuNPs^{z-}$ (second measure).

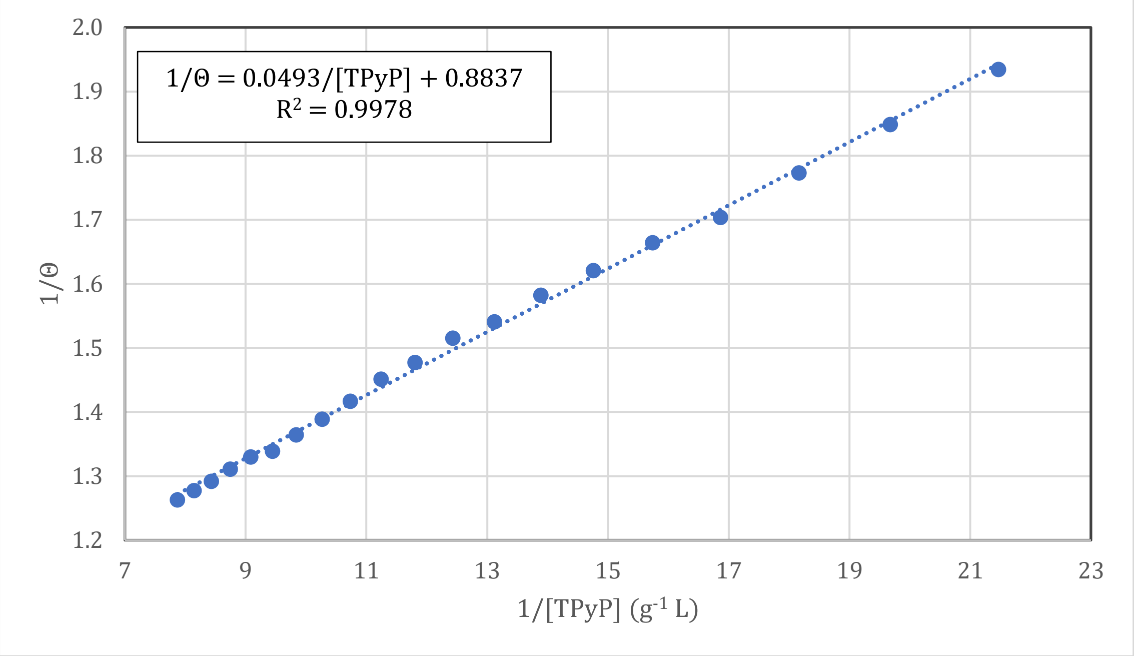

Figure S. 110: Benesi-Hildebrand isotherm for the interaction of the $\{TPyP[RuCl(dppb)(bipy)]_4\}(PF_6)_4$ complex with $AuNPs^{z-}$ (third measure).

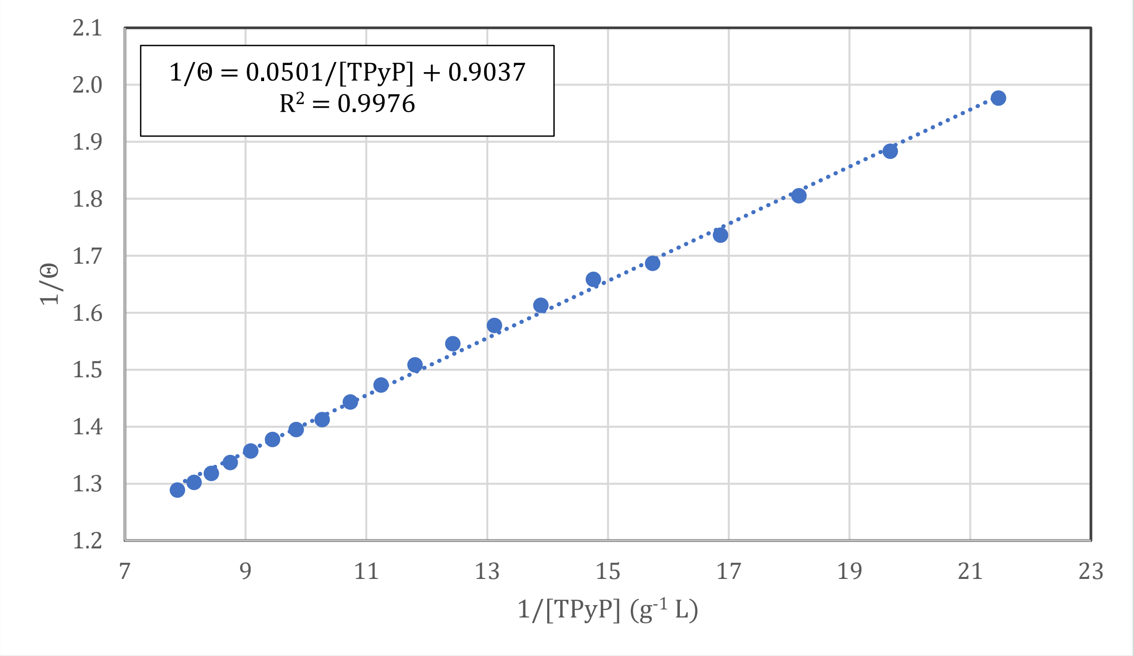

Figure S. 111: Benesi-Hildebrand isotherm for the interaction of the RuCl(*p*-cymene)(Diipmp)](PF_6) complex with $AuNPs^{z-}$ (first measure).

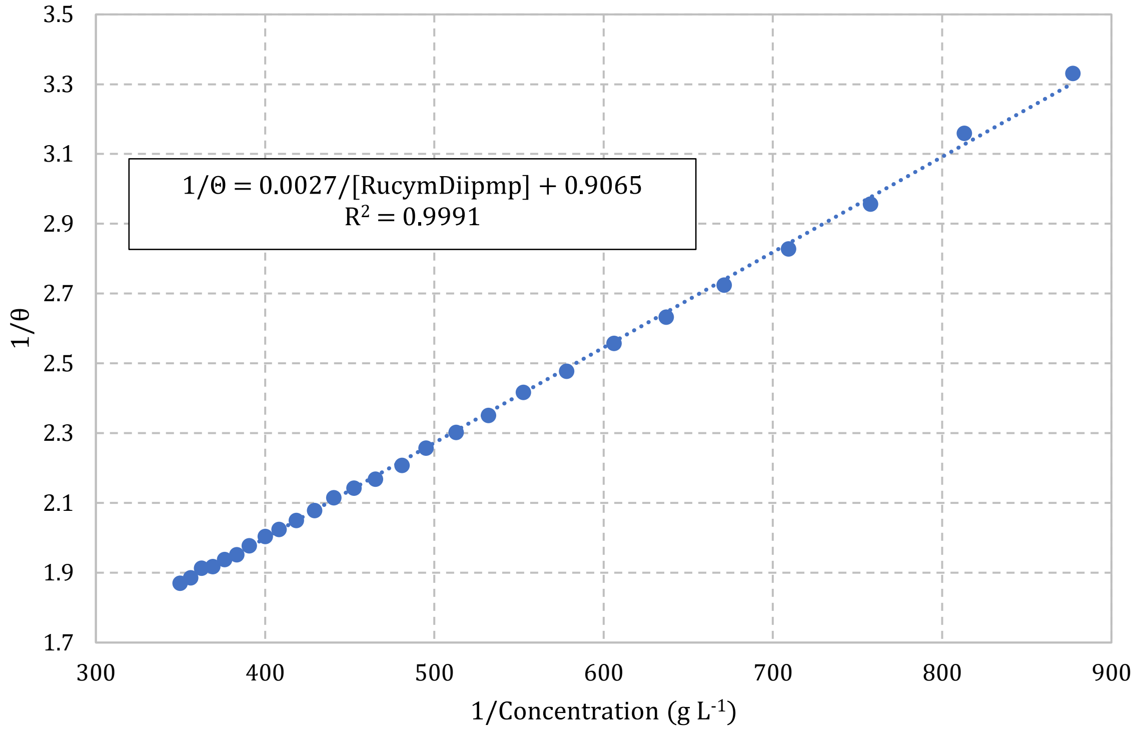

Scatchard isotherm for M^{z+} and $AuNPs^{z-}$ interactions

Figure S. 112: Scatchard isotherm for the interaction of the $[Fe(bipy)_3]Cl_2$ complex with $AuNPs^{z-}$ (first measure).

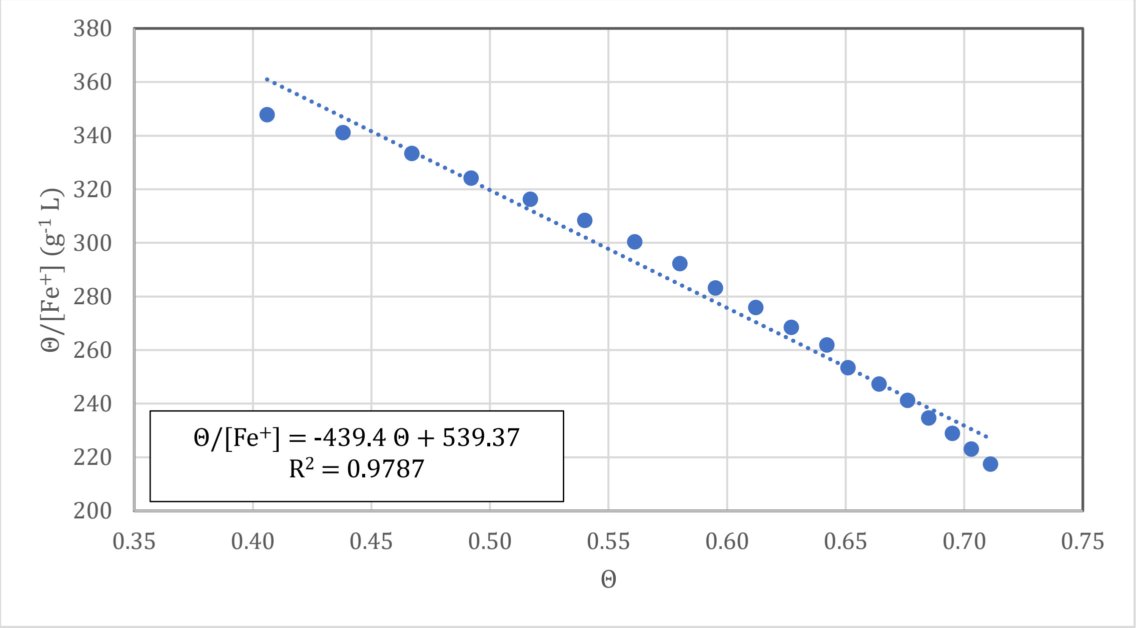

Figure S. 113: Scatchard isotherm for the interaction of the $[Fe(bipy)_3]Cl_2$ complex with $AuNPs^{z-}$ (second measure).

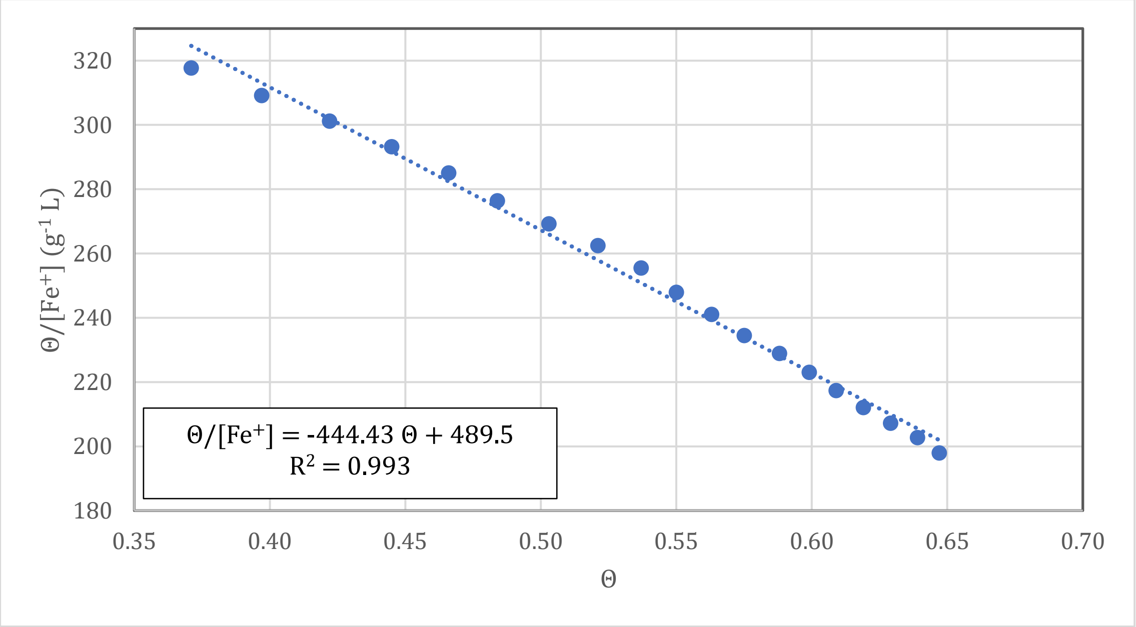

Figure S. 114: Scatchard isotherm for the interaction of the $[Fe(bipy)_3]Cl_2$ complex with $AuNPs^{z-}$ (third measure).

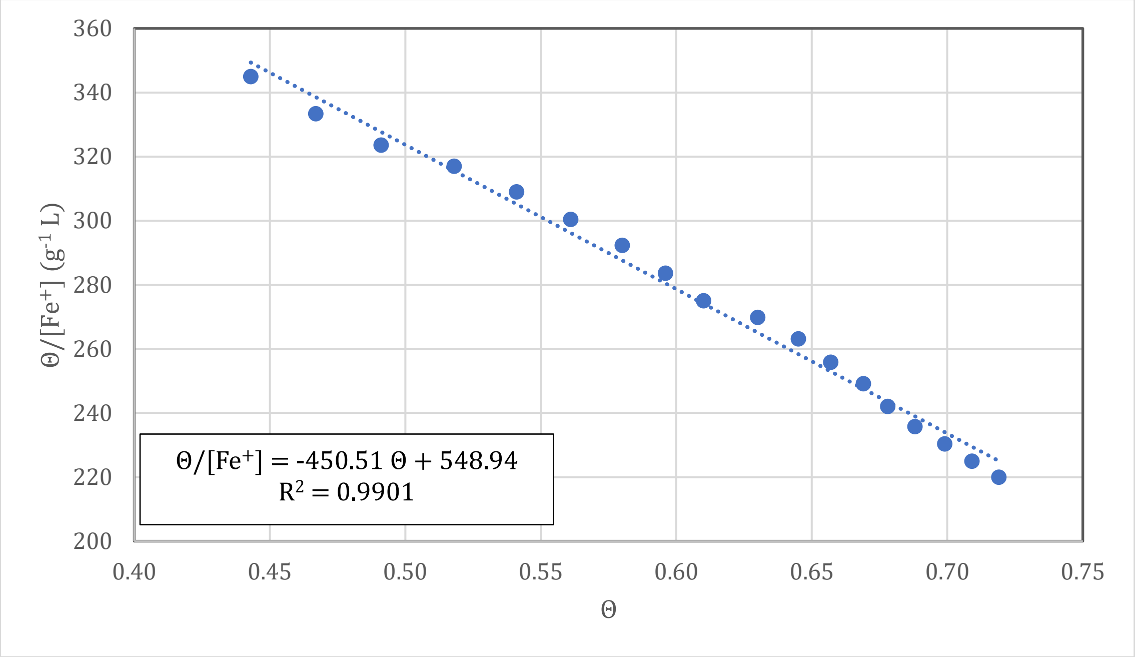

Figure S. 115: Scatchard isotherm for the interaction of the $[Ru(bipy)_3](PF_6)_2$ complex with $AuNPs^{z-}$ (first measure).

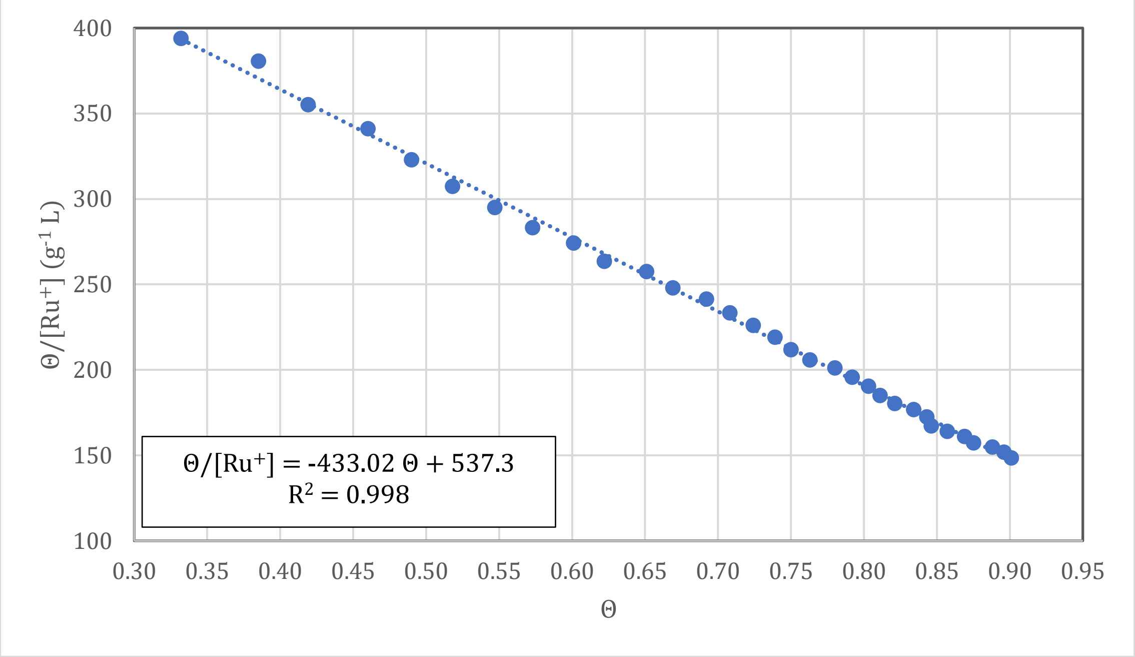

Figure S. 116: Scatchard isotherm for the interaction of the $[Ru(bipy)_3](PF_6)_2$ complex with $AuNPs^{z-}$ (second measure).

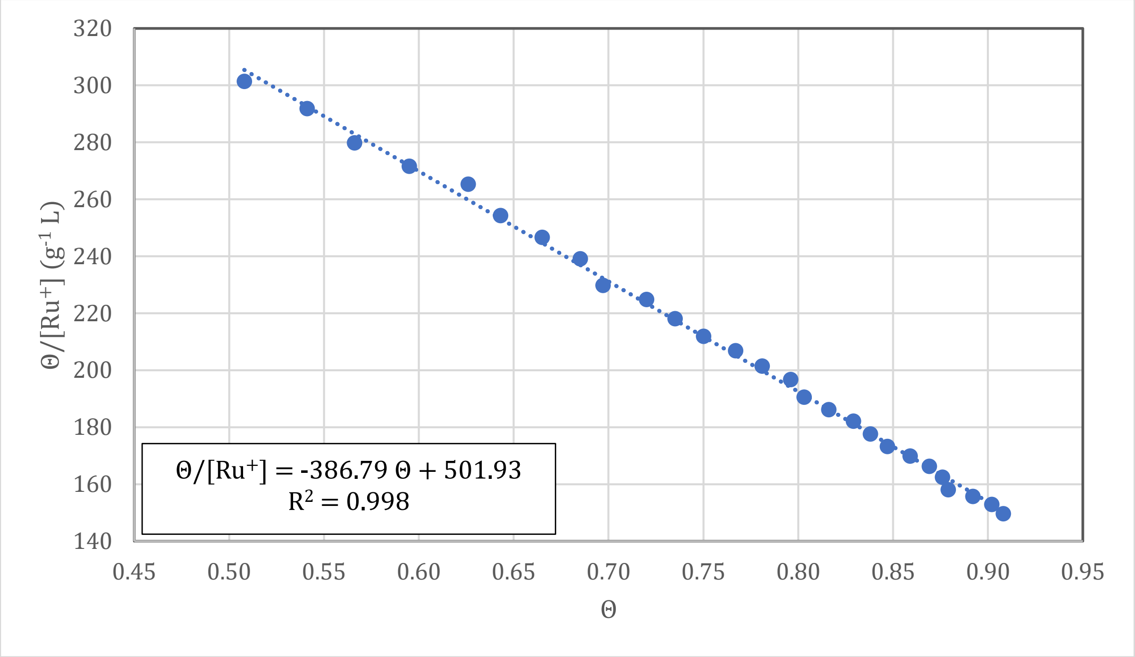

Figure S. 117: Scatchard isotherm for the interaction of the $[Ru(bipy)_3](PF_6)_2$ complex with $AuNPs^{z-}$ (third measure).

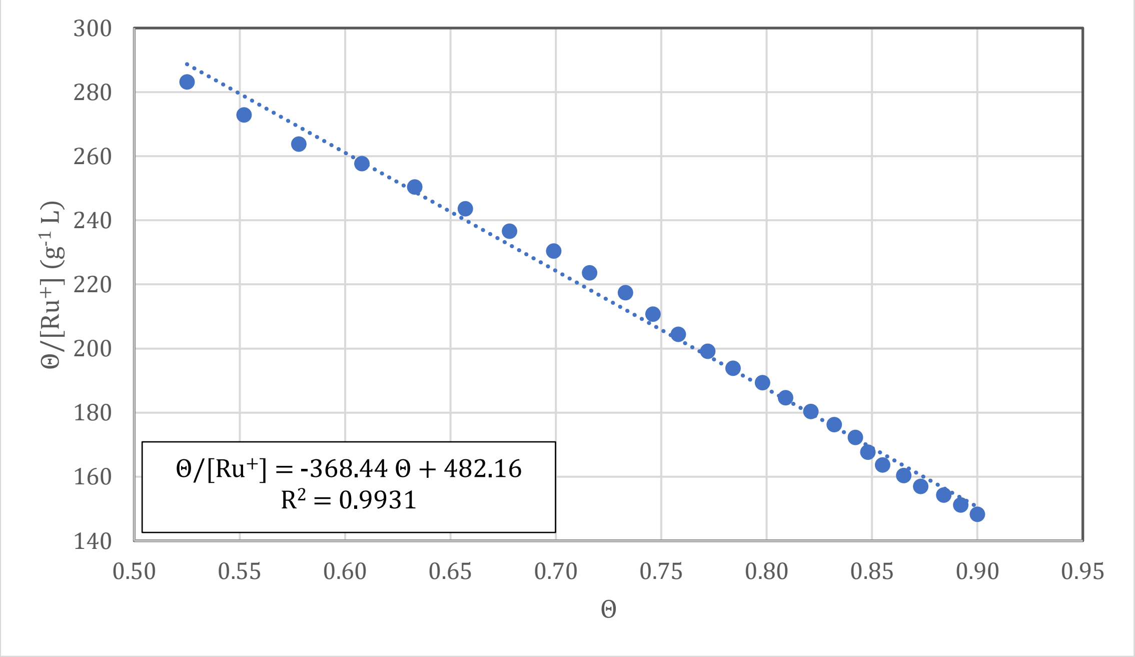

Figure S. 118: Scatchard isotherm for the interaction of the [RuCl(dppb)(bipy)(py)]PF_6 complex with $AuNPs^{z-}$ (first measure).

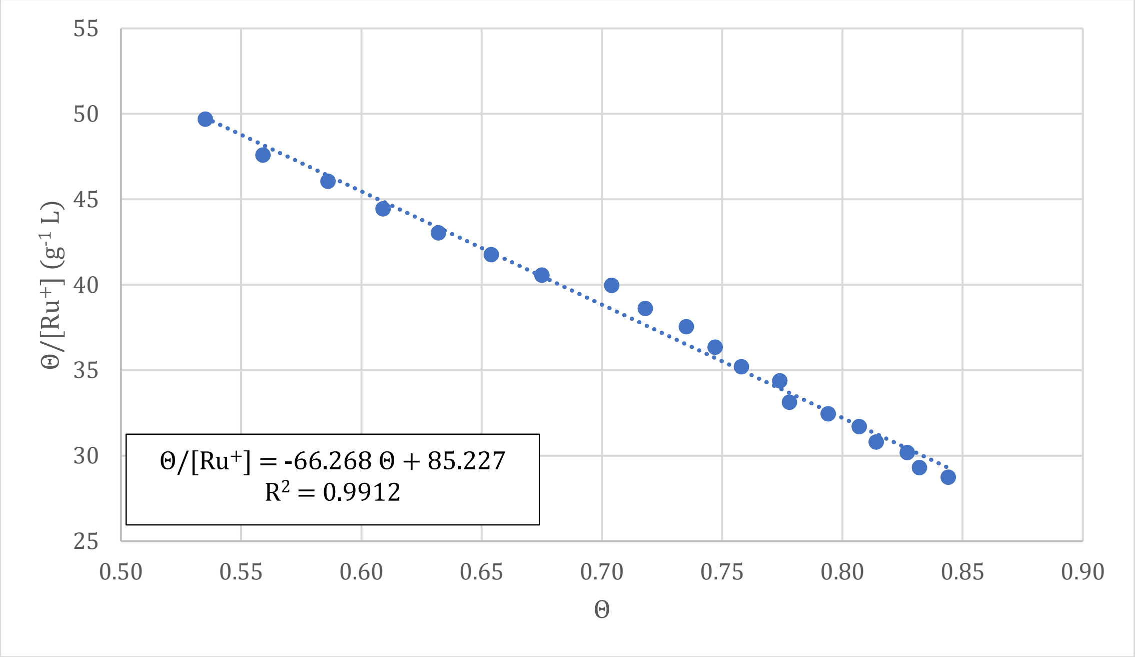

Figure S. 119: Scatchard isotherm for the interaction of the [RuCl(dppb)(bipy)(py)]PF_6 complex with $AuNPs^{n-}$ (second measure).

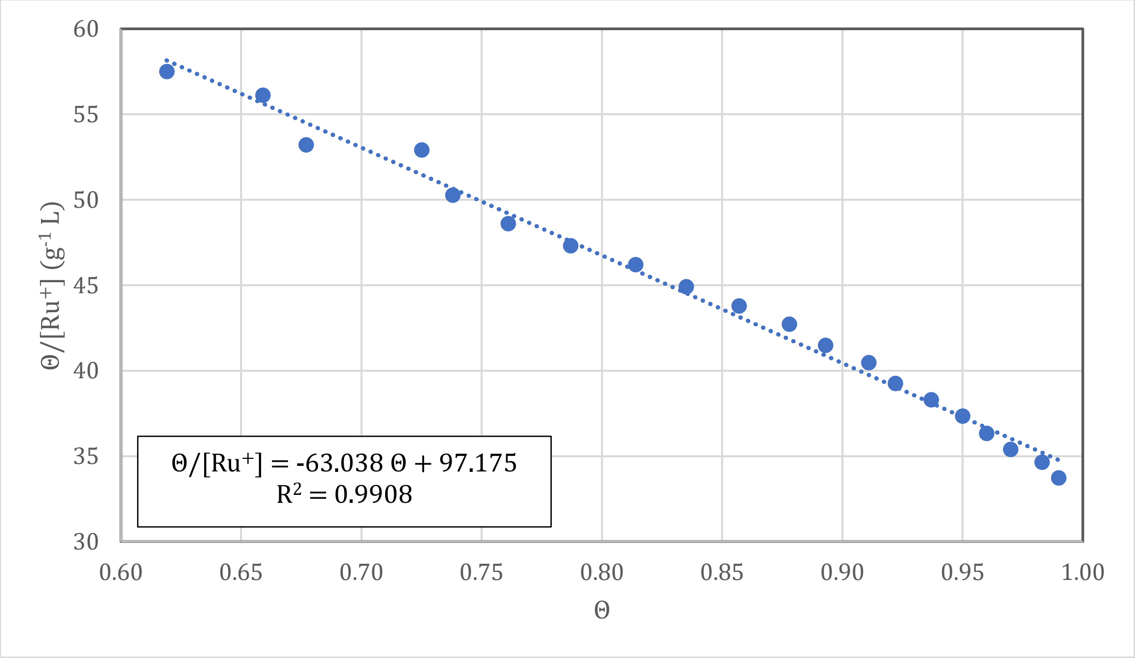

Figure S. 120 Scatchard isotherm for the interaction of the $[RuCl(dppb)(bipy)(py)]PF_6$ complex with $AuNPs^{z-}$ (third measure).

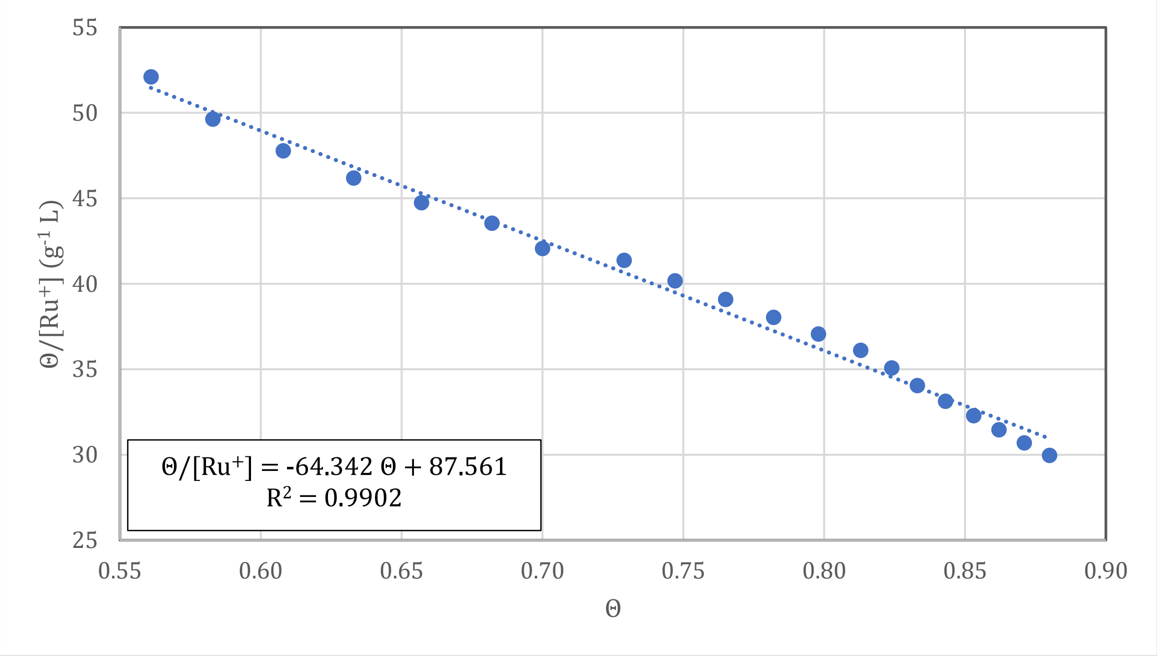

Figure S. 121: Scatchard isotherm for the interaction of the $[RuCl(dppb)(bipy)(tbpy)]PF_6$ complex with $AuNPs^{z-}$ (first measure).

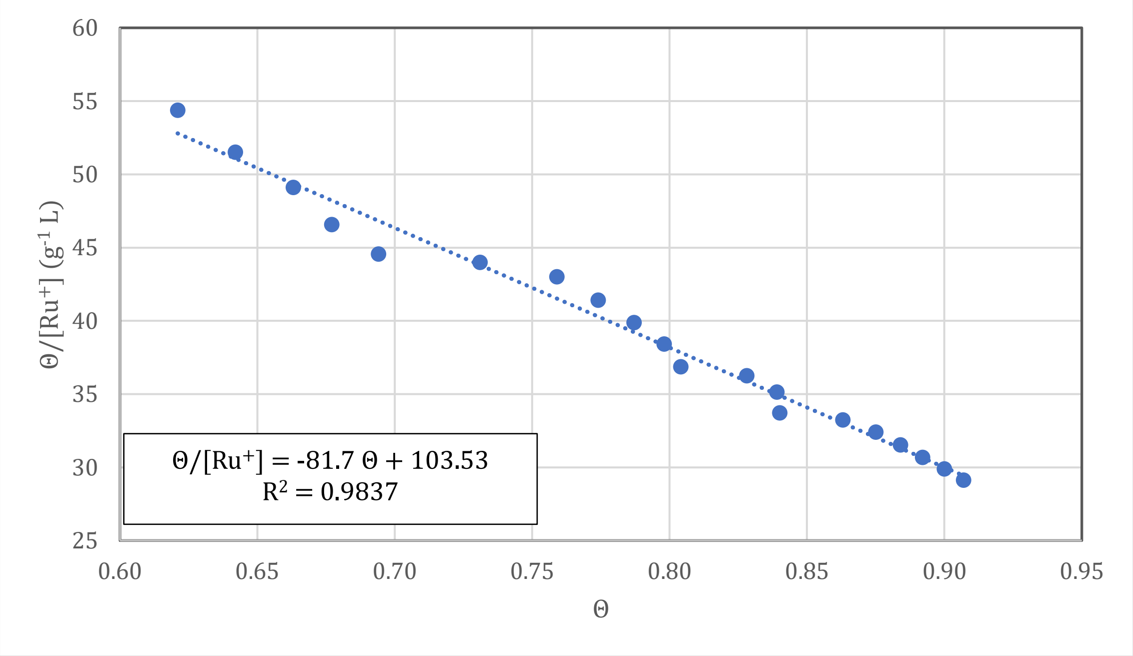

Figure S. 122: Scatchard isotherm for the interaction of the [RuCl(dppb)(bipy)(tbpy)]PF_6 complex with $AuNPs^{z-}$ (second measure).

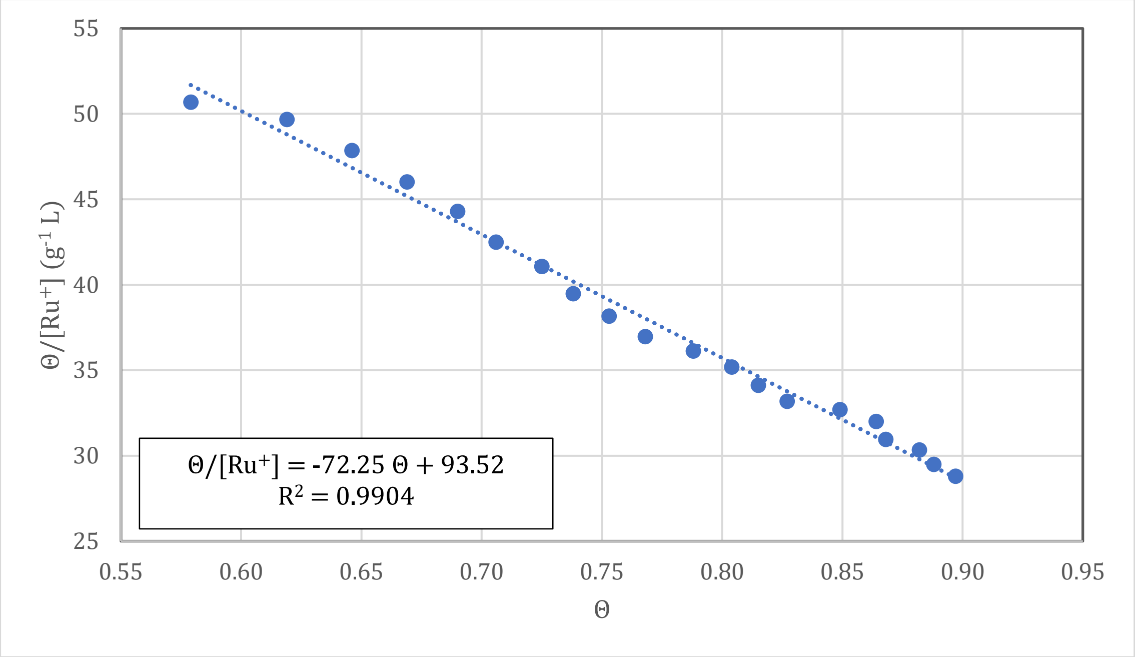

Figure S. 123: Scatchard isotherm for the interaction of the [RuCl(dppb)(bipy)(tbpy)]PF_6 complex with $AuNPs^{z-}$ (third measure).

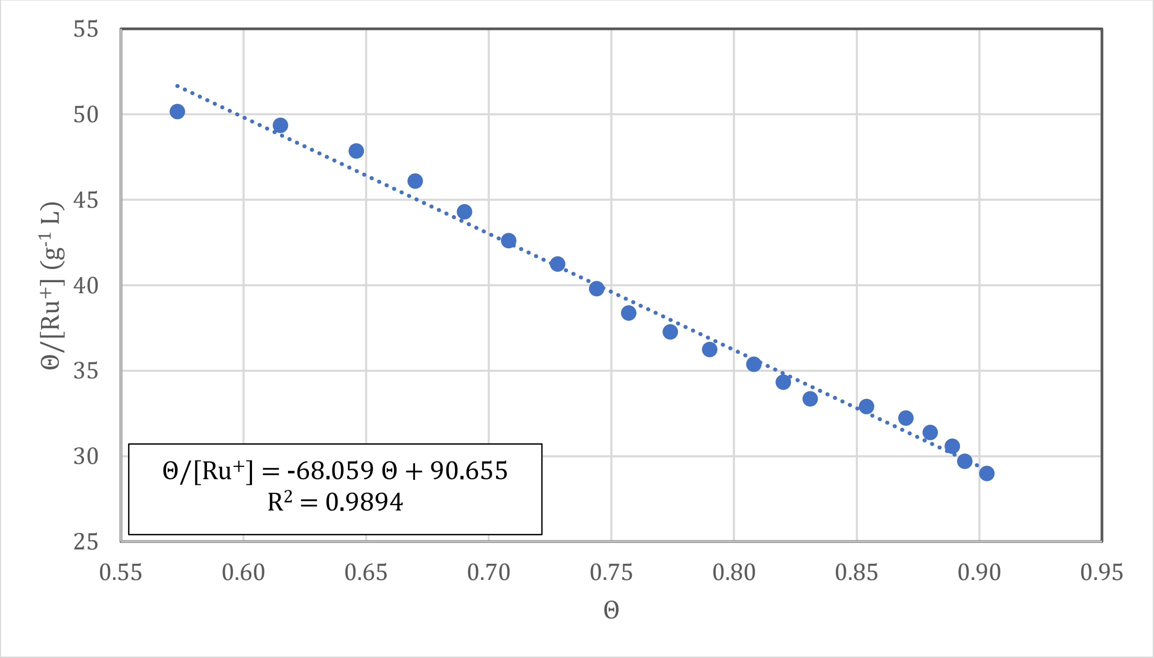

Figure S. 124: Scatchard isotherm for the interaction of the $[RuCl(dppb)(bipy)(mepy)]PF_6$ complex with $AuNPs^{z-}$ (first measure).

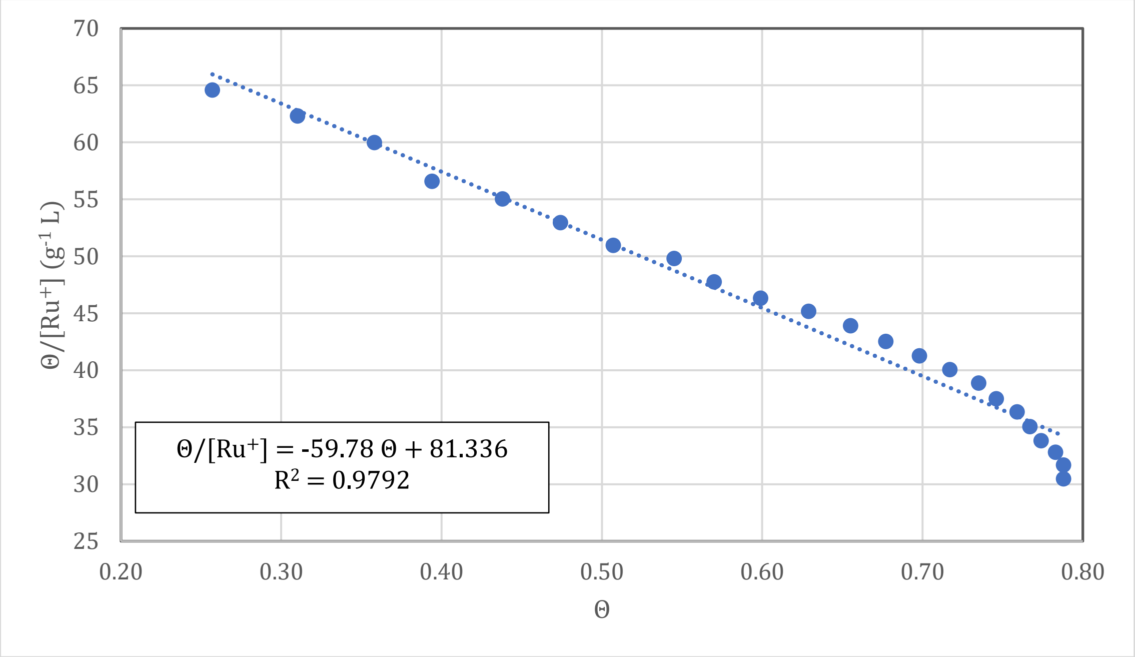

Figure S. 125: Scatchard isotherm for the interaction of the $[RuCl(dppb)(bipy)(mepy)]PF_6$ complex with $AuNPs^{z-}$ (second measure).

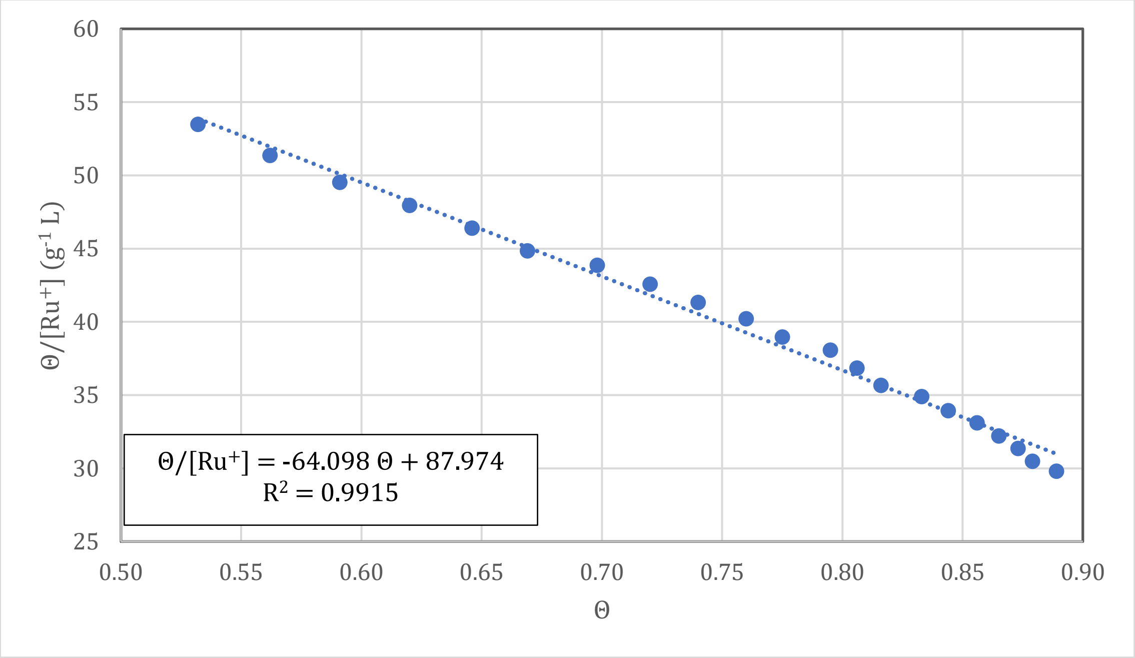

Figure S. 126: Scatchard isotherm for the interaction of the $[RuCl(dppb)(bipy)(mepy)]PF_6$ complex with $AuNPs^{z-}$ (third measure).

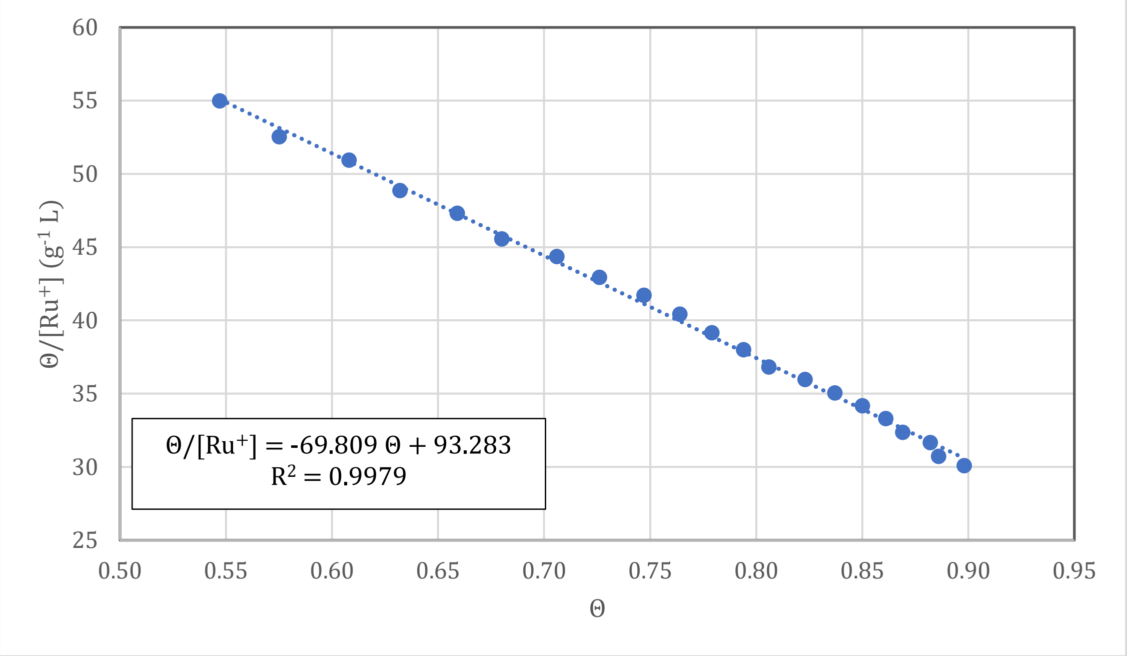

Figure S. 127: Scatchard isotherm for the interaction of the $[RuCl(dppb)(bipy)(vpy)]PF_6$ complex with $AuNPs^{z-}$ (first measure).

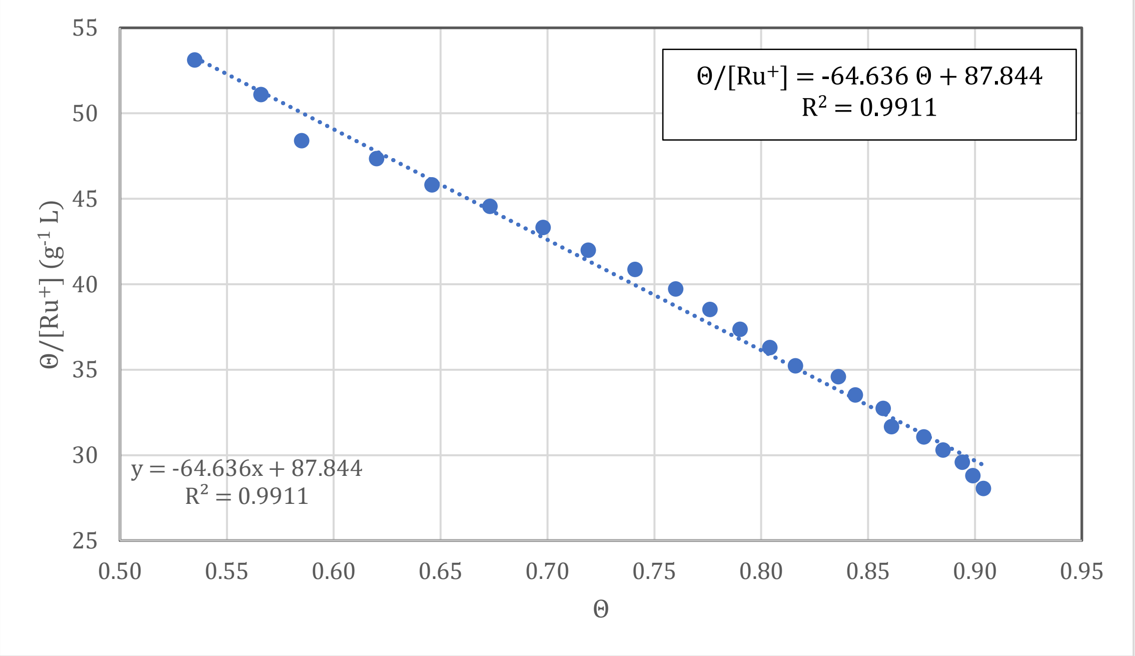

Figure S. 128: Scatchard isotherm for the interaction of the [RuCl(dppb)(bipy)(vpy)]PF_6 complex with $AuNPs^{z-}$ (second measure).

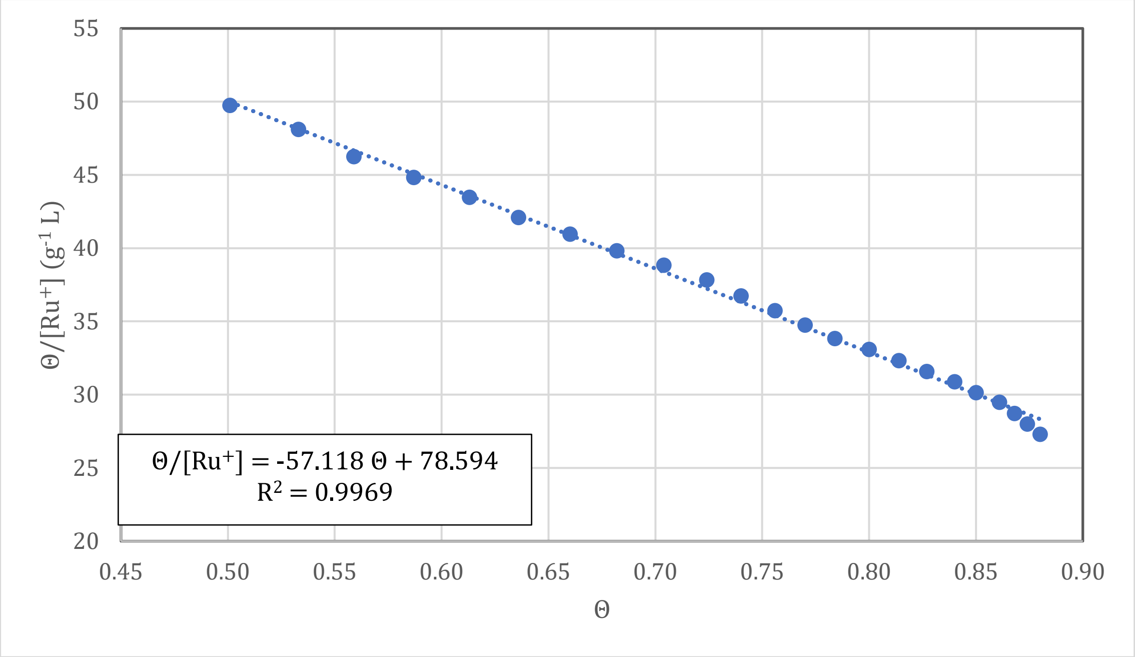

Figure S. 129: Scatchard isotherm for the interaction of the [RuCl(dppb)(bipy)(vpy)]PF_6 complex with $AuNPs^{z-}$ (third measure).

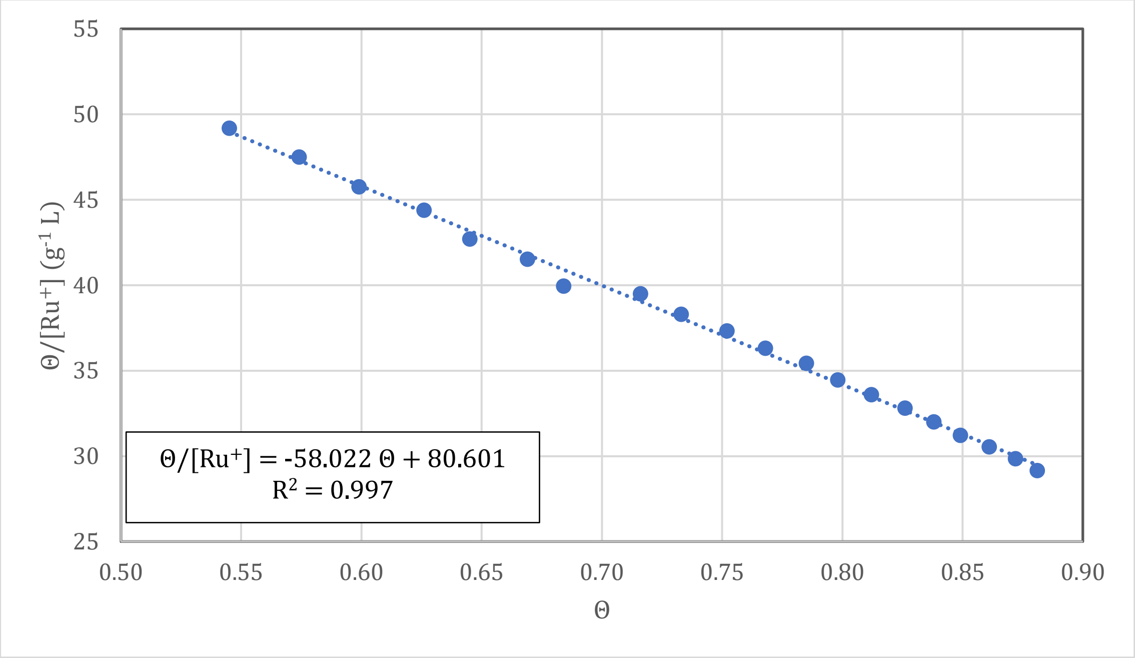

Figure S. 130: Scatchard isotherm for the interaction of the *cis*-[$RuCl_2$(dppb)(bipy)]PF_6 complex with $AuNPs^{z-}$ (first measure).

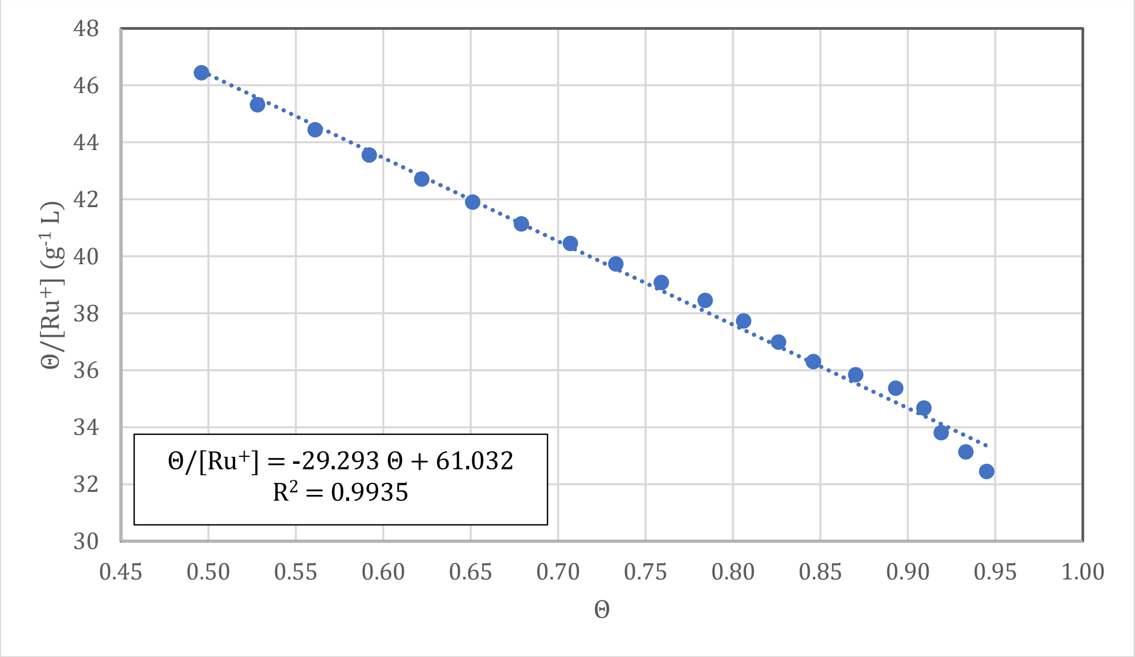

Figure S. 131: Scatchard isotherm for the interaction of the *cis*-[$RuCl_2$(dppb)(bipy)]PF_6 complex with $AuNPs^{z-}$ (second measure).

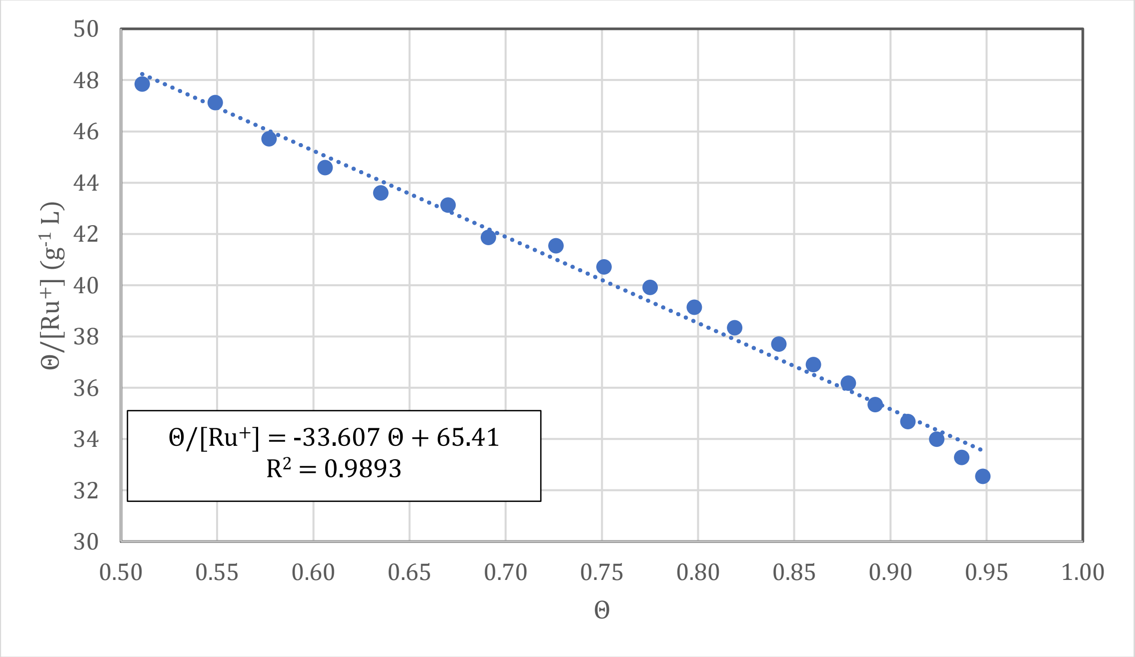

Figure S. 132: Scatchard isotherm for the interaction of the *cis*-$[RuCl_2(dppb)(bipy)]PF_6$ complex with $AuNPs^{z-}$ (third measure).

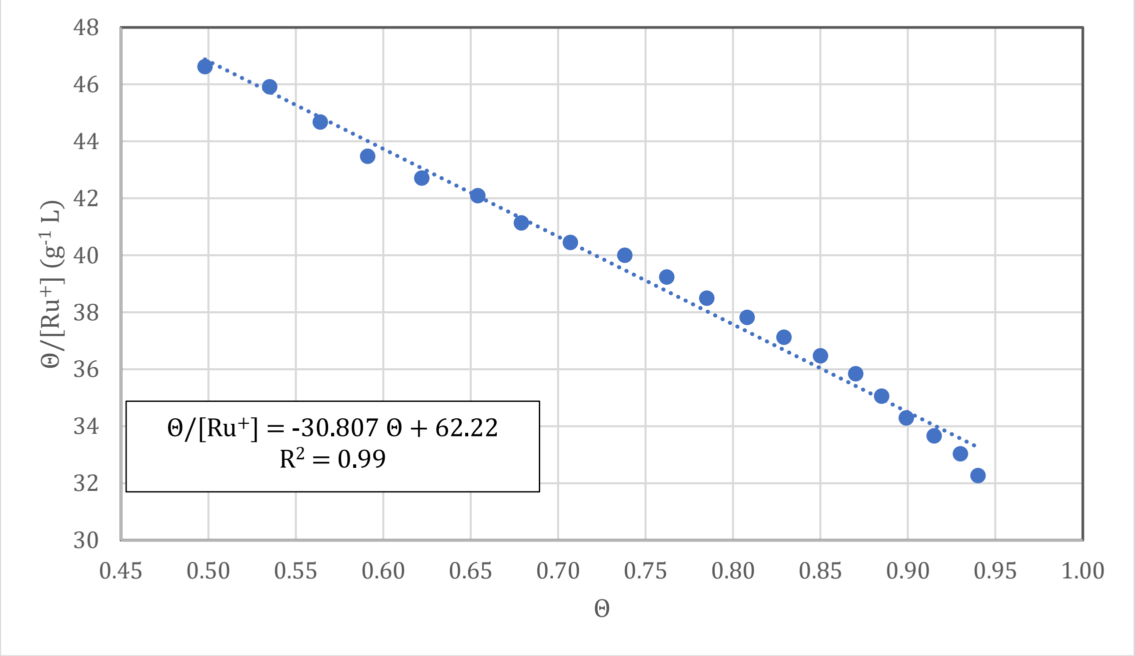

Figure S. 133: Scatchard isotherm for the interaction of the $\{TPyP[RuCl(dppb)(bipy)]_4\}(PF_6)_4$ complex with $AuNPs^{z-}$ (first measure).

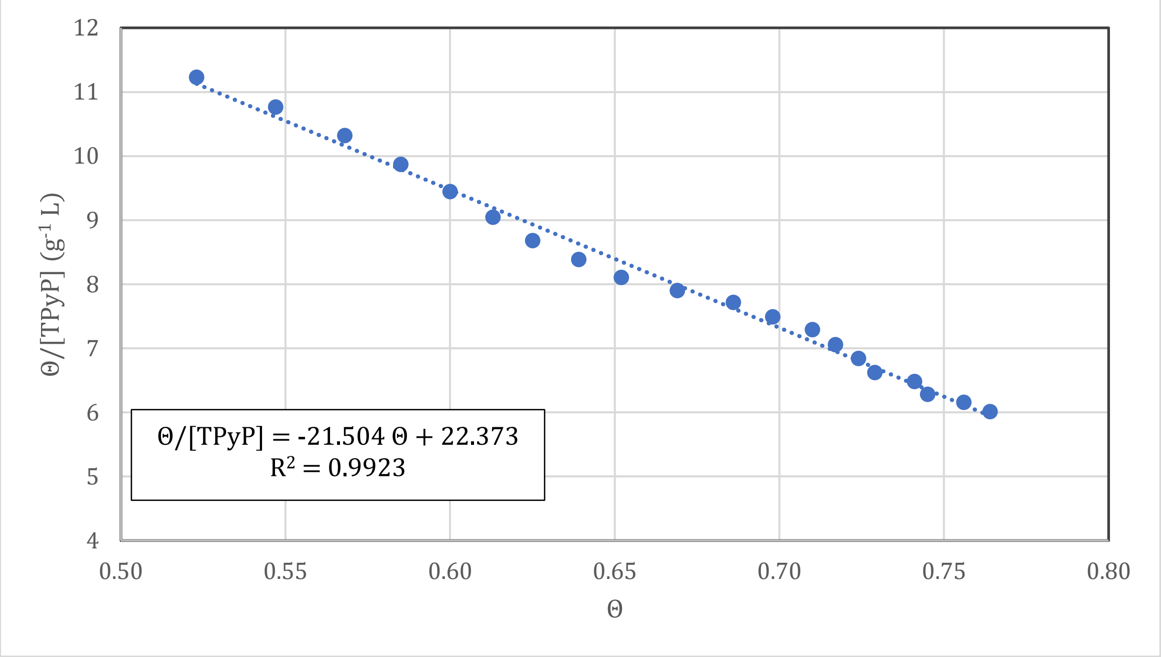

Figure S. 134: Scatchard isotherm for the interaction of the $\{TPyP[RuCl(dppb)(bipy)]_4\}(PF_6)_4$ complex with $AuNPs^{z-}$ (second measure).

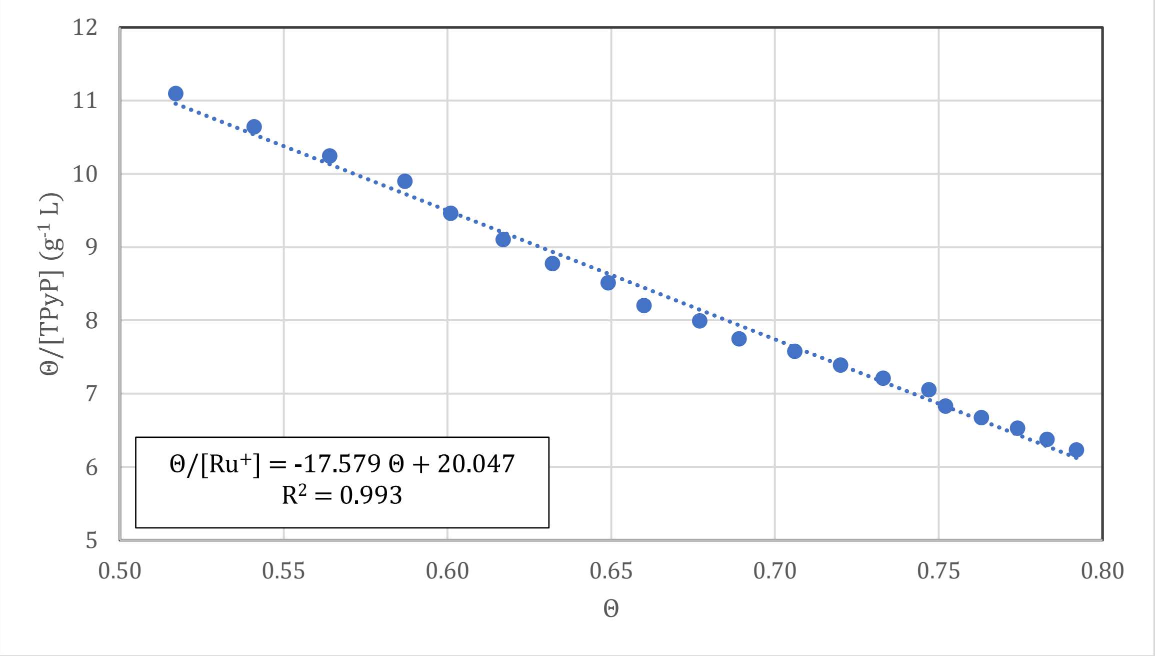

Figure S. 135: Scatchard isotherm for the interaction of the $\{TPyP[RuCl(dppb)(bipy)]_4\}(PF_6)_4$ complex with $AuNPs^{z-}$ (third measure).

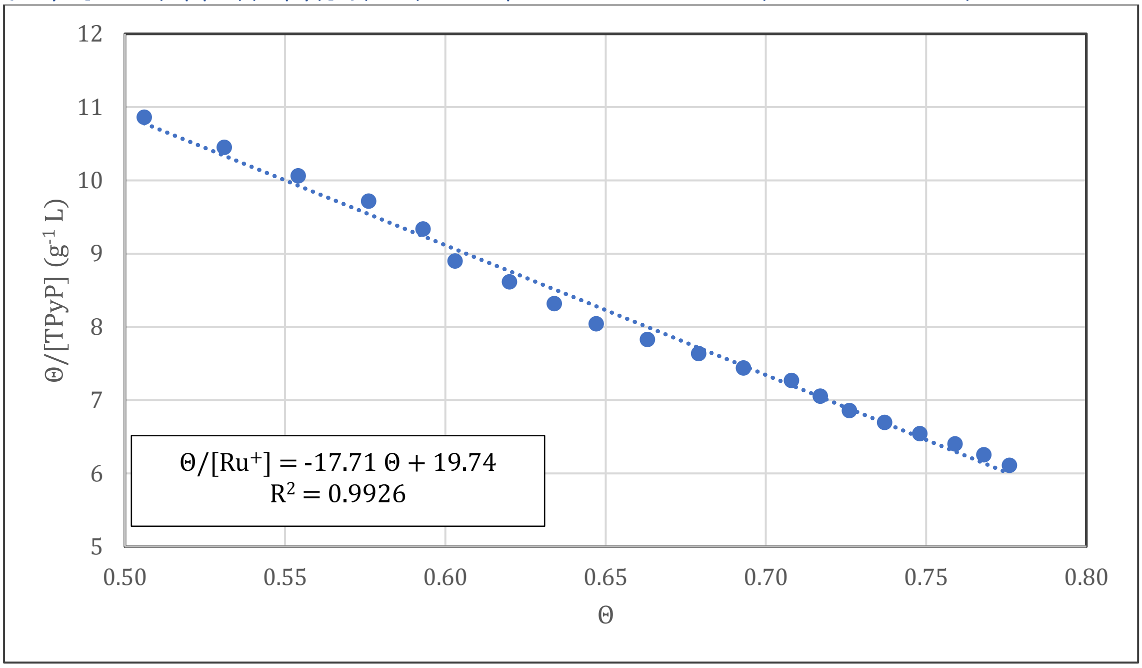

Figure S. 136: Scatchard isotherm for the interaction of the [RuCl(*p*-cymene)(Di*i*mpmp)(PF_6) complex with $AuNPs^{z-}$.

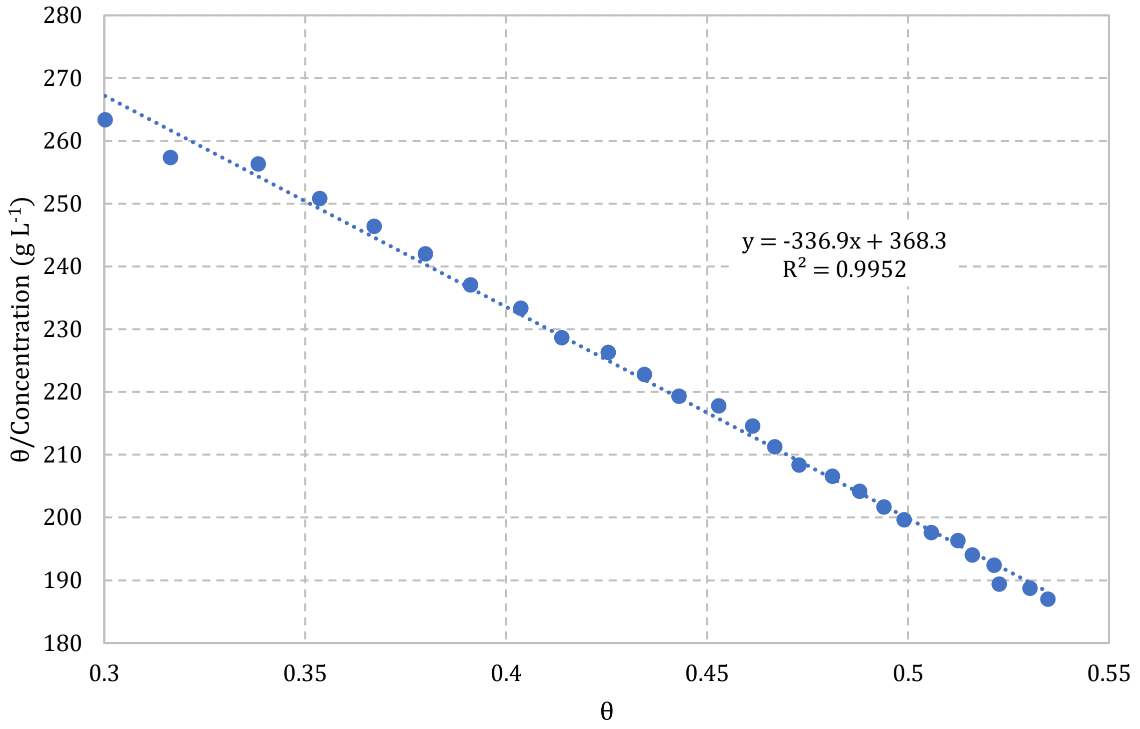

Approximate relative error for $M^{z+}$and $AuNPs^{z-}$ interactions

Figure S. 137: Relative error for the interaction of the $[Fe(bipy)_3]Cl_2$ complex with $AuNPs^{z-}$ (first measure).

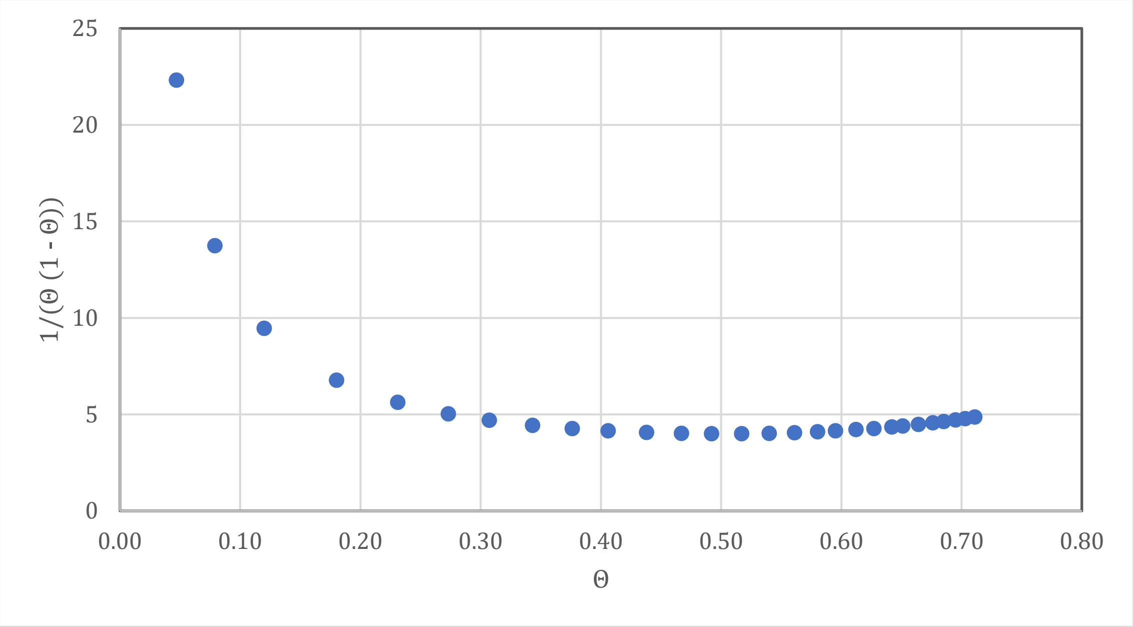

Figure S. 138: Relative error for the interaction of the $[Fe(bipy)_3]Cl_2$ complex with $AuNPs^{z-}$ (second measure).

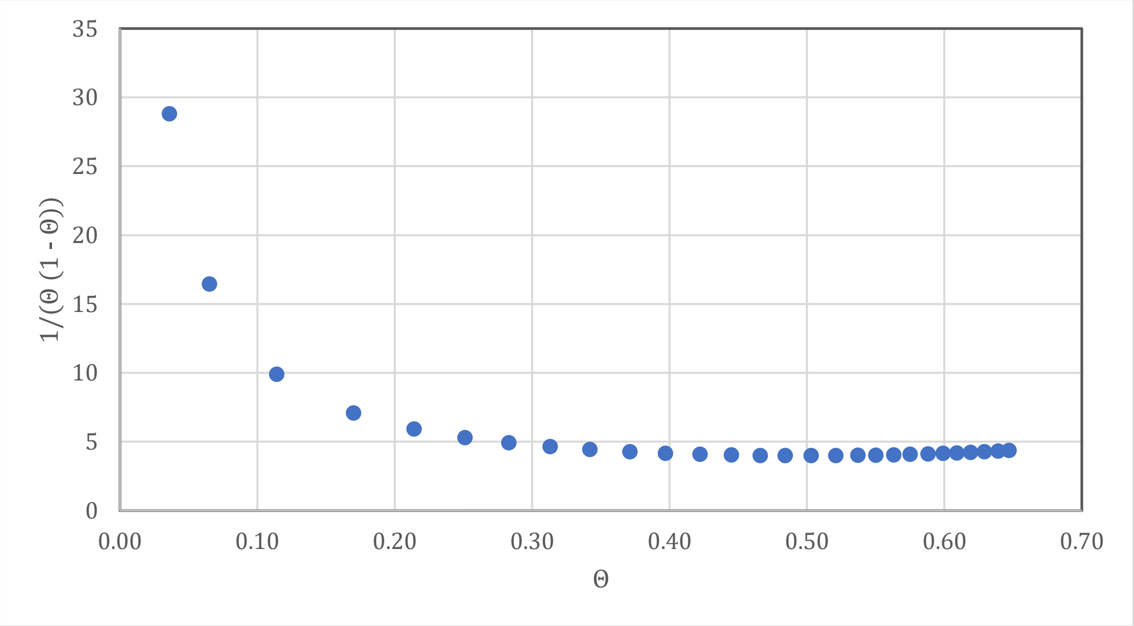

Figure S. 139: Relative error for the interaction of the $[Fe(bipy)_3]Cl_2$ complex with $AuNPs^{z-}$ (third measure).

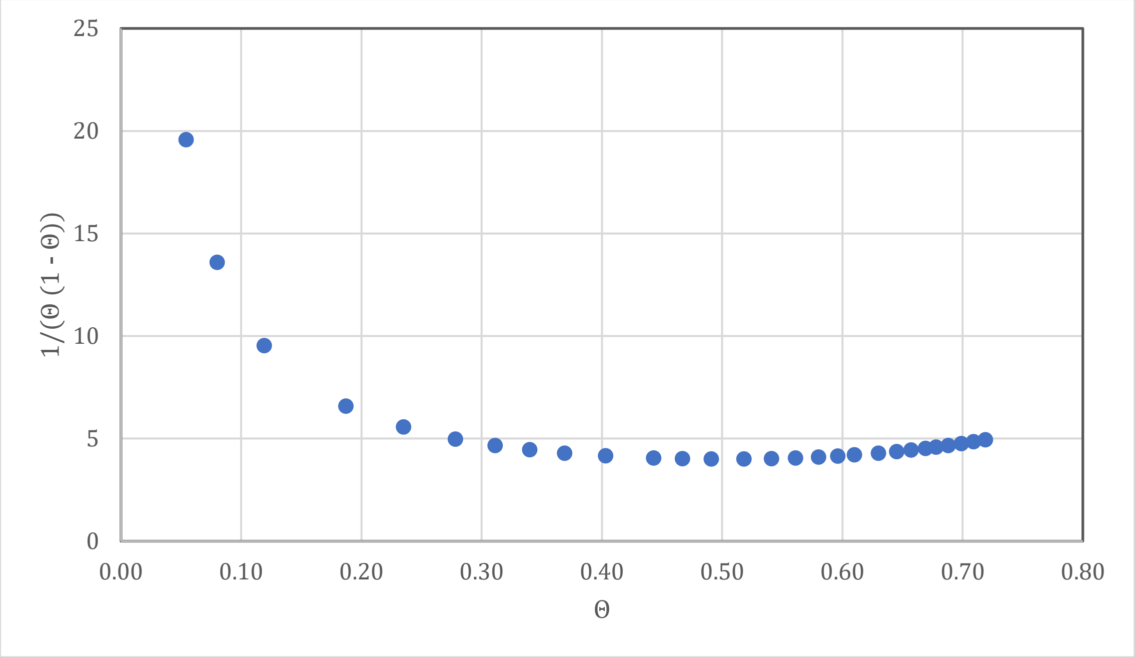

Figure S. 140: Relative error for the interaction of the $[Ru(bipy)_3](PF_6)_2$ complex with $AuNPs^{z-}$ (first measure).

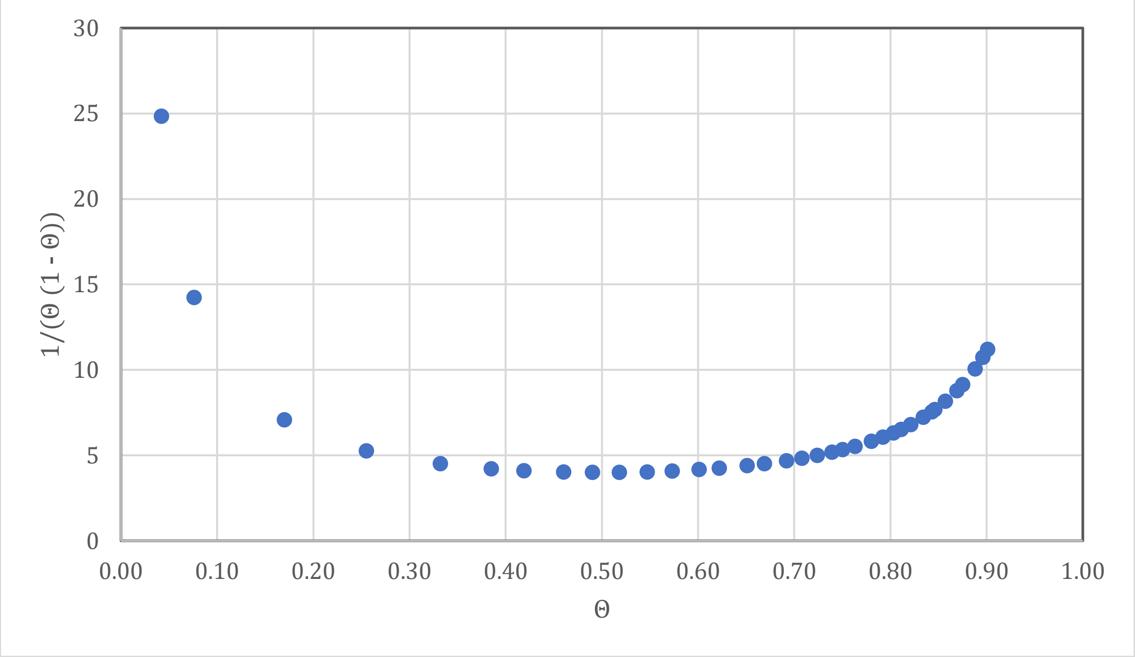

Figure S. 141: Relative error for the interaction of the $[Ru(bipy)_3](PF_6)_2$ complex with $AuNPs^{z-}$ (second measure).

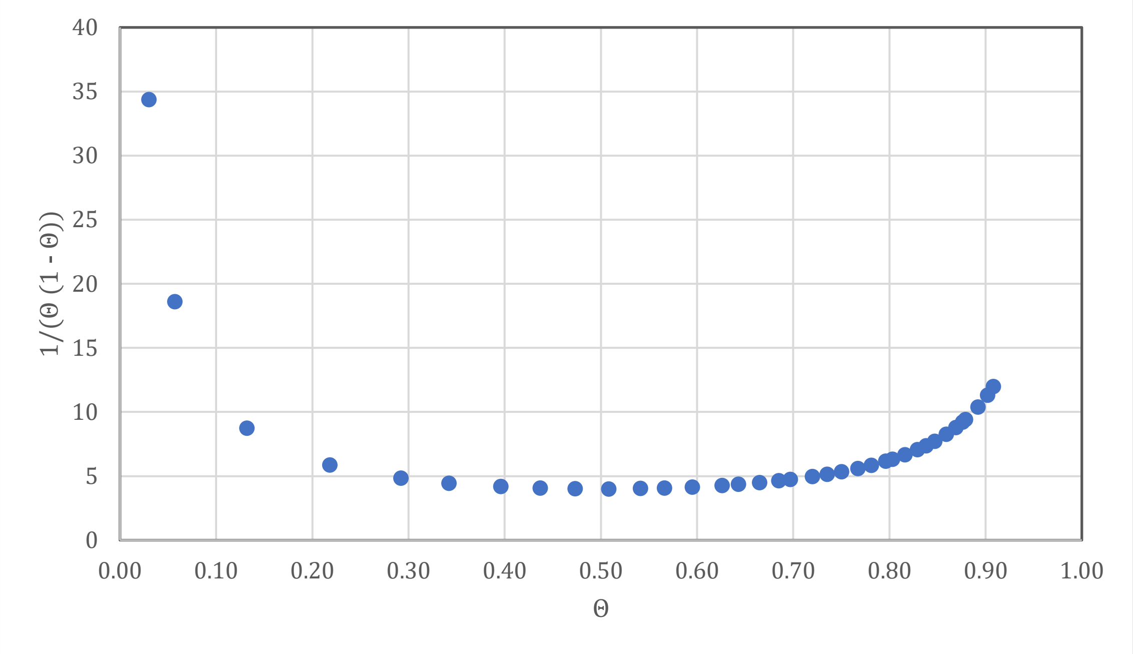

Figure S. 142: Relative error for the interaction of the $[Ru(bipy)_3](PF_6)_2$ complex with $AuNPs^{z-}$ (third measure).

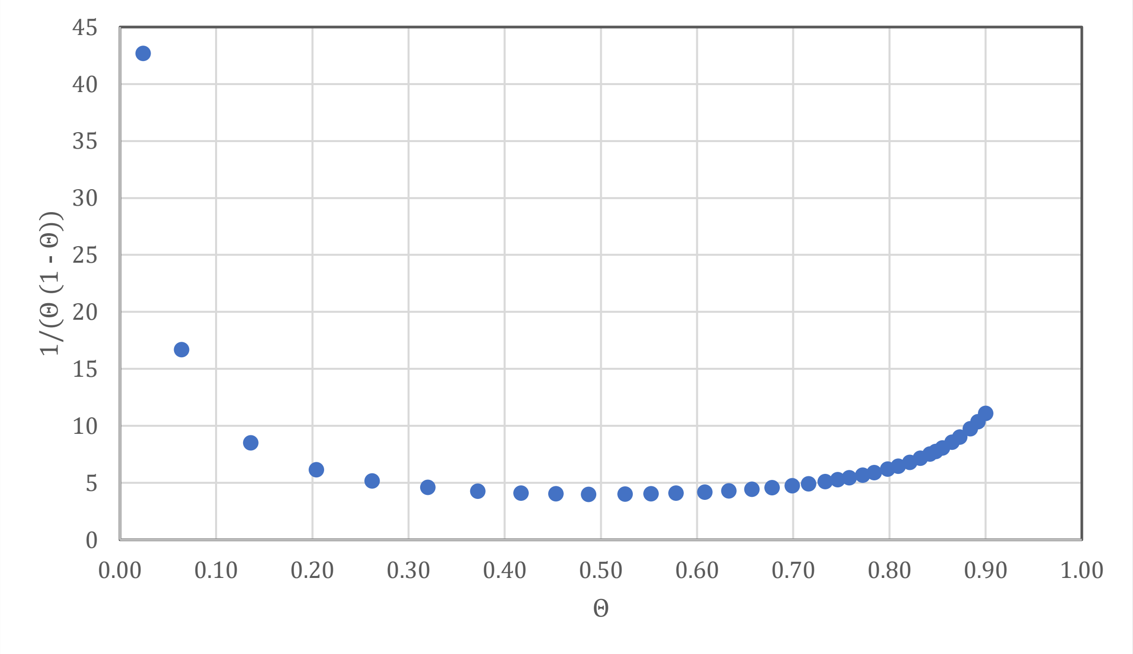

Figure S. 143: Relative error for the interaction of the [RuCl(dppb)(bipy)(py)](PF_6) complex with AuNPs^{z-} (first measure).

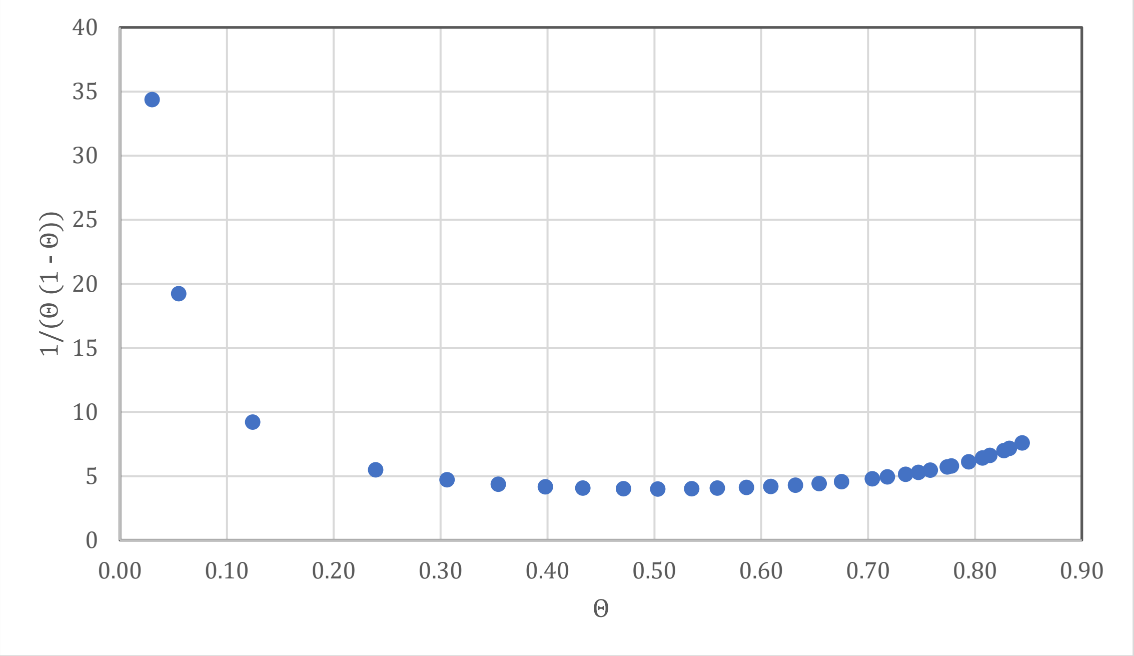

Figure S. 144: Relative error for the interaction of the [RuCl(dppb)(bipy)(py)](PF_6) complex with AuNPs^{z-} (second measure).

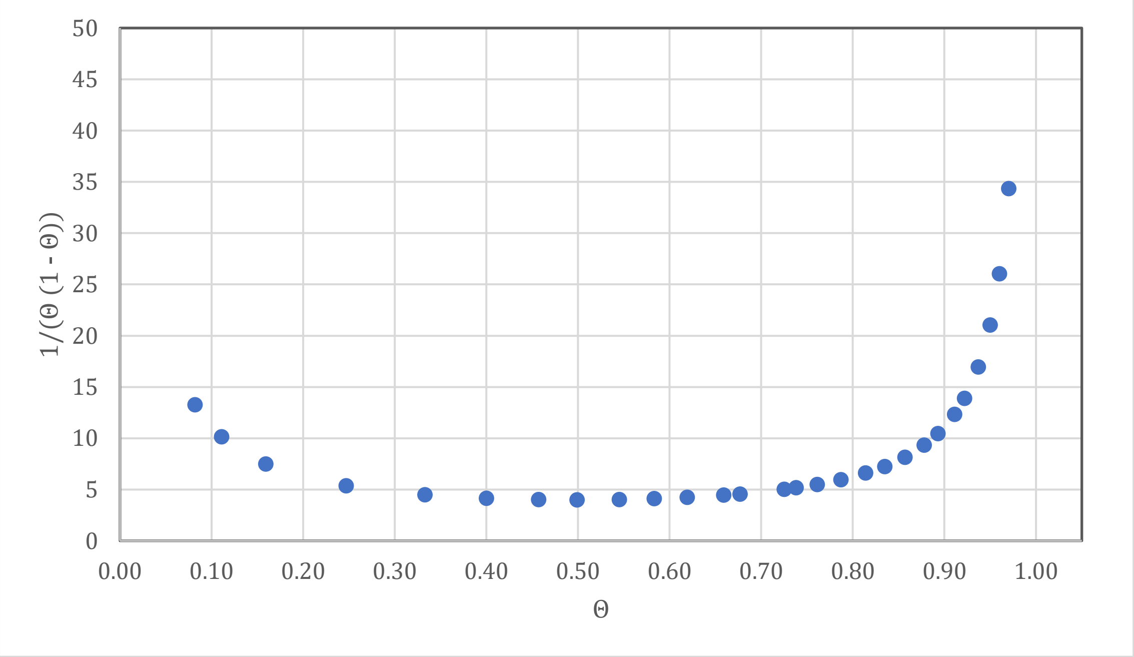

Figure S. 145: Relative error for the interaction of the [RuCl(dppb)(bipy)(py)](PF_6) complex with AuNPs^{z-} (third measure).

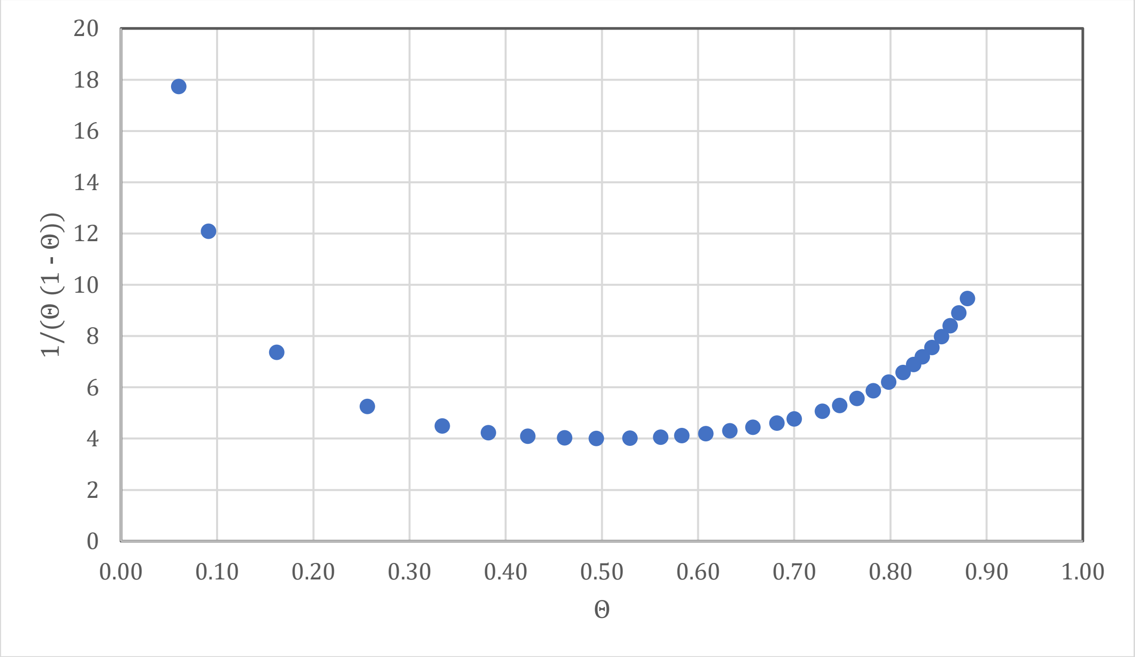

Figure S. 146: Relative error for the interaction of the [RuCl(dppb)(bipy)(tbpy)](PF_6) complex with AuNPs^{z-} (first measure).

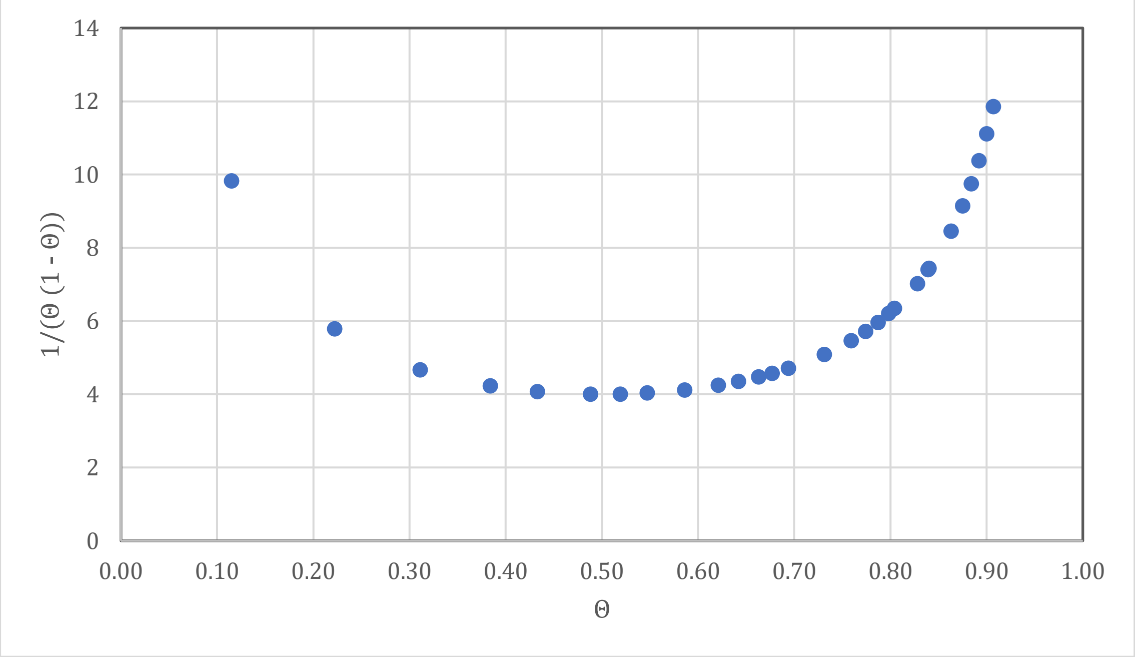

Figure S. 147: Relative error for the interaction of the [RuCl(dppb)(bipy)(*t*bpy)](PF_6) complex with $AuNPs^{z-}$ (second measure).

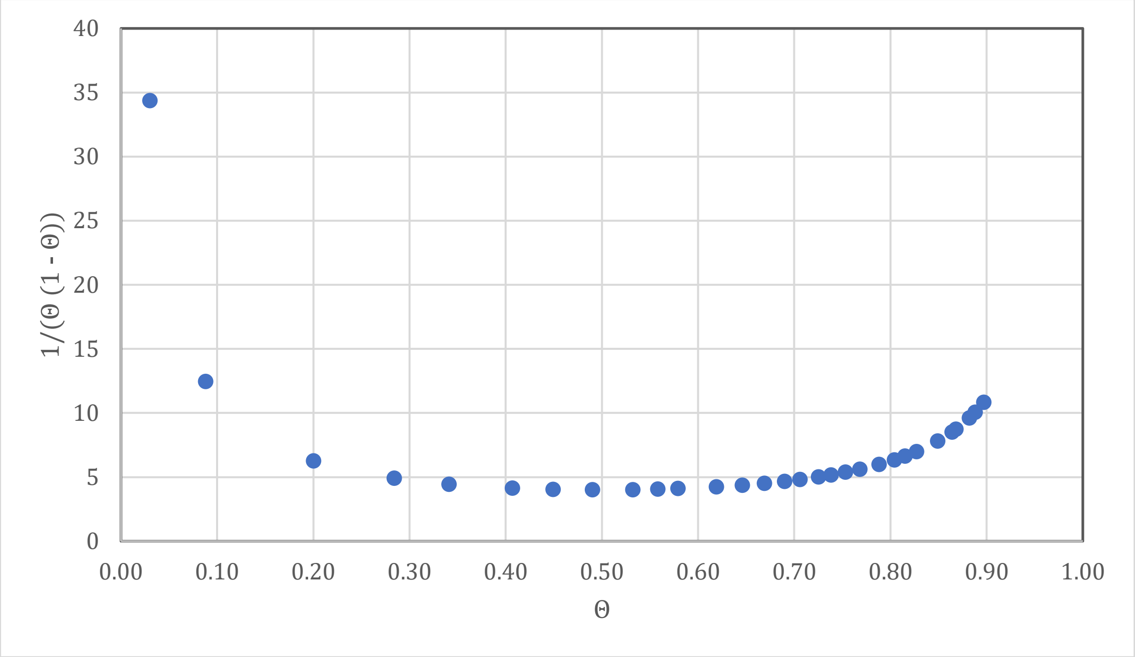

Figure S. 148: Relative error for the interaction of the [RuCl(dppb)(bipy)(*t*bpy)](PF_6) complex with $AuNPs^{z-}$ (third measure).

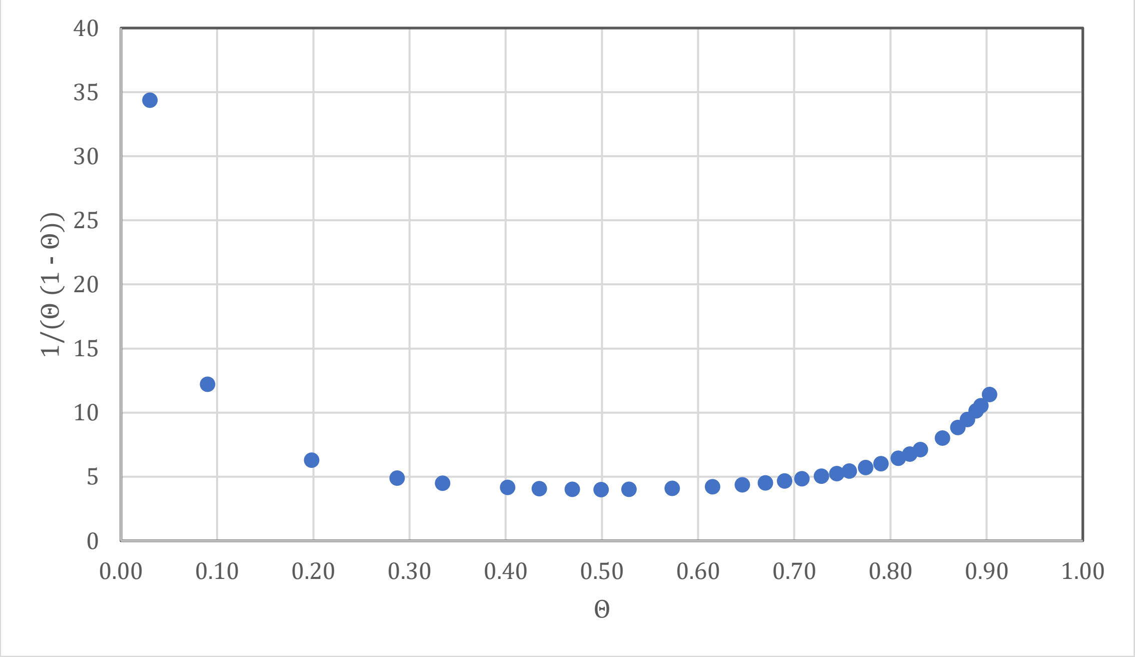

Figure S. 149: Relative error for the interaction of the [RuCl(dppb)(bipy)(mepy)](PF_6) complex with $AuNPs^{z-}$ (first measure).

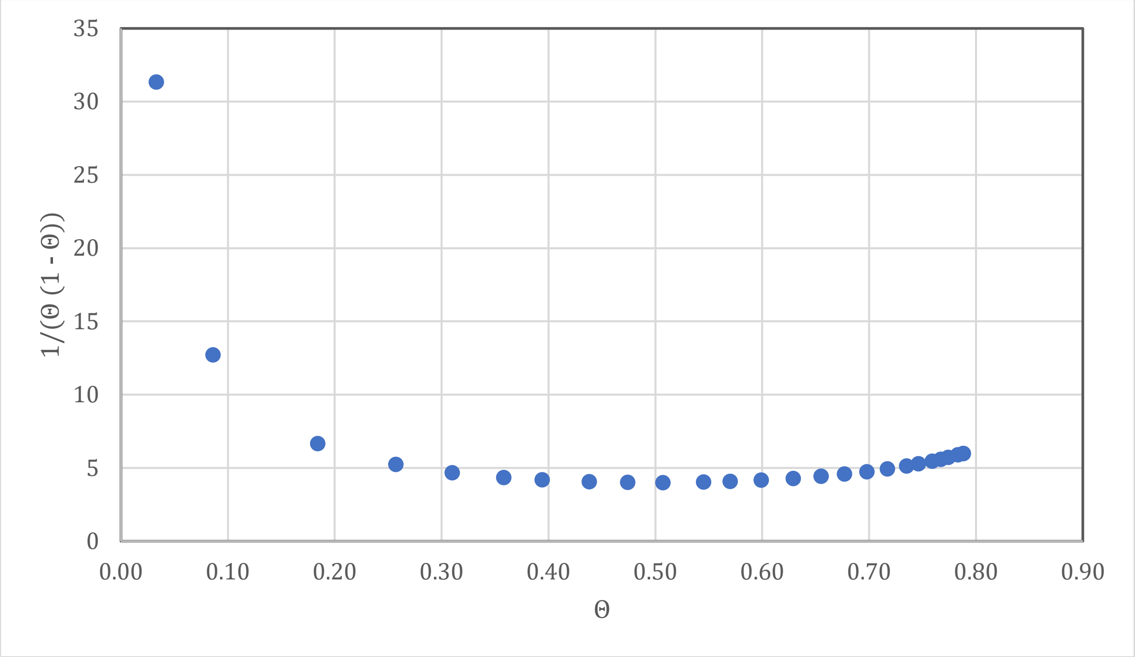

Figure S. 150: Relative error for the interaction of the [RuCl(dppb)(bipy)(mepy)](PF_6) complex with $AuNPs^{z-}$ (second measure).

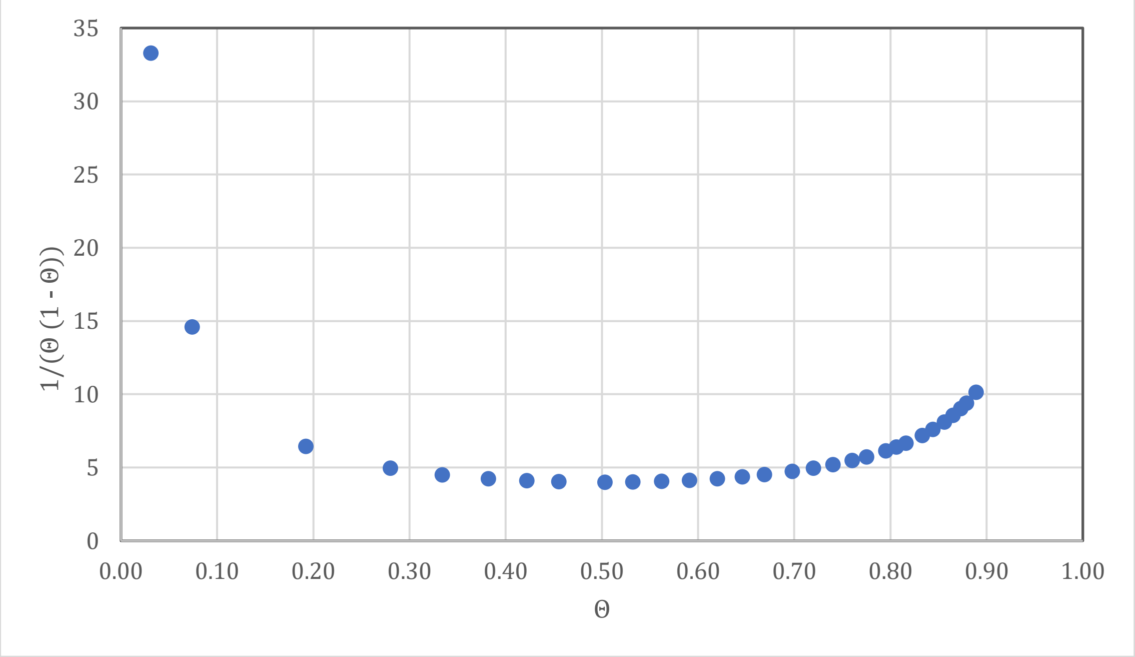

Figure S. 151: Relative error for the interaction of the $[RuCl(dppb)(bipy)(mepy)](PF_6)$ complex with $AuNPs^{z-}$ (third measure).

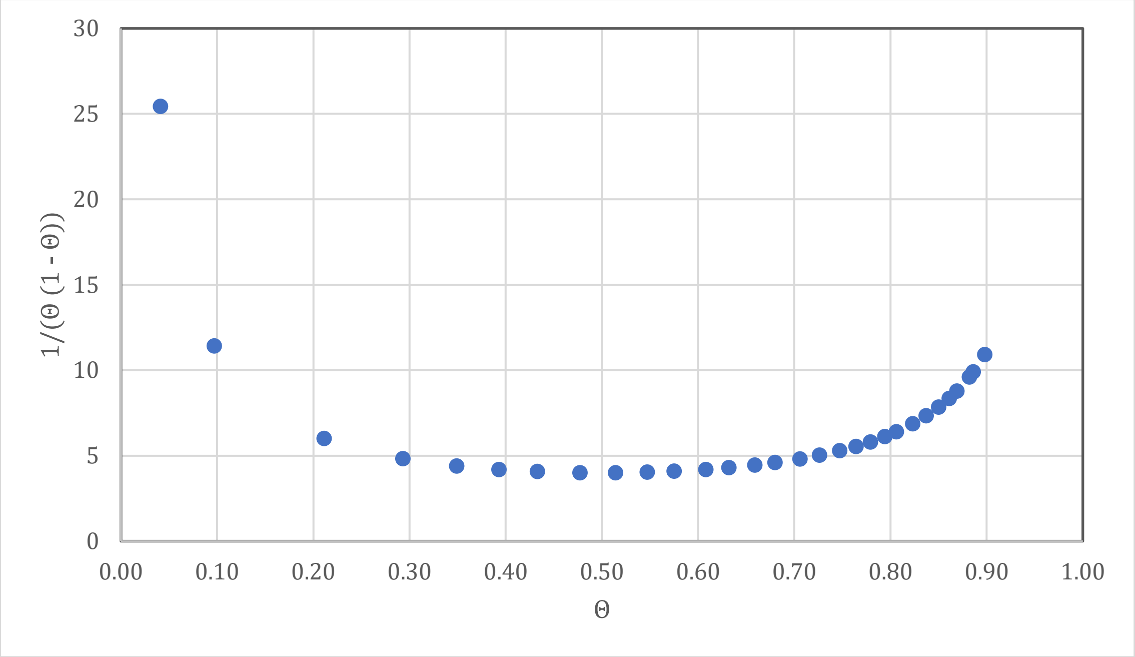

Figure S. 152: Relative error for the interaction of the $[RuCl(dppb)(bipy)(vpy)](PF_6)$ complex with $AuNPs^{z-}$ (first measure).

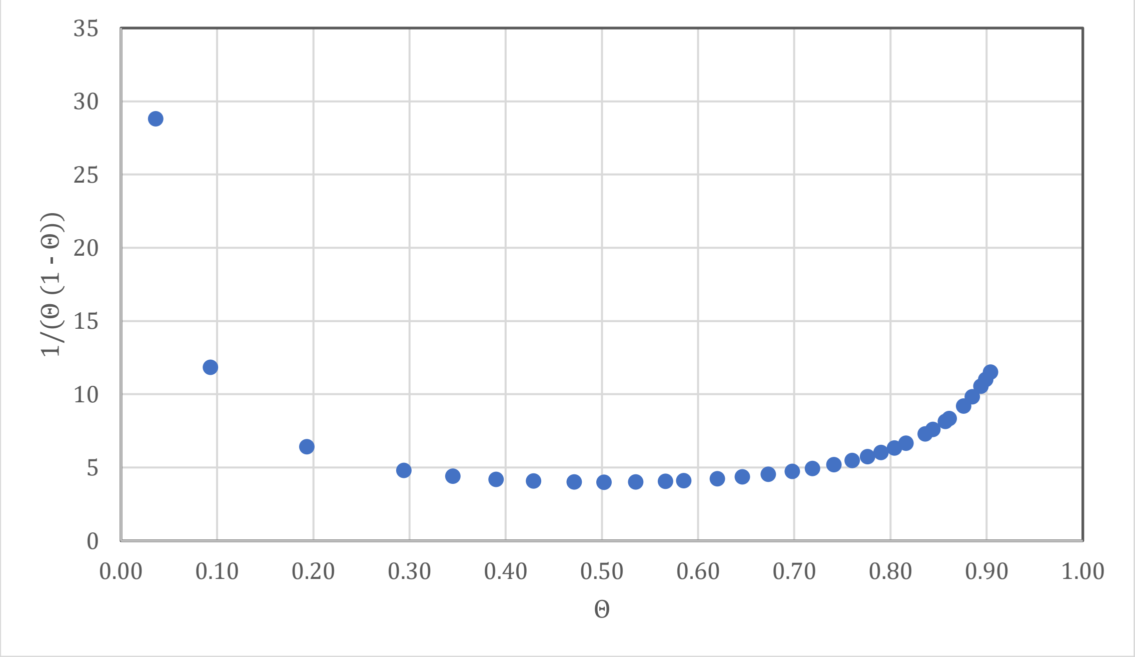

Figure S. 153: Relative error for the interaction of the [RuCl(dppb)(bipy)(vpy)](PF_6) complex with $AuNPs^{z-}$ (second measure).

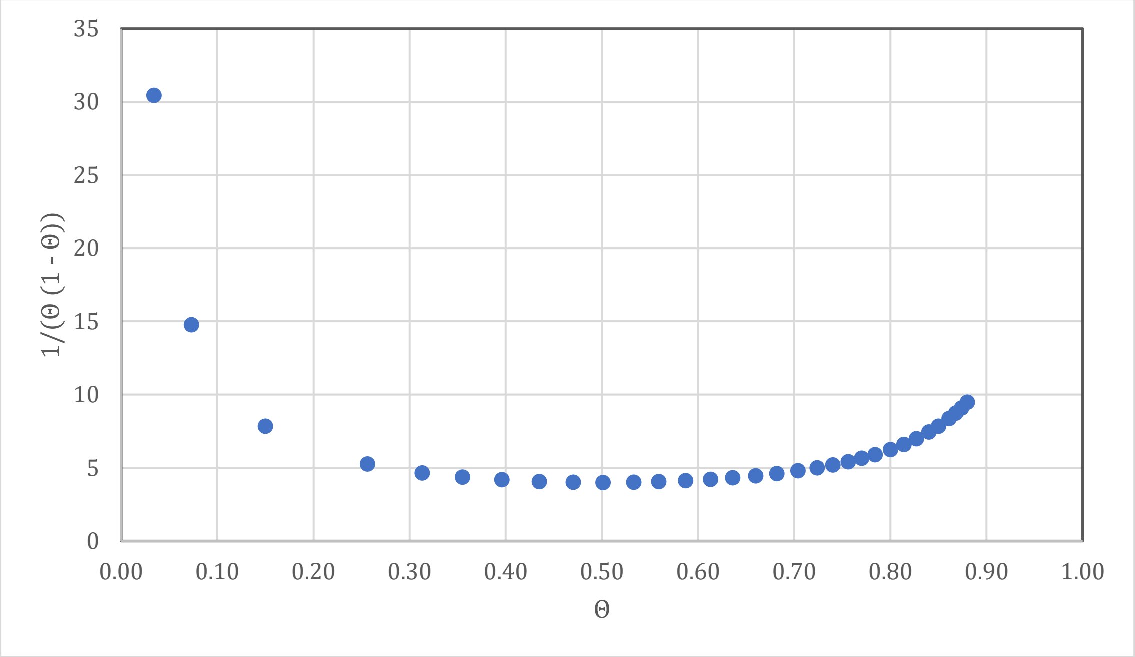

Figure S. 154: Relative error for the interaction of the [RuCl(dppb)(bipy)(vpy)](PF_6) complex with $AuNPs^{z-}$ (third measure).

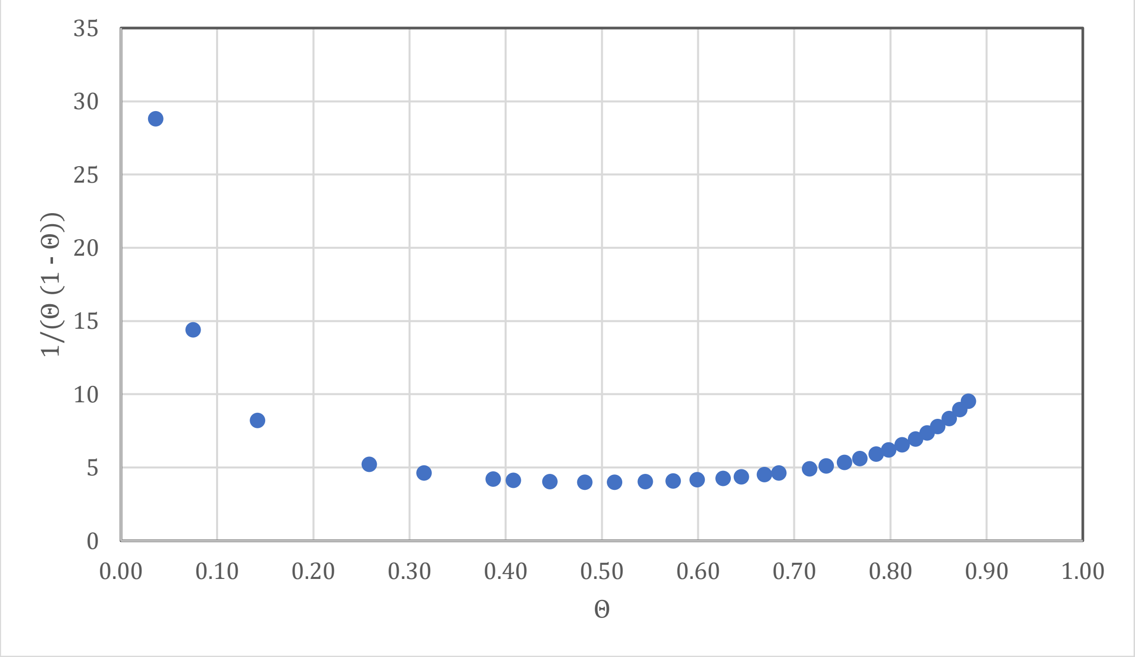

Figure S. 155: Relative error for the interaction of the *cis*-$[RuCl_2(dppb)(bipy)](PF_6)$ complex with $AuNPs^{z-}$ (first measure).

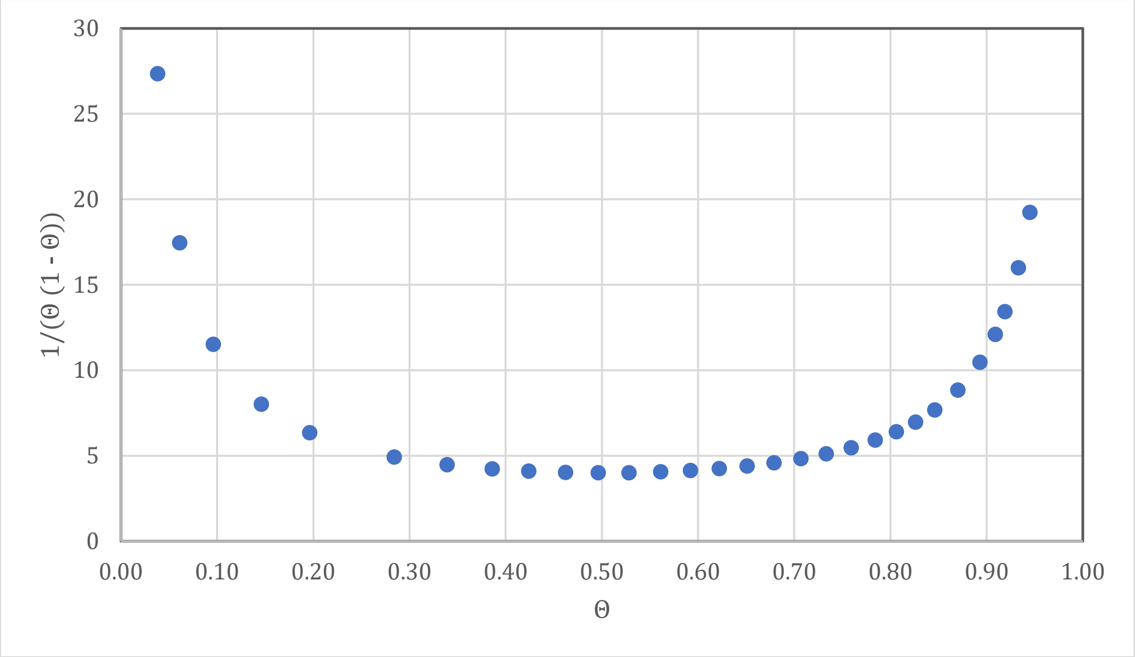

Figure S. 156: Relative error for the interaction of the *cis*-$[RuCl_2(dppb)(bipy)](PF_6)$ complex with $AuNPs^{z-}$ (second measure).

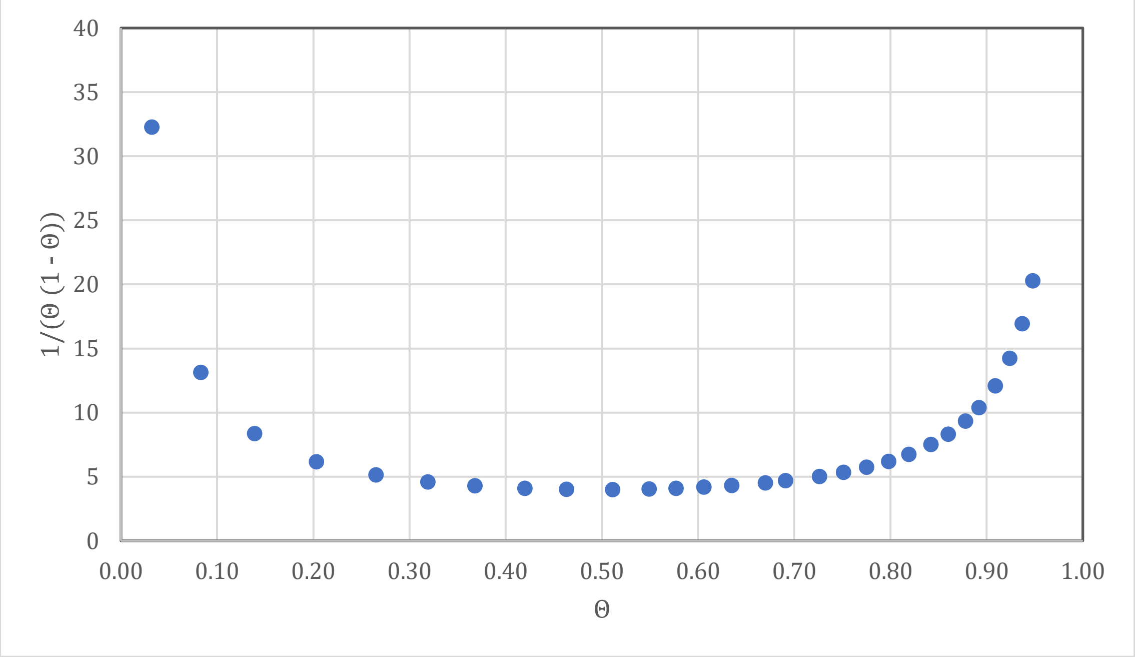

Figure S. 157: Relative error for the interaction of the *cis*-[$RuCl_2$(dppb)(bipy)](PF_6) complex with $AuNPs^{z-}$ (third measure).

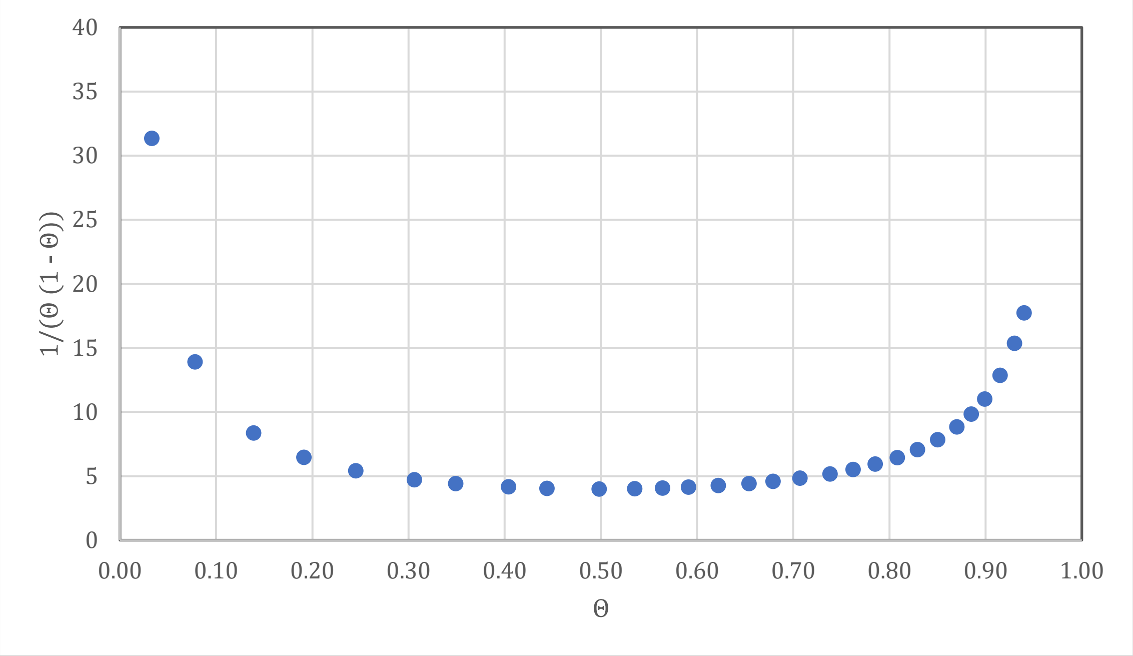

Figure S. 158: Relative error for the interaction of the {TPyP[RuCl(dppb)(bipy)]$_4$}(PF_6)$_4$ complex with $AuNPs^{z-}$ (first measure).

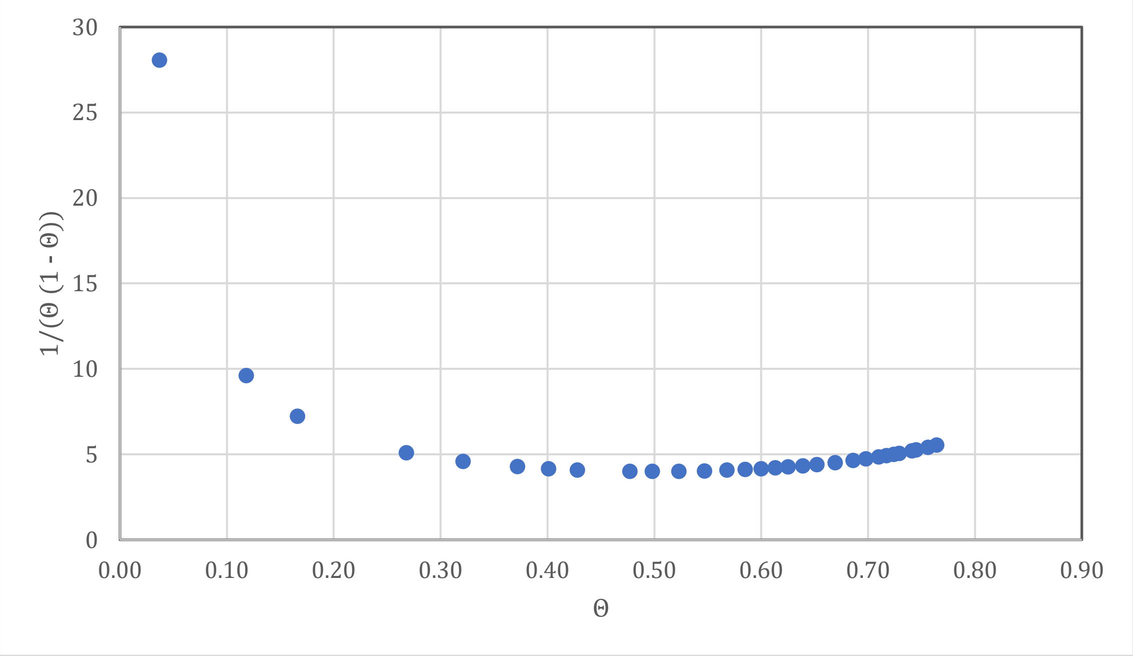

Figure S. 159: Relative error for the interaction of the $\{TPyP[RuCl(dppb)(bipy)]_4\}(PF_6)_4$ complex with $AuNPs^{z-}$ (second measure).

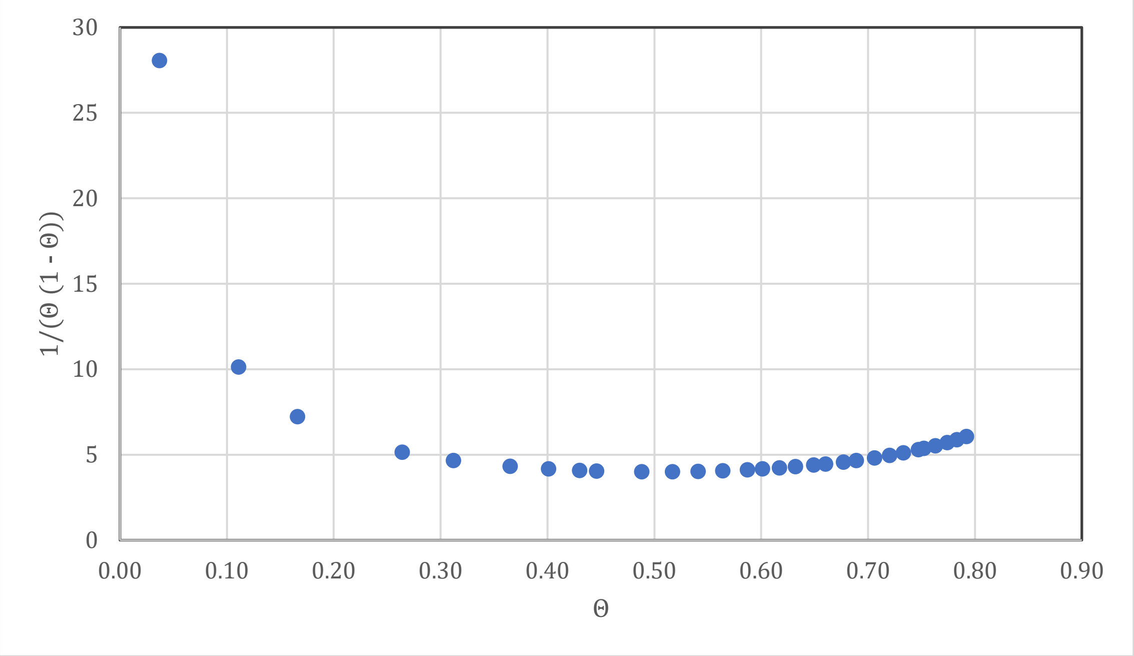

Figure S. 160: Relative error for the interaction of the $\{TPyP[RuCl(dppb)(bipy)]_4\}(PF_6)_4$ complex with $AuNPs^{z-}$ (third measure).

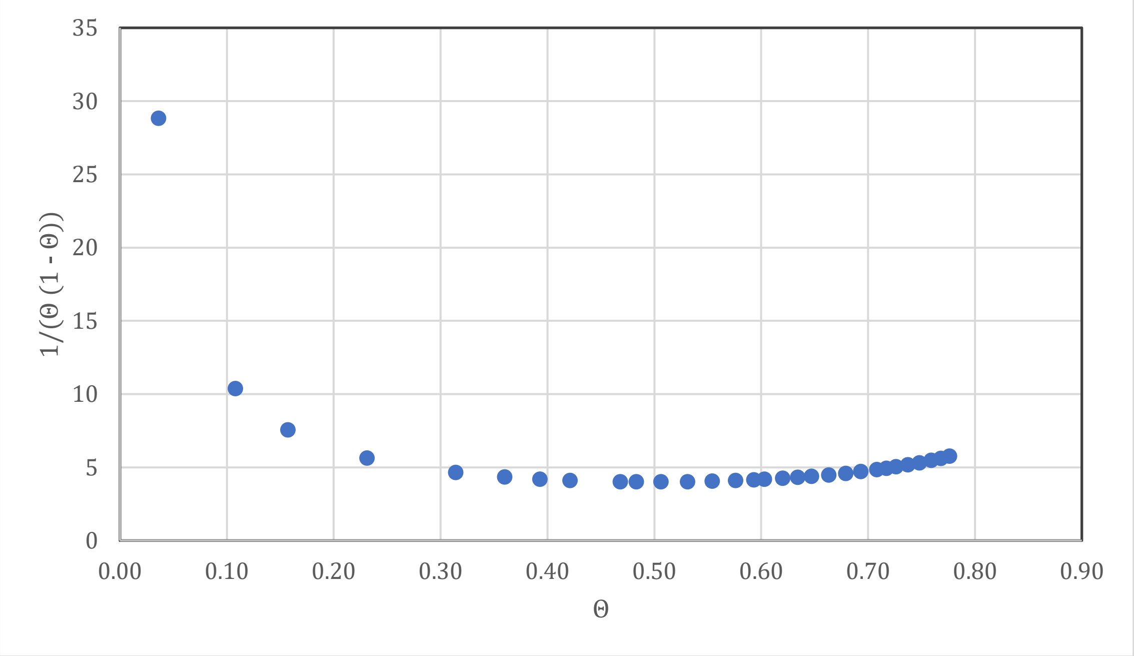

Figure S. 161: Relative error for the interaction of the [RuCl(*p*-cymene)(Di*i*pmp)](PF_6) complex with $AuNPs^{z-}$.

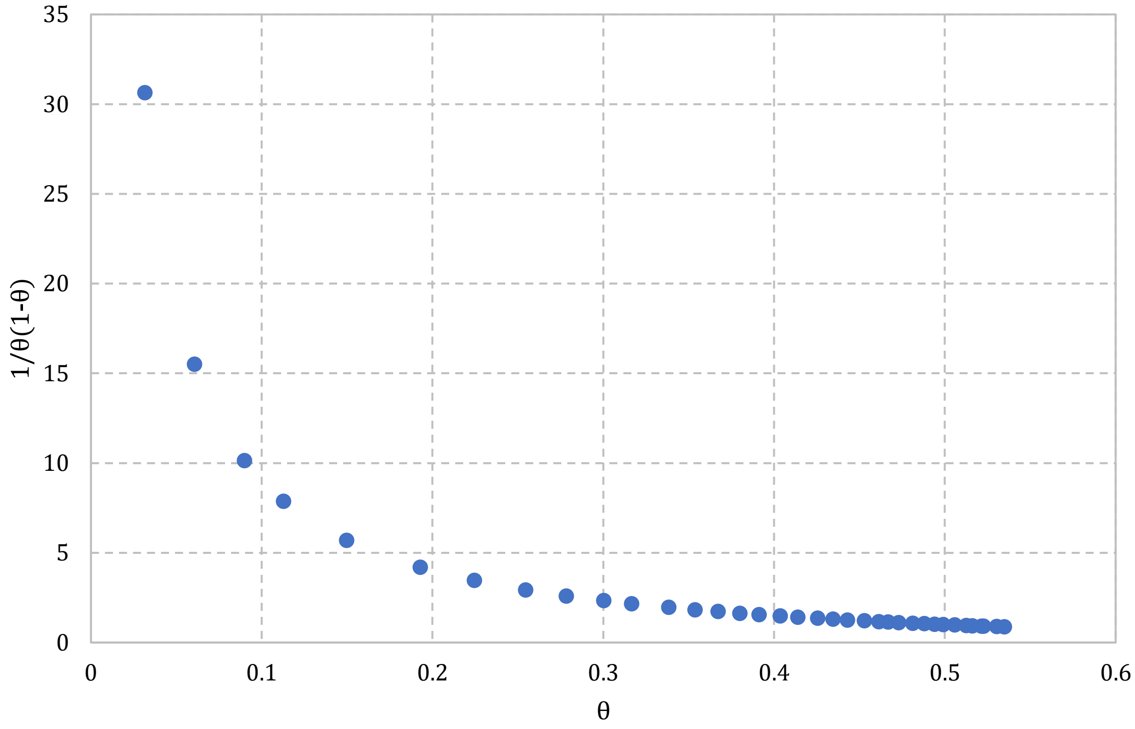

Kinetic of 4-nitrophenol reduction

Table S. 10: Rate constant and half-life at the temperature range between 25 – 50 ºC.

Temperature (ºC)	k_{obs} (s^{-1}) × 10^{-3}	SD × 10^{-4}	$t_{1/2}$ (s)
25.0	1.47	± 1.52	472
30.0	3.53	± 2.25	196
35.0	3.67	± 3.80	189
40.0	1.66	± 4.32	418
45.0	1.66	± 4.71	418
50.0	1.63	± 4.49	425

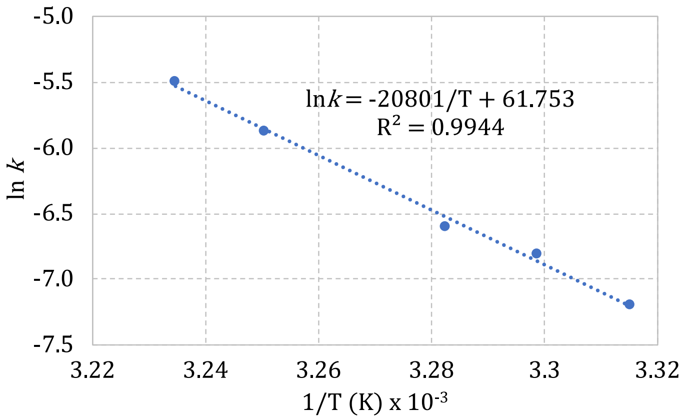

Figure S. 162: Arrhenius plot at the temperature range between 301.65K - 309.15K using $AuNPs^{z-}$ as catalyst on the reduction of 4-NP by $NaHB_4$.

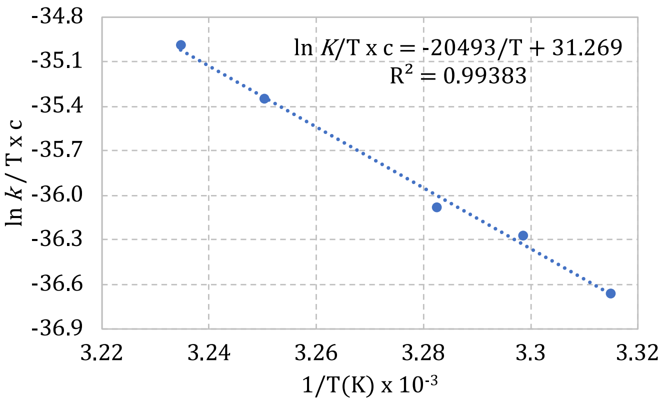

Figure S. 163: Eyring plot at the temperature range between 301.65K - 309.15K using $AuNPs^{z-}$ as catalyst on the reduction of 4-NP by $NaHB_4$.
